# Future Tense and Economic Decisions: Controlling for Cultural Evolution

**DOI:** 10.1371/journal.pone.0132145

**Published:** 2015-07-17

**Authors:** Seán G. Roberts, James Winters, Keith Chen

**Affiliations:** 1 Language and Cognition department, Max Planck Institute for Psycholinguistics, Nijmegen, Netherlands; 2 Language Evolution and Computation Research Unit, University of Edinburgh, Edinburgh, United Kingdom; 3 UCLA Anderson School of Management, Los Angeles, CA, United States of America; University of California, Merced, UNITED STATES

## Abstract

A previous study by Chen demonstrates a correlation between languages that grammatically mark future events and their speakers' propensity to save, even after controlling for numerous economic and demographic factors. The implication is that languages which grammatically distinguish the present and the future may bias their speakers to distinguish them psychologically, leading to less future-oriented decision making. However, Chen's original analysis assumed languages are independent. This neglects the fact that languages are related, causing correlations to appear stronger than is warranted (Galton's problem). In this paper, we test the robustness of Chen's correlations to corrections for the geographic and historical relatedness of languages. While the question seems simple, the answer is complex. In general, the statistical correlation between the two variables is weaker when controlling for relatedness. When applying the strictest tests for relatedness, and when data is not aggregated across individuals, the correlation is not significant. However, the correlation did remain reasonably robust under a number of tests. We argue that any claims of synchronic patterns between cultural variables should be tested for spurious correlations, with the kinds of approaches used in this paper. However, experiments or case-studies would be more fruitful avenues for future research on this specific topic, rather than further large-scale cross-cultural correlational studies.

## Introduction

There is a long-standing debate about whether the constraints of the languages we speak influence the way we think and behave [1–8]. For example, words that refer to colours vary between languages, and can influence the way people process colour [9–12]. New large-scale databases allow researchers to discover and test correlations between linguistic features and other types of behaviour. A recent example is the demonstration by Chen that the way a language allows people to talk about future events predicts whether they will choose to save or spend money [13]: speakers of languages which make a grammatical distinction between the present and the future are less likely to save money. The original hypothesis is that the linguistic distinction makes the future seem further away from the present, and biases the individual against preparing for the future.

This example differs from many previous studies in linguistics in two ways. First, it uses a very large survey of hundreds of thousands of people—a larger and more diverse sample than many such studies. Secondly, it links linguistic constraints to long-term, relatively important decisions (economic behaviour). Most previous studies focused on short-term processing biases.

Being able to link economic behaviour and linguistic traits could have a big impact on public policy, as well as theories in linguistics and economics. Therefore it is important to make sure that the correlation is real and not an artefact of big data analyses. It may seem relatively straightforward to demonstrate an association between two variables, but as this paper hopes to demonstrate, there are problems when considering cultural traits. One of the biggest problems in statistics is ensuring that the data meet standards of independence. The strength of an effect can be artificially high if datapoints are not independent [14, 15]. This is particularly a problem with cultural traits because languages and cultures inherit traits from common historical ancestors and borrow traits from neighbouring cultures. In this paper, we argue that the languages in the data used to demonstrate the link between future tense and savings were not independent. We run a series of analyses that attempt to control for this non-independence.

In the original paper, Chen [13] focuses on a linguistic typological variable which categorises whether a language has a strongly grammaticalised future tense (also referred to as ‘future time reference’ or FTR). For example, in English and Spanish a speaker is forced to make changes to the structure of a sentence when talking about the future as opposed to the present (e.g. “It *will be* …” as opposed to “It *is* …”). Finnish and Mandarin, in contrast, can use the present tense when talking about events in the future. This trait correlated with the propensity of speakers to save money rather than spend money in a given year. Chen’s study has found that speakers of a language with a strongly grammaticalised future tense are less likely to save money.

Chen discusses two possible causal mechanisms that could bring about this effect. These are presented as explicit economic models in the original paper. The first is that obligatory linguistic distinctions could bias beliefs. A constant pressure to mark the present tense as different from the future in one’s language can make the temporal future seem further away by contrast. This would lead to a discounting of the potential reward in the future for a cost paid in the present (saving instead of spending) and therefore bias a speaker to spend rather than save. Put another way, if the future seems further away, you are less concerned with preparing for the future.

The second hypothesised mechanism suggests that speakers of strongly-marking future tense languages are less willing to save because they have more precise beliefs about time. A constant pressure to mark the present tense as different from the future could lead to more precise mental partitioning of time. This could lead to more precise beliefs about the exact point in time when the reward for saving would be higher than the reward for spending. The economic model in [13] demonstrates that a more precise belief about the timing of a reward leads to greater risk aversion. This suggests that individuals with more precise beliefs would be more willing to spend money now rather than risk a possibly smaller reward in the future.

The data that demonstrated the correlation came from two main sources. First, a survey of hundreds of thousands of individuals who indicated what language they spoke and whether they saved money in the last year (the World Values Survey, [16]). Secondly, a typological survey of many of the world’s languages which classified languages as either having a strongly or weakly grammaticalised future tense (the EUROTYP database, see [17]). While the socio-economic features of the individuals were well controlled, the original study assumed that languages could be treated as independent data points. This is an unrealistic assumption because the languages we observe in the world now are related by cultural descent (see also e.g. [18, 19]). This makes it difficult to evaluate the strength of a simple correlation between cultural traits, also known as Galton’s problem. That is, two cultures might have the same traits because they inherited them from the same ancestor culture, rather than there being causal dependencies between the traits. Indeed, spurious correlations between unrelated traits are likely to occur in cultural systems where traits diffuse through time and space [20–22].

This paper tests whether Chen’s hypothesis can be rejected on the basis that cultures are not independent. The main test in this paper is a mixed effects model which controls for phylogenetic and geographic relatedness. Mixed effects modelling provides a powerful framework for defining non-independence in large-scale data that does not require aggregation, and allows for specific questions to be addressed. This method has been used to address similar problems in linguistics (e.g. [23, 24]).

Mixed effects modelling is not the only method that can be used to control for non-independence. In order to get a fuller picture of how different methods assess this correlation, we perform additional tests. First, the method employed in the original paper—regression on matched samples—is replicated, but with additional controls for language family. Secondly, in order to evaluate the relative strength of the correlation, we test whether savings behaviour is better predicted by FTR than by many other linguistic features. Thirdly, we test whether the correlation is robust against controlling for geographic relations between cultures using partial Mantel tests and geographic autocorrelation. Finally, we use phylogenetic methods to conduct a more fine-grained analysis of the relationship between FTR and savings behaviour that takes the historical relationships between languages into account. The different methods lead to different conclusions, and we discuss the implications for large-scale statistical research.

We believe that the economic-Whorfian hypothesis is empirically testable and that large-scale cross-cultural statistical studies can be a useful tool in exploring these kinds of hypotheses. However, the nature of these ‘nomothetic’ studies means that they have weak explanatory power, especially when it comes to determining causal effects. Evidence from experimental studies, for example psycholinguistic priming studies, could help demonstrate a causal effect of language on economic decisions.

There are an increasing number of large-scale statistical studies that propose links between cultural traits (e.g. [24–29]), due to increasing amounts of available data and better access to analysis methods. While some of these studies address theoretical problems in linguistics, others touch on issues of concern to the general public and public policy such as economics, politics, gender equality and health [30–35]. For example, grammatical gender typology predicts female participation in the workforce and politics, with the authors concluding that “the direct and possibly cognitive influence of a language on its speakers and on economic life may have important policy implications.” ([30], p.42). However, many of these studies do not control for cultural relatedness. If these studies have implications for public attitudes and public policy, poorly controlled statistical tests could lead to harmful conclusions. One way to test the robustness of a claim about a synchronic pattern is to control for shared history. This paper discusses some methods for doing this.

### Caveats

Chen’s hypothesis has been criticised on a number of grounds, as summarised below. These involve questions about the suitability of the data and the plausibility of the hypothesis. In this paper, we restrict our focus to testing the existence of a correlation between FTR and savings behaviour, and not to evaluate the likelihood of the causal claim. The methods applied here to savings behaviour could be equally applied to the other indices of future-oriented behaviour analysed in [13] (e.g. smoking, obesity, retirement behaviour etc.). For simplicity, we only consider savings behaviour, and note that the results here are not informative for other variables. We hope this paper demonstrates that the complexity of confirming a correlation between just two variables is complicated enough. Rather than testing each variable individually, future statistical work might consider using an overall index of future-oriented behaviour which could be correlated with overall language future tense obligations, or using a structural equation modelling framework to assess multiple indices of future-oriented behaviour. However, we re-iterate that a more informative test of this hypothesis would be a simple experiment. We chose to focus on savings behaviour partly because it is a candidate for manipulation in an experimental study (for example, via an economic game), while the other variables are not.

We assume that the linguistic typology data is accurate and that people’s answers to survey data is unbiased. We also acknowledge that the data does not cover some linguistic areas such as North America. This limits our ability to test whether Chen’s claim applies universally to all cultures. However, this paper does not try to test the universality of Chen’s claim, but instead aims to test whether the correlation that Chen observed was a spurious artefact of sampling related languages. Part of this involves testing whether the relationship between FTR and savings behaviour is the same within all linguistic groups *in the sample*. However, this should not be taken to imply that these tests also address whether the effect applies universally to all cultures.

There are additional questions about how this hypothesis interacts with factors such as the number of speakers (since number of speakers is related to the extent to which meanings are morphologically encoded, [24, 27]), climate, cultural contact or bilingualism. However, these would be parallel or alternative explanations of the link between FTR and savings. This paper seeks to establish whether the correlation between FTR and savings is present in the first place. Furthermore, more extensive statistical analyses may not be the best way to support or disprove the hypothesis. We suggest that theoretical, idiographic (close studies of individual cases) and experimental methods are more suitable for testing hypotheses generated by identifying patterns in big data.

## Background

### Future-time reference

Future-time reference (FTR) is a linguistic typological variable from [17] (not from the World Atlas of Language Structures, as has sometimes been assumed). Part of the typology distinguishes between languages that obligatorily mark statements about the future. Below are some examples from weather reports in English (1), Spanish (2) and Finnish (3) to illustrate the difference.

**Example 1** [36], p. 2.

It will be mostly cool and windy.

**Example 2** [37]

Ya desde la mañana el viento será muy flojito
Gloss: Ready from the morning he wind be+FUT very weak
Translation: *From the morning, the wind will be very weak*

**Example 3** [36], p. 2.

Sää kylmenee, mutta keskiviikkona tuulee idästä ja pyryttää lunta
Gloss: Weather grow-cold+PRES but Wednesday blow+PRES east and drifting snows
Translation: *The weather becomes cooler, but on Wednesday (the wind) blows from the east and there is drifting snow.*

In the English example, the futureness is marked with the auxiliary *will*. The Spanish example also marks the future tense by using a future tense morpheme *-á* on the main verb. The Finnish example, by comparison, does not require any grammatical marking of the future. This indicates that English and Spanish use ‘strongly’ marked future-time reference while Finnish uses only ‘weakly’ marked future-time reference. [36] notes that Europe is an area with many languages that have no obligatory grammatical marking of future-time reference.

### Savings behaviour

The data on economic behaviour comes from the World Values Survey (WVS, [16]), a large scale, cross-cultural questionnaire that was administered to hundreds of thousands of individuals across the world. One of the questions asked about the propensity to save money:

During the past year, did your family (read out and code one answer):

1: Save money

2: Just get by

3: Spent some savings and borrowed money

4: Spent savings and borrowed money

(World Values Survey, Question V251)

In [13] and in this analysis, individuals were coded as saving money if they answered (1) above, and not saving money otherwise. It is important to understand that, from an economics perspective, saving money is seen as a risk, since one trades buying something in the present for going without something in the present and buying later, when conditions may have changed.

The survey also asked each individual “What language do you normally speak at home?” (question V222), with a list of possible languages to choose from that was adapted for each country.

The World Values Survey was administered in ‘waves’ of a few years each. The first wave to ask about savings behaviour was wave 3 (1995–1998), followed by waves 4 (1999–2004), 5 (2005–2009) and 6 (2010–2014). The latest wave was only released after the original analysis by Chen, so we analyse two sub-sets of the database: waves 3–5 (matching the original analysis) and waves 3–6 (including all possible data).

### Effects of language on behaviour

The Sapir-Whorf hypothesis of linguistic relativity suggests that the way language divides the world into concepts can have an effect on the way speakers think about the world [1] (indeed, Whorf attempted to claim that speakers of the language Hopi had no linguistic means of differentiating time and, as a result, had no conceptualisation of a constant passage of time [38]. However, this theory has been thoroughly disproved [39, 40]). While strong linguistic relativity has been difficult to demonstrate, previous work has demonstrated that languages do carve up the world in radically different ways, including conceptions of time [8, 41, 42]. There is also evidence that grammatical features of a language can alter perception and action. For example, seeing language comprehension as essentially requiring a mental simulation of events (e.g. [43–45]), leads to the prediction that language can modulate the way people think about events. Indeed, studies have shown that manipulating tense can affect processing. Information presented in the present tense is recalled faster than information presented in the past tense [46]. Grammatical aspect (which marks how actions are bounded in time, e.g. whether they are happening repetitively or are ongoing) can also direct comprehenders’ mental simulation to particular features of an action [47–50] or affect the perceived duration of an event [51]. Action verbs in the future tense elicit greater hand motor responses than action verbs in the past tense [52]. Even extremely low-level processes such as eye movements are sensitive to the processing of grammatical tense [53].

Chen’s future orientation hypothesis extends the prediction from small differences in on-line behaviour to longer-term, more substantive decisions. Off-line effects on behaviour are evident in [54] which demonstrates that speakers of languages with different aspect typologies differ in their memory of events as well as on-line reporting of events. The aspectual framing of a question also affects the content of people’s reporting of previously observed events [55]. Long-term effects of language-specific metaphors influencing conceptualisations of time have been demonstrated in bilinguals [56]. There is also evidence that language can affect more substantive decisions. For example, the manipulation of perfective or imperfective aspect in a politician’s speech affects the extent to which participants think a politician will be re-elected [57].

It is therefore not unreasonable to think that languages which mark a difference between present and future would direct its speakers’ to attend to different features of a sentence than languages which do not. This is in line with more moderate versions of linguistic relativity such as the idea of ‘thinking for speaking’ [58], or the idea that speakers pay more attention to aspects of the world that are encoded in language [59]. We suggest that psycholinguistic experiments, in the same vein as the studies cited above, may be the most informative test of Chen’s hypothesis.

### Criticism

Chen’s study has been criticised on several grounds. These can be categorised as problems with the data, problems with the inference and problems with the statistics. In the first category, critics have pointed out that linguistic systems for referring to the future are more complex than the binary strong/weak future tense distinction, and there is variation amongst speakers of the same language [60, 61].

It has also been suggested that there is no clear *a priori* prediction of whether the correlation should be positive or negative. Some suggesting that a linguistic distinction could make speakers think more intently about the future [60] (although the economic models described above do not agree). While this does not follow the conventional scientific method (theories generate predictions which are tested with data), large scale statistical analyses can be used exploratively to ‘jump-start’ the conventional process, after which methods with greater explanatory power can be applied [22].

The direction of causality has also been questioned. Since language change is often driven by cultural practices (e.g. [62, 63]), it could be the case that savings behaviour is driving the linguistic typology [64]. However, we raise three objections to this. Firstly, [13] showed that at least some cultural attitudes could not explain the link between savings behaviour and language. The WVS includes data on whether an individual thinks that saving is an important cultural value, as well as whether they actually saved. These two variables were correlated, but the cultural value variable did not effect the correlation between savings behaviour and future-time reference. This suggests that there are different causal effects at work. Secondly, for cultural attitudes to influence language, they would need to be slower-changing than the linguistic changes they produce. If cultural attitudes changed widely in the short-term, then languages could not adapt to them. This is an empirical question for a particular domain, and we demonstrate below that future-time reference variable is very stable over time, given our small sample. Thirdly, the hypothesis that savings behaviour causes changes to future tense appears to make the wrong prediction. If a society condones saving money, then one might predict that it would develop ways of grammatically marking the future from the present in order to facilitate this. Conversely, a community where saving was not an important cultural value would lose the distinction between the present and the future. In fact, [65] shows exactly this kind of relationship. A community of German speakers in Pennsylvania exhibited a social reluctance to make future commitments, which subsequently led to the attenuation of future tense in their dialect. This kind of process does not seem to fit the empirical finding that speakers of weak future tense languages have a propensity to save.

Finally, the statistics have been questioned. Dahl notes that the World Values Survey has clusters of similar cultures which align with the parts of Europe that have weak FTR languages [61]. This would predict that future tense would correlate with many cultural values that are harder to explain given the future orientation hypothesis. It is certainly surprising that FTR is so predictive of many aspects such as smoking and obesity (see [13]), which might suggest that the FTR variable is just an index of deeper cultural tendencies. We also note that other linguistic distinctions have been found to correlate with savings behaviour. For instance, another study finds that women are less likely to save money than men in countries with languages that make distinctions in grammatical gender [30]. More generally, Lieberman [21] demonstrates using a computational simulation that cultural variables that diffuse geographically are likely to become correlated, even if they are not causally related. The analyses below address these issues by testing whether FTR and savings behaviour are still correlated when controlling for cultural descent and geographical proximity.

### Testing nomothetic hypotheses

**Fig 1:**
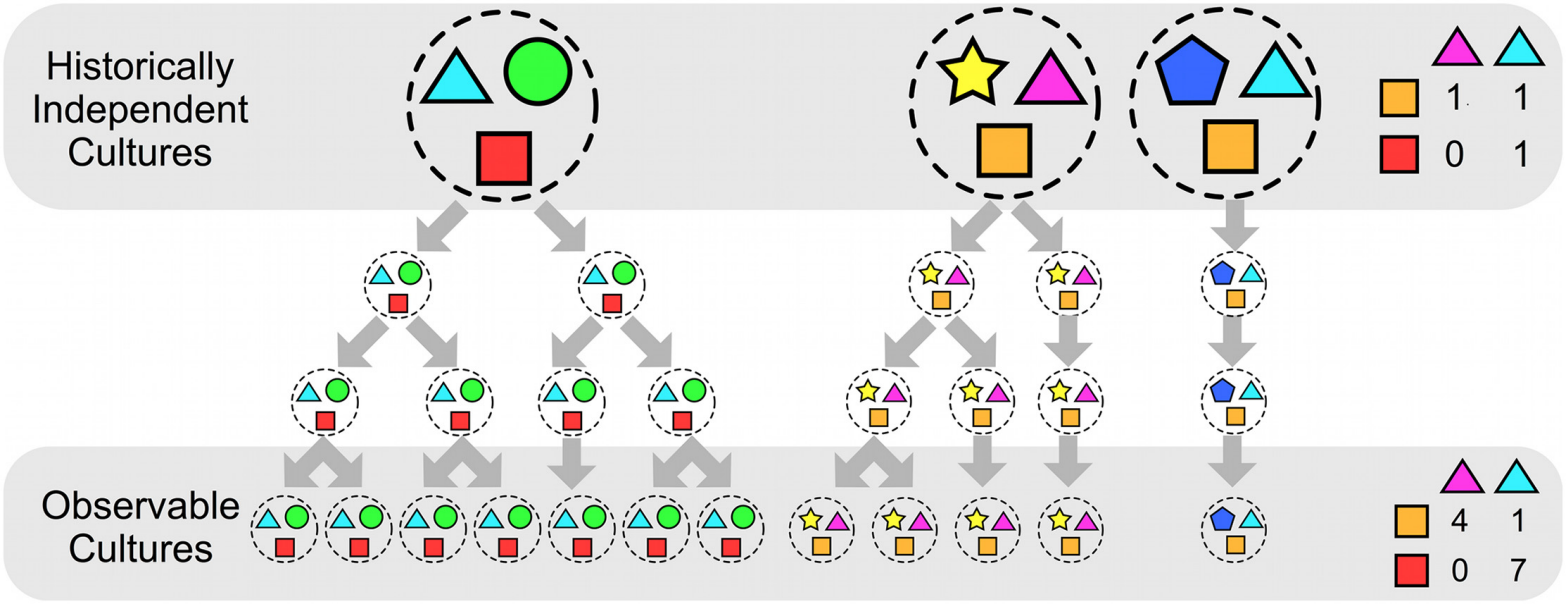
Spurious correlations can be caused by cultural inheritance. An illustration of how cultural inheritance can lead to spurious correlations. At the top are three independent historical cultures, each of which has a bundle of various traits which are represented as coloured shapes. Each trait is causally independent of the others. On the right is a contingency table for the colours of triangles and squares. There is no particular relationship between the colour of triangles and the colour of squares. However, over time these cultures split into new cultures. Along the bottom of the graph are the currently observable cultures. We now see a pattern has emerged in the raw numbers (pink triangles occur with orange squares, and blue triangles occur with red squares). The mechanism that brought about this pattern is simply that the traits are inherited together: there is no causal mechanism whereby pink triangles are more likely to cause orange squares.

**Fig 2:**
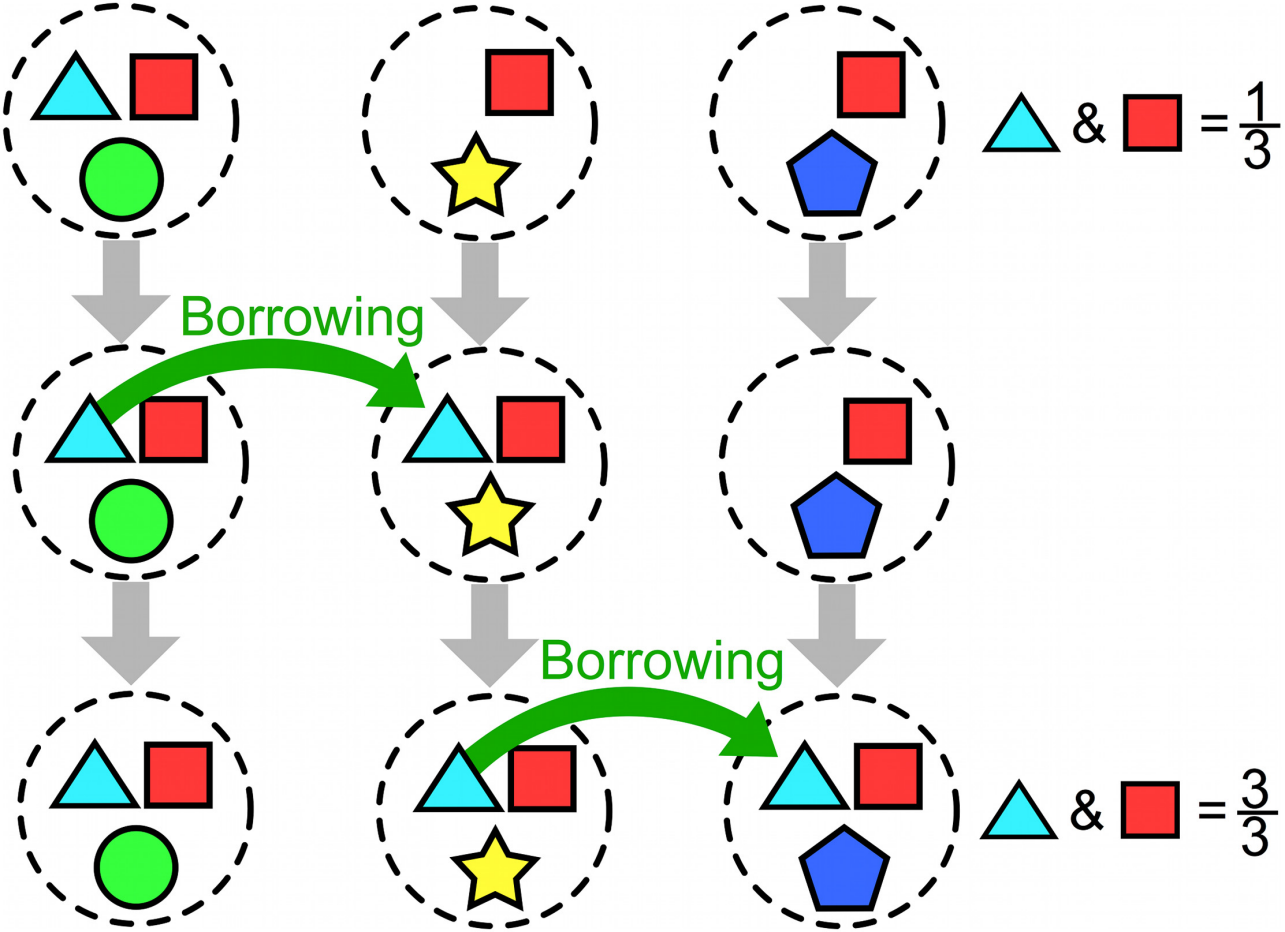
Spurious correlations can be caused by borrowing. An illustration of how borrowing (horizontal cultural inheritance) can lead to spurious correlations. Three cultures (left to right) evolve over time (top to bottom). Each culture has a bundle of various traits which are represented as coloured shapes. Each trait is causally independent of the others. On the right is a count of the number of cultures with both blue triangles and red squares. In the top generation, only one out of three cultures have both. Over some period of time, the blue triangle is borrowed from the culture on the left to the culture in the middle, and then from the culture in the middle to the culture on the right. By the end, all languages have blue triangles and red squares. The mechanism that brought about this pattern is simply that one trait spread through the population: there is no causal mechanism whereby blue triangles are more likely to cause red squares. A similar effect would be caused by a bundle of causally unrelated features being borrowed.

Evaluating claims from large-scale, cross-linguistic databases—a ‘nomothetic’ approach—is a complex task (see [22, 66–69]). Cultures have bundles of traits—both linguistic and behavioural. Demographic processes cause these traits to be inherited as cultures migrate and split, or to be borrowed together as cultures merge. The co-occurrence of particular traits can look very different when considering historically independent ancestor cultures than currently observable ones. Fig 1 illustrates this problem. It shows three independent ancestor cultures, with various traits shown as coloured shapes. There is no particular relationship between the colour of triangles and the colour of squares. However, over time these cultures split into new cultures. If we consider each of the currently observable cultures, we now see a pattern has emerged in the raw numbers (pink triangles occur with orange squares, and blue triangles occur with red squares). The mechanism that brought about this pattern is simply that the traits are inherited together: there is no causal mechanism whereby pink triangles are more likely to cause orange squares. A similar effect is seen when cultural traits are borrowed from neighbouring cultures (Fig 2).

Below, we run a series of analyses that test the robustness of the correlation between FTR and savings behaviour when taking into account inheritance relationships between languages. Since there is little prior theory to support a link between FTR and savings, there is little to motivate predictions. As noted above, some critics have suggested that the opposite correlation might be expected. However, if the correlation is robust, and in the direction predicted by Chen, there are several possible explanations. The first possibility is that Chen’s hypothesis is correct. While the approach in the current paper may not be the best evidence to support Chen’s claim, it may demonstrate that this hypothesis is worth exploring further. However, there are several other possibilities, as discussed below.

#### Savings behaviour affects FTR

As noted above, it may be that socio-economic behaviour shaped the grammatical structure of languages. One could imagine that, if representing future actions explicitly in the language is important, a language might develop alternative ways of expressing promises. Interestingly, a different study found that the stock returns of a firm are linked to the extent to which firms talk about the future in their annual reports [70]. The argument against this line of reasoning is that linguistic traits are expected to change much more slowly than economic traits. Below we estimate that FTR is indeed a stable feature, but that economic behaviour also has a relatively strong phylogenetic signal.

#### FTR and savings behaviour co-diffuse

Cultural features are likely to become correlated because they are historically related or spread geographically [18, 22, 23]. The correlation observed by Chen may be an artefact of these processes. The rest of the paper focuses on assessing this claim. However, there are two other (unlikely) possibilities. It may be that a third variable makes future tense typology and economic behaviour more likely to be inherited or borrowed together. We know of no theory that would predict an inherent link between the inheritance of these traits, but there may be a more general factor. For example, colonisation may involve imposing both linguistic and economic norms, but not others such as diet. Alternatively, some combinations of FTR and savings behaviour could cause a culture to be more likely to proliferate or split, leading to a scenario similar to Galton’s problem. Again, we know of no theories that would predict this.

#### The correlation is an artefact of big data analyses

While increasing the size of a dataset usually increases the statistical power of a test, the noise-to-signal ratio also increases exponentially, meaning that real effects are harder to extract from spurious correlations [71]. Large datasets can be useful if they consist of consistent measurements of well-defined, relatively simple physical properties. However, the cultural datasets are often characterised by underlying complexity and inconsistent criteria. Since the World Values Survey has a large number of respondents and is run slightly differently in a number of countries, it may be the case that the link between future tense marking and economic decisions is just an artefact of noise. In order to address this issue to some degree, one of the analyses below tests whether the link between future tense and economic decisions is stronger than many comparable links in the same dataset.

#### FTR and savings are related diachronically

It is possible for the pattern of FTR and savings behaviour to be affected by borrowing and inheritance but also for a causal link to be present. For example, if there is a causal link, and culture A borrows strong FTR from culture B, then culture A may also change their savings behaviour as a direct consequence of now having strong FTR. That is, a diachronic change in savings behaviour after a change in FTR is positive evidence for the existence of a causal link between the two variables (see [72–74], also applied in e.g. [20], see [75, 76] for discussion). In this case, the causal effect may lead to a synchronic pattern.

While correlated diachronic change may provide evidence for a causal link, the resulting synchronic data should not be treated as being composed of independent samples. We claim that this is the problem with the original study. This paper tests whether the size of the effect in the original study is inflated by synchronic non-independence. If, as we argue, this is the case, it does not rule out that there could be diachronic evidence, but it does mean that synchronic patterns may not be valid evidence for causal effects.

Chen’s claim could be addressed with phylogenetic, diachronic methods. Indeed, the methods are a more powerful tool for providing evidence for a causal mechanism at the same time as taking historical relationships into account. One problem in this case is that the original claim was based on data from many different language families. There are two issues for phylogenetic processes here. First, not all language families in the sample currently have enough linguistic data available (e.g. cognate codings) in order to perform phylogenetic methods. Secondly, it is currently unclear how language families are related diachronically, making studies of effects across language families difficult (indeed, these methods are usually explicitly restricted to studies of languages within the same language family). Within-family analyses are possible, but in the current data there are few language families with enough coverage and variation for an ideal analysis. Furthermore, correlated change is not always evidence for a causal connection. As [77] argue, only parallel change in several clades (replicated bursts) provide good evidence for a causal connection (correlated change in a single clade may be attributable to a single ancestral change). Further work would have to be done to establish whether this was the case.

There is a further complication in the case of this paper. While most phylogenetic studies in linguistics focus only on features of populations (i.e. linguistic features), the economic data in this study relate to individual people. That is, the correlation relates micro-evolution of individual spending behaviour with macro-evolution of languages. It is not clear how to model both cultures and individuals in a phylogenetic framework. Simple aggregation of data within a language is one solution. However, in this case individuals are also affected in their decision to save money by personal factors such as employment status. In the analyses below we suggest that regression techniques can be used to aggregate data within languages. For example, assigning the amount of variance in a variable for a given language that is not accounted for by personal factors (e.g. residualisation). Another approach would be more explicit agent-based modelling [78].

### Null Hypothesis

The null hypothesis is that the correlation between future tense marking and propensity to save money is a spurious artefact of the historical and geographic relatedness of languages. The alternative hypothesis is that there is a tendency for speakers of languages with strong future tense marking to be less likely to save money. The alternative hypothesis does not require an absolute universal with no exceptions, but implies a statistical tendency (see [79]).

## Main analysis: Model Comparison using mixed effects models

The main statistical method we use for testing the correlation between FTR and savings behaviour is mixed effects modelling. Linear mixed-effects models are extensions of the standard linear regression family of models. Mixed effect models have two components: fixed effects and random effects. Fixed effects are much the same as ordinary regression predictor variables. Random effects are used to model sources that could introduce random errors into the data. For example, an analysis of a reaction time experiment could have a random intercept effect for participants, which would control for some participants having higher reaction times across the board. It could also have a random slope effect, which would allow the slope of the regression to be different for each individual. This might provide a better fit to the data if some participants are more sensitive to the experimental manipulation than others.

We would like to control for three sources of non-independence: similarities between people’s economic conditions caused by them being in the same state, similarities in their language and culture caused by historical relations and similarities in their language and culture caused by cultural contact. In our context, we include random effects for country, language family and (linguistic) geographic area. If the FTR and savings behaviour correlation is being driven by particular countries, language families or areas having similar features, then this variance will be absorbed by the random effects, rather than the fixed effect of FTR. This method has been used previously to control for language relatedness in large-scale cross-linguistic comparison [23, 24]. We use a logistic mixed effects model in R [80], using the *lme4* package [81] (version 1.17).

Using propensity to save as our binary dependent variable we performed several separate linear mixed effect analyses based on the fixed effects of (a) FTR, (b) Trust, (c) Unemployment, (d) Marriage, and (e) Sex. As random effects, we included random intercepts for language family, country and geographic area, with each of these intercepts having random slopes for the fixed effect (no models included interactions). The language family was assigned according to the definitions in WALS, and provides a control for vertical cultural transmission. The geographic areas were assigned as the Autotyp linguistic areas that each language belonged to [82] (not the geographic area in which the respondents lived, which is effectively handled by the random effect by country). These areas are designed to reflect areas where linguistic contact is known to have occurred, providing a good control for horizontal cultural transmission.

There are two main ways of extracting significance from mixed effects models. The first is to compare the fit of a model with a given fixed effect (the main model) to a model without that fixed effect (the null model). Each model will fit the data to some extent, as measured by likelihood (the probability of observing the data given the model), and the main model should allow a better fit to the data. The extent of the improvement of the main model over the null model can be quantified by comparing the difference in likelihoods using the likelihood ratio test. The probability distribution of the likelihood ratio statistic can be approximated by a chi-squared distribution (with degrees of freedom equal to the difference in degrees of freedom between the null model and main model, [83]). This yields a p-value which indicates whether the main model is preferred over the null model. That is, a low p-value suggests that the given fixed effect significantly improves the fit of the model, and is therefore correlated with the dependent variable.

The second method of calculating significance for a given fixed effect is the Wald-z statistic. In the current case, the proportion of people saving money is estimated for weak-FTR speakers and for strong-FTR speakers (given the variance accounted for by the additional random effects). The difference between these estimates is taken as the increase in the probability of saving due to speaking a weak-FTR language. Given a measure of variance of the fixed effect (the standard error), the Wald statistic is calculated, which can be compared to a chi-squared distribution in order to produce a p-value. A p-value below a given criterion (e.g. p < 0.05) indicates that there is a significant increase in the probability of saving due to speaking a weak FTR language compared to a strong FTR language.

While the two methods of deriving probability values will provide the same results given a sample size that approaches the limit [84], there can be differences in limited samples. The consensus in the mixed effects modelling literature is to prefer the likelihood ratio test over the Wald-z test [85–88]. The likelihood ratio test makes fewer assumptions and is more conservative. In our particular case, there were also problems estimating the standard error, making the Wald-z statistic unreliable (this was a problem with the mixed effects modelling software *lme4*, which is described in S3 Appendix).

We used two versions of the WVS dataset in order to test the robustness of the method: the first includes data up to 2009, so-called waves 3 to 5 (the first wave to ask about savings behaviour was wave 3). This dataset is the source for the original analysis and for the other statistical analyses in the current paper. The second dataset includes extra data from wave 6 that was recorded from 2010 to 2014 and released after the publication of [13] and after the initial submission of this paper.

## Results

In this paper we test the robustness of the correlation between strongly marked future tense and the propensity to save money [13]. The null hypothesis is that there is no reliable association between FTR and savings behaviour, and that previous findings in support of this were an artefact of of the geographic or historical relatedness of languages.

**Fig 3:**
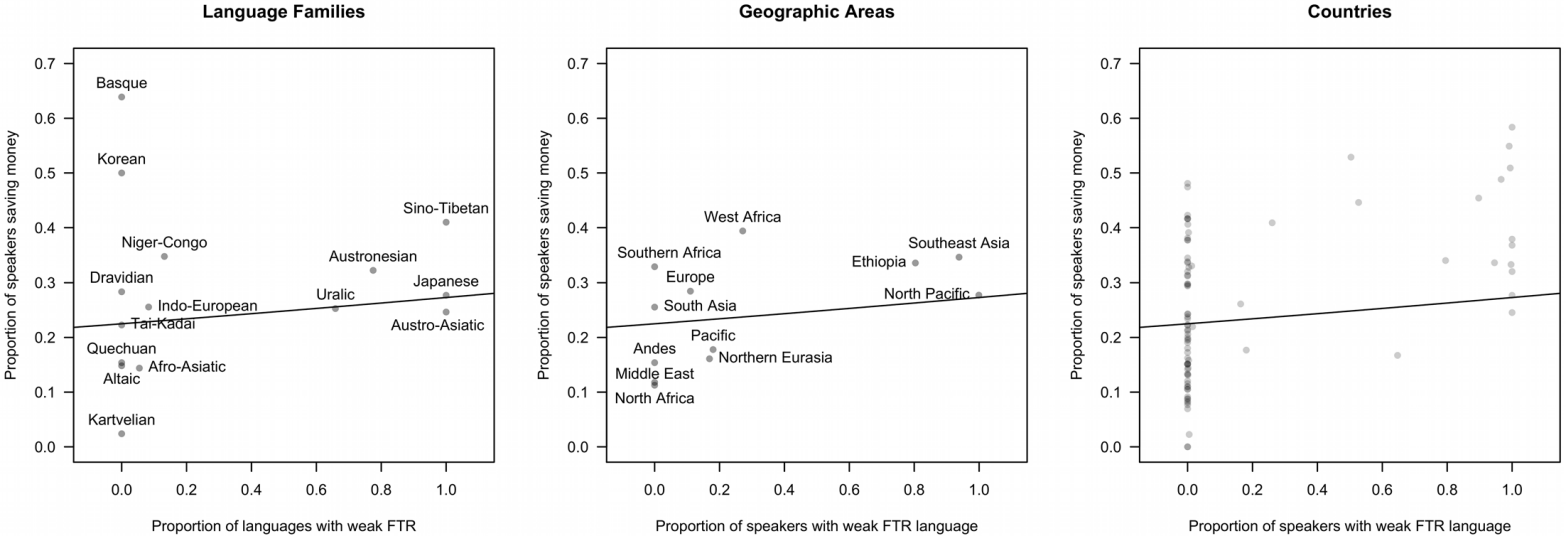
Aggregation of data by language family, area and country. Proportion of speakers saving money as a function of the proportion of languages with a weak FTR language, aggregated over language family (left), geographic area (middle) and country (right). The line in each graph represents the mixed effects model regression (waves 3–6).

As a simple way of visualising the data, Fig 3, shows the data aggregated over countries, language families and linguistic areas (S10 Appendix shows summary information for each language within each country). The overall trend is still evident, though it appears weaker. This is slightly misleading since different countries and language families do not have the same distribution of socioeconomic statuses, which effect savings behaviour. The analyses below control for these effects.

In this section we report the results from the main mixed effects model. Table 1 shows the results of the model comparison for waves 3 to 5 of the WVS dataset. The model estimates that speakers of weak FTR languages are 1.5 times more likely to save money than speakers of weak FTR languages (estimate in logit scale = 0.41, 95%CI from likelihood surface = [0.08, 0.75]). According to the Wald-z test, this is a significant difference (z = 24, p = 0.02, though see note above on unreliability of Wald-z p-values in our particular case). However, the likelihood ratio test (comparing the model with FTR as a fixed effect to its null model) finds only a marginal difference between the two models in terms of their fit to the data (*χ* ^2^ = 2.72, p = 0.1). That is, while there is a correlation between FTR and savings behaviour, FTR does not significantly increase the amount of explained variation in savings behaviour (S1 Appendix includes additional analyses which show that the results are not qualitatively different when including a random effect for year of survey or individual language).

**Table 1:** Results of the model comparison using mixed effects modelling using waves 3 to 5.

	Wald-z				Likelihood ratio test	
Model (fixed effect)	Estimate	Std. Error	Z value	Pr (>z)	*χ* ^2^	Pr (>*χ* ^2^)
Model A (Weak FTR)	0.41	0.17	2.40	0.01646	2.72	0.0992
Model B (No Trust)	-0.13	0.06	-2.20	0.02760	3.59	0.0583
Model C (Employment)	0.60	0.10	6.10	< 0.00001	17.41	< 0.0001
Model D (Sex female)	-0.11	0.05	-2.36	0.01851	4.10	0.0429

Results for fixed effects for various models (columns 2–5), and the comparison between the respective null model and the model with the given fixed effect. Data comes from waves 3 to 5 of the World Values Survey. Estimates are on a logit scale.

The effect of FTR weakens when we add data from wave 6 of the WVS (model E, see Table 2): the estimate of the effect weak FTR on savings behaviour drops from 1.5 times more likely to 1.3 times more likely (estimate in logit scale = 0.26, 95% CI from likelihood surface = [-0.06, 0.57]). FTR is no longer a significant predictor of savings behaviour according to either the Wald-z test (z = 1.58, p = 0.11) or the likelihood ratio test (*χ* ^2^ = 1.15, p = 0.28).

**Table 2:** Results of the model comparison using mixed effects modelling using waves 3 to 6.

	Wald-z				Likelihood ratio test	
Model (fixed effect)	Estimate	Std. Error	Z value	Pr (>z)	*χ* ^2^	Pr (>*χ* ^2^)
Model E (Weak FTR)	0.26	0.16	1.58	0.11502	1.15	0.2830
Model F (No Trust)	-0.16	0.06	-2.65	0.00796	5.30	0.0213
Model G (Employment)	0.61	0.09	6.60	< 0.00001	18.66	< 0.0001
Model H (Sex female)	-0.12	0.03	-3.58	0.00035	6.54	0.0106

Results for fixed effects for various models (columns 2–5), and the comparison between the the respective null model and the model with the given fixed effect. Data comes from waves 3 to 6 of the World Values Survey. Estimates are on a logit scale.

In contrast, employment status, trust and sex (models F, G and H) are significant predictors of savings behaviour according to both the Wald-z test and the likelihood ratio test (employed respondents, respondents who are male or trust others are more likely to save). Furthermore, the effect for employment, sex and trust are stronger when including data from wave 6 in comparison with just waves 3–5.

It’s possible that the results are affected by immigrants, who may already be more likely to take economic risks (in one sense, many immigrants are paying a short-term cost in the hope of a long-term gain). However, only 5% of the data come from individuals with either an immigrant father or mother. Also, the effects were slightly weaker when excluding immigrants (see S1 Appendix). There were also no qualitative differences when using continent instead of Autotyp linguistic area to control for geographic relatedness, nor when using language genus instead of language family to control for genealogical relatedness (see S1 Appendix).

We can explore how the effect of FTR differs across countries, language families and geographic areas by looking at the estimates for the random effects (due to convergence problems, the random slope and intercept estimates come from Bayesian mixed effects models [89]. There are no qualitative differences between the two types of mixed effects model for any result, see S2 Appendix). If people had the same propensity to save across the board according to country, family or area, then the random intercepts should not vary greatly. This is not crucial for the hypothesis, and we expect the random intercept to reflect differences in propensity to save, especially by country. If the effect of FTR on savings behaviour was consistently strong and in the same direction across countries, families or areas, then the random slopes for FTR would not vary greatly. If the slopes do vary, it does not necessarily mean that there is no effect of FTR on savings, only that the strength of the effect varies for different sub-sets of the data.

**Fig 4:**
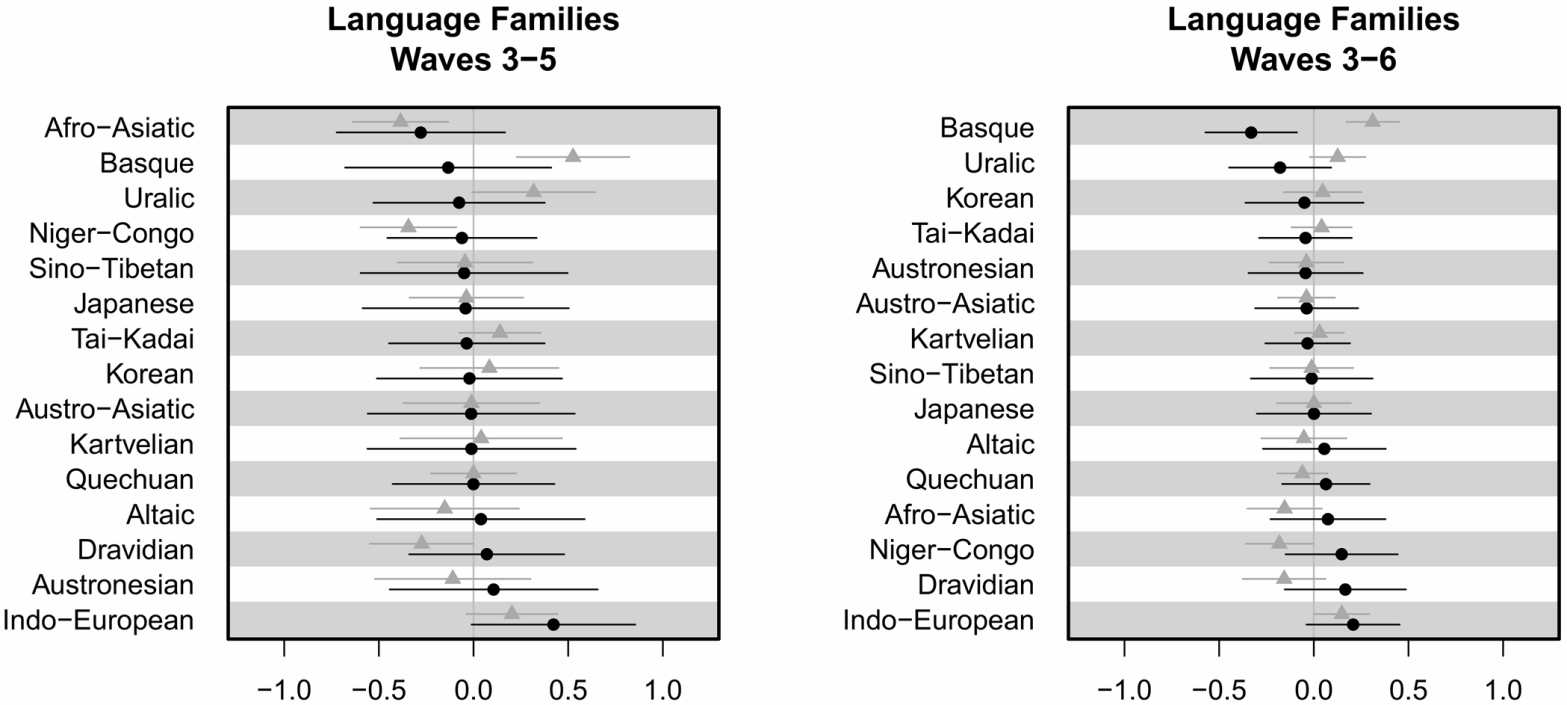
Random intercepts and slopes by language family. For each language family, the graph shows the random slope for FTR (black dots) and random intercept (grey triangles), with a bar showing 1 standard error. The results are shown for models run on waves 3–5 (left) and 3–6 (right). Language families are sorted by random slope.

For example, Fig 4 shows the random intercepts and FTR slope for language families. Higher intercepts indicate higher overall propensity to save. The random slopes for FTR by family show by how much the FTR effect estimate should be adjusted for each family (on a logit scale). The random slopes vary, indicating that speakers from different language families have a different overall propensity to save. The FTR random slopes do not vary to a great extent, but in the results for both waves 3–5 and waves 3–6, the Indo-European language family is an outlier. This suggests that the effect of FTR on savings might be stronger for speakers of Indo-European languages. This could be what is driving the overall correlation.

**Fig 5:**
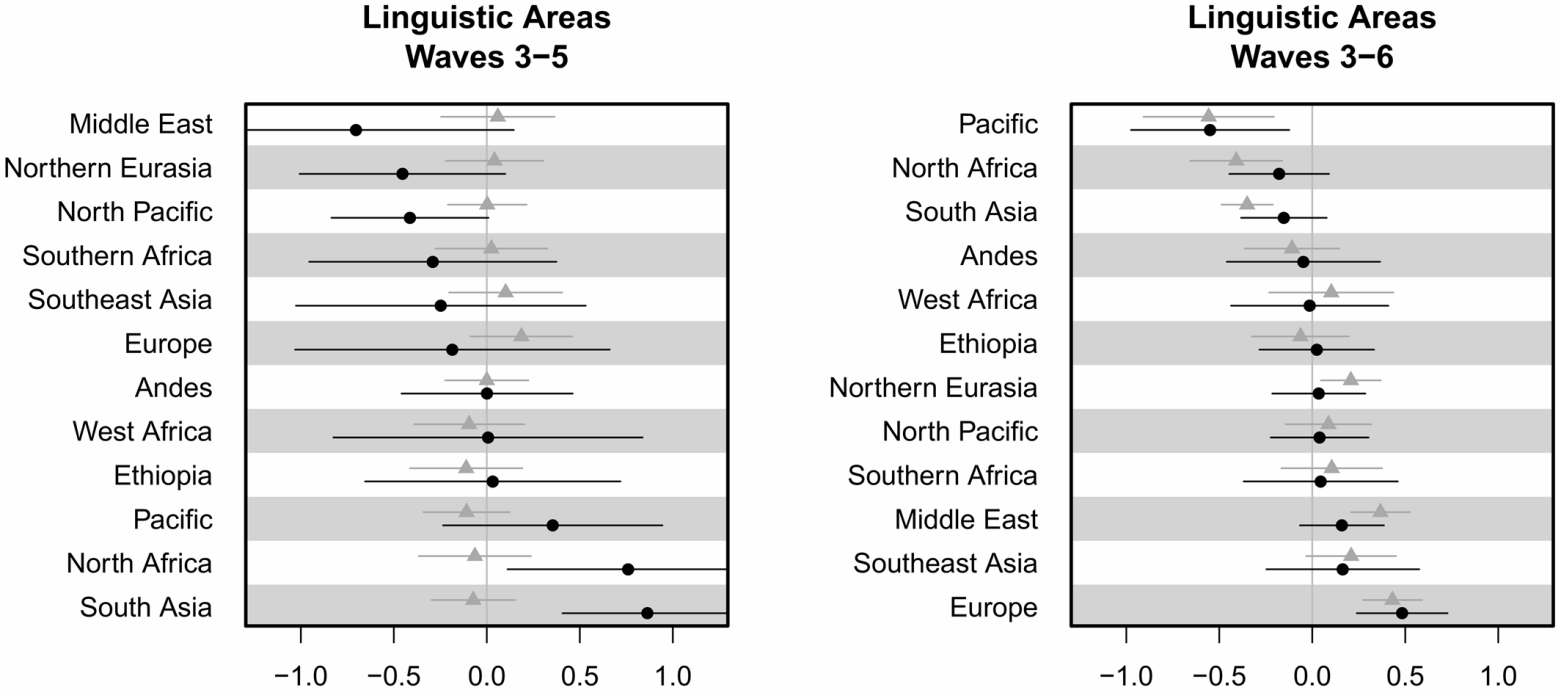
Random intercepts and slopes by geographic area. For each area, the graph shows the random slope for FTR (black dots) and random intercept (grey triangles), with a bar showing 1 standard error. The results are shown for models run on waves 3–5 (left) and 3–6 (right). Areas are sorted by random slope.

Fig 5 shows the random intercepts and FTR slope for each linguistic area. For waves 3–5, the intercepts do not vary considerably by area, suggesting that the overall propensity to save does not vary by area (in comparison with country and family). However, the FTR random slope does vary, with the effect of FTR on saving being stronger in South Asia and weaker in the Middle East. The picture changes when looking at the data from waves 3–6. Now, the random slopes vary to a greater extent, and the FTR slope is quite different in some cases. For example, the effect of FTR is stronger in Europe and weakest in the Pacific. Again, this points to Europe as the source of the overall correlation.

The random intercept for a given country (see S2 Appendix for full details) is correlated with that country’s per-capita GDP (waves 3–5: r = 0.24, t = 2.1, p = 0.04; waves 3–6: r = 0.23, t = 2.1, p = 0.04), which means that respondents from wealthier countries are more likely to save money in general. The random slopes by country are negatively correlated with the random intercept by country (for waves 3–6, r = -0.97), which balances out the influence of the intercept. So, for example, take the proportion of people saving money in Saudi Arabia. The estimated difference between people who speak strong and weak FTR languages, taking into account both the overall intercept, the fixed effect, the random intercept and the random slope, is actually very small (less than 1% difference in proportions). The largest difference happens to be for Australia, where it is estimated that 33% of strong-FTR speakers save and 49% of weak-FTR speakers save.

One possible explanation for the results is that the model comparison is overly conservative in the case of FTR, and we are failing to detect a real effect (type II error). There are two reasons why this might not be the case. First, it should be noted that the predicted model for FTR only included FTR as a fixed effect, and did not include any of the other fixed effects that are predictors of savings behaviour (e.g., unemployment, see S1 Appendix). As such, this model is anti-conservative with respect to the significance of FTR (indeed, FTR has a weaker significance again when including other variables in the same model, see section 5 of S1 Appendix). Second, with the inclusion of wave 6 of the WVS, the significance decreases. This suggests that the original correlation for FTR is partially an artefact of the structural properties of the dataset (see also the section below on the small number bias).

This is further supported by the following finding: when running the same model, but without random slopes for FTR by country, language family and linguistic area, the FTR fixed effect is significant according to the Wald-z test (data from waves 3–6, logit estimate for FTR = 0.20, std. error = 0.05, z = 3.83, p = 0.0001) and according to the likelihood ratio test (*χ* ^2^ = 14.32, p = 0.0002, see section 6.1 of S1 Appendix for details). That is, if we assume that FTR has the same effect across all language families and areas, the correlation is strong, but if we allow the effect of FTR to vary then the effect of FTR is weakened. In other words, controlling for differences in historical inheritance and contact reduces the strength of the correlation between FTR and savings behaviour.

Therefore, part of the answer to whether FTR is related to savings behaviour depends on whether or not one should control for differing strengths of the effect over the world. Theoretically, if one assumes that the cognitive effects are universal, one might expect the effect of FTR to be consistent across countries, areas and linguistic families. However, model comparison demonstrates that random slopes by country and area are warranted by the data (they significantly improve the fit of the model), and when including these random slopes, the relationship between FTR and savings behaviour is not significant (data from waves 3–6, logit estimate for FTR = 0.28, std. error = 0.15, z = 1.84, p = 0.066; likelihood ratio test *χ* ^2^ = 1.58, p = 0.21, for full details, see section 6 of S1 Appendix).

### Differences in wave 6

The strength of the correlation between FTR and savings behaviour is weaker when including data from wave 6. We attribute this to the general improvement in coverage and diversity of respondents. The proportion of people saving remains roughly the same (24.5% before wave 6, 23.0% including wave 6). The same is true for proportion of speakers of FTR languages (83.9% before wave 6, 86.3% including wave 6).

Before wave 6, there were an average of 3.9 languages per country. This increases to 4.16 when including wave 6, although this is not as big an increase as the increase from wave 4 to wave 5. There was no variation in FTR value for many countries (54 out of 75), linguistic areas (5 out of 12) and language families (10 out of 15), although the proportion of countries without variation decreases in wave 6 (59 out of 85). FTR is not a significant predictor of savings behaviour when considering only the countries with variation in FTR (FTR logit estimate = -0.19, std. err. = 0.16, z = -1.13, p = 0.25). For the same data, FTR is significant when running the model without random slopes, although the effect size is much reduced compared to the model with full data (FTR logit estimate = -0.17, std. err. = 0.05, z = -3.11, p = 0.002).

Wave 6 includes data from 10 countries previously not attested. One of these is the Netherlands, which is one of the languages identified as an outlier in the methods section below. Looking at the data, we find that, prior to wave 6, none of the Dutch speakers lived in the Netherlands. In wave 6, 1747 Dutch speakers were included, all of whom lived in the Netherlands.

The random effects are similar for waves 3–5 and waves 3–6 by country and family, but not by area. This suggests that the major differences in the two datasets has to do with wider or denser sampling of geographic areas.

The largest proportional increases of cases are for Dutch, Uzbek, Korean, Hausa and Maori, all at least doubling in size. Three of these have strongly marking FTR. In each case, the proportion of people saving reduces to be closer to an even split. Wave 6 also includes two previously unattested languages: Shona and Cebuano.

### Small Number Bias

The estimated FTR coefficient is stronger with smaller sub-samples of the data (FTR coefficient for wave 3 = 0.57; waves 3–4 = 0.72; waves 3–5 = 0.41; waves 3–6 = 0.26; see S1 Appendix). This could be indicative of a small number bias [90], where smaller datasets tend to have more extreme aggregated values. As the data is added over the years, a fuller sample is achieved and the statistical effect weakens. The weakest statistical result is evident when the FTR coefficient estimate is as precise as possible (when all the data is used).

In comparison, the coefficient for employment status is *weaker* with smaller sub-samples of the data (employment coefficient for wave 3 = 0.41, waves 3–4 = 0.54, waves 3–5 = 0.60, waves 3–6 = 0.61). That is, employment status does not appear to exhibit a small number bias and as the sample size increases we can be increasingly confident that employment status has an effect on savings behaviour.

### Heteroskedasticity

From Fig 3, it is clear that the data exhibits heteroskedasticity—there is more variance in savings for strong-FTR languages than for weak-FTR languages (in the whole data the variance in saving behaviour is 1.4 times greater for strong-FTR languages). There could be two explanations for this. First, the weak-FTR languages could be under-sampled. Indeed, there are 5 times as many strong-FTR respondents than weak-FTR respondents and 3 times as many strong-FTR languages as weak-FTR languages. This could mean that the variance for weak-FTR languages is being underestimated. In line with this, the difference in the variance for the two types of FTR decreases as data is added over waves. If this is the case, it could increase the type I error rate (incorrectly rejecting the null hypothesis). The test using random independent samples (see methods section below) may be one way of avoiding this problem, although this also relies on aggregating the data.

However, perhaps heteroskedasticity is part of the phenomenon. As we discuss below, it is possible that the Whorfian effect only applies in a particular case. For example, perhaps only speakers of strong-FTR languages, or languages with strong-FTR plus some other linguistic feature are susceptible to the effect (a unidirectional implication). It may be possible to use Monte-Carlo sampling methods to test this, (similar to the independent samples test, but estimating quantiles, see [91]), although it is not clear exactly how to select random samples from the current individual-level data. Since the original hypothesis does not make this kind of claim, we do not pursue this issue here.

### Overview of results from alternative methods

In addition to the mixed-effects modelling above, we carried out a range of other methods that are also able to control for non-independence of data (see Table 3). The details of these analyses can be found in the materials and methods section. These tests were carried out on the original data (waves 3–5).

**Table 3:** Summary of statistical methods used in this paper.

Test	Summary of test	Is data aggregated?	Control for language family	Control for geographic area	Control for country	Is the correlation robust?
Mixed effects model	Is the correlation robust when controlling for the random influence of language family, geographic area or country?	No	Yes	Yes	Yes	No
Regression on matched samples	Does FTR predict savings when comparing individuals that are matched on many levels, including language family?	No	Yes	No	Yes	Yes
Serendipity test	Is savings behaviour more strongly associated with FTR than other linguistic variables?	No	Yes	No	Yes	Yes
Independent samples	Do speakers of strong FTR languages have a lower average propensity to save in historically independent languages?	Yes	Yes	No	No	Yes
Partial Mantel test	Is the difference in saving behaviour between two linguistic groups predicted by the difference in FTR, over and above the differences in phylogeny and geography?	Yes	Yes	Yes	No	Yes
Partial Stratified Mantel test	As above, but only comparing samples within language families.	Yes	Yes	Yes	No	No
Geographic autocorrelation	Does the relationship between FTR and savings exhibit geographical clustering?	Yes	No	Yes	No	Yes
Phylogenetic Generalised Least Squares	Does FTR predict saving behaviour when controlling for phylogeny?	Yes	Yes	No	No	Yes
PGLS within families	As above, but separately for each language family.	Yes	Yes	No	No	No

A summary of the statistical methods used to assess whether the relationship between obligatory future tense (FTR) and the propensity to save money is robust to controlling for shared cultural history. Some methods aggregate the data over languages (column 3). Columns 4, 5 and 6 state whether the method implements a control for language family, geographic area and country, respectively. The mixed effects model is the only method that does not aggregate the data and which provides an explicit control for language family, geographic area and country. The final column suggests whether the overall result for the given method demonstrates that the relationship between FTR and savings behaviour is robust. However, this does indicate the status of tests for a given method (see text for details).

We replicated part of the original study using a regression on matched samples framework, but with additional controls for language family. Regression on matched samples essentially splits the data into bins where, within each bin, datapoints are matched for a set of variables (slightly confusingly called ‘fixed effects’, although the concept is different from ‘fixed effects’ in a mixed effects framework). The test then compares the distribution of the independent variable over a specific dependent variable within each matched sample. In our case, each bin includes individual survey respondents who came from the same country, spoke a language from the same language family, were surveyed in the same survey year and had the same economic status, level of education and so on (see the materials and methods section for details). The dependent variable was economic savings behaviour and the test compared the distribution of this variable over FTR language types.

The language family of a speaker’s language was a significant predictor of savings behaviour, but the strength of FTR in a speaker’s language also remained significant. While the replication suggests that the effects are robust, it does not indicate whether FTR is *special* in its relationship with savings behaviour. It is possible that a range of linguistic variables are correlated with savings behaviour, since cultural traits are inherited in bundles. Therefore, we ran a ‘serendipity’ test. 192 other regressions on matched samples were run, each one using a different linguistic dependent variable instead of FTR. We found only 2 other variables out of 192 that predicted savings behaviour better than the FTR variable. This suggests that there is a low probability of finding a correlation with the same strength as FTR and savings by chance.

The other methods for controlling for phylogenetic or geographic relatedness employed in this paper usually require aggregation of data over languages. The original data consisted of survey results from individual people, so the proportion of speakers of a particular language saving money had to be aggregated. However, the regressions on matched samples showed that savings behaviour of an individual is also predicted by their particular socioeconomic status and their cultural attitudes. Therefore, using a simple aggregation of people saving within a given language is misleading. Instead, we used the residuals from the regression on matched samples. That is, the regression predicts some amount of the variance in savings behaviour based on income, education, sex and so on. The residuals represent the amount of variation in the savings behaviour that is not explained by these factors. These can be aggregated by language, providing a variable that represents the savings behaviour of its speakers while taking into account non-linguistic factors. We can then test the correlation between this residualised variable and the language’s FTR typology.

One way of ensuring independence of data points is to run a test on a sub-sample of the data where the datapoints are known to be independent at some level. Samples were taken for strong and weak FTR languages so that each language within a sample came from and independent language family. The strong-FTR sample had a lower propensity to save (as measured by the residualised variable) than the weak-FTR sample in 99% of cases.

We controlled for geographic relatedness using Mantel tests involving physical distance and geographic distance. The difference between two languages in the FTR variable or savings behaviour is correlated with the phylogenetic distance between them. That is, languages which are more closely related are more similar than distantly related languages. This suggests that controlling for relatedness is warranted. However, the difference between two languages in the FTR variable or savings behaviour was *not* correlated with geographic distance between them. The correlation between FTR and savings behaviour remained significant when controlling for both physical distance and phylogenetic distance (r = 0.14, p = 0.001, 95% CI[0.08, 0.19]).

We also used a phylogenetic framework to control for the historical relatedness between languages. Both the savings variable and the FTR variable had strong phylogenetic signals (changes in each were estimated to be historically dependent and not due to random drift). This suggests that both variables are not affected to a large extent by horizontal transmission. The FTR variable was also very stable over time, being within the top 6% of the most stable linguistic features in WALS. This argues against the interpretation that savings behaviour affects the FTR variable. We controlled for historical relatedness using a Phylogenetic Generalised Least Squares test (PGLS) and the correlation remained robust (coefficient = 0.91, p = 0.03, 95% CI [1.71, 0.11]).

We explored some of the assumptions that went into the phylogenetic test. The original test assumed that the classifications used to generate the phylogeny reflected historical relatedness of cultural groups and that they are balanced across language families. We tested the latter assumption by using an alternative phylogenetic tree. Since there is no time depth data beyond the level of language families, we tested the correlation under a range of reasonable overall time depths and rates of change. Since the phylogeny between language families is not clear, we assumed a single common ancestor at a reasonable time depth. The correlation was robust to wide changes in these parameters. The correlation was also robust when permuting the data (the actual data exhibited a stronger link than 97% of random permutations of the data).

Despite being robust to many alternative tests, the correlation was not robust to all tests. In the replications of the regression on matched samples from [13], one of the regressions revealed no significant link between strong FTR and savings behaviour when controlling for language family (although the correlation was robust in more conservative models).

A stratified Mantel test permuting the data only within language families produced a stronger correlation than the actual data 5.5% of the time, failing the standard significance criterion of 5%.

The Phylogenetic Generalised Least Squares test was not significant when scaling branch lengths according to a Brownian Motion model (although this model fit the data less well than other branch length scaling assumptions). Also, the correlation was only significant in the PGLS test when assuming that the most recent split in the phylogeny happened relatively recently (within the last 630 years, making the assumptions about branch depth as in the materials and methods section). However, given the particular languages in the dataset (e.g. Dutch and Afrikaans) and the overall time-depth, this assumption seems reasonable.

The result was robust to the removal of any one particular data point, though a small number of datapoints were found to have strong influence over the results. The results were robust when removing these strong influences, though a larger sample of languages could lead to a more accurate picture.

The link between FTR and savings behaviour was not significant when running PGLS tests within each language family separately. In one case, the trend was in the opposite direction to the predicted one. This is perhaps the weakest point of the analysis. It suggests that the effect can only be observed looking across language families. However, the variation and statistical power is greatly reduced in these samples (number of languages ranging from 3 to 36 languages).

## Discussion

This paper used mixed effects modelling, along with other analyses, to assess the strength of the correlation between whether a language has an obligatorily marked future tense (FTR) and savings behaviour, while controlling for the relatedness of languages. In all analyses, the effect of FTR on savings behaviour is diminished when accounting for language relatedness. In the main mixed effects analysis, FTR does not significantly contribute to the explanation of the variation in savings behaviour. The result does not uphold the hypothesis that constraints on the expression of future tense affect the future-orientation of speakers’ choices.

The contrast between the original result and the current one highlights the danger of running cross-cultural comparisons without controlling for relations between cultures in time and space. Many other papers demonstrating correlations between linguistic and cultural phenomena have used almost no control for linguistic relatedness [25, 26, 29, 30] (although there are exceptions, e.g. [24]). Applying these controls is relatively easy within most statistical frameworks, and so researchers should be highly sceptical of results from studies that do not control for language family or contact.

In addition to the mixed effects model, several other methods were used to assess the correlation. These included a replication of the original regression on matched samples with controls for language family, Mantel tests controlling for phylogeny and geography and Phylogenetic Generalised Least Squares regression which included more fine-grained controls for phylogenetic relationships. In contrast to the mixed effects model, the correlation between FTR and savings behaviour remained robust in many cases, although not in all. The robustness of the correlation was surprising, especially to two of the authors (SR and JW), who expected the correlation to be a spurious artefact, as they have demonstrated for other correlations of this type [22]. As we discuss below, there are problems with these methods such as how to aggregate individual people’s data over languages and how to combine multiple language families in a single analysis. For example, the Phylogenetic Generalised Least Squares test controlled for the historical relationships between languages using a phylogenetic tree of language descent. We are aware that there is currently no consensus on how to integrate data over multiple language families. More detailed linguistic phylogenies would be invaluable for testing the kind of hypothesis studied here as well as for many other investigations. Results were more modest from tests with more fine-grained control of historical relatedness, possibly suggesting that a denser sampling of languages provides more accurate results. The selection of languages has a very low within-family density, possibly limiting the precision with which effects of language family can be estimated within a regression framework. Some hypotheses (particularly those relating to historical change) are better assessed by dense sampling of a single language family than a wide sampling of a few languages from many language families [74, 92, 93]. A better test of the hypothesis may be to gather more data on economic decisions and grammatical future tense orientation for a single language family and use phylogenetic methods. However, we suggest that, in this particular case, the mixed effects modelling approach is the most straightforward and comprehensive test of the hypothesis.

While we provide evidence to suggest that the original correlation reported by Chen is an artefact of the relatedness of languages, we discourage the view that the results disprove Chen’s general theory. The link between FTR and savings behaviour is one of a number of correlations discussed in [13] and subsequent work and the results here do not speak directly to any of these other results. However, the other results are susceptible to the same non-independence problem. Future work could re-analyse each correlation and control for relatedness. We also note that the correlation does appear to be stronger in some language families or geographic areas. The effect may be real for those cases, even if the effect does not hold across all languages. It may be the case that other properties of language or culture disrupt the effect of FTR on savings behaviour. It should be noted that the strength of the correlation in the original paper partly resulted from having non-independent datapoints.

The implication of the current paper is that the most informative next steps for exploring the hypothesis should involve experiments, simulations or more detailed idiographic case-studies, rather than more large-scale, cross-cultural statistical work. These alternative methods have more explanatory power to demonstrate causal links.

Below we discuss some further implications of the paper.

### Differences between methods

The mixed effects model suggested that the relationship between FTR and savings behaviour is just an artefact of historical and geographic relatedness. However, the relationship remained robust when using other methods. Two issues deserve discussion here: why do the different methods lead to different conclusions? and what are the implication of these differences to large-scale statistical studies of cultural traits?

To address the first issue, there are three aspects that set the mixed effects model apart from the other methods which arguably make it a better test. First, it does not require the aggregation of data over languages, cultures or countries. Secondly, it combines controls for both historical and geographical relatedness. Finally, the mixed effects framework allows the flexibility to ask specific questions.

Turning to the first difference, the socio-economic input data was raw responses from individual people. Other methods such as the PGLS are more typically run with one datapoint representing a whole language or culture. Indeed, there are few large-scale linguistic studies which have data at the individual speaker level: most concentrate on comparing typological variables between languages or dialects. Discrete categorisations of a typological variable over many speakers of course ignore variation between speakers, but are usually a suitable abstraction. Part of the reason that this abstraction is suitable is that language users typically strive to be co-ordinated. Other cultural traits may be different, however, especially economic traits where behaviour is contingent (e.g. large incomes in one section of the population will necessarily mean lower incomes in another). In this case, it may be more suitable to assess each individual respondent, rather than aggregating the data over respondents. That is, the aggregation masks some of the variation.

The second difference is the ability to control for phylogenetic and geographic relatedness separately. The mixed effects model included random effects for language family, country and continent. The PGLS framework uses a single covariance matrix to represent the relatedness of languages, which we used to control for historical relatedness only. The difference between the PGLS result and the mixed effects result may be due to the complex interaction between historical and geographic relatedness. In general, then, when exploring large-scale cross-cultural variation, both history and geography should be taken into account. This does not mean that the phylogenetic framework is not suitable. There are phylogenetic methods for combining historical and geographical controls, for example ‘geophylo’ techniques [94].

The phylogenetic methods may also have yielded a negative result if the resolution of the phylogenies was greater (e.g. more accurate branch length scaling within and between languages). However, given that the sample of the languages was very broad and not very deep, this issue is unlikely to make a large difference. Furthermore, the disadvantage of these techniques is that typically much more information is needed, in both phylogenetic and geographic resolution. In many cases, only categorical language groups may be currently available. Other statistical methods, such as mixed effects modelling, may be more suited to analysing data involving coarse categorical groups (see also Bickel’s ‘family bias method’, which uses coarse categorical data to control for correlations between families, [95]).

While the regression on matched samples did not aggregate and included some control for both historical and geographic relatedness, we suggest that the third difference is the flexibility of the framework. The mixed effects model allows researchers to precisely define the structure of the data, distinguishing between fixed-effect variables (e.g. FTR), and random-effect variables that represent a sample of the full data (e.g. language family). While in standard regression frameworks the error is collected under a single term, in a mixed effects framework there is a separate error term for each random effect. This allows more detailed explanations of the structure of the data through looking at the error terms, random slopes and intercepts of particular language families.

#### Supporting correlational claims from big data

In the section above, we described differences between the mixed effects modelling result, which suggested that the correlation between FTR and savings behaviour was an artefact of historical and geographical relatedness, and other methods, for which the correlation remained robust. Clearly, different methods leading to different results is concerning and raises several questions: How should researchers asses different results? How should results from different methods be integrated? Which method is best for dealing with large-scale cross-linguistic correlations?

The first two questions come down to a difference in perspectives on statistical methods: emphasising validity and emphasising robustness (for a fuller discussion, see Supporting information of [96]). Researchers who emphasise validity often choose a single test and try to categorically confirm or rule-out a correlation as a line of inquiry. The focus is usually on ensuring that the data is correct and appropriate and that all the assumptions of the test are met (so-called ‘internal validity’). While this is very suitable for many studies, when using noisy, non-experimentally controlled data, a robustness approach may be used. Researchers who emphasise high robustness often run a number of different tests to come to a more probabilistic conclusion about how related two variables are. This paper favours the latter approach, so we would encourage the perspective that the correlation between FTR and savings behaviour is robust to some but not all controls for non-independence. For the reasons outlined in the previous section, we think that the mixed effects model is the most appropriate test given the particular data and question at hand. We do not think that this is automatically the best solution for any given linguistic correlation.

While we think that multiple tests are informative, it might also be possible to criticise this approach as ‘anti-fishing’. That is, researchers could apply multiple tests until they find one that disconfirms the hypothesis. This is a difficult topic that does not have a straightforward answer. Previously, we have argued that one of the roles of large-scale cross-linguistic statistics is to act as feasibility studies for more extensive (and expensive) future research, rather than proof of a theory in itself [22]. In this light, a probabilistic conclusion may be all that is needed. However, we would argue that analyses of alternative data are more informative, if available, than multiple analyses of the same data.

For the question of whether a language’s grammar affects a speaker’s attitude to time and future-oriented decisions, as we have mentioned above, we believe tailored questionnaires or psycholinguistic priming studies are suitable next steps. A separate question is what the best approach is for researchers exploring large-scale cross-linguistic datasets in the future. The analyses in this paper, as well as in our other work [22, 66] suggests that any correlation should control for historical and geographical relatedness. All of the analyses performed in this paper were done with freely available data, with free software on ordinary laptops. There is no excuse for not doing these tests. Researchers should not seriously consider claims of correlations without these kinds of controls.

Of course, specific questions will require specific controls. In this paper we considered variables that address relevant concerns from economics (cultural attitudes, GDP, origin of legal system etc.). While economists are well informed about the importance of these variables, linguists may not be. We therefore suggest that interdisciplinary collaboration is very valuable in this kind of study.

Correlational studies will always be more controversial than results from controlled experiments. However, while there has been much criticism of Chen’s hypothesis (see the ‘Criticism’ section above), we note that, as with Atkinson’s work on phoneme diversity and migration [97], the controversy has at least created a debate and provided an opportunity for researchers to interface with each other.

#### Data sources and types in correlational studies

The source and type of the data are key elements that guide the decision about which statistical test to run. In this paper, the data consisted of individual-level responses. This allowed more powerful statistics based on individuals rather than aggregations. This is ‘big data’, but still only represents a sample of the total population. Therefore, the data can be noisier. As [71] notes, noisy signals increase in strength as the data size increases.

The data also came from a survey which was not designed with the current hypothesis in mind. This often means that the data are just proxies for the measures of interest. For example, the ‘language at home’ question was not linguistically informed and, consequently, matching answers to languages recognised by linguists was not straightforward. We also have little data on bilingualism or other language knowledge. The economic question is perhaps not ideal, either. Chen’s hypothesis is really about future-oriented behaviours, which may not be ideally captured in a categorical answer on saving or spending money. The survey was taken at different points in time, with some of the variation possibly being due to long-term economic changes. Now that Chen’s hypothesis is more fleshed out, it should be possible to design more tailored questionnaires.

## Conclusion

In the previous study, savings behaviour was found to correlate with the way an individual’s language marked the future tense. The explanation was a Whorfian effect of language on thought. In the current study, we applied controls for the relatedness of languages and cultures. The results were quite complicated, with the result being robust to some tests, but not to others. In general, the effect of language on behaviour was weaker when controlling for relatedness. In the cases where data was not aggregated and when the strictest controls for historical and geographical relatedness were applied (the mixed effects model with random slopes), the correlation between savings behaviour and future tense was not significant.

While we have demonstrated that exploring correlations in cross-cultural data is difficult, we have not disproved the idea that language can affect thought in a way that has tangible, long-term, aggregate effects on behaviour. In this particular case, we note that psychological priming experiments are possible, and potentially more informative. Despite this, cross-cultural statistical correlations may still have a role in motivating and guiding research.

## Materials and Methods

All data and code used to run the analyses are available in S1 Appendix (mixed effects models), S2 Appendix (Bayesian mixed effects models), S4 Appendix (raw WVS data), S5 Appendix (code for running mixed effects models), S6 Appendix (conversion from WVS languages to WALS and ISO languages), S7 Appendix (residualised savings behaviour variable), S8 Appendix (code for all other analyses).

### Data

The data on savings behaviour came from the World Values Survey [16]. This is a survey administered in 98 countries over two decades. The original study was done on the first five waves of survey results running from 1981 to 2009. All tests in this paper are done on this dataset. After the original submission of this paper, a new wave was released running from 2010 to 2014. Data from this 6th wave is included in one of the mixed effects models.

Datapoints from the World Values Survey (WVS) were linked to the Eurotyp typological variable FTR [17] and to the World Atlas of Language Structures [98] (see S6 and S9 Appendices). This involved identifying the name of the language in the WVS with the WALS language code. The data used in this paper can be seen in the Supporting information. The process was not entirely straightforward, since languages have many alternative names (e.g. “Bamanakan” is also known as “Bambara”). When there was not an immediate match in WALS, the alternative names were checked in the Ethnologue. Languages with alternative names were cross-referenced with the country in which the respondent completed the WVS. Not all languages in the WVS could be linked with data from WALS, in some cases because the data was not available, and in others because it was not clear what language was being referred to in WVS. These were excluded.

Another problem is that the languages listed in the WVS split and lump languages differently to WALS. For example, ‘Croatian’ and ‘Serbian’ are listed as different languages in WVS, but WALS includes them both under ‘Serbian-Croatian’ (the WVS ‘splits’ the languages while WALS ‘lumps’ them). Similarly, ‘Seraiki’ is considered a dialect of Panjabi (or Punjabi) in WALS. The converse problem is lumping: respondents who say they speak ‘Arabic’ may be describing one of several types of Arabic detailed in WALS.

When lumping occurs, some distinctions are based on the country that the respondent is answering the survey in (see the variable LangCountry in S6 Appendix). For example, respondents who say they speak Arabic from Egypt are coded as speaking Egyptian Arabic. Those who say they speak Arabic from Morocco are coded as speaking Moroccan Arabic. In more unclear situations, the population of speakers is taken into account. For example, the majority of ‘Chinese’ speakers in Malaysia will speak Mandarin, while the majority of ‘Chinese’ speakers in the USA will speak Cantonese. However, the situation in Australia is too close to call, so these are left uncoded. Some additional problems occur with dialect chains, such as in Thailand where respondents answered “Thai: Northern” or “Thai: Southern”, which don’t easily fit with a WALS language.

Cases from the WVS that do not have a response to the ‘Family savings’ question, or cases that are not linked with a WALS code are removed. Some languages had too few cases in the WVS or too few linguistic features in WALS, and so were removed. 142,630 cases were available for waves 3–5, and an additional 47,288 for the 6th wave.

Additional linguistic variables came from the World Atlas of Language Structures [98]. The linguistic variables in WALS were coded into binary or ranked variables. The coding scheme can be seen in the Supporting information. Where it made sense, variables were coerced to binary categories. This was done because the FTR variable is binary, and in order to increase the sample size in each category where possible. Some binary codings were taken from [99], since they use similar tests. The coding resulted in the following data: 70 binary linguistic features (features with only two possible values, features with only two values in the WVS sub-sample and some features from [99] that are coerced to binary features); 71 categorical features (the number of values has been collapsed in some cases, and for many categorical features some values don’t exist in the WVS sub-sample); 6 variables that can be meaningfully ranked; 22 variables that are not relevant (these are mainly categorisations of sub-types of languages or do not have enough variation in meaningful values); 19 variables for which there is no variation in the WVS sample; and 2 variables that are not directly linguistic in nature.

## Replication of regression on matched samples: Controlling for language family

The original analyses from [13] were replicated and extended to control for language family in order to provide some control for language relatedness.

### Method

The original analysis in [13] used regression on matched samples, also known as fixed-effect logistic regressions or conditional logistic regressions. The term ‘fixed effect’ used here should not be confused with the term ‘fixed effect’ in a mixed effect model. This test effectively splits the data into bins where every data point within a bin is identical for a set of variables (the ‘fixed effects’ such as age, employment status etc.), but differ on a key variable (here, FTR). A regression is then performed within each bin, comparing variation in FTR with variation in savings behaviour. The original regressions included fixed effects for country of residence, income decile within that country, marital status, sex, highest education level attained, age (in ten-year bins), number of children, survey wave, and religion (from a set of 74). The regressions described below added fixed effects for language family (so that no individuals were compared who spoke languages from different language families) and standard errors were clustered at the language family level.

Each set of regressions in the original study [13] was replicated, but below we only report the main test of the correlation between FTR strength and propensity to save. For the rest of the results, see the supporting information. The structure of each regression is illustrated in Table 4 and in Table 5. Each column is an individual regression and each row is a variable. Columns further to the right add increasing numbers of variables (from first to last row), and so are more conservative.

**Table 4:** Regression on matched samples predicting savings behaviour of individuals by the strength of future-time reference.

Regression	(1)	(2)	(3)	(4)	(5)	(6)
Strong_FTR	0.427 [0.076]**	0.480 [0.059]**	0.457 [0.065]**	0.452 [0.069]**	0.452 [0.075]**	0.481 [0.069]**
Altaic	0.774 [0.010]**	0.698 [0.030]**	1.017 [0.055]	1.340 [0.160]*	1.382 [0.200]*	1.490 [0.335]
Austro-Asiatic	1.875 [0.022]**	2.355 [0.135]**	2.483 [0.102]**	3.447 [0.213]**	3.830 [0.210]**	3.927 [0.306]**
Austronesian	1.417 [0.201]*	1.325 [0.147]*	1.489 [0.237]*	1.909 [0.394]**	1.932 [0.459]**	2.156 [0.542]**
Basque	10.287 [0.155]**	7.641 [1.014]**	8.710 [1.094]**	10.372 [1.647]**	11.815 [1.640]**	13.501 [3.511]**
Dravidian	1.858 [0.023]**	1.050 [0.080]	1.266 [0.086]**	1.753 [0.188]**	1.829 [0.226]**	1.840 [0.459]*
Finno-Ugric	1.130 [0.158]	0.682 [0.054]**	0.802 [0.103]	1.138 [0.162]	1.190 [0.240]	1.269 [0.328]
Indo-European	1.682 [0.014]**	1.229 [0.046]**	1.492 [0.108]**	2.049 [0.248]**	2.147 [0.296]**	2.276 [0.458]**
Japanese	1.006 [0.171]	0.719 [0.097]*	0.843 [0.154]	1.079 [0.363]	1.100 [0.410]	1.255 [0.365]
Kartvelian	0.138 [0.002]**	0.165 [0.022]**	0.202 [0.019]**	0.282 [0.072]**	0.272 [0.065]**	0.283 [0.079]**
Korean	1.000 [0.000]	1.000 [0.000]	1.000 [0.000]	1.000 [0.000]	1.000 [0.000]	1.000 [0.000]
Niger-Congo	2.317 [0.035]**	1.559 [0.134]**	2.117 [0.223]**	2.377 [0.381]**	2.402 [0.456]**	2.497 [0.565]**
Quechuan	1.031 [0.013]*	0.983 [0.043]	1.121 [0.103]	2.016 [0.204]**	2.179 [0.315]**	2.521 [0.886]**
Sino-Tibetan	1.212 [0.203]	1.208 [0.169]	1.256 [0.170]	1.691 [0.286]**	1.853 [0.302]**	2.009 [0.320]**
Tai-Kadai	1.817 [0.023]**	0.917 [0.056]	1.189 [0.052]**	1.485 [0.122]**	1.601 [0.128]**	1.619 [0.148]**
Legal origin:France		0.519 [0.047]**	0.588 [0.034]**	0.579 [0.080]**	0.593 [0.105]**	0.578 [0.093]**
Legal origin:Germany		0.509 [0.055]**	0.526 [0.042]**	0.472 [0.064]**	0.504 [0.075]**	0.486 [0.071]**
Legal origin:Scandinavia		0.837 [0.139]	0.859 [0.117]	0.720 [0.158]	0.813 [0.123]	0.809 [0.119]
LnPCGDP		1.180 [0.067]**	1.172 [0.062]**	1.226 [0.121]*	1.220 [0.120]*	1.212 [0.112]*
(max) Growth_PCGDP			0.219 [0.306]	0.112 [0.148]	0.113 [0.132]	0.100 [0.129]
Unemployed			0.514 [0.026]**	0.512 [0.029]**	0.510 [0.029]**	0.514 [0.029]**
(max) RealIntRate			0.995 [0.000]**	0.996 [0.001]**	0.995 [0.001]**	0.996 [0.001]**
(max) LegalRightsIndex			1.015 [0.023]	0.983 [0.020]	0.984 [0.024]	0.980 [0.020]
most people can be trusted					1.233 [0.057]**	1.234 [0.057]**
important in life: family					0.886 [0.033]**	0.887 [0.033]**
AverTrust					0.690 [0.214]	0.751 [0.228]
AverFamImp					0.727 [0.449]	0.720 [0.464]
LanguageShare						1.094 [0.181]
FTRShare						0.634 [0.197]
Observations	152,055	149,349	140,497	140,497	134,534	134,534

The table shows the results of regressions 1 to 6, predicting the savings behaviour of individuals in the WVS. This table is equivalent to Table 1 in [13]. Robust standard errors in brackets.

* significant at 5%;

** significant at 1%.

**Table 5:** Regressions on matched samples with language family fixed effects.

Regression	(7)	(8)	(9)	(10)	(11)	(12)
	FamSaved	FamSaved	FamSaved	FamSaved	FamSaved	FamSaved
Prediction_FTR	0.427 [0.076]**	0.559 [0.143]*	0.560 [0.146]*	0.618 [0.180]	0.591 [0.118]**	0.590 [0.114]**
Unemployed			0.669 [0.023]**	0.681 [0.044]**	0.679 [0.041]**	0.681 [0.042]**
most people can be trusted					1.065 [0.038]	1.067 [0.038]
important in life: family					0.948 [0.047]	0.949 [0.048]
important child qualities: thrift						1.129 [0.075]
Fixed effects						
Age × sex	Yes	Yes	Yes	Yes	Yes	Yes
Country × wave	No	Yes	Yes	Yes	Yes	Yes
Income × Edu	No	Yes	Yes	Yes	Yes	Yes
Married × Num of Children	No	No	No	Yes	Yes	Yes
All FEs Interacted	Yes	Yes	Yes	Yes	Yes	Yes
Language family	Yes	Yes	Yes	Yes	Yes	Yes
Observations	152,055	60,368	60,368	23,011	21768	21,768

The table shows the results of regressions 7 to 12, predicting the savings behaviour of individuals in the WVS. This table is equivalent to Table 3 in [13]. Robust standard errors are reported in brackets; all regressions are clustered at the country level.

* significant at 5%;

** significant at 1%.

The first set of regressions compared savings behaviour across countries. Regression 1 includes only non-choice fixed effects (age, sex) and language family. Regression 2 adds the origin of the country’s legal system and the log of its per-capita gross domestic product (PCGDP), following [100]. Regression 3 adds growth rate of PCGDP, level of unemployment, real interest rates, and the WDI legal-rights index. Regression 4 adds a fixed effect for continent. Regression 5 adds fixed effects for measures of trust from the WVS, including whether an individual thinks “most people can be trusted”, how important a respondent says that family is to them and the average of both these variables at the country level. Regression 6 adds controls for the share of a country which speaks a household’s language, and what share speak a language with the same kind of FTR.

The within-country regressions were also replicated from [13]. Regression 7 includes only non-choice variables (e.g. age and sex). Regressions 8 and 9 add fully interacted fixed effects for country, time, income and education. Regressions 10, 11 and 12 include controls for (nuclear) family structure. Regression 11 adds whether individuals’ responses to whether they think that others are generally trustworthy are on average. Regression 12 adds an individual’s attitude to thrift as an important value to each children.

### Results

Table 4 shows results for regressions 1 to 6. The strength of FTR is a significant predictor of savings behaviour in each regression. Individuals who speak a language with strong FTR are between 52% and 57% less likely to report having saved this year. The effect size is not very different from the original regression in [13] (mean coefficient over regressions in original = 0.453, in current = 0.458). As in the original, measures of trust at the family level are significant predictors (individuals who think others are generally trustworthy are on average 23% more likely to have saved this year).

However, the language family fixed effects are also significant predictors. In the most conservative regression (regression 6), 10 out of 14 language families have significant effects. Many of these also show larger effects than any in the original regressions. For example, speakers of Indo-European languages are 128% more likely to have saved this year than the average.

The results suggest that there are similarities between speakers of languages within the same language family. This suggests that a full exploration of the effect of language relatedness is warranted.

Table 5 shows that the strength of FTR when comparing individuals within a country remains a significant predictor for all but one of the regressions. The regression estimates that individuals who speak a language with strong FTR are between 57% (regression 7) and 39% (regression 10) less likely to report having saved in the current year. The results for regression 10, where only individuals from the same countries are compared, is not significant at the 5% level. This could be due to a loss of power because as other variables are introduced for regressions 11 and 12, which are more conservative, the FTR variable becomes significant again.

Results were not qualitatively different using the language families according to the alternative phylogeny.

### Aggregating savings behaviour over languages

The comparative methods below require a single value for each language representing the extent to which its speakers save money. A simple measure would be the mean probability of saving for speakers of each language. However, these means would hide imbalances in the data that could bias the results. For example, speakers of one language might happen to be more often employed than speakers of another. Since the regressions above demonstrate that employment is a significant predictor of savings behaviour, this would bias the results. Therefore, we use the residuals from regression 11 above (the deviation of each datapoint from the predicted values) aggregated over languages. This captures the variance in savings behaviour between languages that is not accounted for by other factors (age, sex, country, wave, income, education, marital status, number of children and language family, unemployment rate and attitudes to trust and thrift). The residuals are available in S7 Appendix. Selected tests were also done using the residuals from regression 9.

## Comparison of strength of correlation

### Method

The second extension to the original regression involved running the same analysis on matched samples with different linguistic features. Regression 3 from the analysis above (regression 3 from [13], Table 1, p. 703,) was run with other linguistic variables from WALS. The aim was to assess the strength of the correlation between savings behaviour and future tense by comparing it with the correlation between savings behaviour and comparable linguistic features. This is effectively a test of serendipidy: what is the probability of finding a ‘significant’ correlation with savings behaviour when choosing a linguistic variable at random? Put another way, because large, complex datasets are more likely to have spurious correlations, it is difficult to assess the strength of a correlation using standard conventions. One way to assess the strength of a correlation is by comparing it to similar correlations within the same data. If there are many linguistic features that equally predict economic behaviour, then the argument for a causal link between tense and economic behaviour is weakened. The null hypothesis is that future tense variable will not result in a correlation stronger than most of the other linguistic variables. For each variable in WALS, a logistic regression was run with the propensity to save money as the dependent variable and independent variables including the WALS variable, log per-capita GDP, the growth in per-capita GDP, unemployment rate, real interest rate, the WDI legal rights index and variables specifying the legal origins of the country in which the survey was carried out.

### Results

Two linguistic variables resulted in the likelihood function being non-concave which lead to non-convergence. These are removed from the analysis (the analysis was also run using independent variables to match regression 5 from [13], but this lead to 13 features failing to converge. In any case, the results from regression 3 and regression 5 were highly correlated, r = 0.97. Therefore, the results from regression 3 were used). The fit of the regressions was compared using AIC and BIC. The two measures were highly correlated (r = 0.999). The FTR variable lead to a lower BIC score (a better fit) than 99% of the linguistic variables.

Only two variables out of 192 provided a better fit: number of cases [101] and the position of the negative morpheme with respect to subject, object, and verb [102]. We note that the number of cases and the presence of strongly marked FTR are correlated (tau = 0.21, z = 3.2, p = 0.001). It may also be tempting to link it with studies that show a relationship between population size and morphological complexity [27]. However, there is not a significant difference in the mean populations for languages divided either by the (binarised) number of cases or by FTR (by number of cases: t = -0.4759, p = 0.6385; by FTR: t = -0.3044, p = 0.762). The effect of the order of negative morphemes is harder to explain, and can be attributed to a spurious correlation.

While the future tense variable does not provide the best fit, it is robust against controls for language family and performs better than the vast majority of linguistic variables, providing support that it its relationship with savings behaviour is not spurious.

### Independent tests

One way to test whether the correlation between savings and FTR is robust to historical relatedness is to compare independent samples. Here, we assume that languages in different language families are independent. We test whether samples of historically independent languages with strong FTR have a lower probability of saving than a random sample of languages.

Two random samples were chosen: the first sample was made up of one strong-FTR language from each language family. The second sample was made up of one weak-FTR language from each language family. The mean savings residual for each sample was compared. This process was repeated 10,000 times to estimate the probability that strong FTR languages have a lower mean propensity to save. If there was a significant relationship, then we would expect the strong FTR languages to have a lower savings propensity than the general sample for more than 95% of the samples.

Strong-FTR languages had a lower propensity to save in 99% of tests for the WALS family classification (also in 99% of the samples for the alternative classification). The correlation appears to be robust to this method. However, this is a coarser and more conservative test than the ones below, because the sample sizes are much reduced.

### Testing for phylogenetic signal

Structural features of language vary with regards to their stability over time [103]. Here, we assess the stability of FTR and savings behaviour.

#### Phylogenetic tree

**Fig 6:**
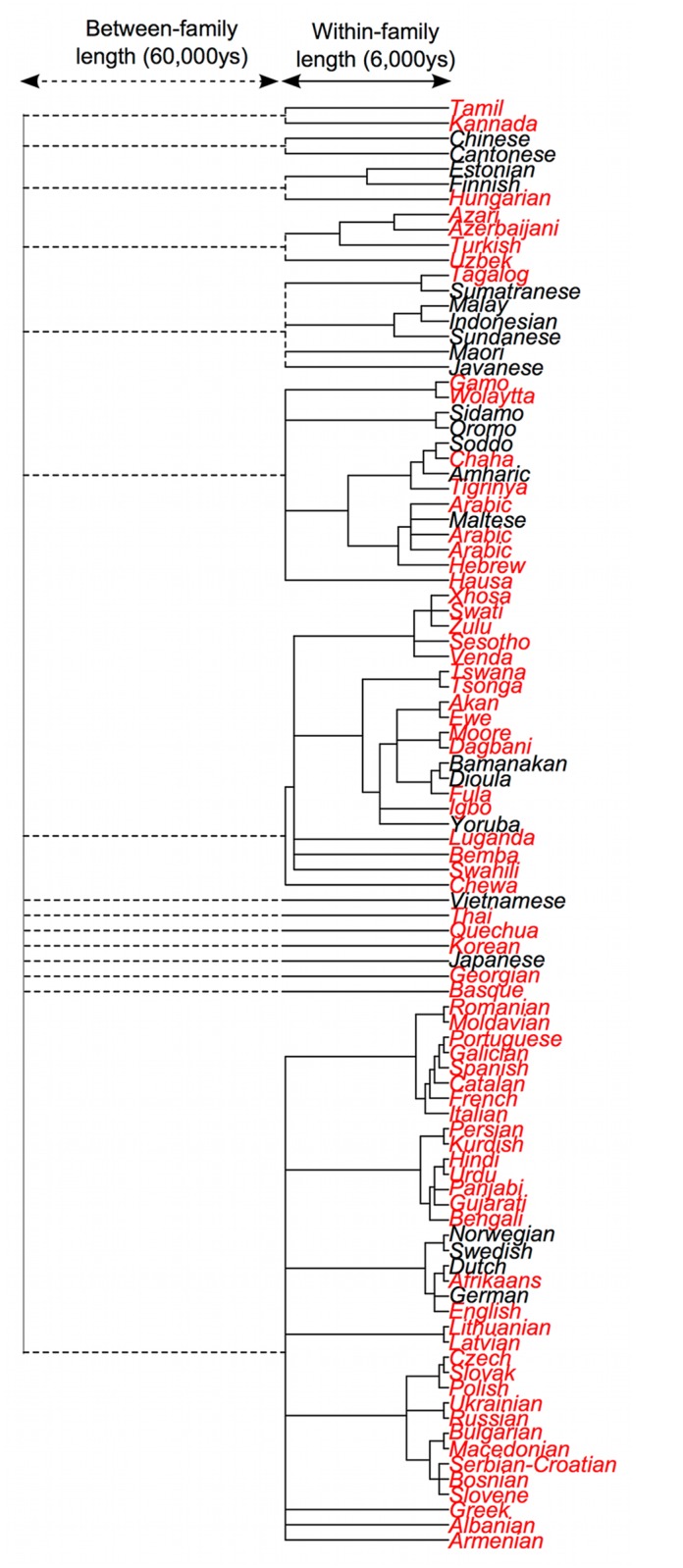
The phylogenetic tree used to control for language relatedness. Language names are shown with the colour representing the FTR variable (black = weak, red = strong).

Language classifications from the Ethnologue [104] were used to generate a phylogenetic tree (using the *AlgorithmTreeFromLabels* program [105]). This is done by grouping languages within the same family or genus under the same node, so that they are represented as being more related than languages from different families or genera. The branch lengths were scaled so that language families had a time depth of 6,000 years and language families were assumed to belong to a common root node 60,000 years ago. Although these are unrealistic assumptions for the actual history of languages, this procedure provides a reasonable way of preserving the assumption that each language family is effectively independent while specifying more fine-grained relationships within language families. Where appropriate, the tree was rooted using a language isolate as an outgroup. The Ethnologue tree is depicted in Fig 6. Note that we assume that linguistic traits and economic behaviours have the same inheritance histories. An alternative phylogenetic tree was produced using the classifications in [106]. These trees are used throughout the analyses in the following sections.

#### Results: Savings

The variable representing the economic behaviour of speakers of each language was taken from the residuals of the savings variable from regression 11. The phylogenetic trees described above were used to test for a phylogenetic signal in the data. The savings variable for each language is continuous, so we use the branch length scaling parameter *λ* [107] as calculated in the *geiger* package in R [108]. The savings variable has a *λ* of 0.757 for the Ethnologue tree, which is significantly different from a trait with no phylogenetic signal (log likelihood of model with *λ* = 0: 22.328, p = 0.000002) and significantly different from a trait changing by Brownian motion (log likelihood = 65.41, p = 6.09×10^−16^). The results were not qualitatively different for the alternative phylogenetic tree. This suggests that there is a strong phylogenetic signal in the savings variable. See the last section for the results using the residuals from regression 9.

#### Results: FTR

Since the FTR variable for languages is discrete, the strength of the phylogenetic signal was estimated using the Fritz and Purvis test [109] using the *caper* package in R [110]. The estimated strength of the signal D was 0.450, which is significantly different from an expected model with no phylogenetic structure (p ≈ 0), and significantly different from an expected Brownian motion model (p = 0.015). The results were not qualitatively different for the alternative phylogenetic tree. This suggests that the FTR variable has a strong phylogenetic signal. Indeed, given the phylogeny above, there are only 15 changes needed to capture the evolution of the 95 languages studied. Assuming that language families are 6,000 years old, that families have a common ancestor 60,000 years ago, this is one change every 78,000 years of cultural evolution (74,000 years for the alternative phylogeny, calculated using parsimony score from the R package *phangorn* version 1.99-1 [111]).

#### Results: Stability

We measured the stability of the FTR variable in the phylogeny using Maslova’s method of estimating transition probabilities (e.g. [74]). This method considers a binary linguistic feature with values *A* or *B*. Within a given time period, the value may change from *A* to *B*, from *B* to *A* or remain the same. The stability measure is the estimated probability of the value staying the same. However, this assumes that there are no unobserved switches (e.g. from *A* to *B* then back to *A*). To limit the number of unobserved switches, part of the measure is estimated from closely related languages, which limits the time period under consideration. See [103] and [76] for a fuller description of this measure. We used Dediu & Cysouw’s implementation [103] of this measure which groups languages under WALS genera and which has been previously applied to the variables in WALS. Dediu & Cysouw find that linguistic features in WALS vary widely in their stability, but different methods of measuring stability are highly correlated.

The relative ranking for the stability of the FTR variable is 0.916 (0 being most unstable feature, 1 being the most stable feature, actual value = 0.936), which ranks as the 9th most stable linguistic feature out of 139 features (top 6%). This suggests that the FTR variable is not affected by processes specific to language families or by borrowing.

The savings variable is very voatile in comparison. The proportion of people saving within a language changes on average by 29% over 10 years of surveys, and can change by up to 80%. For example, 100% of Italian speakers were saving money in 1997, compared to 18.4% in 2000. Some critics suggest that the savings behaviour might be affecting the FTR variable, instead of the other way around. However, the stability of the FTR variable argues against this interpretation.

While these stability estimates suggest that FTR is stable, we know that obligatory future tense emerged relatively recently in language families such as Indo-European (see [17]). The stability estimates may be affected by the small sample of languages within each family. A diachronic study would need to gather wider data on language families in order to make sure the estimates for ancestral state reconstruction were accurate. In any case, there is a strong phylogenetic signal in this data.

Another issue is that the FTR variable is slightly different from the kinds of linguistic variable usually considered for this kind of test. A language has a strongly marked FTR if it is *obligatory* to mark the future tense morphologically. Languages could change to innovate or lose ways of marking the future tense morphologically without changing the status of FTR. That is, the stability of FTR does not mean that tense morphology is stable or changes slowly. Indeed, tenses can show multiple parallel innovations within a language over a short time. However, the important point for this paper from the tests of phylogenetic signal and stability is that a language’s FTR status is not independent from its history.

### Mantel tests for correlated distance

The linguistic and economic traits exhibited by communities are not independent. They may be related by historical descent or by geographic diffusion. We conduct a series of tests to determine whether the correlation between FTR and savings is a spurious effect of spatial or phylogenetic auto-correlation.

#### Data

The patristic distances between each pair of languages in the WVS was calculated according to the phylogenetic tree generated from the Ethnologue. This produced a phylogenetic distance matrix for languages. The alternative phylogenetic tree produced using the classifications in [106] was used to produce an alternative phylogenetic distance matrix. Large values for a given pair of languages mean that they are more distantly related.

The variable representing the savings behaviour of speakers in each language was the residual of the savings variable from the regression controlling for many other factors (see section ‘Generating residuals after current economic conditions’). For each pair of languages, the absolute difference between this residual measure was used to produce a savings distance matrix for languages. Large values for a given pair of languages mean that they are more different in their savings behaviour. Using the residuals effectively means that the mantel tests that follow take into consideration the variation explained by other factors as well as geographic and phylogenetic distance.

The geographic locus of a language was taken from WALS. The geographic distance matrix was calculated using a program developed by [112]. This calculated the distance between two languages as the great circle distance between the two if they were on the same continent. If they were on different continents, a route connecting the two languages via plausible land bridges and sea crossings was taken into account. Large values for a given pair of language communities mean that they are physically further apart.

#### Method

Distance matrices were compared using Mantel and partial Mantel tests. Mantel tests compare the structure that is shared by two matrices, and can be used to control for variables that are spatially autocorrelated such as ecological factors. There has recently been some doubt about the reliability of Mantel tests [113–116]. However, [114] conclude that Mantel tests are not susceptible to type 1 errors (suggesting a relationship when there is none), but they should only be applied to data that can be expressed using pairwise comparisons, like geographic distance. In contrast, [113] demonstrate that the Mantel test has appropriate type 1 errors and is as strong as other methods for detecting spatial correlations such as Greary’s and Moran’s (see below). [115] demonstrate that Mantel tests are more susceptible to type 2 errors (suggesting no relationship when there is one) than standard regression analyses. [116] use a simulation to demonstrate that Mantel tests make type 1 errors when there is horizontal transmission. However, in the section above, we demonstrate that both the FTR variable and the residual savings variable had a strong phylogenetic signal. This is not what we would expect if there were large effects from horizontal transmission. Therefore, we see Mantel tests in this context as at least informative, since they provide a finer-grained comparison of the geographic relations between languages than, for example, geographic regions as random effects in a mixed-effects model.

Mantel tests are computed as follows. First, the real correlation between the two matrices is calculated. However, the significance of this correlation is not straightforward to compute, so it is computed. This is done by permuting one of the matrices, calculating the new correlation, then repeating this process to produce a distribution of correlations. If the real correlation is an outlier on this distribution, then the relationship between the two matrices is probably not due to chance.

Partial mantel tests, like partial correlations, allow the comparison of two variables while controlling for others. For example, if we want to compare matrix A and matrix B while controlling for a third matrix C. Two residual matrices are created by comparing A with C and B with C. These two residual matrices are then compared using a standard mantel test.

The Mantel tests were carried out with spearman rank correlations using the R package *ecodist* [117]. The significance levels were computed from a distribution of one million random permutations.

#### Results

Complete data for 95 languages were available. A summary of the results is shown in Table 6. FTR distance is significantly correlated with geographic distance (r = 0.151, p = 0.00132; Pearson r = 0.027, p = 0.598), and phylogenetic distance (r = 0.133, p = 0.008; Pearson r = 0.145, p = 0.0006). Savings distance is correlated with geographic distance (r = 0.091, p = 0.009; Pearson r = -0.012, p = 0.810) and with phylogenetic distance (r = 0.091, p = 0.009; Pearson r = 0.135, p = 0.00009). Phylogenetic distance and geographic distance are significantly correlated (r = 0.376, p = 0.000001, Pearson r = 0.107, p = 0.000001).

**Table 6:** Results for the Mantel tests.

Distance contrast	Mantel r	2.5%CI	97.5%CI	p
FTR vs Phylo	0.145	0.096	0.174	0.008 *
FTR vs Geo	0.027	0.109	0.196	0.001 *
Savings vs Phylo	0.141	0.020	0.099	0.159
Savings vs Geo	-0.018	0.058	0.131	0.009 *
Savings vs FTR	-0.161	-0.093	-0.186	0.002 *
Savings vs FTR (partial Phylo)	-0.144	-0.085	-0.176	0.002 *
Savings vs FTR (partial Geo)	-0.162	-0.081	-0.169	0.003 *
Savings vs FTR (partial Phylo and Geo)	-0.144	-0.080	-0.167	0.003 *
Savings vs FTR (partial Phylo) (alternative tree)	-0.144	-0.093	-0.181	0.002 *
Savings vs FTR (partial Phylo and Geo) (alternative tree)	-0.144	-0.080	-0.185	0.003 *
Phylo vs Geo	0.107	0.349	0.403	0.000001 *

Mantel regression coefficients, confidence intervals and estimated probabilities for different comparisons of distance between FTR strength, savings behaviour, phylogenetic history and geographic location. The final 5 comparisons compare savings behaviour and strength of FTR while partialling out the effects of phylogenetic distance and geographic distance.

* indicates significance at the 0.05 level.

FTR distance and savings distance are significantly correlated, as predicted by Chen (r = 0.135, p = 0.002; Pearson r = 0.130, p = 0.003). Furthermore, this correlation remains significant when controlling for phylogenetic distance (r = 0.128, p = 0.002; Pearson r = 0.113, p = 0.007), geographic distance (r = 0.123, p = 0.003; Pearson r = 0.130, p = 0.003) or both phylogenetic and geographic distance (r = 0.121, p = 0.003, Pearson r = 0.113, p = 0.006). This result is not qualitatively different using the alternative phylogenetic distance (Controlling for phylogenetic distance: r = 0.134, p = 0.002; Pearson r = 0.112, p = 0.007; controlling for phylogenetic and geographic distance: r = 0.124, p = 0.003; Pearson r = 0.113, p = 0.007). While the strength of the correlation between FTR and savings does decrease under these controls, the difference is relatively small.

#### Small populations

One problem with geographic distances when aggregating values over languages is that larger populations are likely to be less well represented by a single point. For example, while WALS suggests that the locus of English lies in England, it’s clearly spoken in many countries. Larger languages may also be affected by global contact. To address this issue, the same analyses were carried out on languages with small numbers of speakers, since a small language is more likely to be geographically concentrated. This was done by only considering languages with populations equal or less than the median value for the sample (51 languages with 6,535 or fewer speakers). That is, we tested whether the results hold when only considering small languages.

The results are summarised in Table 7. For the sample of small languages, FTR and savings were significantly correlated (r = 0.227, p = 0.00008). Furthermore, the correlation remains significant when controlling for phylogenetic distance (r = 0.217, p = 0.001), geographic distance (r = 0.226, p = 0.001;) or both phylogenetic and geographic distance (r = 0.216, p = 0.001;). The result is not qualitatively different using the alternative phylogeny (controlling for phylogeny: r = 0.217, p = 0.001; controlling for phylogeny and geography: r = 0.216, p = 0.001;). We note that the correlation coefficient is actually greater in this sample of small languages than in the full sample.

**Table 7:** Results for the Mantel tests for small populations.

Distance contrast	Mantel r	2.5%CI	97.5%CI	p
FTR vs Phylo	0.033	-0.014	0.092	0.616
FTR vs Geo	0.091	0.044	0.141	0.099
Savings vs Phylo	0.105	0.045	0.173	0.078
Savings vs Geo	0.082	0.024	0.153	0.101
Savings vs FTR	-0.188	-0.119	-0.268	0.004 *
Savings vs FTR (partial Phylo)	-0.186	-0.120	-0.272	0.004 *
Savings vs FTR (partial Geo)	-0.182	-0.120	-0.256	0.005 *
Savings vs FTR (partial Phylo and Geo)	-0.182	-0.121	-0.278	0.005 *
Savings vs FTR (partial Phylo) (alternative tree)	-0.188	-0.127	-0.273	0.004 *
Savings vs FTR (partial Phylo and Geo) (alternative tree)	-0.183	-0.124	-0.274	0.005 *
Phylo vs Geo	0.335	0.296	0.381	0.000001 *

Mantel regression coefficients, confidence intervals and estimated probabilities for different comparisons of distance between FTR strength, savings behaviour, phylogenetic history and geographic location. The final 5 comparisons compare savings behaviour and strength of FTR while partialling out the effects of phylogenetic distance and geographic distance.

* indicates significance at the 0.05 level.

### Stratified Mantel tests

The Mantel test works by randomly permuting the distance matrices. This might be unreasonable if we know something about the stratification of the data. For example, permutations that align distantly related languages may result in lower correlations. To test this, a stratified Mantel test was conducted using the R package *vegan* [118]. Permutations were only allowed within language families. The results are summarised in Table 8. Savings and FTR are significantly correlated (Kendall’s tau = 0.110, p = 0.009; Pearson r = 0.130, p = 0.012). This correlation remains robust when controlling for phylogeny (Kendall’s tau = 0.106, p = 0.008; Pearson r = 0.113, p = 0.023) and geography (Kendall’s tau = 0.103, p = 0.009; Pearson r = 0.130, p = 0.013).

**Table 8:** Results for stratified Mantel tests.

Distance contrast	Pearson r	p	Kendall’s tau	p
Savings vs FTR	0.161	0.007	0.122	0.003
Savings vs FTR (partial Phylo)	0.144	0.008	0.115	0.003
Savings vs FTR (partial Geo)	0.162	0.004	0.117	0.003

Mantel regression coefficients and estimated probabilities for different comparisons. The last two comparisons compare savings behaviour and strength of FTR while partialling out the effects of phylogenetic distance and geographic distance.

### Geographic Autocorrelation

**Fig 7:**
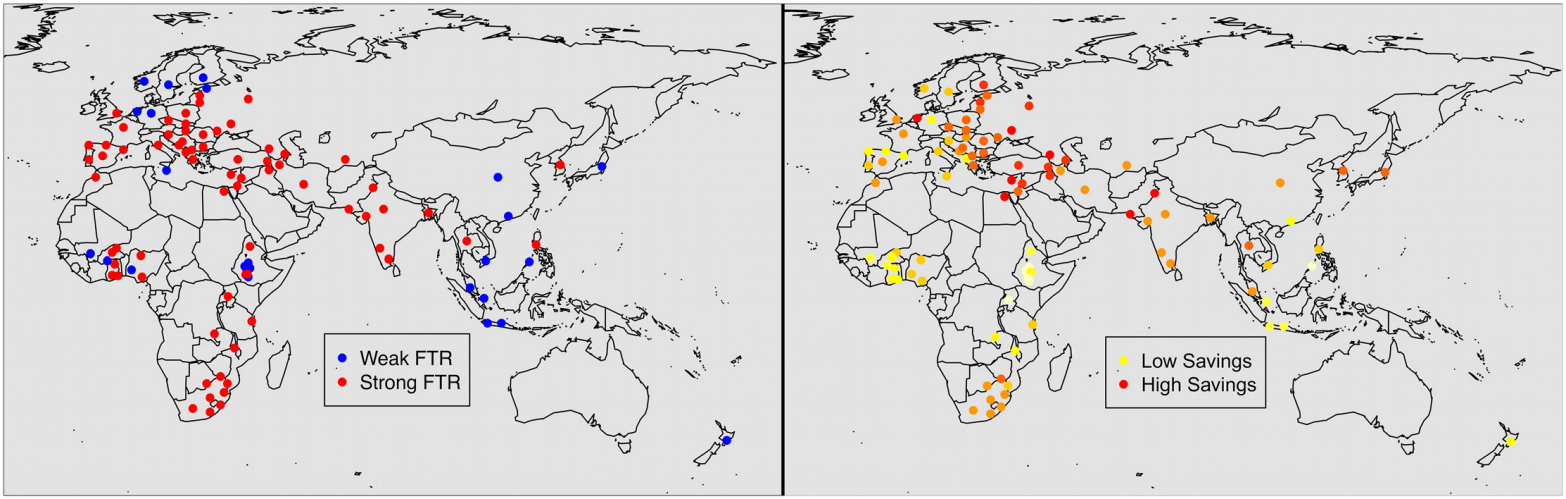
Geographic distribution of FTR and savings. The map on the left shows the geographic distribution ‘strong’ and ‘weak’ FTR languages. The map on the right shows the distribution of the savings residuals variable. Points represent languages and colour represents the value of the propensity to save residuals. The values range from a low propensity (yellow) to a high propensity(red).

One concern with the linguistic data was that it picked out European languages, which tend to be spoken in countries which are more economically prosperous than some other parts of the world (criticism by Dahl, see Fig 7). We can test this by looking at whether the data cluster into European and non-European regions. More generally, we would like to know whether the structure is random, clustered or dispersed. We can use geographic autocorrelation to assess this.

The savings residuals are geographically autocorrelated and are more dispersed than would be expected by chance (Moran’s I observed = -0.151, expected = -0.010, sd = 0.012, p = 9.6×10^−34^). Dispersion occurs when variants are in competition, and in the case of savings behaviour, this makes sense since the proportion of a population saving money constraints the proportion that spend. However, the FTR was also significantly dispersed (Moran’s I observed = -0.052, expected = -0.011, sd = 0.012, p = 0.0004).

The impact of the autocorrelation on the correlation between FTR and savings can be assessed using a geographically weighted regression (GWR), which weights observations by their geographic proximity. As in the PGLS analysis below, the savings residual was entered as the dependent variable and the FTR variable was entered as the independent variable. The geographically weighted regression resulted in a better fit than an OLS model (F = 0.3569, df1 = 72.94, df2 = 93.00, p = 0.000005). The variance of the FTR variable varies significantly across regions (F(15.5, 72.9) = 4.7×10^6^, p < 2.2×10^−16^).

In order for the OLS to converge, the data for Quechua had to be omitted. It’s likely that this is because Quechua is the only data point in the Americas, and so much further away from other data points. (Optimised bandwidth = 823.20, global FTR coefficient = -1.3548, n = 95, Effective number of parameters (residual: 2traceS—traceS’S): 29.29, Effective degrees of freedom (residual: 2traceS—traceS’S): 65.71, Sigma (residual: 2traceS—traceS’S): 1.03, Effective number of parameters (model: traceS): 23.61, Effective degrees of freedom (model: traceS): 71.39, Sigma (model: traceS): 0.99, Sigma (ML): 0.86, AICc (GWR p. 61, eq 2.33; p. 96, Eq 4.21): 307.836, AIC (GWR p. 96, Eq 4.22): 264.07, Residual sum of squares: 69.91, Quasi-global R2: 0.77; OLS residuals = 277.20, GWR residuals = 69.91.)

The FTR coefficients of the GWR do not appear to cluster by region. That is, the data does not appear to divide into ‘European’ and ‘non-European’ categories. In order to test the effect of geography, the predicted FTR values from the GWR were included into a PGLS model (predicting savings from FTR with observations weighted by a phylogenetic tree, see below). This effectively removes the variance due to geographic spread. The results from the PGLS show that the correlation between savings and FTR is weakened, but still significant (r = -1.814, t = -2.094, p = 0.039).

### Phylogenetic Generalised Least Squares

In order to test how savings behaviour is affected by FTR, a test is required that allows a continuous dependent variable (the savings residuals) and a discrete independent variable (FTR) that also takes the historical relationships between languages into account. Phylogenetic Generalised Least Squares (PGLS) is a method for calculating relationships between observations that are not independent. The expected similarity between each pair of observations is estimated to produce an expected covariance matrix. The covariance matrix is used to weight observations in a standard linear generalised least squares regression. When analysing observations that are related in a phylogeny, the similarity reflects the phylogenetic distance between two observations on the tree.

We assume that all language families are related to each other deep in time by a single node. This means that the similarity between any two languages from the different language families will be equally large, while the similarity between languages within a language family will be more fine-grained. To be clear, although we analyse languages from multiple families, we don’t make any assumptions about the topology of the tree between language families (apart from that they are connected deed in time somehow).

There are several methods of calculating the covariance matrix for a phylogeny. For example, the traits can be assumed to change according to Brownian motion (in which case PGLS is equivalent to an independent contrasts test), or the similarity between traits decreases exponentially with distance in the phylogeny (Ornsten-Uhlenbeck model). Some models, such as Grafen’s model re-scale the branch lengths, which we consider inappropriate here. The test of phylogenetic signal above demonstrated that both the FTR and savings variable were unlikely to be changing according to Brownian motion. Therefore, in the tests below we use Pagel’s covariance matrix [107], which takes a Brownian motion covariance matrix and scales the off-diagonal values by the estimated phylogenetic signal strength.

The correlation between FTR and the savings residuals was negative and significant (for Pagel’s covariance matrix, r = -0.91, df = 95 total, 93 residual, t = -2.23, p = 0.028, 95% CI [-1.71, -0.11]). The results were not qualitatively different for the alternative phylogeny (r = -1.00, t = -2.47, p = 0.01, 95% CI [-1.8, -0.21]). As reported above, adding the GWR coefficient did not qualitatively change the result (r = -1.814, t = -2.094, p = 0.039). This agrees with the correlation found in [13].

Out of three models tested, Pagel’s covariance matrix resulted in the best fit of the data, according to log likelihood (Pagel’s model: Log likelihood = -175.93; Brownian motion model: Log likelihood = -209.18, FTR r = 0.37, t = 0.878, p = 0.38; Ornsten-Uhlenbeck model: Log likelihood = -185.49, FTR r = -1.33, t = -3.29, p = 0.0014). The fit of the Pagel model was significantly better than the Brownian motion model (Log likelihood difference = 33.2, L-ratio = 66.49, p < 0.0001). The results were not qualitatively different for the alternative phylogeny (Pagel’s model: Log likelihood = -176.80; Brownian motion model: Log likelihood = -213.92, FTR r = 0.38, t = 0.88, p = 0.38; Ornsten-Uhlenbeck model: Log likelihood = -185.50, r = -1.327, t = -3.291, p = 0.001). The results for these tests run with the residuals from regression 9 are not qualitatively different (see the Supporting information).

#### PGLS within language families

The PGLS test was run within each language family. Only 6 families had enough observations and variation for the test. Table 9 shows the results. FTR did not significantly predict savings behaviour within any of these families. This contrasts with the results above, potentially for two reasons. First is the issue of combining all language families into a single tree. Assuming all families are equally independent and that all families have the same time-depth is not realistic. This may mean that families that do not fit the trend so well may be balanced out by families that do. In this case, the lack of significance within families suggests that the correlation is spurious. However, a second issue is that the results within language families have a very low number of observations and relatively little variation, so may not have enough statistical power. For instance, the result for the Uralic family is only based on 3 languages. In this case, the lack of significance within families may not be informative.

**Table 9:** PGLS tests within each language family.

Family	N	Pagel LnLik	Pagel FTR r	Pagel FTR p	BM LnLik	BM FTR r	BM FTR p
Afro-Asiatic	14	-25.01	-0.35	0.68	-25.26	0.12	0.88
Austronesian	7	-9.12	-0.57	0.6	-12.03	-2.61	0.16
Indo-European	36	-60.86	-0.61	0.49	-68.56	1.25	0.14
Niger-Congo	20	-22.41	-0.76	0.12	-22.89	-0.8	0.11
Uralic	3	-0.76	1.08	0.32	-0.76	1.08	0.36

The first and second column specify the language family and and the number of languages within that family. Columns 3 to 5 specify the log likelihood of the fit of the model, the correlation coefficient of the FTR variable and the associated probability according to Pagel’s covariance matrix. Columns 6 to 8 show the same measures according to a Brownian motion covariance matrix.

The use of PGLS with multiple language families and with a residualised variable is, admittedly, experimental. We believe that the general concept is sound, but further simulation work would need to be done to work out whether it is a viable method. One particularly thorny issue is how to integrate language families. We suggest that the mixed effects models are a better test of the correlation between FTR and savings behaviour in general (and the results of these tests suggest that the correlation is spurious).

#### Fragility of data

Since the sample size is relatively small, we would like to know whether particular data points are affecting the result. For all data points, the strength of the relationship between FTR and savings behaviour was calculated while leaving that data point out (a ‘leave one out’ analysis). The FTR variable remains significant when removing any given data point (maximum p-value for the FTR coefficient = 0.035). The influence of each point can be estimated using the dfbeta measure. The dfbeta for a given data point is the difference in the FTR coefficient when removing that data point, scaled by the standard error. That is, how drastic is the change in the results when removing the datapoint. The usual cut-off used to identify points with a large influence is $2\sqrt{n}$, where *n* is the number of data points (in our case *n* = 95, so the cutoff is 0.2). 6 of the 95 data points had absolute dfbetas greater than the cutoff (mean of all absolute dfbetas = 0.06, max = 0.52). These were (in descending order of influence): Dutch (Indo-European), German (Indo-European), Chaha (Afro-Asiatic), Egyptian Arabic (Afro-Asiatic), North Levantine Arabic (Afro-Asiatic) and Gamo (Afro-Asiatic). The direction of the influence was not always the same, however. Removing Dutch, Gamo and Chaha actually resulted in a stronger FTR coefficient.

The FTR variable remains significant when removing all of these data points from the analysis. Since the high-influence languages come from just two language families, we also ran a PGLS model excluding all Indo-European and Afro-Asiatic languages (50 languages). In this case, the FTR variable is no longer significant (coefficient = -0.94, t = -1.94, p = 0.059). However, the result is marginal and surprisingly robust given that more than half of the data was removed.

We can further test the robustness of the result by obtaining the distribution of results when the FTR variable is permuted (the values of FTR are randomly re-assigned to a language, without replacement). This is effectively the same as disrupting the phylogenetic history of the values. If a significant proportion of random permutations lead to a stronger correlation between FTR and savings behaviour, then this would suggest that the correlation in the real data could also be due to chance co-incidence of values.

There are around 10^22^ non-identical permutations of the 95 FTR data points, which is not feasible to exhaustively calculate, so 100,000 unique random permutations were tested. The correlation between savings behaviour and the permuted FTR variable was calculated with PGLS using Pagel’s covariance matrix, as above.

0.7% of the permutations resulted in regressions which converged and had a larger absolute regression coefficient for FTR. 0.3% had a regression coefficient that was negative and lower. Further analysis of the permutations leading to stronger results reveal that there is a median of 34 changes from the actual data (median changes for all permutations = 36). That is, the permutations that lead to stronger results are not the product of small changes to the original data. This suggests that the probability of the real data having a strong correlation due to chance is small.

We can explore the permutations to see whether changing values for a particular language is more likely to affect the results than changes to others. In the sample of permutations that lead to stronger results, the language most likely to be changed was Dutch (changed in 95% of the permutations that result in a lower p-value), suggesting that it has a high influence or is a possible outlier. This agrees with the leave-one-out analysis. Also in line with the leave-one-out analysis was the finding that Egyptian Arabic was changed least often in this sample (2% of permutations resulting in a better p-value).

The results above are for random permutations across the entire data. We can also permute the FTR variable within language families. This is a stricter test, since it results in permutations that are closer to the original data. 100,000 such permutations were tested.

3% of the permutations resulted in regressions which converged and had a larger absolute regression coefficient for FTR. 2.2% had a regression coefficient that was negative and lower. The permutations leading to stronger results have a median of 20 changes to the original data (minimum = 12, maximum = 28).

The savings variable can be subjected to the same permutation tests. 3.5% of the permutations resulted in regressions which converged and had a larger absolute regression coefficient for FTR. 1.8% had a regression coefficient that was negative and lower. Permutations which produced stronger results had an average of 25% difference in the savings values compared to the original savings values.

When savings were permuted only within language families, 6.1% of the permutations resulted in regressions which converged and had a larger absolute regression coefficient for FTR. 5.6% had a regression coefficient that was negative and lower. Given a significance threshold of 5%, this suggests that the correlation between FTR and savings is only marginally significant.

We can permute both the FTR and the savings variable within families. All of the regressions that were tested converged. 5.6% had a larger absolute regression coefficient for FTR. 5.1% had a a regression coefficient that was negative and lower. We also note that the number of permutations with strong positive correlations is much lower than the number with strong negative correlations (mean r = -0.23, t = -177.3, p < 0.0001), which demonstrates a bias towards negative results.

In this section, the aggregated data was permuted in order to assess how likely the real link between a language’s FTR and the savings behaviour of its speakers. The results show that the values assigned to languages can be swapped randomly within families and still produce correlations that are as strong. Put another way, we would expect equally strong correlations between a speaker’s savings behaviour and the FTR system of a language *related* to the one they speak. This weakens the claim that a language’s FTR system has an influence on its speakers’ savings behaviour.

### Branch length assumptions in PGLS

The phylogenetic trees used in the analysis above involved assumptions about the branch lengths (time depth) of the connections within and between language families. To test the dependence of the result on these assumptions, the same analysis was run with different assumptions about the time depth of the phylogenetic tree within and between language families. The time depth within language families was varied between 0 and 12,000 years (the main tree assumes 6,000 years) and the time depth between language families was varied between 0 and 80,000 years (the main tree assumes 60,000 years). See S11 Appendix.

The correlation between FTR and savings remained significant at the 0.05 level for all branch length assumptions tested (all correlations were negative). The most significant results come from short within-family branch lengths. The between-family branch lengths have little impact on the results. This suggests that the results of the PGLS analysis are robust against branch length assumptions. However, we note that we are assuming fairly simple branch length manipulations. Further tests could be carried out by estimating branch lengths from lexical data or cognates, etc.

### Branch depth assumptions in PGLS

The analyses above assume that splits in the phylogenetic tree happen at particular interval, as well as assumptions about the overall time-depth. In order to test this assumption about intervals, the branch lengths of the phylogenetic tree was scaled according to Grafen’s method. Internal nodes on the tree are assigned a height based on the number of descendants that node has. The heights are scaled so that the root height is 1, and then raised to the power *ρ*. Small values of *ρ* (<1) make the splits appear earlier in the tree and larger values of *ρ* make the splits appear later (see S11 Appendix).

Note that this method disrupts the distinctions between branch lengths within and between language families so that, for instance, language families with a larger number of languages tend to have common ancestors further back in time. In other words, this assumes a common rate of linguistic divergence for the whole tree, while the analyses above only make this assumption for the branches between language families.

The analysis above was run on trees using this method for a range of *ρ* values from 0.01 to 3. If we assume that the whole tree spans 60,000 years, when *ρ* is 0.01, 1 and 3, then 90% of the splits in the tree occur within the last 58,000, 16,600 and 350 years, respectively. Another way to think about this is that, when *ρ* is 0.01, 1 and 3, then the last divergence between two languages happened 57,000, 630, and 0.07 years ago. Clearly, *ρ* = 0.01 is too low and *ρ* = 3 is too high for a plausible estimate. The fit of the model is best for values of *ρ* around 0.15 (best model: 90% of splits occur within the last 37,500 years, last split 30,351 years ago, log likelihood = -170.8; worst model: *ρ* = 3, 90% of splits occur within the last 350 years, last split 0.07 years ago, log likelihood = -177.9). For the best-fitting model, the correlation between FTR and savings behaviour is not significant (correlation coefficient = -0.713, t = -1.79, p = 0.076). The test is significant at the 0.05 level for values of *ρ* above 1. That is, the correlation between FTR and savings behaviour is only robust, given this tree topology, when the cultures we have data for diverge relatively recently (within the last 16,600 years). This is fairly plausible given that we don’t have information on the phylogeny between language families. Put another way, the correlation is robust if we assume that the last divergence in languages happened less than 630 years ago. Given that the data includes Dutch and Afrikaans, which diverged in the 17th century [119], this seems like a reasonable assumption.

## PLGS for residuals from alternative regression

Since the repeated logits invert much more reliably with regression 9 than 11 (see section ‘Regressions with language family fixed effects’), selected tests were run with the residuals generated from regression 9. There were no qualitative differences. The correlation between savings and FTR was negative and significant (Pagel’s model -210.2021, FTR r = -1.529, t = -2.597 p = 0.01). The results were stronger, although the overall fit worse, for the Ornsten-Uhlenbeck model (log likelihood = -217.726, FTR r = -2.116, t = -3.710, p = 0.0004). Pagel’s model resulted in a better fit than the Brownian motion model (Brownian motion log likelihood = -252.704, FTR r = 0.675, t = 1.006, p = 0.317; log likelihood difference = 42.51, L.ratio = 85.003, p < 0.0001). Manipulating the branch length assumptions, as above, did not result in p-values for Pagel’s model above 0.033 (see S11 Appendix).

## Supporting Information

S1 Appendix Additional mixed effects modelling. (PDF)

S2 Appendix Additional Bayesian mixed effects modelling. (PDF)

S3 Appendix Convergence problems in fixed effect probability estimates. (PDF)

S4 Appendix Raw data for main mixed effects model. Raw data combined from the World Values Survey and various linguistic sources (see main text).
(ZIP)

S5 Appendix Mixed effects modelling code. R code for running the mixed effects models.
(ZIP)

S6 Appendix Table of links between World Values Survey and language WALS/iso codes. (ZIP)

S7 Appendix FTR residuals from the regression on matched samples. The residuals represent the amount of variation in the savings behaviour that is not explained by various factors in the regression (see section ‘Aggregating savings behaviour over languages’).
(ZIP)

S8 Appendix Code for running various tests. See README files within the various sub-folders.
(ZIP)

S9 Appendix How the World Values Survey was linked to WALS data. Notes on the various variables in the main data file, and how they were calculated.
(PDF)

S10 Appendix Distribution of savings behaviour by FTR type by country in the World Values Survey (waves 3–6). For each country, a graph showing the proportion of speakers of each language saving money for strong and weak FTR. Circle size indicates the proportion of observations for a given language. Red lines indicate the overall mean for the FTR type.
(PDF)

S11 Appendix Extra details on the PGLS robustness tests. Figures illustrating the manipulations of the phylogenetic tree used in the robustness tests for the PGLS analyses.
(PDF)
