# Supplementary material for: Future Tense and Economic Decisions: Controlling for Cultural Evolution
Source: PLoS One. 2015 Jul 17;10(7):e0132145. doi: 10.1371/journal.pone.0132145 (PMC4506144; doi:10.1371/journal.pone.0132145)
Supplement: S1 Appendix — (PDF) [file pone.0132145.s001.pdf]

Future Tense and economic savings: Additional mixed effects modelling

These models look at how savings behaviour is related to obligatory future tense marking, as part of Roberts, Winters & Chen (2014). The models are mixed effects models, run in R using the lme4 package version 1.17. The tables display the output from lme4. For more details on how to interpret the results, see the main paper and the lme4 manual.

For each model structure there are usually three analyses: A 'main model' with a fixed effect for FTR, a 'null model' without the fixed effect (but identical random effects), and a model comparison analysis between these two.

Tables 1 and 2 show summaries for the main models in the paper.

Table 1: **Results of the model comparison using mixed effects modelling using waves 1 to 5.**

	Within-model				Comparison with null model	
**Model (fixed effect)**	**Estimate**	**Std. Error**	**Z value**	**Pr (>z)**	χ^2	**Pr (>χ^2)**
Model A (Weak FTR)	0.41	0.17	2.40	0.01646	2.72	0.0992
Model B (No Trust)	-0.13	0.06	-2.20	0.02760	3.59	0.0583
Model C (Employment)	0.60	0.10	6.10	< 0.00001	17.41	< 0.0001
Model D (Sex female)	-0.11	0.05	-2.36	0.01851	4.10	0.0429

Results for fixed effects for various models (columns 2-5), and the comparison between the respective null model and the model with the given fixed effect. Data comes from waves 1 to 5 of the World Values Survey. Estimates are on a logit scale.

Table 2: **Results of the model comparison using mixed effects modelling using waves 1 to 6.**

	Within-model				Comparison with null model	
**Model (fixed effect)**	**Estimate**	**Std. Error**	**Z value**	**Pr (>z)**	χ^2	**Pr (>χ^2)**
Model E (Weak FTR)	0.26	0.16	1.58	0.11502	1.15	0.2830
Model F (No Trust)	-0.16	0.06	-2.65	0.00796	5.30	0.0213
Model G (Employment)	0.61	0.09	6.60	< 0.00001	18.66	< 0.0001
Model H (Sex female)	-0.12	0.03	-3.58	0.00035	6.54	0.0106

Results for fixed effects for various models (columns 2-5), and the comparison between the the respective null model and the model with the given fixed effect. Data comes from waves 1 to 6 of the World Values Survey. Estimates are on a logit scale.

1 Main models

	Estimate	Std. Error	z value	Pr(>$\|z\|$)
(Intercept)	-1.32	0.23	-5.73	< 0.00001
FTR weak	0.57	0.20	2.89	0.00391

Table 3: Main model with data from wave 3: Main model
saveYes ˜FTR + (1 + FTR | country) + (1 + FTR | Autotyp.area) + (1 + FTR | family)
(AIC = 42474.9, BIC = 42571.47)

	Estimate	Std. Error	z value	Pr(>$\|z\|$)
(Intercept)	-1.77	0.22	-8.04	< 0.00001

Table 4: Main model with data from wave 3: Null model.
saveYes ˜1 + (1 + FTR | country) + (1 + FTR | Autotyp.area) + (1 + FTR | family)
(AIC = 42477.79, BIC = 42565.57)

	Df	AIC	BIC	logLik	deviance	Chisq	Chi Df	Pr(>Chisq)
m2	10	42477.79	42565.57	-21228.89	42457.79			
m1	11	42474.90	42571.47	-21226.45	42452.90	4.89	1	0.0271

Table 5: Main model with data from wave 3: Model comparison between main and null model.

	Estimate	Std. Error	z value	Pr(>|z|)
(Intercept)	-1.37	0.17	-8.07	< 0.00001
FTR weak	0.72	0.22	3.33	0.00087

Table 6: Main model with data from wave 3 and 4: Main model
saveYes ˜FTR + (1 + FTR | country) + (1 + FTR | Autotyp.area) + (1 + FTR | family)
(AIC = 82826.75, BIC = 82930.04)

	Estimate	Std. Error	z value	Pr(>|z|)
(Intercept)	-1.80	0.16	-11.08	< 0.00001

Table 7: Main model with data from wave 3 and 4: Null model.
saveYes ˜1 + (1 + FTR | country) + (1 + FTR | Autotyp.area) + (1 + FTR | family)
(AIC = 82825.71, BIC = 82919.61)

	Df	AIC	BIC	logLik	deviance	Chisq	Chi Df	Pr(>Chisq)
m2	10	82825.71	82919.61	-41402.86	82805.71			
m1	11	82826.75	82930.04	-41402.38	82804.75	0.96	1	0.3276

Table 8: Main model with data from wave 3 and 4: Model comparison between main and null model.

	Estimate	Std. Error	z value	Pr(>|z|)
(Intercept)	-1.24	0.15	-8.19	< 0.00001
FTR weak	0.41	0.17	2.40	0.01646

Table 9: Main model with data from waves 3-5: Main model
saveYes ˜FTR + (1 + FTR | country) + (1 + FTR | Autotyp.area) + (1 + FTR | family)
(AIC = 141311.87, BIC = 141420.42)

	Estimate	Std. Error	z value	Pr(>|z|)
(Intercept)	-1.38	0.18	-7.56	< 0.00001

Table 10: Main model with data from waves 3-5: Null model.
saveYes ˜1 + (1 + FTR | country) + (1 + FTR | Autotyp.area) + (1 + FTR | family)
(AIC = 141312.59, BIC = 141411.27)

	Df	AIC	BIC	logLik	deviance	Chisq	Chi Df	Pr(>Chisq)
m2	10	141312.59	141411.27	-70646.29	141292.59			
m1	11	141311.87	141420.42	-70644.93	141289.87	2.72	1	0.0992

Table 11: Main model with data from waves 3-5: Model comparison between main and null model.

	Estimate	Std. Error	z value	Pr(>|z|)
(Intercept)	-1.24	0.14	-8.89	< 0.00001
FTR weak	0.26	0.16	1.58	0.11502

Table 12: Main model with data from waves 3-6: Main model
saveYes ˜FTR + (1 + FTR | country) + (1 + FTR | Autotyp.area) + (1 + FTR | family)
(AIC = 193847.37, BIC = 193959.07)

	Estimate	Std. Error	z value	Pr(>|z|)
(Intercept)	-1.33	0.13	-10.04	< 0.00001

Table 13: Main model with data from waves 3-6: Null model.
saveYes ˜1 + (1 + FTR | country) + (1 + FTR | Autotyp.area) + (1 + FTR | family)
(AIC = 193846.52, BIC = 193948.07)

	Df	AIC	BIC	logLik	deviance	Chisq	Chi Df	Pr(>Chisq)
m2	10	193846.52	193948.07	-96913.26	193826.52			
m1	11	193847.37	193959.07	-96912.69	193825.37	1.15	1	0.2830

Table 14: Main model with data from waves 3-6: Model comparison between main and null model.

2 Respondent sex

	Estimate	Std. Error	z value	Pr(>|z|)
(Intercept)	-1.33	0.17	-8.03	< 0.00001
sex female	-0.11	0.05	-2.36	0.01851

Table 15: Model predicting savings behaviour by respondent sex with data from waves 3-5: Main model
saveYes ˜sex2 + (1 + sex2 | country) + (1 + sex2 | Autotyp.area) + (1 + sex2 | family)
(AIC = 141032.63, BIC = 141141.16)

	Estimate	Std. Error	z value	Pr(>|z|)
(Intercept)	-1.26	0.16	-7.69	< 0.00001

Table 16: Model predicting savings behaviour by respondent sex with data from waves 3-5: Null model.
saveYes ˜1 + (1 + sex2 | country) + (1 + sex2 | Autotyp.area) + (1 + sex2 | family)
(AIC = 141034.73, BIC = 141133.4)

	Df	AIC	BIC	logLik	deviance	Chisq	Chi Df	Pr(>Chisq)
m2	10	141034.73	141133.40	-70507.36	141014.73			
m1	11	141032.63	141141.16	-70505.31	141010.63	4.10	1	0.0429

Table 17: Model predicting savings behaviour by respondent sex with data from waves 3-5: Model comparison between main and null model.

	Estimate	Std. Error	z value	Pr(>|z|)
(Intercept)	-1.24	0.12	-10.51	< 0.00001
sex female	-0.12	0.03	-3.58	0.00035

Table 18: Model predicting savings behaviour by respondent sex with data from waves 3-6: Main model
saveYes ˜sex2 + (1 + sex2 | country) + (1 + sex2 | Autotyp.area) + (1 + sex2 | family)
(AIC = 193483.67, BIC = 193595.36)

	Estimate	Std. Error	z value	Pr(>|z|)
(Intercept)	-1.11	0.14	-8.01	< 0.00001

Table 19: Model predicting savings behaviour by respondent sex with data from waves 3-6: Null model.
saveYes ˜1 + (1 + sex2 | country) + (1 + sex2 | Autotyp.area) + (1 + sex2 | family)
(AIC = 193488.21, BIC = 193589.74)

	Df	AIC	BIC	logLik	deviance	Chisq	Chi Df	Pr(>Chisq)
m2	10	193488.21	193589.74	-96734.10	193468.21			
m1	11	193483.67	193595.36	-96730.84	193461.67	6.54	1	0.0106

Table 20: Model predicting savings behaviour by respondent sex with data from waves 3-6: Model comparison between main and null model.

3 Respondent unemployment

	Estimate	Std. Error	z value	Pr(>|z|)
(Intercept)	-1.61	0.13	-12.12	< 0.00001
employed	0.60	0.10	6.10	< 0.00001

Table 21: Model predicting savings behaviour by respondent employment status with data from waves 3-5: Main model saveYes ˜unem + (1 + unem | country) + (1 + unem | Autotyp.area) + (1 + unem | family)
(AIC = 137555.38, BIC = 137663.68)

	Estimate	Std. Error	z value	Pr(>|z|)
(Intercept)	-1.88	0.15	-12.84	< 0.00001

Table 22: Model predicting savings behaviour by respondent employment status with data from waves 3-5: Null model. saveYes ˜1 + (1 + unem | country) + (1 + unem | Autotyp.area) + (1 + unem | family)
(AIC = 137570.78, BIC = 137669.24)

	Df	AIC	BIC	logLik	deviance	Chisq	Chi Df	Pr(>Chisq)
m2	10	137570.78	137669.24	-68775.39	137550.78			
m1	11	137555.38	137663.68	-68766.69	137533.38	17.41	1	< 0.0001

Table 23: Model predicting savings behaviour by respondent employment status with data from waves 3-5: Model comparison between main and null model.

	Estimate	Std. Error	z value	Pr(>$|z|$)
(Intercept)	-1.53	0.10	-15.35	< 0.00001
employed	0.61	0.09	6.60	< 0.00001

Table 24: Model predicting savings behaviour by respondent employment status with data from waves 3-6: Main model saveYes ˜unem + (1 + unem | country) + (1 + unem | Autotyp.area) + (1 + unem | family)
(AIC = 189063.38, BIC = 189174.86)

	Estimate	Std. Error	z value	Pr(>$|z|$)
(Intercept)	-1.67	0.13	-12.63	< 0.00001

Table 25: Model predicting savings behaviour by respondent employment status with data from waves 3-6: Null model. saveYes ˜1 + (1 + unem | country) + (1 + unem | Autotyp.area) + (1 + unem | family)
(AIC = 189080.04, BIC = 189181.39)

	Df	AIC	BIC	logLik	deviance	Chisq	Chi Df	Pr(>Chisq)
m2	10	189080.04	189181.39	-94530.02	189060.04			
m1	11	189063.38	189174.86	-94520.69	189041.38	18.66	1	< 0.0001

Table 26: Model predicting savings behaviour by respondent employment status with data from waves 3-6: Model comparison between main and null model.

4 Respondent trust

	Estimate	Std. Error	z value	Pr(>|z|)
(Intercept)	-1.35	0.15	-8.73	< 0.00001
No Trust	-0.13	0.06	-2.20	0.02760

Table 27: Model predicting savings behaviour by respondent trust with data from waves 3-5: Main model
saveYes ˜trustYes + (1 + trustYes | country) + (1 + trustYes | Autotyp.area) + (1 + trustYes | family)
(AIC = 132523.22, BIC = 132631.13)

	Estimate	Std. Error	z value	Pr(>|z|)
(Intercept)	-1.56	0.14	-10.82	< 0.00001

Table 28: Model predicting savings behaviour by respondent trust with data from waves 3-5: Null model.
saveYes ˜1 + (1 + trustYes | country) + (1 + trustYes | Autotyp.area) + (1 + trustYes | family)
(AIC = 132524.81, BIC = 132622.9)

	Df	AIC	BIC	logLik	deviance	Chisq	Chi Df	Pr(>Chisq)
m2	10	132524.81	132622.90	-66252.40	132504.81			
m1	11	132523.22	132631.13	-66250.61	132501.22	3.59	1	0.0583

Table 29: Model predicting savings behaviour by respondent trust with data from waves 3-5: Model comparison between main and null model.

	Estimate	Std. Error	z value	Pr(>|z|)
(Intercept)	-1.25	0.13	-9.81	< 0.00001
No Trust	-0.16	0.06	-2.65	0.00796

Table 30: Model predicting savings behaviour by respondent trust with data from waves 3-6: Main model
saveYes ˜trustYes + (1 + trustYes | country) + (1 + trustYes | Autotyp.area) + (1 + trustYes | family)
(AIC = 183898.58, BIC = 184009.74)

	Estimate	Std. Error	z value	Pr(>|z|)
(Intercept)	-1.46	0.10	-14.02	< 0.00001

Table 31: Model predicting savings behaviour by respondent trust with data from waves 3-6: Null model.
saveYes ˜1 + (1 + trustYes | country) + (1 + trustYes | Autotyp.area) + (1 + trustYes | family)
(AIC = 183901.88, BIC = 184002.94)

	Df	AIC	BIC	logLik	deviance	Chisq	Chi Df	Pr(>Chisq)
m2	10	183901.88	184002.94	-91940.94	183881.88			
m1	11	183898.58	184009.74	-91938.29	183876.58	5.30	1	0.0213

Table 32: Model predicting savings behaviour by respondent trust with data from waves 3-6: Model comparison between main and null model.

5 Sex, Unemployment and Trust

	Estimate	Std. Error	z value	Pr(>|z|)
(Intercept)	-1.52	0.13	-11.61	< 0.00001
FTR weak	0.28	0.15	1.92	0.05470
employed	0.67	0.02	28.97	< 0.00001

Table 33: Model predicting savings behaviour by FTR and unemployment (data from waves 3-6): Main model
saveYes ˜FTR + unem + (1 + FTR | country) + (1 + FTR | Autotyp.area) + (1 + FTR | family)
(AIC = 189182.24, BIC = 189303.85)

	Estimate	Std. Error	z value	Pr(>|z|)
(Intercept)	-1.52	0.13	-11.54	< 0.00001
FTR weak	0.27	0.15	1.76	0.07795
employed	0.67	0.02	29.28	< 0.00001
sex female	-0.19	0.01	-16.51	< 0.00001

Table 34: Model predicting savings behaviour by FTR, unemployment and sex (data from waves 3-6): Main model
saveYes ˜FTR + unem + sex2 + (1 + FTR | country) + (1 + FTR | Autotyp.area) + (1 + FTR | family)
(AIC = 188775.91, BIC = 188907.65)

	Estimate	Std. Error	z value	Pr(>|z|)
(Intercept)	-1.94	0.18	-10.97	< 0.00001
FTR weak	0.27	0.19	1.44	0.14872
employed	0.67	0.02	29.04	< 0.00001
sex female	-0.19	0.01	-16.76	< 0.00001
famImp2Not very important	0.11	0.12	0.93	0.35485
famImp2Rather important	0.35	0.11	3.17	0.00151
famImp2Very important	0.43	0.11	3.90	0.00010

Table 35: Model predicting savings behaviour by FTR, unemployment, sex and responses to questions on the importance of family (data from waves 3-6): Main model
saveYes ˜FTR + unem + sex2 + famImp2 + (1 + FTR | country) + (1 + FTR | Autotyp.area) + (1 + FTR | family)
(AIC = 188244.15, BIC = 188406.24)

	Estimate	Std. Error	z value	Pr(>|z|)
(Intercept)	-1.86	0.17	-10.96	< 0.00001
FTR weak	0.32	0.17	1.82	0.06937
employed	0.65	0.02	27.07	< 0.00001
sex female	-0.19	0.01	-16.47	< 0.00001
famImp2Not very important	0.13	0.13	1.02	0.30625
famImp2Rather important	0.35	0.11	3.07	0.00215
famImp2Very important	0.43	0.11	3.82	0.00014
No Trust	-0.25	0.01	-18.55	< 0.00001

Table 36: Model predicting savings behaviour by FTR, unemployment, sex, responses to questions on the importance of family and whether people can be trusted (data from waves 3-6): Main model
saveYes ~FTR + unem + sex2 + famImp2 + trustYes + (1 + FTR | country) + (1 + FTR | Autotyp.area) + (1 + FTR | family)
(AIC = 178767.06, BIC = 178938.46)

	Df	AIC	BIC	logLik	deviance	Chisq	Chi Df	Pr(>Chisq)
m.main	11	193847.37	193959.07	-96912.69	193825.37			
big1	12	189182.24	189303.85	-94579.12	189158.24	4667.13	1	< 0.0001
big2	13	188775.91	188907.65	-94374.96	188749.91	408.33	1	< 0.0001
big25	16	188244.15	188406.24	-94106.07	188212.15	537.77	3	< 0.0001
big3	17	178767.06	178938.46	-89366.53	178733.06	9479.08	1	< 0.0001

Table 37: Model comparison for models with different variables (data from waves 3-6). m.main = main model, then adding unemployment (big1), sex (big2), the importance of family (big25) and whether people can be trusted (big3)

6 Without random slopes

	Estimate	Std. Error	z value	Pr(>|z|)
(Intercept)	-1.35	0.13	-10.20	< 0.00001
FTR weak	0.12	0.12	0.96	0.33667

Table 38: Model without random slope for FTR by country (data from waves 3-6):
saveYes ˜FTR + (1 | country) + (1 + FTR | Autotyp.area) + (1 + FTR | family)
(AIC = 193862.7, BIC = 193954.09)

	Df	AIC	BIC	logLik	deviance	Chisq	Chi Df	Pr(>Chisq)
noCountrySlope	9	193862.70	193954.09	-96922.35	193844.70			
m.main	11	193847.37	193959.07	-96912.69	193825.37	19.33	2	0.0001

Table 39: Model comparison between main model and model without random slope for FTR by country (data from wave 6)

	Estimate	Std. Error	z value	Pr(>|z|)
(Intercept)	-1.21	0.13	-9.66	< 0.00001
FTR weak	0.31	0.17	1.86	0.06287

Table 40: Model without random slope for FTR by area (data from waves 3-6):
saveYes ˜FTR + (1 + FTR | country) + (1 | Autotyp.area) + (1 + FTR | family)
(AIC = 193849.28, BIC = 193940.67)

	Df	AIC	BIC	logLik	deviance	Chisq	Chi Df	Pr(>Chisq)
noAreaSlope	9	193849.28	193940.67	-96915.64	193831.28			
m.main	11	193847.37	193959.07	-96912.69	193825.37	5.90	2	0.0522

Table 41: Model comparison between main model and model without random slope for FTR by area (data from waves 3-6)

	Estimate	Std. Error	z value	Pr(>|z|)
(Intercept)	-1.25	0.13	-9.53	< 0.00001
FTR weak	0.28	0.15	1.84	0.06562

Table 42: Model without random slope for FTR by family (data from waves 3-6):
saveYes ˜FTR + (1 + FTR | country) + (1 + FTR | Autotyp.area) + (1 | family)
(AIC = 193844.84, BIC = 193936.23)

	Df	AIC	BIC	logLik	deviance	Chisq	Chi Df	Pr(>Chisq)
noFamilySlope	9	193844.84	193936.23	-96913.42	193826.84			
m.main	11	193847.37	193959.07	-96912.69	193825.37	1.47	2	0.4798

Table 43: Model comparison between main model and model without random slope for FTR by family (data from waves 3-6)

6.1 Summary

The comparisons above suggest that all random slopes are warranted, except for family. Below is a full model exploration without random slopes by family, and without random slopes by both family and area. Also, a model with no random slopes for any random effect.

	Estimate	Std. Error	z value	Pr(>|z|)
(Intercept)	-1.25	0.13	-9.53	< 0.00001
FTR weak	0.28	0.15	1.84	0.06562

Table 44: Model without random slope by family: Main model
saveYes ˜FTR + (1 + FTR | country) + (1 + FTR | Autotyp.area) + (1 | family)
(AIC = 193844.84, BIC = 193936.23)

	Estimate	Std. Error	z value	Pr(>|z|)
(Intercept)	-1.39	0.12	-11.10	< 0.00001

Table 45: Model without random slope by family: Null model.
saveYes ˜1 + (1 + FTR | country) + (1 + FTR | Autotyp.area) + (1 | family)
(AIC = 193844.42, BIC = 193925.65)

	Df	AIC	BIC	logLik	deviance	Chisq	Chi Df	Pr(>Chisq)
m2	8	193844.42	193925.65	-96914.21	193828.42			
m1	9	193844.84	193936.23	-96913.42	193826.84	1.58	1	0.2092

Table 46: Model without random slope by family: Model comparison between main and null model.

	Estimate	Std. Error	z value	Pr(>|z|)
(Intercept)	-1.17	0.12	-9.71	< 0.00001
FTR weak	0.51	0.15	3.42	0.00064

Table 47: Model without random slope by family and without random slope by area: Main model
saveYes ˜FTR + (1 + FTR | country) + (1 | Autotyp.area) + (1 | family)
(AIC = 193850.89, BIC = 193921.97)

	Estimate	Std. Error	z value	Pr(>|z|)
(Intercept)	-1.33	0.12	-11.29	< 0.00001

Table 48: Model without random slope by family and without random slope by area: Null model.
saveYes ˜1 + (1 + FTR | country) + (1 | Autotyp.area) + (1 | family)
(AIC = 193858.4, BIC = 193919.33)

	Df	AIC	BIC	logLik	deviance	Chisq	Chi Df	Pr(>Chisq)
m2	6	193858.40	193919.33	-96923.20	193846.40			
m1	7	193850.89	193921.97	-96918.45	193836.89	9.51	1	0.0020

Table 49: Model without random slope by family and without random slope by area: Model comparison between main and null model.

	Estimate	Std. Error	z value	Pr(>|z|)
(Intercept)	-1.25	0.12	-10.44	< 0.00001
FTR weak	0.20	0.05	3.83	0.00013

Table 50: Model without random slope for any fixed effect (waves 3-6): Main model
saveYes ˜FTR + (1 | country) + (1 | Autotyp.area) + (1 | family)
(AIC = 193917.46, BIC = 193968.23)

	Estimate	Std. Error	z value	Pr(>|z|)
(Intercept)	-1.27	0.13	-10.10	< 0.00001

Table 51: Model without random slope for any fixed effect (waves 3-6): Null model.
saveYes ˜1 + (1 | country) + (1 | Autotyp.area) + (1 | family)
(AIC = 193929.77, BIC = 193970.39)

	Df	AIC	BIC	logLik	deviance	Chisq	Chi Df	Pr(>Chisq)
m2	4	193929.77	193970.39	-96960.89	193921.77			
m1	5	193917.46	193968.23	-96953.73	193907.46	14.32	1	0.0002

Table 52: Model without random slope for any fixed effect (waves 3-6): Model comparison between main and null model.

7 Without random effects

	Estimate	Std. Error	z value	Pr(>$|z|$)
(Intercept)	-1.09	0.12	-9.33	$<$ 0.00001
FTR weak	0.38	0.14	2.73	0.00633

Table 53: Model without random effect for country (data from waves 3-6):
saveYes ˜FTR + (1 + FTR | Autotyp.area) + (1 + FTR | family)
(AIC = 200692.01, BIC = 200773.24)

	Df	AIC	BIC	logLik	deviance	Chisq	Chi Df	Pr(>Chisq)
noCountry	8	200692.01	200773.24	-100338.00	200676.01			
m.main	11	193847.37	193959.07	-96912.69	193825.37	6850.63	3	$<$ 0.0001

Table 54: Model comparison between main model and model without random effect for country.

	Estimate	Std. Error	z value	Pr(>$|z|$)
(Intercept)	-1.12	0.11	-10.30	$<$ 0.00001
FTR weak	0.31	0.15	2.11	0.03465

Table 55: Model without random effect for area (data from waves 3-6):
saveYes ˜FTR + (1 + FTR | country) + (1 + FTR | family)
(AIC = 193856.99, BIC = 193938.22)

	Df	AIC	BIC	logLik	deviance	Chisq	Chi Df	Pr(>Chisq)
noArea	8	193856.99	193938.22	-96920.49	193840.99			
m.main	11	193847.37	193959.07	-96912.69	193825.37	15.62	3	0.0014

Table 56: Model comparison between main model and model without random effect for area.

	Estimate	Std. Error	z value	Pr(>|z|)
(Intercept)	-1.32	0.14	-9.52	< 0.00001
FTR weak	0.25	0.15	1.66	0.09694

Table 57: Model without random effect for family (data from waves 3-6):
saveYes ˜FTR + (1 + FTR | country) + (1 + FTR | Autotyp.area)
(AIC = 193873.84, BIC = 193955.08)

	Df	AIC	BIC	logLik	deviance	Chisq	Chi Df	Pr(>Chisq)
noFamily	8	193873.84	193955.08	-96928.92	193857.84			
m.main	11	193847.37	193959.07	-96912.69	193825.37	32.47	3	< 0.0001

Table 58: Model comparison between main model and model without random effect for family.

8 With random intercept for year

	Estimate	Std. Error	z value	Pr(>|z|)
(Intercept)	-1.27	0.14	-9.11	< 0.00001
FTR weak	0.22	0.17	1.26	0.20609

Table 59: Model including random intercept by year, data from waves 3-6: Main model
saveYes ˜FTR + (1 + FTR | country) + (1 + FTR | Autotyp.area) + (1 + FTR | family) + (1 | year)
(AIC = 193401.16, BIC = 193523.02)

	Estimate	Std. Error	z value	Pr(>|z|)
(Intercept)	-1.35	0.13	-10.13	< 0.00001

Table 60: Model including random intercept by year, data from waves 3-6: Null model.
saveYes ˜1 + (1 + FTR | country) + (1 + FTR | Autotyp.area) + (1 + FTR | family) + (1 | year)
(AIC = 193399.9, BIC = 193511.6)

	Df	AIC	BIC	logLik	deviance	Chisq	Chi Df	Pr(>Chisq)
m2	11	193399.90	193511.60	-96688.95	193377.90			
m1	12	193401.16	193523.02	-96688.58	193377.16	0.73	1	0.3914

Table 61: Model including random intercept by year, data from waves 3-6: Model comparison between main and null model.

	Df	AIC	BIC	logLik	deviance	Chisq	Chi Df	Pr(>Chisq)
m.main	11	193847.37	193959.07	-96912.69	193825.37			
m1	12	193401.16	193523.02	-96688.58	193377.16	448.21	1	< 0.0001

Table 62: Model comparison between main model and model with random intercept by year

9 With random intercept for language

	Estimate	Std. Error	z value	Pr(>|z|)
(Intercept)	-1.16	0.12	-9.78	< 0.00001
FTR weak	0.46	0.17	2.80	0.00513

Table 63: Model including random intercept by language, data from waves 3-6: Main model
saveYes ˜FTR + (1 + FTR | country) + (1 + FTR | Autotyp.area) + (1 + FTR | family) + (1 | lang)
(AIC = 193497.67, BIC = 193619.53)

	Estimate	Std. Error	z value	Pr(>|z|)
(Intercept)	-1.39	0.10	-13.31	< 0.00001

Table 64: Model including random intercept by language, data from waves 3-6: Null model.
saveYes ˜1 + (1 + FTR | country) + (1 + FTR | Autotyp.area) + (1 + FTR | family) + (1 | lang)
(AIC = 193499.57, BIC = 193611.27)

	Df	AIC	BIC	logLik	deviance	Chisq	Chi Df	Pr(>Chisq)
m2	11	193499.57	193611.27	-96738.79	193477.57			
m1	12	193497.67	193619.53	-96736.84	193473.67	3.90	1	0.0483

Table 65: Model including random intercept by language, data from waves 3-6: Model comparison between main and null model.

	Df	AIC	BIC	logLik	deviance	Chisq	Chi Df	Pr(>Chisq)
m.main	11	193847.37	193959.07	-96912.69	193825.37			
m1	12	193497.67	193619.53	-96736.84	193473.67	351.70	1	< 0.0001

Table 66: Model comparison between main model and model with random intercept by language

10 With random intercept for language and year

	Estimate	Std. Error	z value	Pr(>|z|)
(Intercept)	-1.13	0.11	-10.33	< 0.00001
FTR weak	0.47	0.17	2.77	0.00569

Table 67: Model including random intercept by language and year, data from waves 3-6: Main model
saveYes ˜FTR + (1 + FTR | country) + (1 + FTR | Autotyp.area) + (1 + FTR | family) + (1 | lang) + (1 | year)
(AIC = 193035.43, BIC = 193167.44)

	Estimate	Std. Error	z value	Pr(>|z|)
(Intercept)	-1.42	0.12	-12.29	< 0.00001

Table 68: Model including random intercept by language and year, data from waves 3-6: Null model.
saveYes ˜1 + (1 + FTR | country) + (1 + FTR | Autotyp.area) + (1 + FTR | family) + (1 | lang) + (1 | year)
(AIC = 193033.85, BIC = 193155.7)

	Df	AIC	BIC	logLik	deviance	Chisq	Chi Df	Pr(>Chisq)
m2	12	193033.85	193155.70	-96504.93	193009.85			
m1	13	193035.43	193167.44	-96504.72	193009.43	0.42	1	0.5176

Table 69: Model including random intercept by language and year, data from waves 3-6: Model comparison between main and null model.

	Df	AIC	BIC	logLik	deviance	Chisq	Chi Df	Pr(>Chisq)
m.main	11	193847.37	193959.07	-96912.69	193825.37			
m.lang	12	193497.67	193619.53	-96736.84	193473.67	351.70	1	< 0.0001
m.langAndYear	13	193035.43	193167.44	-96504.72	193009.43	464.24	1	< 0.0001

Table 70: Model comparison between main model and model with random intercept by language

11 Model with continent instead of Autotyp area

	Estimate	Std. Error	z value	Pr(>$\|z\|$)
(Intercept)	-1.20	0.13	-9.13	$<$ 0.00001
FTR weak	0.27	0.14	1.96	0.05006

Table 71: Model including random effect for continent instead of Autotyp area, data from waves 3-6: Main model
saveYes ˜FTR + (1 + FTR | country) + (1 + FTR | continent) + (1 + FTR | family)
(AIC = 193862.3, BIC = 193973.99)

	Estimate	Std. Error	z value	Pr(>$\|z\|$)
(Intercept)	-1.32	0.15	-9.00	$<$ 0.00001

Table 72: Model including random effect for continent instead of Autotyp area, data from waves 3-6: Null model.
saveYes ˜1 + (1 + FTR | country) + (1 + FTR | continent) + (1 + FTR | family)
(AIC = 193861.9, BIC = 193963.44)

	Df	AIC	BIC	logLik	deviance	Chisq	Chi Df	Pr(>Chisq)
m2	10	193861.90	193963.44	-96920.95	193841.90			
m1	11	193862.30	193973.99	-96920.15	193840.30	1.60	1	0.2057

Table 73: Model including random effect for continent instead of Autotyp area, data from waves 3-6: Model comparison between main and null model.

12 Language genus instead of language family

	Estimate	Std. Error	z value	Pr(>|z|)
(Intercept)	-1.17	0.11	-10.78	< 0.00001
FTR weak	0.31	0.17	1.84	0.06602

Table 74: Model including random effect for language genus instead of language family (data from waves 3-6, models did not converge after 500,000 function evaluations) Main model
saveYes ˜FTR + (1 + FTR | country) + (1 + FTR | Autotyp.area) + (1 + FTR | genus wals)
(AIC = 193663.61, BIC = 193775.31)

	Estimate	Std. Error	z value	Pr(>|z|)
(Intercept)	-1.18	0.11	-10.33	< 0.00001

Table 75: Model including random effect for language genus instead of language family (data from waves 3-6, models did not converge after 500,000 function evaluations) Null model.
saveYes ˜1 + (1 + FTR | country) + (1 + FTR | Autotyp.area) + (1 + FTR | genus wals)
(AIC = 193663.74, BIC = 193765.28)

	Df	AIC	BIC	logLik	deviance	Chisq	Chi Df	Pr(>Chisq)
m2	10	193663.74	193765.28	-96821.87	193643.74			
m1	11	193663.61	193775.31	-96820.80	193641.61	2.13	1	0.1442

Table 76: Model including random effect for language genus instead of language family (data from waves 3-6, models did not converge after 500,000 function evaluations) Model comparison between main and null model.

13 Without immigrants

	Estimate	Std. Error	z value	Pr(>$|z|$)
(Intercept)	-1.26	0.13	-9.54	< 0.00001
FTR weak	0.28	0.17	1.63	0.10232

Table 77: Model excluding respondents whose mother or father were immigrants, data from waves 3-6: Main model
saveYes ˜FTR + (1 + FTR | country) + (1 + FTR | Autotyp.area) + (1 + FTR | family)
(AIC = 182043.07, BIC = 182154.17)

	Estimate	Std. Error	z value	Pr(>$|z|$)
(Intercept)	-1.37	0.16	-8.74	< 0.00001

Table 78: Model excluding respondents whose mother or father were immigrants, data from waves 3-6: Null model.
saveYes ˜1 + (1 + FTR | country) + (1 + FTR | Autotyp.area) + (1 + FTR | family)
(AIC = 182042.46, BIC = 182143.45)

	Df	AIC	BIC	logLik	deviance	Chisq	Chi Df	Pr(>Chisq)
m2	10	182042.46	182143.45	-91011.23	182022.46			
m1	11	182043.07	182154.17	-91010.54	182021.07	1.39	1	0.2390

Table 79: Model excluding respondents whose mother or father were immigrants, data from waves 3-6: Model comparison between main and null model.

<table>
<tr><th>form</th><th>aic</th><th>sig</th></tr>
<tr><td>saveYes ~ FTR + unem + sex2 + famImp2 + trustYes + (1 + FTR |country) + (1 + FTR|Autotyp.area) + (1 + FTR|family)</td><td>178767.06</td><td></td></tr>
<tr><td>saveYes ~ 1 + (1 + FTR |country) + (1 + FTR|Autotyp.area) + (1 + FTR|family)</td><td>182042.46</td><td>N/A</td></tr>
<tr><td>saveYes ~ FTR + (1 + FTR |country) + (1 + FTR|Autotyp.area) + (1 + FTR|family)</td><td>182043.07</td><td></td></tr>
<tr><td>saveYes ~ trustYes + (1 + trustYes |country) + (1 + trustYes|Autotyp.area) + (1 + trustYes|family)</td><td>183898.58</td><td>*</td></tr>
<tr><td>saveYes ~ 1 + (1 + trustYes |country) + (1 + trustYes|Autotyp.area) + (1 + trustYes|family)</td><td>183901.88</td><td>N/A</td></tr>
<tr><td>saveYes ~ FTR + unem + sex2 + famImp2 + (1 + FTR |country) + (1 + FTR|Autotyp.area) + (1 + FTR|family)</td><td>188244.15</td><td></td></tr>
<tr><td>saveYes ~ FTR + unem + sex2 + (1 + FTR |country) + (1 + FTR|Autotyp.area) + (1 + FTR|family)</td><td>188775.91</td><td></td></tr>
<tr><td>saveYes ~ unem + (1 + unem |country) + (1 + unem|Autotyp.area) + (1 + unem|family)</td><td>189063.38</td><td>*</td></tr>
<tr><td>saveYes ~ 1 + (1 + unem |country) + (1 + unem|Autotyp.area) + (1 + unem|family)</td><td>189080.04</td><td>N/A</td></tr>
<tr><td>saveYes ~ FTR + unem + (1 + FTR |country) + (1 + FTR|Autotyp.area) + (1 + FTR|family)</td><td>189182.24</td><td></td></tr>
<tr><td>saveYes ~ 1 + (1 + FTR |country) + (1 + FTR|Autotyp.area) + (1 + FTR|family) + (1|lang) + (1|year)</td><td>193033.85</td><td>N/A</td></tr>
<tr><td>saveYes ~ FTR + (1 + FTR |country) + (1 + FTR|Autotyp.area) + (1 + FTR|family) + (1|lang) + (1|year)</td><td>193035.43</td><td>*</td></tr>
<tr><td>saveYes ~ 1 + (1 + FTR |country) + (1 + FTR|Autotyp.area) + (1 + FTR|family) + (1|year)</td><td>193399.9</td><td>N/A</td></tr>
<tr><td>saveYes ~ FTR + (1 + FTR |country) + (1 + FTR|Autotyp.area) + (1 + FTR|family) + (1|year)</td><td>193401.16</td><td></td></tr>
<tr><td>saveYes ~ sex2 + (1 + sex2 |country) + (1 + sex2|Autotyp.area) + (1 + sex2|family)</td><td>193483.67</td><td>*</td></tr>
<tr><td>saveYes ~ 1 + (1 + sex2 |country) + (1 + sex2|Autotyp.area) + (1 + sex2|family)</td><td>193488.21</td><td>N/A</td></tr>
<tr><td>saveYes ~ FTR + (1 + FTR |country) + (1 + FTR|Autotyp.area) + (1 + FTR|family) + (1|lang)</td><td>193497.67</td><td>*</td></tr>
<tr><td>saveYes ~ 1 + (1 + FTR |country) + (1 + FTR|Autotyp.area) + (1 + FTR|family) + (1|lang)</td><td>193499.57</td><td>N/A</td></tr>
<tr><td>saveYes ~ FTR + (1 + FTR |country) + (1 + FTR|Autotyp.area) + (1 + FTR|genus wals)</td><td>193663.61</td><td></td></tr>
<tr><td>saveYes ~ 1 + (1 + FTR |country) + (1 + FTR|Autotyp.area) + (1 + FTR|genus wals)</td><td>193663.74</td><td>N/A</td></tr>
<tr><td>saveYes ~ 1 + (1 + FTR |country) + (1 + FTR|Autotyp.area) + (1|family)</td><td>193844.42</td><td>N/A</td></tr>
<tr><td>saveYes ~ FTR + (1 + FTR |country) + (1 + FTR|Autotyp.area) + (1|family)</td><td>193844.84</td><td></td></tr>
<tr><td>saveYes ~ FTR + (1 + FTR |country) + (1 + FTR|Autotyp.area) + (1|family)</td><td>193844.84</td><td></td></tr>
<tr><td>saveYes ~ 1 + (1 + FTR |country) + (1 + FTR|Autotyp.area) + (1 + FTR|family)</td><td>193846.52</td><td>N/A</td></tr>
<tr><td>saveYes ~ FTR + (1 + FTR |country) + (1 + FTR|Autotyp.area) + (1 + FTR|family)</td><td>193847.37</td><td></td></tr>
<tr><td>saveYes ~ FTR + (1 + FTR |country) + (1|Autotyp.area) + (1 + FTR|family)</td><td>193849.28</td><td></td></tr>
<tr><td>saveYes ~ FTR + (1 + FTR |country) + (1|Autotyp.area) + (1|family)</td><td>193850.89</td><td>*</td></tr>
<tr><td>saveYes ~ FTR + (1 + FTR |country) + (1 + FTR|family)</td><td>193856.99</td><td>*</td></tr>
<tr><td>saveYes ~ 1 + (1 + FTR |country) + (1|Autotyp.area) + (1|family)</td><td>193858.4</td><td>N/A</td></tr>
<tr><td>saveYes ~ 1 + (1 + FTR |country) + (1 + FTR|continent) + (1 + FTR|family)</td><td>193861.9</td><td>N/A</td></tr>
<tr><td>saveYes ~ FTR + (1 + FTR |country) + (1 + FTR|continent) + (1 + FTR|family)</td><td>193862.3</td><td></td></tr>
<tr><td>saveYes ~ FTR + (1 |country) + (1 + FTR|Autotyp.area) + (1 + FTR|family)</td><td>193862.7</td><td></td></tr>
<tr><td>saveYes ~ FTR + (1 + FTR |country) + (1 + FTR|Autotyp.area)</td><td>193873.84</td><td></td></tr>
<tr><td>saveYes ~ FTR + (1 |country) + (1|Autotyp.area) + (1|family)</td><td>193917.46</td><td>*</td></tr>
<tr><td>saveYes ~ 1 + (1 |country) + (1|Autotyp.area) + (1|family)</td><td>193929.77</td><td>N/A</td></tr>
<tr><td>saveYes ~ FTR + (1 + FTR |Autotyp.area) + (1 + FTR|family)</td><td>200692.01</td><td>*</td></tr>
</table>

Table 80: Summary of models for data from waves 1-6, sorted by AIC. The third column indicates whether the coefficient for the first fixed effect within the model is significant (though these estimates are unreliable).

14 Singular fits

In the main model (waves 3-5), the random slopes and the random intercepts are exactly correlated. This indicates that the model is overfitted, probably due to too few levels of the random effect. One way around this is to use Bayesian mixed effects models using the *blme* package (Dorie, 2011, see Chung et al., 2013). See Supporting material S2 for details.

References

Chung, Y., Rabe-Hesketh, S., Dorie, V., Gelman, A., and Liu, J. (2013). A nondegenerate penalized likelihood estimator for variance parameters in multilevel models. *Psychometrika*, 78(4):685–709. [28]

Dorie, V. (2011). blme: Bayesian linear mixed-effects models. *URL: http://CRAN. R-project. org/package= blme.* [28]
