# Supplementary material for: Future Tense and Economic Decisions: Controlling for Cultural Evolution
Source: PLoS One. 2015 Jul 17;10(7):e0132145. doi: 10.1371/journal.pone.0132145 (PMC4506144; doi:10.1371/journal.pone.0132145)
Supplement: S2 Appendix — (PDF) [file pone.0132145.s002.pdf]

Future tense and economic savings:
Additional Bayesian mixed effects modelling

When using the standard R package for mixed effects modelling (*lme4*, **?**) the random slopes and the random intercepts are exactly correlated. This indicates that the model is overfitted, probably due to too few levels of the random effect. One way around this is to use Bayesian mixed effects models using the *blme* package (Dorie, 2011, see **?**). Below are the results. There is no qualitative difference between the two types of mixed effects model: the FTR variable is not a significant predictor within the model, and does not significantly improve the fit of the model.

References

Dorie, V. (2011). blme: Bayesian linear mixed-effects models. *URL: http://CRAN. R-project. org/package= blme.* [1]

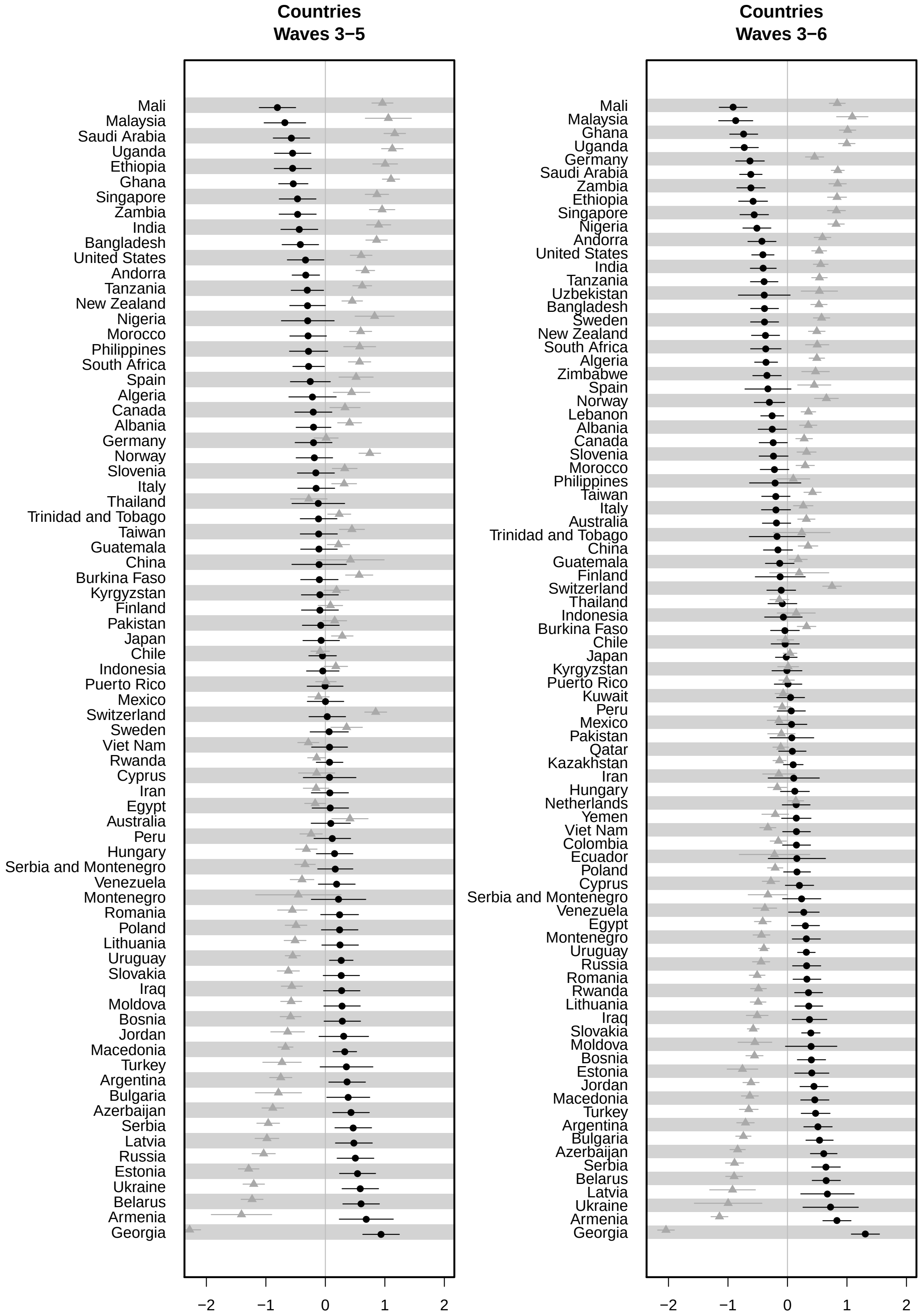

Figure 1: Random slopes (black dots) and random intercepts (grey triangles) for countries for the Bayesian mixed effects models, run on data from the WVS waves 3-5 (left) and waves 3-6 (right). Country names come from the WVS.

1 Main models

	Estimate	Std. Error	z value	Pr(>|z|)
(Intercept)	-1.13	0.22	-5.06	< 0.00001
FTR weak	0.59	0.44	1.33	0.18238

Table 1: Main model with data from waves 3-5: Main model
saveYes ˜FTR + (1 + FTR | country) + (1 + FTR | Autotyp.area) + (1 + FTR | family)
(AIC = 141328.16, BIC = 141436.71)

	Estimate	Std. Error	z value	Pr(>|z|)
(Intercept)	-1.34	0.20	-6.66	< 0.00001

Table 2: Main model with data from waves 3-5: Null model.
saveYes ˜1 + (1 + FTR | country) + (1 + FTR | Autotyp.area) + (1 + FTR | family)
(AIC = 141325.83, BIC = 141424.51)

	Df	AIC	BIC	logLik	deviance	Chisq	Chi Df	Pr(>Chisq)
m2	10	141325.83	141424.51	-70652.91	141305.83			
m1	11	141328.16	141436.71	-70653.08	141306.16	0.00	1	1.0000

Table 3: Main model with data from waves 3-5: Model comparison between main and null model.

	Estimate	Std. Error	z value	Pr(>$\vert z \vert$)
(Intercept)	-1.23	0.17	-7.17	< 0.00001
FTR weak	0.25	0.24	1.07	0.28425

Table 4: Main model with data from waves 3-6: Main model
saveYes ˜FTR + (1 + FTR | country) + (1 + FTR | Autotyp.area) + (1 + FTR | family)
(AIC = 193852, BIC = 193963.7)

	Estimate	Std. Error	z value	Pr(>$\vert z \vert$)
(Intercept)	-1.28	0.16	-7.98	< 0.00001

Table 5: Main model with data from waves 3-6: Null model.
saveYes ˜1 + (1 + FTR | country) + (1 + FTR | Autotyp.area) + (1 + FTR | family)
(AIC = 193852.33, BIC = 193953.87)

	Df	AIC	BIC	logLik	deviance	Chisq	Chi Df	Pr(>Chisq)
m2	10	193852.33	193953.87	-96916.17	193832.33			
m1	11	193852.00	193963.70	-96915.00	193830.00	2.33	1	0.1272

Table 6: Main model with data from waves 3-6: Model comparison between main and null model.

2 Respondent sex

	Estimate	Std. Error	z value	Pr(>$|z|$)
(Intercept)	-1.09	0.60	-1.81	0.06971
sex female	-0.02	0.17	-0.13	0.89678

Table 7: Model predicting savings behaviour by respondent sex with data from waves 3-5: Main model
saveYes ˜sex2 + (1 + sex2 | country) + (1 + sex2 | Autotyp.area) + (1 + sex2 | family)
(AIC = 141084.11, BIC = 141192.65)

	Estimate	Std. Error	z value	Pr(>$|z|$)
(Intercept)	-1.08	0.20	-5.50	< 0.00001

Table 8: Model predicting savings behaviour by respondent sex with data from waves 3-5: Null model.
saveYes ˜1 + (1 + sex2 | country) + (1 + sex2 | Autotyp.area) + (1 + sex2 | family)
(AIC = 141073.03, BIC = 141171.7)

	Df	AIC	BIC	logLik	deviance	Chisq	Chi Df	Pr(>Chisq)
m2	10	141073.03	141171.70	-70526.52	141053.03			
m1	11	141084.11	141192.65	-70531.05	141062.11	0.00	1	1.0000

Table 9: Model predicting savings behaviour by respondent sex with data from waves 3-5: Model comparison between main and null model.

	Estimate	Std. Error	z value	Pr(>|z|)
(Intercept)	-0.17	0.55	-0.32	0.75251
sex female	0.13	0.13	1.01	0.31171

Table 10: Model predicting savings behaviour by respondent sex with data from waves 3-6: Main model
saveYes ˜sex2 + (1 + sex2 | country) + (1 + sex2 | Autotyp.area) + (1 + sex2 | family)
(AIC = 193538.02, BIC = 193649.71)

	Estimate	Std. Error	z value	Pr(>|z|)
(Intercept)	-1.09	0.15	-7.12	< 0.00001

Table 11: Model predicting savings behaviour by respondent sex with data from waves 3-6: Null model.
saveYes ˜1 + (1 + sex2 | country) + (1 + sex2 | Autotyp.area) + (1 + sex2 | family)
(AIC = 193518.76, BIC = 193620.29)

	Df	AIC	BIC	logLik	deviance	Chisq	Chi Df	Pr(>Chisq)
m2	10	193518.76	193620.29	-96749.38	193498.76			
m1	11	193538.02	193649.71	-96758.01	193516.02	0.00	1	1.0000

Table 12: Model predicting savings behaviour by respondent sex with data from waves 3-6: Model comparison between main and null model.

3 Respondent unemployment

	Estimate	Std. Error	z value	Pr(>|z|)
(Intercept)	-1.50	0.21	-7.05	< 0.00001
employed	0.75	0.25	3.06	0.00225

Table 13: Model predicting savings behaviour by respondent employment status with data from waves 3-5: Main model saveYes ˜unem + (1 + unem | country) + (1 + unem | Autotyp.area) + (1 + unem | family)
(AIC = 137578.61, BIC = 137686.92)

	Estimate	Std. Error	z value	Pr(>|z|)
(Intercept)	-1.69	0.15	-11.48	< 0.00001

Table 14: Model predicting savings behaviour by respondent employment status with data from waves 3-5: Null model. saveYes ˜1 + (1 + unem | country) + (1 + unem | Autotyp.area) + (1 + unem | family)
(AIC = 137594.11, BIC = 137692.57)

	Df	AIC	BIC	logLik	deviance	Chisq	Chi Df	Pr(>Chisq)
m2	10	137594.11	137692.57	-68787.05	137574.11			
m1	11	137578.61	137686.92	-68778.31	137556.61	17.50	1	< 0.0001

Table 15: Model predicting savings behaviour by respondent employment status with data from waves 3-5: Model comparison between main and null model.

	Estimate	Std. Error	z value	Pr(>|z|)
(Intercept)	-1.57	0.27	-5.73	< 0.00001
employed	0.63	0.21	3.07	0.00217

Table 16: Model predicting savings behaviour by respondent employment status with data from waves 3-6: Main model saveYes ˜unem + (1 + unem | country) + (1 + unem | Autotyp.area) + (1 + unem | family)
(AIC = 189089.34, BIC = 189200.82)

	Estimate	Std. Error	z value	Pr(>|z|)
(Intercept)	-1.71	0.11	-16.13	< 0.00001

Table 17: Model predicting savings behaviour by respondent employment status with data from waves 3-6: Null model. saveYes ˜1 + (1 + unem | country) + (1 + unem | Autotyp.area) + (1 + unem | family)
(AIC = 189086.92, BIC = 189188.26)

	Df	AIC	BIC	logLik	deviance	Chisq	Chi Df	Pr(>Chisq)
m2	10	189086.92	189188.26	-94533.46	189066.92			
m1	11	189089.34	189200.82	-94533.67	189067.34	0.00	1	1.0000

Table 18: Model predicting savings behaviour by respondent employment status with data from waves 3-6: Model comparison between main and null model.

4 Respondent trust

	Estimate	Std. Error	z value	Pr(>$\|z\|$)
(Intercept)	-1.22	0.18	-6.77	< 0.00001
No Trust	0.08	0.25	0.34	0.73384

Table 19: Model predicting savings behaviour by respondent trust with data from waves 3-5: Main model saveYes ˜trustYes + (1 + trustYes | country) + (1 + trustYes | Autotyp.area) + (1 + trustYes | family) (AIC = 132574.44, BIC = 132682.34)

	Estimate	Std. Error	z value	Pr(>$\|z\|$)
(Intercept)	-1.13	0.30	-3.74	0.00019

Table 20: Model predicting savings behaviour by respondent trust with data from waves 3-5: Null model. saveYes ˜1 + (1 + trustYes | country) + (1 + trustYes | Autotyp.area) + (1 + trustYes | family) (AIC = 132573.1, BIC = 132671.19)

	Df	AIC	BIC	logLik	deviance	Chisq	Chi Df	Pr(>Chisq)
m2	10	132573.10	132671.19	-66276.55	132553.10			
m1	11	132574.44	132682.34	-66276.22	132552.44	0.66	1	0.4156

Table 21: Model predicting savings behaviour by respondent trust with data from waves 3-5: Model comparison between main and null model.

	Estimate	Std. Error	z value	Pr(>|z|)
(Intercept)	-1.33	0.19	-6.86	< 0.00001
No Trust	-0.04	0.48	-0.08	0.93882

Table 22: Model predicting savings behaviour by respondent trust with data from waves 3-6: Main model saveYes ˜trustYes + (1 + trustYes | country) + (1 + trustYes | Autotyp.area) + (1 + trustYes | family) (AIC = 183958.81, BIC = 184069.98)

	Estimate	Std. Error	z value	Pr(>|z|)
(Intercept)	-1.20	0.23	-5.21	< 0.00001

Table 23: Model predicting savings behaviour by respondent trust with data from waves 3-6: Null model. saveYes ˜1 + (1 + trustYes | country) + (1 + trustYes | Autotyp.area) + (1 + trustYes | family) (AIC = 183964.41, BIC = 184065.47)

	Df	AIC	BIC	logLik	deviance	Chisq	Chi Df	Pr(>Chisq)
m2	10	183964.41	184065.47	-91972.21	183944.41			
m1	11	183958.81	184069.98	-91968.41	183936.81	7.60	1	0.0058

Table 24: Model predicting savings behaviour by respondent trust with data from waves 3-6: Model comparison between main and null model.

5 Sex, Unemployment and Trust

	Estimate	Std. Error	z value	Pr(>|z|)
(Intercept)	-1.51	0.17	-8.91	< 0.00001
FTR weak	0.27	0.23	1.15	0.25055
employed	0.67	0.02	28.90	< 0.00001

Table 25: Model predicting savings behaviour by FTR and unemployment (data from waves 3-6): Main model
saveYes ˜FTR + unem + (1 + FTR | country) + (1 + FTR | Autotyp.area) + (1 + FTR | family)
(AIC = 189187.33, BIC = 189308.94)

	Estimate	Std. Error	z value	Pr(>|z|)
(Intercept)	-1.51	0.17	-8.90	< 0.00001
FTR weak	0.26	0.23	1.12	0.26122
employed	0.67	0.02	29.14	< 0.00001
sex female	-0.19	0.01	-16.50	< 0.00001

Table 26: Model predicting savings behaviour by FTR, unemployment and sex (data from waves 3-6): Main model
saveYes ˜FTR + unem + sex2 + (1 + FTR | country) + (1 + FTR | Autotyp.area) + (1 + FTR | family)
(AIC = 188780.9, BIC = 188912.64)

	Estimate	Std. Error	z value	Pr(>|z|)
(Intercept)	-1.92	0.21	-9.01	< 0.00001
FTR weak	0.26	0.23	1.11	0.26623
employed	0.67	0.02	28.96	< 0.00001
sex female	-0.19	0.01	-16.76	< 0.00001
famImp2Not very important	0.11	0.14	0.79	0.43007
famImp2Rather important	0.35	0.13	2.68	0.00735
famImp2Very important	0.43	0.13	3.30	0.00098

Table 27: Model predicting savings behaviour by FTR, unemployment, sex and responses to questions on the importance of family (data from waves 3-6): Main model
saveYes ˜FTR + unem + sex2 + famImp2 + (1 + FTR | country) + (1 + FTR | Autotyp.area) + (1 + FTR | family)
(AIC = 188249.06, BIC = 188411.15)

	Estimate	Std. Error	z value	Pr(>|z|)
(Intercept)	-1.85	0.21	-8.70	< 0.00001
FTR weak	0.30	0.22	1.38	0.16713
employed	0.65	0.02	26.99	< 0.00001
sex female	-0.19	0.01	-16.46	< 0.00001
famImp2Not very important	0.13	0.15	0.87	0.38253
famImp2Rather important	0.35	0.14	2.59	0.00958
famImp2Very important	0.43	0.13	3.21	0.00134
No Trust	-0.25	0.01	-18.51	< 0.00001

Table 28: Model predicting savings behaviour by FTR, unemployment, sex, responses to questions on the importance of family and whether people can be trusted (data from waves 3-6): Main model
saveYes ˜FTR + unem + sex2 + famImp2 + trustYes + (1 + FTR | country) + (1 + FTR | Autotyp.area) + (1 + FTR | family)
(AIC = 178772.34, BIC = 178943.74)

	Df	AIC	BIC	logLik	deviance	Chisq	Chi Df	Pr(>Chisq)
m.main	11	193852.00	193963.70	-96915.00	193830.00			
big1	12	189187.33	189308.94	-94581.67	189163.33	4666.67	1	< 0.0001
big2	13	188780.90	188912.64	-94377.45	188754.90	408.43	1	< 0.0001
big25	16	188249.06	188411.15	-94108.53	188217.06	537.84	3	< 0.0001
big3	17	178772.34	178943.74	-89369.17	178738.34	9478.71	1	< 0.0001

Table 29: Model comparison for models with different variables (data from waves 3-6). m.main = main model, then adding unemployment (big1), sex (big2), the importance of family (big25) and whether people can be trusted (big3)

6 Without random slopes

	Estimate	Std. Error	z value	Pr(>|z|)
(Intercept)	-1.33	0.19	-7.03	< 0.00001
FTR weak	0.05	0.21	0.26	0.79666

Table 30: Model without random slope for FTR by country (data from waves 3-6):
saveYes ˜FTR + (1 | country) + (1 + FTR | Autotyp.area) + (1 + FTR | family)
(AIC = 193867.67, BIC = 193959.06)

	Df	AIC	BIC	logLik	deviance	Chisq	Chi Df	Pr(>Chisq)
noCountrySlope	9	193867.67	193959.06	-96924.84	193849.67			
m.main	11	193852.00	193963.70	-96915.00	193830.00	19.67	2	0.0001

Table 31: Model comparison between main model and model without random slope for FTR by country (data from wave 6)

	Estimate	Std. Error	z value	Pr(>|z|)
(Intercept)	-1.22	0.16	-7.62	< 0.00001
FTR weak	0.28	0.19	1.42	0.15674

Table 32: Model without random slope for FTR by area (data from waves 3-6):
saveYes ˜FTR + (1 + FTR | country) + (1 | Autotyp.area) + (1 + FTR | family)
(AIC = 193850.59, BIC = 193941.98)

	Df	AIC	BIC	logLik	deviance	Chisq	Chi Df	Pr(>Chisq)
noAreaSlope	9	193850.59	193941.98	-96916.30	193832.59			
m.main	11	193852.00	193963.70	-96915.00	193830.00	2.59	2	0.2744

Table 33: Model comparison between main model and model without random slope for FTR by area (data from waves 3-6)

	Estimate	Std. Error	z value	Pr(>|z|)
(Intercept)	-1.19	0.25	-4.79	< 0.00001
FTR weak	0.30	0.37	0.80	0.42084

Table 34: Model without random slope for FTR by family (data from waves 3-6):
saveYes ˜FTR + (1 + FTR | country) + (1 + FTR | Autotyp.area) + (1 | family)
(AIC = 193851.96, BIC = 193943.35)

	Df	AIC	BIC	logLik	deviance	Chisq	Chi Df	Pr(>Chisq)
noFamilySlope	9	193851.96	193943.35	-96916.98	193833.96			
m.main	11	193852.00	193963.70	-96915.00	193830.00	3.96	2	0.1382

Table 35: Model comparison between main model and model without random slope for FTR by family (data from waves 3-6)

6.1 Summary

The comparisons above suggest that random slopes are only warranted for country. Below is a full model exploration with random slopes only for country.

	Estimate	Std. Error	z value	Pr(>|z|)
(Intercept)	-1.17	0.13	-8.77	< 0.00001
FTR weak	0.50	0.13	3.80	0.00014

Table 36: Model with random slope by country only: Main model
saveYes ˜FTR + (1 + FTR | country) + (1 | Autotyp.area) + (1 | family)
(AIC = 193851.81, BIC = 193922.89)

	Estimate	Std. Error	z value	Pr(>|z|)
(Intercept)	-1.32	0.14	-9.28	< 0.00001

Table 37: Model with random slope by country only: Null model.
saveYes ˜1 + (1 + FTR | country) + (1 | Autotyp.area) + (1 | family)
(AIC = 193859.33, BIC = 193920.25)

	Df	AIC	BIC	logLik	deviance	Chisq	Chi Df	Pr(>Chisq)
m2	6	193859.33	193920.25	-96923.66	193847.33			
m1	7	193851.81	193922.89	-96918.90	193837.81	9.52	1	0.0020

Table 38: Model with random slope by country only: Model comparison between main and null model.

7 Without random effects

	Estimate	Std. Error	z value	Pr(>|z|)
(Intercept)	-1.09	0.25	-4.34	0.00001
FTR weak	0.28	0.40	0.69	0.48988

Table 39: Model without random effect for country (data from waves 3-6):
saveYes ˜FTR + (1 + FTR | Autotyp.area) + (1 + FTR | family)
(AIC = 200693.4, BIC = 200774.63)

	Df	AIC	BIC	logLik	deviance	Chisq	Chi Df	Pr(>Chisq)
noCountry	8	200693.40	200774.63	-100338.70	200677.40			
m.main	11	193852.00	193963.70	-96915.00	193830.00	6847.39	3	< 0.0001

Table 40: Model comparison between main model and model without random effect for country.

	Estimate	Std. Error	z value	Pr(>|z|)
(Intercept)	-1.10	0.13	-8.40	< 0.00001
FTR weak	0.29	0.18	1.63	0.10329

Table 41: Model without random effect for area (data from waves 3-6):
saveYes ˜FTR + (1 + FTR | country) + (1 + FTR | family)
(AIC = 193857.86, BIC = 193939.09)

	Df	AIC	BIC	logLik	deviance	Chisq	Chi Df	Pr(>Chisq)
noArea	8	193857.86	193939.09	-96920.93	193841.86			
m.main	11	193852.00	193963.70	-96915.00	193830.00	11.85	3	0.0079

Table 42: Model comparison between main model and model without random effect for area.

	Estimate	Std. Error	z value	Pr(>|z|)
(Intercept)	-1.31	0.17	-7.84	< 0.00001
FTR weak	0.29	0.19	1.53	0.12661

Table 43: Model without random effect for family (data from waves 3-6):
saveYes ˜FTR + (1 + FTR | country) + (1 + FTR | Autotyp.area)
(AIC = 193875.2, BIC = 193956.43)

	Df	AIC	BIC	logLik	deviance	Chisq	Chi Df	Pr(>Chisq)
noFamily	8	193875.20	193956.43	-96929.60	193859.20			
m.main	11	193852.00	193963.70	-96915.00	193830.00	29.20	3	< 0.0001

Table 44: Model comparison between main model and model without random effect for family.

8 With random intercept for year

	Estimate	Std. Error	z value	Pr(>$\|z\|$)
(Intercept)	-1.26	0.19	-6.61	< 0.00001
FTR weak	0.21	0.25	0.86	0.38795

Table 45: Model including random intercept by year, data from waves 3-6: Main model
saveYes ˜FTR + (1 + FTR | country) + (1 + FTR | Autotyp.area) + (1 + FTR | family) + (1 | year)
(AIC = 193406.28, BIC = 193528.14)

	Estimate	Std. Error	z value	Pr(>$\|z\|$)
(Intercept)	-1.15	0.23	-4.90	< 0.00001

Table 46: Model including random intercept by year, data from waves 3-6: Null model.
saveYes ˜1 + (1 + FTR | Autotyp.area) + (1 + FTR | family) + (1 | year)
(AIC = 199008.02, BIC = 199089.25)

	Df	AIC	BIC	logLik	deviance	Chisq	Chi Df	Pr(>Chisq)
m2	8	199008.02	199089.25	-99496.01	198992.02			
m1	12	193406.28	193528.14	-96691.14	193382.28	5609.74	4	< 0.0001

Table 47: Model including random intercept by year, data from waves 3-6: Model comparison between main and null model.

	Df	AIC	BIC	logLik	deviance	Chisq	Chi Df	Pr(>Chisq)
m.main	11	193852.00	193963.70	-96915.00	193830.00			
m.year	12	193406.28	193528.14	-96691.14	193382.28	447.72	1	< 0.0001

Table 48: Model comparison between main model and model with random intercept by year

9 With random intercept for language

	Estimate	Std. Error	z value	Pr(>|z|)
(Intercept)	-1.16	0.15	-7.61	< 0.00001
FTR weak	0.42	0.26	1.59	0.11112

Table 49: Model including random intercept by language, data from waves 3-6: Main model
saveYes ˜FTR + (1 + FTR | country) + (1 + FTR | Autotyp.area) + (1 + FTR | family) + (1 | lang)
(AIC = 193503.36, BIC = 193625.21)

	Estimate	Std. Error	z value	Pr(>|z|)
(Intercept)	-1.11	0.21	-5.32	< 0.00001

Table 50: Model including random intercept by language, data from waves 3-6: Null model.
saveYes ˜1 + (1 + FTR | Autotyp.area) + (1 + FTR | family) + (1 | lang)
(AIC = 195308.33, BIC = 195389.57)

	Df	AIC	BIC	logLik	deviance	Chisq	Chi Df	Pr(>Chisq)
m2	8	195308.33	195389.57	-97646.17	195292.33			
m1	12	193503.36	193625.21	-96739.68	193479.36	1812.97	4	< 0.0001

Table 51: Model including random intercept by language, data from waves 3-6: Model comparison between main and null model.

	Df	AIC	BIC	logLik	deviance	Chisq	Chi Df	Pr(>Chisq)
m.main	11	193852.00	193963.70	-96915.00	193830.00			
m.lang	12	193503.36	193625.21	-96739.68	193479.36	350.64	1	< 0.0001

Table 52: Model comparison between main model and model with random intercept by language

10 With random intercept for language and year

	Estimate	Std. Error	z value	Pr(>|z|)
(Intercept)	-1.20	0.17	-7.00	< 0.00001
FTR weak	0.40	0.28	1.41	0.15918

Table 53: Model including random intercept by language and year, data from waves 3-6: Main model
saveYes ˜FTR + (1 + FTR | country) + (1 + FTR | Autotyp.area) + (1 + FTR | family) + (1 | lang) + (1 | year)
(AIC = 193038.01, BIC = 193170.02)

	Estimate	Std. Error	z value	Pr(>|z|)
(Intercept)	-1.35	0.15	-9.05	< 0.00001

Table 54: Model including random intercept by language and year, data from waves 3-6: Null model.
saveYes ˜1 + (1 + FTR | country) + (1 + FTR | Autotyp.area) + (1 + FTR | family) + (1 | lang) + (1 | year)
(AIC = 193039.04, BIC = 193160.89)

	Df	AIC	BIC	logLik	deviance	Chisq	Chi Df	Pr(>Chisq)
m2	12	193039.04	193160.89	-96507.52	193015.04			
m1	13	193038.01	193170.02	-96506.01	193012.01	3.03	1	0.0817

Table 55: Model including random intercept by language and year, data from waves 3-6: Model comparison between main and null model.

	Df	AIC	BIC	logLik	deviance	Chisq	Chi Df	Pr(>Chisq)
m.main	11	193852.00	193963.70	-96915.00	193830.00			
m.lang	12	193503.36	193625.21	-96739.68	193479.36	350.64	1	< 0.0001
m.langAndYear	13	193038.01	193170.02	-96506.01	193012.01	467.35	1	< 0.0001

Table 56: Model comparison between main model and model with random intercept by language

11 Model with continent instead of Autotyp area

	Estimate	Std. Error	z value	Pr(>|z|)
(Intercept)	-1.22	0.23	-5.36	< 0.00001
FTR weak	0.23	0.31	0.74	0.45942

Table 57: Model including random effect for continent instead of Autotyp area, data from waves 3-6: Main model
saveYes ˜FTR + (1 + FTR | country) + (1 + FTR | continent) + (1 + FTR | family)
(AIC = 193867.31, BIC = 193979)

	Estimate	Std. Error	z value	Pr(>|z|)
(Intercept)	-1.29	0.23	-5.68	< 0.00001

Table 58: Model including random effect for continent instead of Autotyp area, data from waves 3-6: Null model.
saveYes ˜1 + (1 + FTR | country) + (1 + FTR | continent) + (1 + FTR | family)
(AIC = 193866.42, BIC = 193967.96)

	Df	AIC	BIC	logLik	deviance	Chisq	Chi Df	Pr(>Chisq)
m2	10	193866.42	193967.96	-96923.21	193846.42			
m1	11	193867.31	193979.00	-96922.65	193845.31	1.11	1	0.2922

Table 59: Model including random effect for continent instead of Autotyp area, data from waves 3-6: Model comparison between main and null model.

12 Language genus instead of language family

	Estimate	Std. Error	z value	Pr(>|z|)
(Intercept)	-1.22	0.23	-5.36	< 0.00001
FTR weak	0.23	0.31	0.74	0.45942

Table 60: Model including random effect for language genus instead of language family (data from waves 3-6, models did not converge after 500,000 function evaluations) Main model
saveYes ˜FTR + (1 + FTR | country) + (1 + FTR | continent) + (1 + FTR | family)
(AIC = 193867.31, BIC = 193979)

	Estimate	Std. Error	z value	Pr(>|z|)
(Intercept)	-1.29	0.23	-5.68	< 0.00001

Table 61: Model including random effect for language genus instead of language family (data from waves 3-6, models did not converge after 500,000 function evaluations) Null model.
saveYes ˜1 + (1 + FTR | country) + (1 + FTR | continent) + (1 + FTR | family)
(AIC = 193866.42, BIC = 193967.96)

	Df	AIC	BIC	logLik	deviance	Chisq	Chi Df	Pr(>Chisq)
m2	10	193866.42	193967.96	-96923.21	193846.42			
m1	11	193867.31	193979.00	-96922.65	193845.31	1.11	1	0.2922

Table 62: Model including random effect for language genus instead of language family (data from waves 3-6, models did not converge after 500,000 function evaluations) Model comparison between main and null model.

13 Without immigrants

	Estimate	Std. Error	z value	Pr(>|z|)
(Intercept)	-1.25	0.19	-6.61	< 0.00001
FTR weak	0.28	0.24	1.16	0.24658

Table 63: Model excluding respondents whose mother or father were immigrants, data from waves 3-6: Main model saveYes ˜FTR + (1 + FTR | country) + (1 + FTR | Autotyp.area) + (1 + FTR | family)
(AIC = 182047.71, BIC = 182158.8)

	Estimate	Std. Error	z value	Pr(>|z|)
(Intercept)	-1.32	0.19	-7.05	< 0.00001

Table 64: Model excluding respondents whose mother or father were immigrants, data from waves 3-6: Null model. saveYes ˜1 + (1 + FTR | country) + (1 + FTR | Autotyp.area) + (1 + FTR | family)
(AIC = 182047.65, BIC = 182148.64)

	Df	AIC	BIC	logLik	deviance	Chisq	Chi Df	Pr(>Chisq)
m2	10	182047.65	182148.64	-91013.83	182027.65			
m1	11	182047.71	182158.80	-91012.85	182025.71	1.94	1	0.1635

Table 65: Model excluding respondents whose mother or father were immigrants, data from waves 3-6: Model comparison between main and null model.

<table>
<tr><th>form</th><th>aic</th><th>sig</th></tr>
<tr><td>saveYes ˜ FTR + unem + sex2 + famImp2 + trustYes + (1 + FTR |country) + (1 + FTR|Autotyp.area) + (1 + FTR|family)</td><td>178772.34</td><td></td></tr>
<tr><td>saveYes ˜ 1 + (1 + FTR |country) + (1 + FTR|Autotyp.area) + (1 + FTR|family)</td><td>182047.65</td><td>N/A</td></tr>
<tr><td>saveYes ˜ FTR + (1 + FTR |country) + (1 + FTR|Autotyp.area) + (1 + FTR|family)</td><td>182047.71</td><td></td></tr>
<tr><td>saveYes ˜ trustYes + (1 + trustYes |country) + (1 + trustYes|Autotyp.area) + (1 + trustYes|family)</td><td>183958.81</td><td></td></tr>
<tr><td>saveYes ˜ 1 + (1 + trustYes |country) + (1 + trustYes|Autotyp.area) + (1 + trustYes|family)</td><td>183964.41</td><td>N/A</td></tr>
<tr><td>saveYes ˜ FTR + unem + sex2 + famImp2 + (1 + FTR |country) + (1 + FTR|Autotyp.area) + (1 + FTR|family)</td><td>188249.06</td><td></td></tr>
<tr><td>saveYes ˜ FTR + unem + sex2 + (1 + FTR |country) + (1 + FTR|Autotyp.area) + (1 + FTR|family)</td><td>188780.9</td><td></td></tr>
<tr><td>saveYes ˜ 1 + (1 + unem |country) + (1 + unem|Autotyp.area) + (1 + unem|family)</td><td>189086.92</td><td>N/A</td></tr>
<tr><td>saveYes ˜ unem + (1 + unem |country) + (1 + unem|Autotyp.area) + (1 + unem|family)</td><td>189089.34</td><td>*</td></tr>
<tr><td>saveYes ˜ FTR + unem + (1 + FTR |country) + (1 + FTR|Autotyp.area) + (1 + FTR|family)</td><td>189187.33</td><td></td></tr>
<tr><td>saveYes ˜ FTR + (1 + FTR |country) + (1 + FTR|Autotyp.area) + (1 + FTR|family) + (1|lang) + (1|year)</td><td>193038.01</td><td></td></tr>
<tr><td>saveYes ˜ 1 + (1 + FTR |country) + (1 + FTR|Autotyp.area) + (1 + FTR|family) + (1|lang) + (1|year)</td><td>193039.04</td><td>N/A</td></tr>
<tr><td>saveYes ˜ FTR + (1 + FTR |country) + (1 + FTR|Autotyp.area) + (1 + FTR|family) + (1|year)</td><td>193406.28</td><td></td></tr>
<tr><td>saveYes ˜ FTR + (1 + FTR |country) + (1 + FTR|Autotyp.area) + (1 + FTR|family) + (1|lang)</td><td>193503.36</td><td></td></tr>
<tr><td>saveYes ˜ 1 + (1 + sex2 |country) + (1 + sex2|Autotyp.area) + (1 + sex2|family)</td><td>193518.76</td><td>N/A</td></tr>
<tr><td>saveYes ˜ sex2 + (1 + sex2 |country) + (1 + sex2|Autotyp.area) + (1 + sex2|family)</td><td>193538.02</td><td></td></tr>
<tr><td>saveYes ˜ FTR + (1 + FTR |country) + (1|Autotyp.area) + (1 + FTR|family)</td><td>193850.59</td><td></td></tr>
<tr><td>saveYes ˜ FTR + (1 + FTR |country) + (1|Autotyp.area) + (1|family)</td><td>193851.81</td><td>*</td></tr>
<tr><td>saveYes ˜ FTR + (1 + FTR |country) + (1 + FTR|Autotyp.area) + (1|family)</td><td>193851.96</td><td></td></tr>
<tr><td>saveYes ˜ FTR + (1 + FTR |country) + (1 + FTR|Autotyp.area) + (1 + FTR|family)</td><td>193852</td><td></td></tr>
<tr><td>saveYes ˜ 1 + (1 + FTR |country) + (1 + FTR|Autotyp.area) + (1 + FTR|family)</td><td>193852.33</td><td>N/A</td></tr>
<tr><td>saveYes ˜ FTR + (1 + FTR |country) + (1 + FTR|family)</td><td>193857.86</td><td></td></tr>
<tr><td>saveYes ˜ 1 + (1 + FTR |country) + (1|Autotyp.area) + (1|family)</td><td>193859.33</td><td>N/A</td></tr>
<tr><td>saveYes ˜ 1 + (1 + FTR |country) + (1 + FTR|continent) + (1 + FTR|family)</td><td>193866.42</td><td>N/A</td></tr>
<tr><td>saveYes ˜ 1 + (1 + FTR |country) + (1 + FTR|continent) + (1 + FTR|family)</td><td>193866.42</td><td>N/A</td></tr>
<tr><td>saveYes ˜ FTR + (1 + FTR |country) + (1 + FTR|continent) + (1 + FTR|family)</td><td>193867.31</td><td></td></tr>
<tr><td>saveYes ˜ FTR + (1 + FTR |country) + (1 + FTR|continent) + (1 + FTR|family)</td><td>193867.31</td><td></td></tr>
<tr><td>saveYes ˜ FTR + (1 |country) + (1 + FTR|Autotyp.area) + (1 + FTR|family)</td><td>193867.67</td><td></td></tr>
<tr><td>saveYes ˜ FTR + (1 + FTR |country) + (1 + FTR|Autotyp.area)</td><td>193875.2</td><td></td></tr>
<tr><td>saveYes ˜ 1 + (1 + FTR |Autotyp.area) + (1 + FTR|family) + (1|lang)</td><td>195308.33</td><td>N/A</td></tr>
<tr><td>saveYes ˜ 1 + (1 + FTR |Autotyp.area) + (1 + FTR|family) + (1|year)</td><td>199008.02</td><td>N/A</td></tr>
<tr><td>saveYes ˜ FTR + (1 + FTR |Autotyp.area) + (1 + FTR|family)</td><td>200693.4</td><td></td></tr>
</table>

Table 66: Summary of models for data from waves 3-6, sorted by AIC. The third column indicates whether the coefficient for the first fixed effect within the model is significant (though these estimates are unreliable).
