# Supplementary material for: Future Tense and Economic Decisions: Controlling for Cultural Evolution
Source: PLoS One. 2015 Jul 17;10(7):e0132145. doi: 10.1371/journal.pone.0132145 (PMC4506144; doi:10.1371/journal.pone.0132145)
Supplement: S3 Appendix — (PDF) [file pone.0132145.s003.pdf]

Future Tense and economic savings: Convergence problems in fixed effect probability estimates.

Seán Roberts

1 Introduction

Roberts, Winters & Chen use mixed effects modelling with a logistic link function (using *lme4* version 1.1-7 in R). In the course of running the models, we noticed that the estimates of significance for fixed effects change depending on the order of the rows in the dataset.

That is, if I run a model on a dataset, I get certain estimate for my fixed effect and it has a certain p-value. I run the model again, and I get the same estimate and p-value. Now, I shuffle the order of rows (the data is not mixed, just the rows are in a different order). Running the model a third time, the p-value is very different.

For the data from the paper, the estimated p-value for the FTR fixed effect can be between p=0.01 and p=0.04. Obviously, these are crucial differences given conventional significance levels.

A certain amount of difference between runs is expected. The p-value estimates are just estimated, and there will be differences between values for a number of reasons to do with the optimiser algorithm. However, the magnitude of the differences in the current study are large, and the order of the data frame is an unexpected source of this difference.

The difference appears to be in the standard errors, while the differences in coefficient estimates are much smaller. The model fit (AIC etc.) are the same, suggesting there are multiple optimal convergences, and the order of the data pushes the optimiser into different ones. However, there are slightly different estimates every time I shuffle the data frame (not just two or three unique estimates). In some cases, the model did not converge simply because of a shuffling of the rows.

The magnitude of the problem in this study is likely to lie partially with the structure of my particular data. It's reasonably large (nearly 200,000 cases), and has nested random effects. We tried centering the data, using contrast coding and feeding starting values to lmer based on a previous fit. This did not significantly

change the results. We also tried using different ways of calculating p-values, but this made no difference.

Below, we run the model multiple times with different row orders, and report the range of p-values. However, this seems inelegant and potentially quite confusing.

For the purposes of this study, the best solution is to focus on the model comparison tests rather than the p-values of the fixed effect within the main model. The problem above does not affect model comparison estimates (using anova), since these are based on differences in model fit. Also, The fixed effect coefficient estimates are also reasonably robust. Therefore, in the main paper, we report the fixed effect coefficient estimate and the p-value from a model comparison with a null model, rather than the p-values from within the main model.

This issue is being pursued by Ben Bolker and others on the lme4 github page `https://github.com/lme4/lme4/issues/262`. See also CrossValidated post: `http://tinyurl.com/q63acrz`.

We note that the problem does not appear to be present when using Bayesian linear mixed effects modelling (in R package *blame*).

2 Distribution of significance estimates

In this section, we present the results of running the main model in the paper multiple times, with each run having the order of rows in the data frame shuffled.

The model was run 70 for each of the datasets (waves 1-5 and waves 1-6). Table 1 and 2 show how model parameters vary when permuting row order.

	Min	Max	Mean	SD	SD.proportion
Intercept Estimate	-1.24e+00	-1.24e+00	-1.24e+00	3.27e-05	2.6e-05
Intercept Std. Error	1.47e-01	1.68e-01	1.55e-01	4.84e-03	0.031
Intercept z-value	-8.47e+00	-7.40e+00	-8.05e+00	2.47e-01	0.031
Intercept p-value	2.55e-17	1.36e-13	6.98e-15	2.06e-14	3
FTR Estimate	4.12e-01	4.12e-01	4.12e-01	4.47e-05	0.00011
FTR Std. Error	1.66e-01	1.97e-01	1.81e-01	6.69e-03	0.037
FTR z-value	2.10e+00	2.49e+00	2.28e+00	8.36e-02	0.037
FTR p-value	0.0129	0.0361	2.30e-02	5.11e-03	0.22
AIC	1.41e+05	1.41e+05	1.41e+05	0.00e+00	0
BIC	1.41e+05	1.41e+05	1.41e+05	0.00e+00	0
Log likelihood	-7.06e+04	-7.06e+04	-7.06e+04	1.68e-06	2.4e-11
Deviance	1.41e+05	1.41e+05	1.41e+05	0.00e+00	0
DF resid.	1.43e+05	1.43e+05	1.43e+05	0.00e+00	0

Table 1: Distribution of model estimates and p-values for data from waves 1-5. The last column is the Standard deviation as a proportion of the mean.

	Min	Max	Mean	SD	SD.proportion
Intercept Estimate	-1.24e+00	-1.24e+00	-1.24e+00	4.66e-05	3.8e-05
Intercept Std. Error	1.21e-01	1.51e-01	1.35e-01	7.27e-03	0.054
Intercept z-value	-1.03e+01	-8.22e+00	-9.17e+00	4.97e-01	0.054
Intercept p-value	1.17e-24	2.08e-16	7.46e-18	3.16e-17	4.2
FTR Estimate	2.57e-01	2.58e-01	2.58e-01	7.30e-05	0.00028
FTR Std. Error	0.148	0.221	1.75e-01	1.60e-02	0.091
FTR z-value	1.17e+00	1.74e+00	1.48e+00	1.32e-01	0.089
FTR p-value	0.08	0.24	1.42e-01	3.59e-02	0.25
AIC	1.94e+05	1.94e+05	1.94e+05	0.00e+00	0
BIC	1.94e+05	1.94e+05	1.94e+05	0.00e+00	0
Log likelihood	-9.69e+04	-9.69e+04	-9.69e+04	0.00e+00	0
Deviance	1.94e+05	1.94e+05	1.94e+05	0.00e+00	0
DF resid.	1.90e+05	1.90e+05	1.90e+05	0.00e+00	0

Table 2: Distribution of model estimates and p-values for data from waves 1-6. The last column is the Standard deviation as a proportion of the mean.

3 Reproducible example with synthesised data

```
library(lme4)
set.seed(999)

# make a somewhat complex data frame
x = c(rnorm(10000),rnorm(10000,0.1))
x = sample(x)
y = jitter(x,amount=10)
a = rep(1:20,length.out=length(x))
y[a==1] = jitter(y[a==1],amount=3)
y[a==2] = jitter(x[a==2],amount=1)
y[a>3 & a<6] = rnorm(sum(a>3 & a<6))
# convert to binary variables
y = y >0
x = x >0
# make a data frame
d = data.frame(x1=x,y1=y,a1=a)

# run model
m1 = glmer(x1~y1+(1+y1|a1),data=d,family=binomial(link='logit'))

# shuffle order of rows
d = d[sample(nrow(d)),]

# run model again
m2 = glmer(x1~y1+(1+y1|a1),data=d,family=binomial(link='logit'))

# show output
summary(m1)
summary(m2)
```
