# Supplementary figures and images for: Future Tense and Economic Decisions: Controlling for Cultural Evolution
Source: PLoS One. 2015 Jul 17;10(7):e0132145. doi: 10.1371/journal.pone.0132145 (PMC4506144; doi:10.1371/journal.pone.0132145)

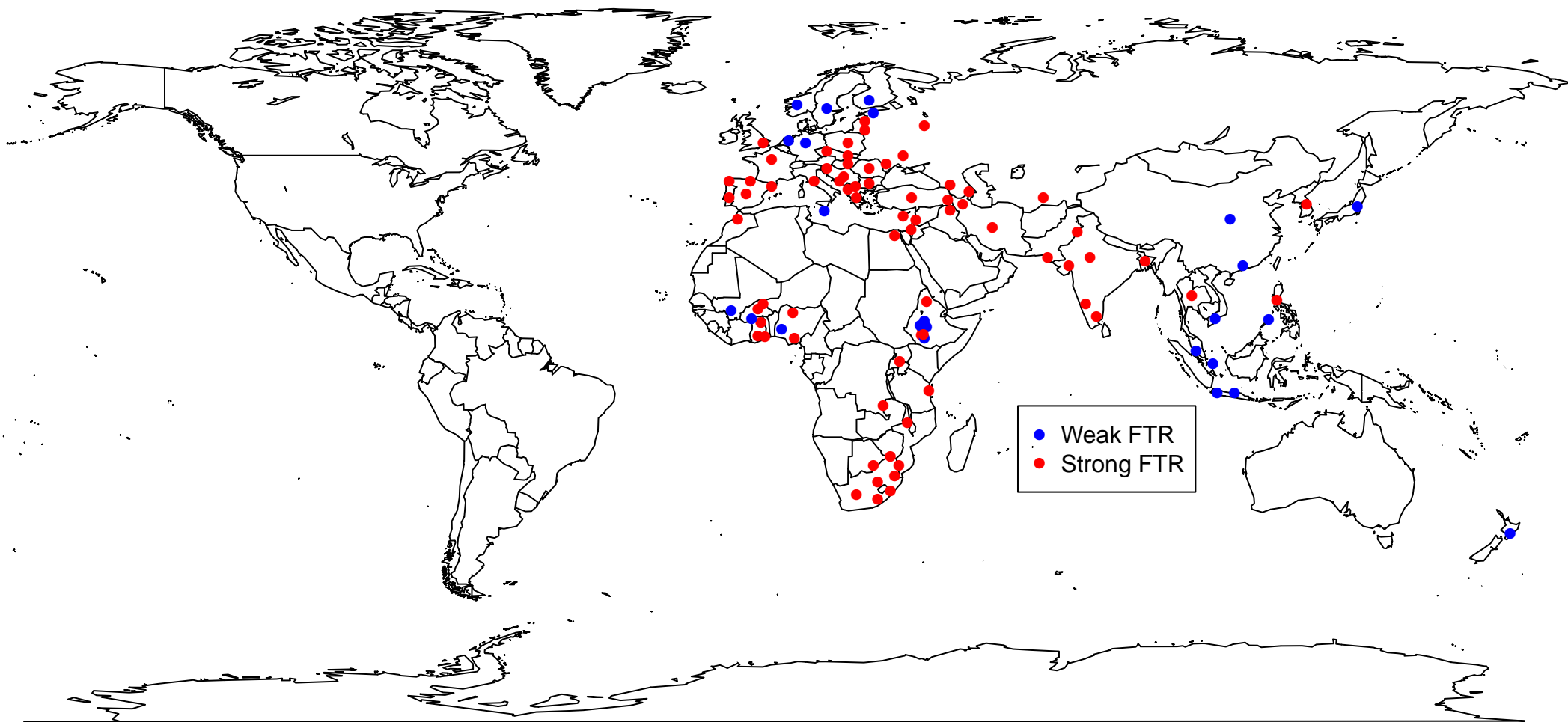

Supplement: S8 Appendix — See README files within the various sub-folders. (ZIP) [file pone.0132145.s008.zip › S3_Code/Residuals_new/GeoDist/Map_FTR.pdf]

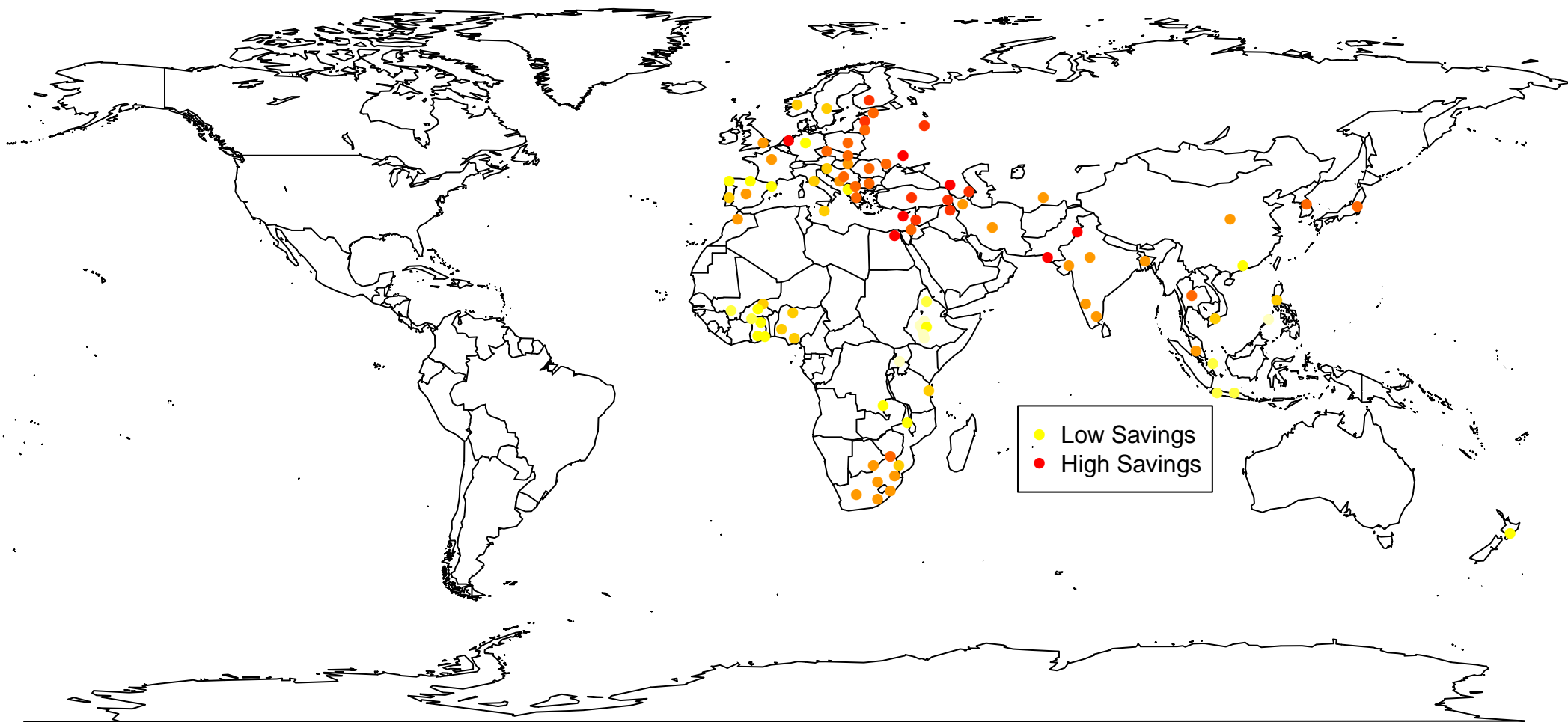

Supplement: S8 Appendix — See README files within the various sub-folders. (ZIP) [file pone.0132145.s008.zip › S3_Code/Residuals_new/GeoDist/Map_FTR_Resid.pdf]

$\rho = 0.1$

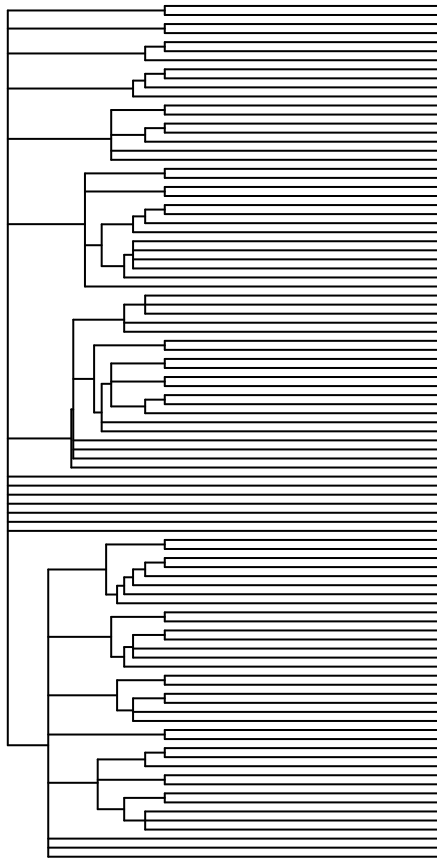

$\rho = 1$

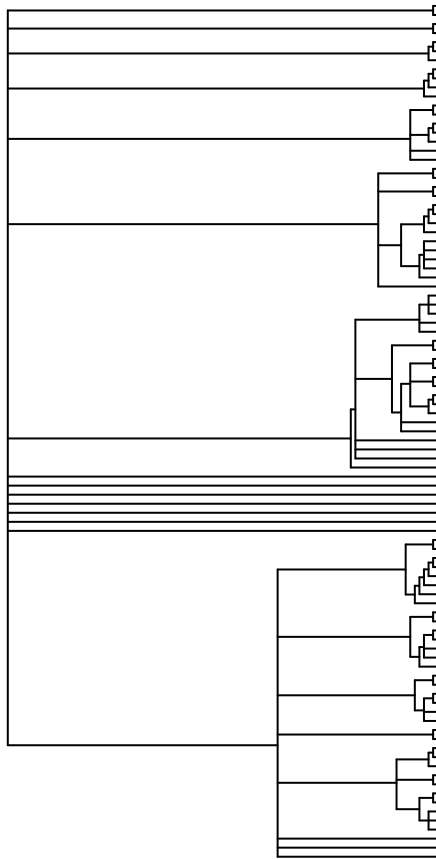

$\rho = 2$

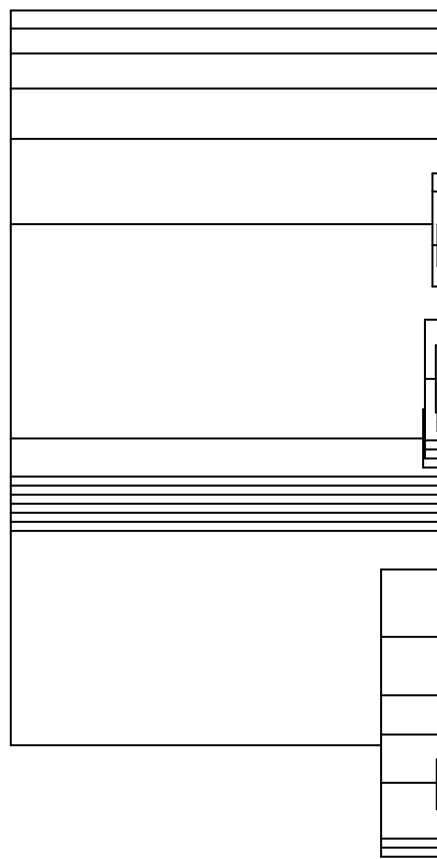

Supplement: S8 Appendix — See README files within the various sub-folders. (ZIP) [file pone.0132145.s008.zip › S3_Code/Residuals_new/PGLS/BranchDepthManip_Graphs.pdf]

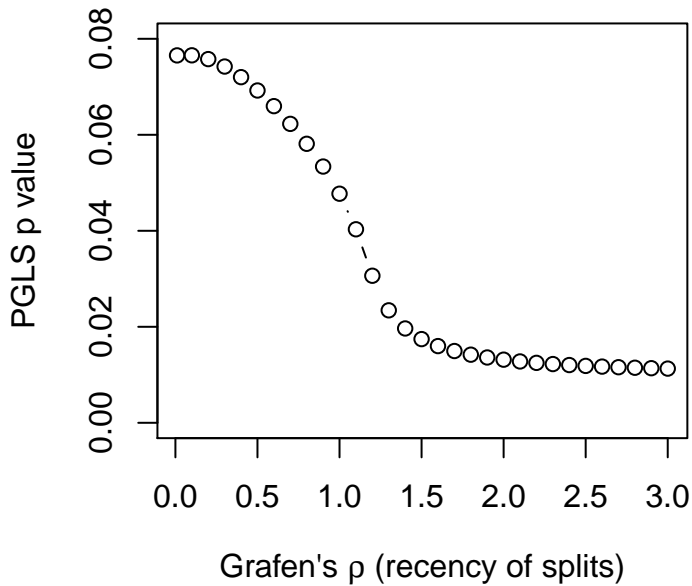

Supplement: S8 Appendix — See README files within the various sub-folders. (ZIP) [file pone.0132145.s008.zip › S3_Code/Residuals_new/PGLS/BranchDepthManip_PGLS.pdf]

**Between = 40,000, Within = 3,000**

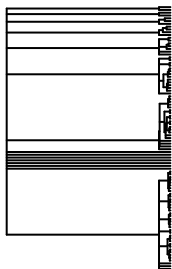

**Between = 80,000, Within = 3,000**

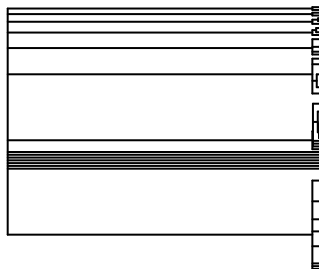

**Between = 40,000, Within = 12,000**

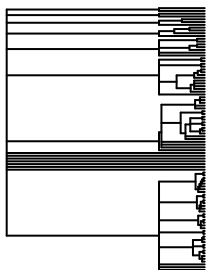

**Between = 80,000, Within = 12,000**

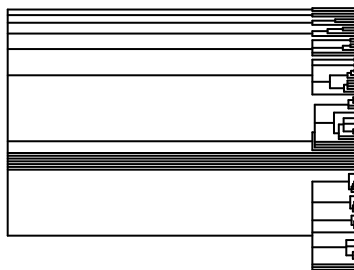

Supplement: S8 Appendix — See README files within the various sub-folders. (ZIP) [file pone.0132145.s008.zip › S3_Code/Residuals_new/PGLS/BranchLenghtManip_Graphs.pdf]

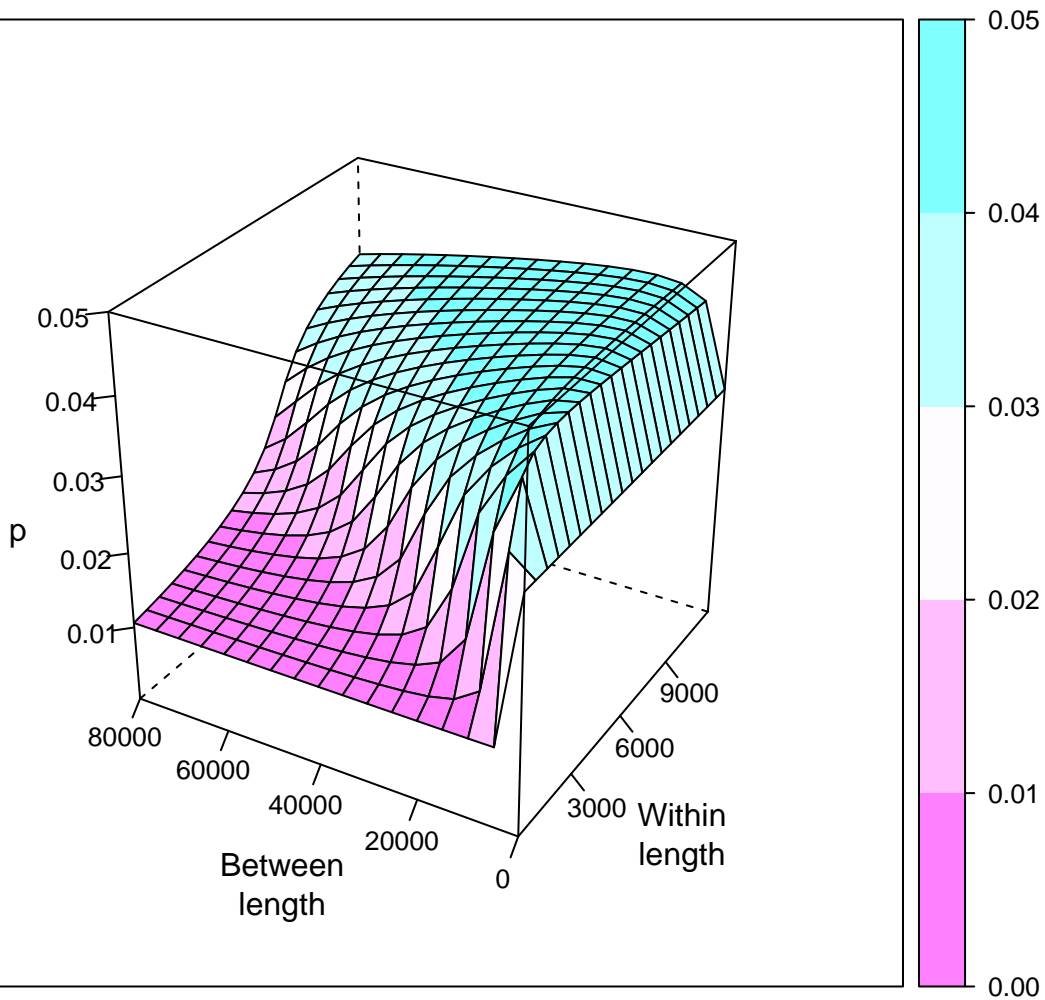

Supplement: S8 Appendix — See README files within the various sub-folders. (ZIP) [file pone.0132145.s008.zip › S3_Code/Residuals_new/PGLS/BranchLengthManip_PGLS.pdf]
