# Supplementary material for: Future Tense and Economic Decisions: Controlling for Cultural Evolution
Source: PLoS One. 2015 Jul 17;10(7):e0132145. doi: 10.1371/journal.pone.0132145 (PMC4506144; doi:10.1371/journal.pone.0132145)
Supplement: S9 Appendix — Notes on the various variables in the main data file, and how they were calculated. (PDF) [file pone.0132145.s009.pdf]

Linking the World Values Survey to the World Atlas of Language Structures

Seán Roberts

1 Sources

The following data sources were used:
KC: Keith Chen's data on the FTR variable (Chen, 2013)
WVS: The World Values Survey (Five wave aggregated file 1981-2005) (World Values Survey Association, 2009)
WALS: The World Atlas of Language Structures (Haspelmath et al., 2008)
Ethnologue: The Ethnologue (Gordon, 2005)
I also include data from Harald Hammarström's PhD thesis.

2 File *WW_Selections2*

3 File *Futureness_Plus_WALS_Cat.tab*

This file links the languages described in the WVS to data from WALS, Ethnologue and KC. These only include waves 1-5.

First, I went over the list of languages Prof. Chen gave me and matched them up with the correct language identification code in WALS and the Ethnologue. This wasn't entirely straightforward, since languages have many alternative names (e.g. "Bamanakan" is also known as "Bambara"). When there was not an immediate match in WALS, the alternative names were checked in the Ethnologue. For languages with alternative names, I checked that it made sense given the country in which the respondent completed the WVS. Not all languages in the WVS could be linked with data from WALS, in some cases because the data was not available, and in others because it was not clear what language was being referred to in WVS.

Another problem is that the languages listed in the World values survey split and lump languages differently to WALS. For example, 'Croatian' and 'Serbian'

are listed as different languages in WVS, but WALS includes them both under 'Serbian-Croatian' (Splitting). Similarlly, 'Seraiki' is considered a dialect of Panjabi (or Punjabi) in WALS. The converse problem is lumping: respondents who say they speak 'Arabic' may be describing one of several types of Arabic detailed in WALS.

When lumping occurs, I've made some distinctions based on the country that the respondent is answering the survey in (see the variable LangCountry). For example, respondents who say they speak Arabic from Egypt are coded as speaking Egyptian Arabic. Those who say they speak Arabic from Morocco are coded as speaking Moroccan Arabic. In more unclear situations, the population of speakers is taken into account. For example, the majority of 'Chinese' speakers in Malaysia will speak Mandarin, while the majority of 'Chinese' speakers in the USA will speak Cantonese. However, the situation in Australia is too close to call, so these are left uncoded.

Some additional problems occur with dialect chains, such as in Thailand where respondents answered "Thai:Northern" or "Thai: Southern", which don't easily fit with a WALS language.

Cases from the WVS that do not have a response to the 'Family savings' question, or cases that are not linked with a WALS code are removed.

3.1 Variable description

The last 192 variables come from WALS. The first 18 variables are the the following:

- *language*: The name of the language from KC
- *Lang_WVS*: The labelling of the language in the WVS
- *family*: Language family from KC
- *genus*: Language genus from KC
- *FTR*: Future tense marking from KC
- *WALS*: WALS identification code
- *ISO*: ISO code (Ethnologue)
- *latitude*: Latitude of the language locus according to WALS

- *longitude*: Longitude of the language locus according to WALS
- *LangCountry*: The country in which the WVS respondent carried out the survey. If there is a value here, it means that the corresponding language only applies to respondents from this country.
- *Notes*: Notes from SR on mapping between KC language and WALS code.
- *any_ftr*: From KC
- *prediction_ftr*: From KC
- *inflectional_ftr*: From KC
- *genus_wals*: Language genus from WALS
- *family_wals*: Language family from WALS
- *subfamily_wals*: Language sub-family from WALS
- *hh_family*: Language genus from Harald Hammarström.
- *wvs.case*: The case number of the WVS entry (assigned by me), could be used to link other variables from the WVS.

4 Raw data

Files ww_FiveWavesD.csv and WW_selections2_autotyp.csv contain raw data extracted from the world values survey combined with all the relevant data from the file above. ww_FiveWavesD.csv contain data from waves 1-5 only.

- *lang*: Language of respondent. Variable G016 from WVS
- *save*: Family savings during the past year, all values. Variable X044 from WVS
- *sex*: Sex of Respondent. Variable X001 from WVS
- *sex2*: Sex of Respondent, divided into Male/Female (the WVS only recognises the following categories: "Don't know", "Female', "Male', "Missing', "Unknown', "No answer"). Variable X001 from WVS
- *age*: Age of Respondent, integer. Variable X003 from WVS
- *edu*: Education level of respondent. Variable X025R from WVS

- *edu2*: Education level as a rank variable: Elementary, SecondaryPrep, SecondaryTech, StartedElemenary ,StartedSecondaryPrep, StartedSecondary-Tech, StartedUni, Uni .
- *unemployed*: Employment status of respondent, all. Variable X028 from WVS
- *married*: Marriage status of the respondent. Variable X007 from WVS
- *married2*: True if married.
- *numChildren*: The number of children the respondent has, categorical. Variable X011 from WVS
- *religion*: The religion of the respondent. VariableF025) from WVS
- *year*: Survey year. Variable S020 from WVS
- *country*: Survey country. Variable S003 from WVS
- *saveYes*: Did the respondent save money in the last year? "Save"=True; "Just get by", "Spent some savings and borrowed money" and "Spent savings and borrowed money" = False.
- *trustYes*: True if respondent responded "Most people can be trusted" to question "Most people can be trusted" (V23).
- *famImp2*: Respondent answer to " indicate how important it is in your life.". Ranked variable: Not at all important, Not very important, Rather important, Very important.
- *unem*: Is the respondent employed or retired.
- *ageCut*: Age cut into 10-year bins
- *numChildrenX*: The number of children the respondent has, integer.
- *family*: See section 3.
- *Genus*: See section 3.
- *genus_wals*: Genus according to WALS.
- *family_wals*: Language family according to WALS.
- *subfamily_wals*: Language sub-family according to WALS.

- *hh_family*: Language family according to Hammarström.
- *continent*: Continent that the language's language family originates in.
- *FTR*: See section 3.
- *wals*: See section 3.
- *iso*: See section 3.
- *lat*: See section 3.
- *long*: See section 3.
- *any_ftr*: See section 3.
- *prediction_ftr*: See section 3.
- *inflectional_ftr*: See section 3.
- *nResp*: Number of respondents for the corresponding language
- *pop*: Number of speakers of the corresponding language (world total, from Ethnologue)
- *lit*: Literacy of the corresponding country, percentage (from `http://en.wikipedia.org/wiki/List_of_countries_by_literacy_rate`)
- *lit.m*: Literacy of males in the corresponding country
- *lit.f*: Literacy of females in the corresponding counter.
- *MotImm*: Mother was an immigrant (V243)
- *FathImm*: Father was an immigrant (V244)
- *Autotyp.area*: Linguistic area according to the AutoTyp database (Nichols et al., 2013).

References

Chen, M. K. (2013). The effect of language on economic behavior: Evidence from savings rates, health behaviors, and retirement assets. *American Economic Review*, 103(2):690–731. [1]

Gordon, R. (2005). *Ethnologue: Languages of the World, 15th Edition.* SIL International. [1]

Haspelmath, M., Dryer, M. S., Gil, D., and Comrie, B. (2008). *World Atlas of Language Structures*, volume Available online at http://wals.info/feature/22 Accessed on 2013-04-18. Munich: Max Planck Digital Library. [1]

Nichols, J., Witzlack-Makarevich, A., and Bickel, B. (2013). The autotyp genealogy and geography database: 2013 release. [5]

World Values Survey Association (2009). World Values Survey 1981-2008 Official Aggregate v. 20090901. *Madrid: ASEP/JDS.* [1]
