# Supplementary material for: Future Tense and Economic Decisions: Controlling for Cultural Evolution
Source: PLoS One. 2015 Jul 17;10(7):e0132145. doi: 10.1371/journal.pone.0132145 (PMC4506144; doi:10.1371/journal.pone.0132145)
Supplement: S10 Appendix — For each country, a graph showing the proportion of speakers of each language saving money for strong and weak FTR. Circle size indicates the proportion of observations for a given language. Red lines indicate the overall mean for the FTR type. (PDF) [file pone.0132145.s010.pdf]

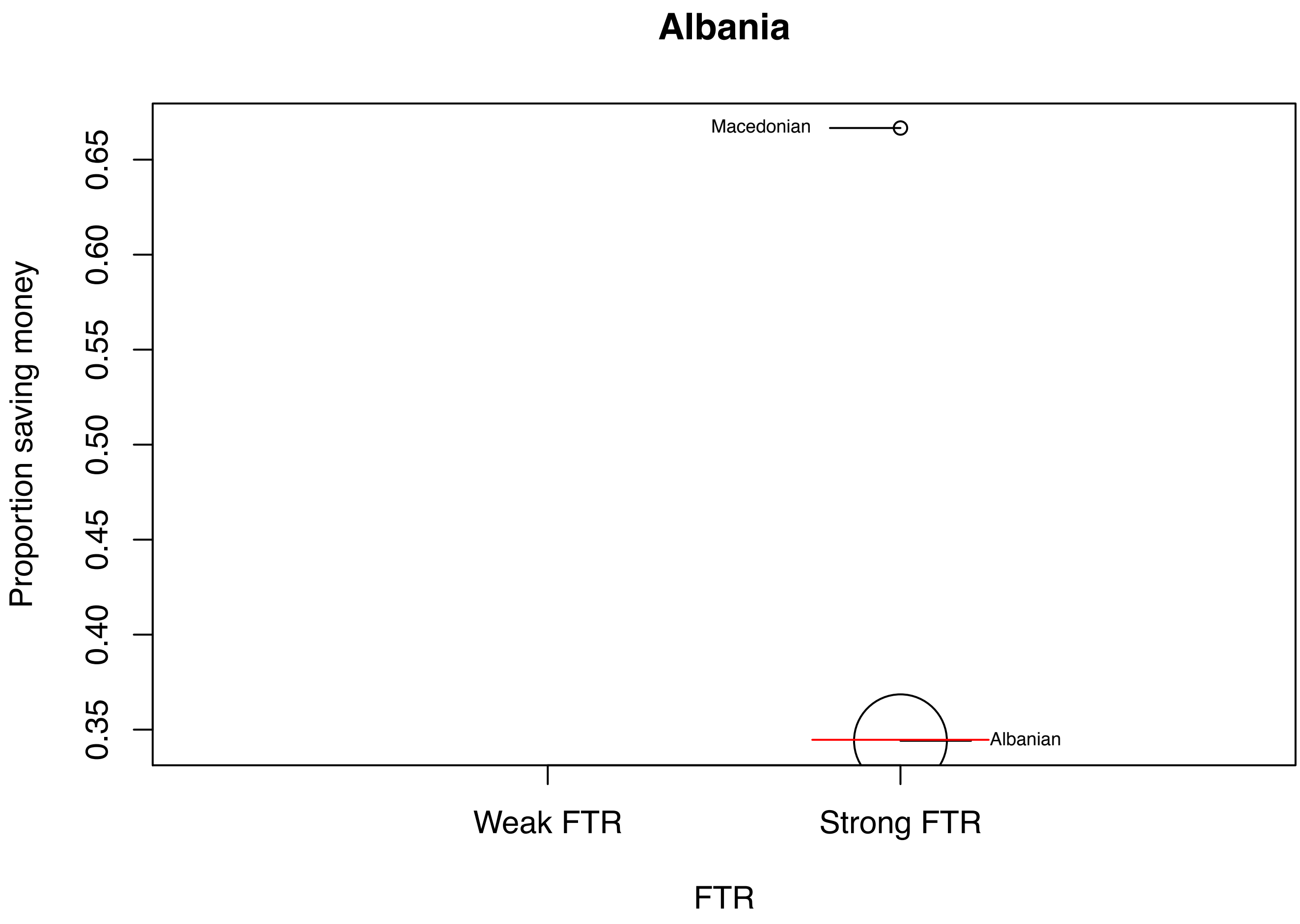
Albania
Macedonian
Albanian
Proportion saving money
0.35
0.40
0.45
0.50
0.55
0.60
0.65
Weak FTR
Strong FTR
FTR

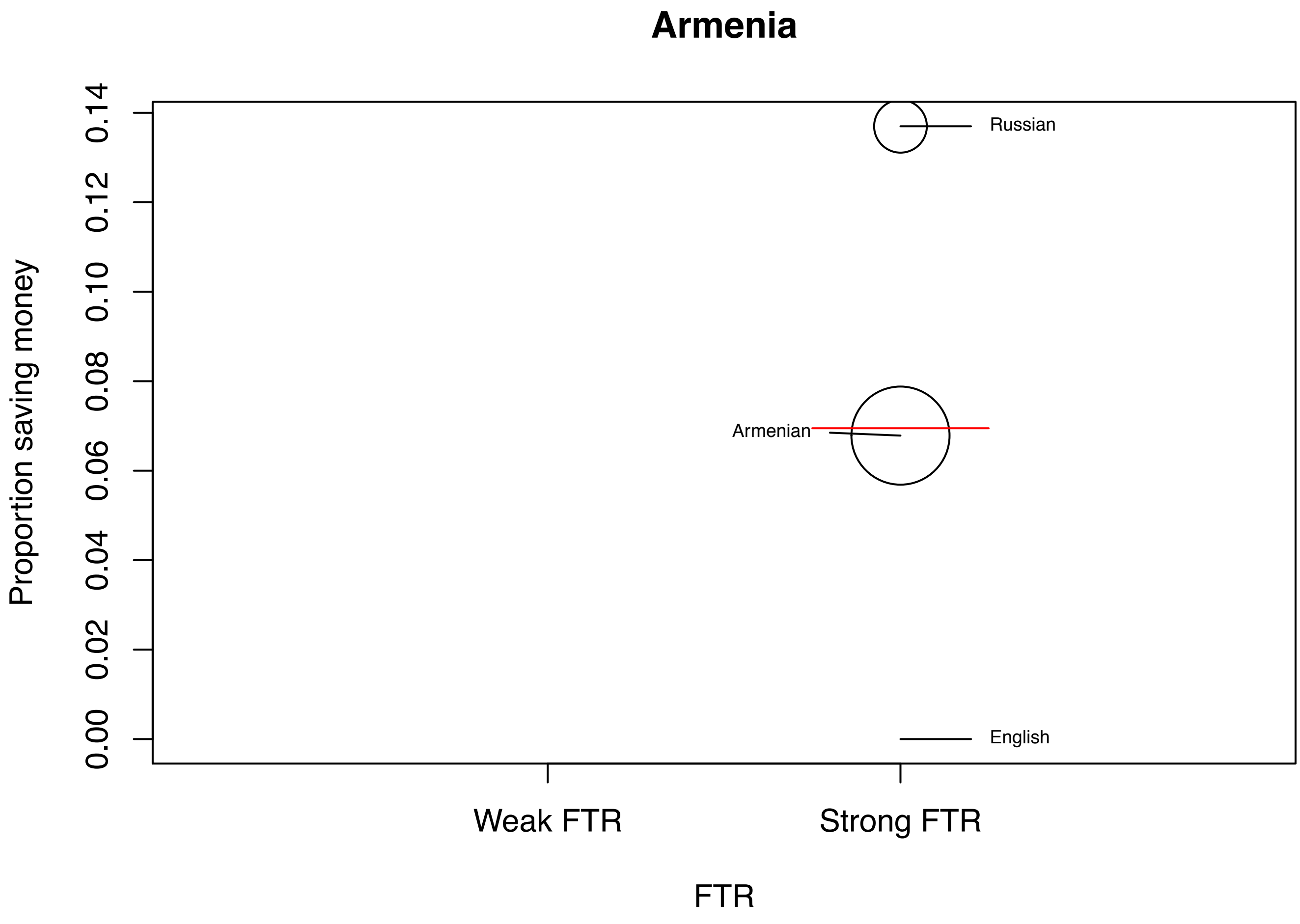
Armenia
Russian
Armenian
English
Proportion saving money
0.00
0.02
0.04
0.06
0.08
0.10
0.12
0.14
Weak FTR
Strong FTR
FTR

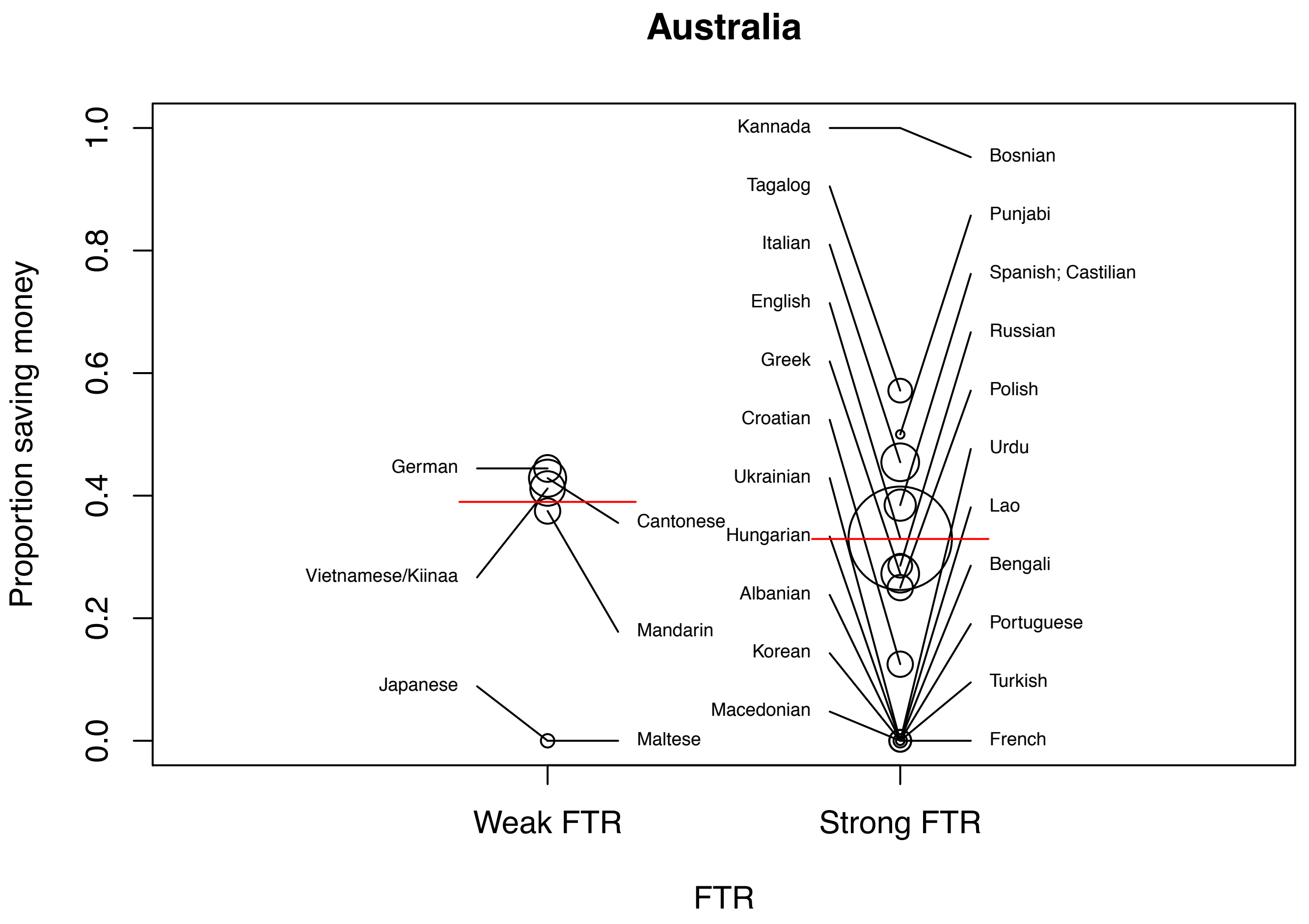
Australia
Proportion saving money
0.0
0.2
0.4
0.6
0.8
1.0
German
Vietnamese/Kiinaa
Japanese
Cantonese
Mandarin
Maltese
Kannada
Tagalog
Italian
English
Greek
Croatian
Ukrainian
Hungarian
Albanian
Korean
Macedonian
Bosnian
Punjabi
Spanish; Castilian
Russian
Polish
Urdu
Lao
Bengali
Portuguese
Turkish
French
Weak FTR
Strong FTR
FTR

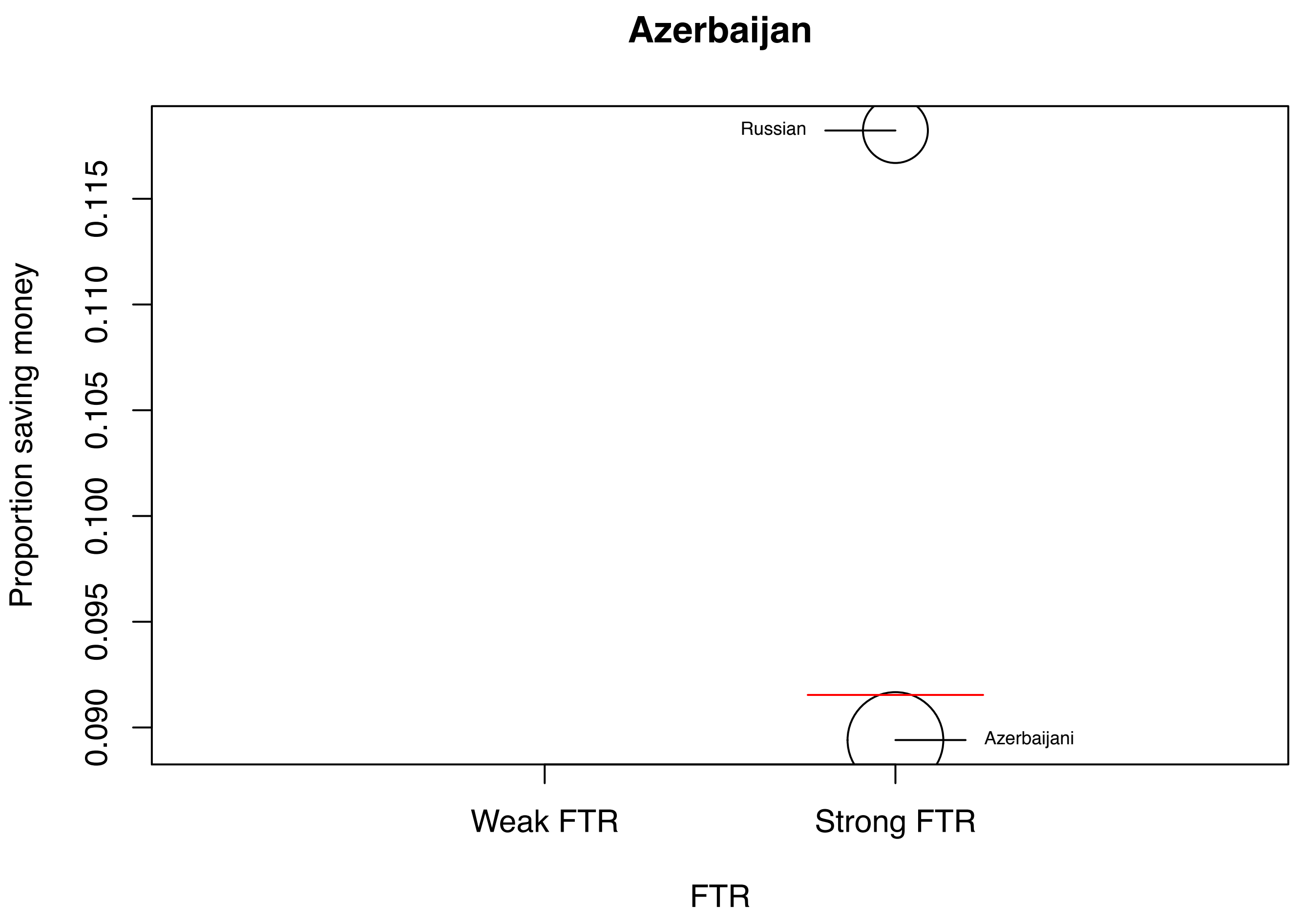
Azerbaijan
Proportion saving money
0.090
0.095
0.100
0.105
0.110
0.115
Russian
Azerbaijani
Weak FTR
Strong FTR
FTR

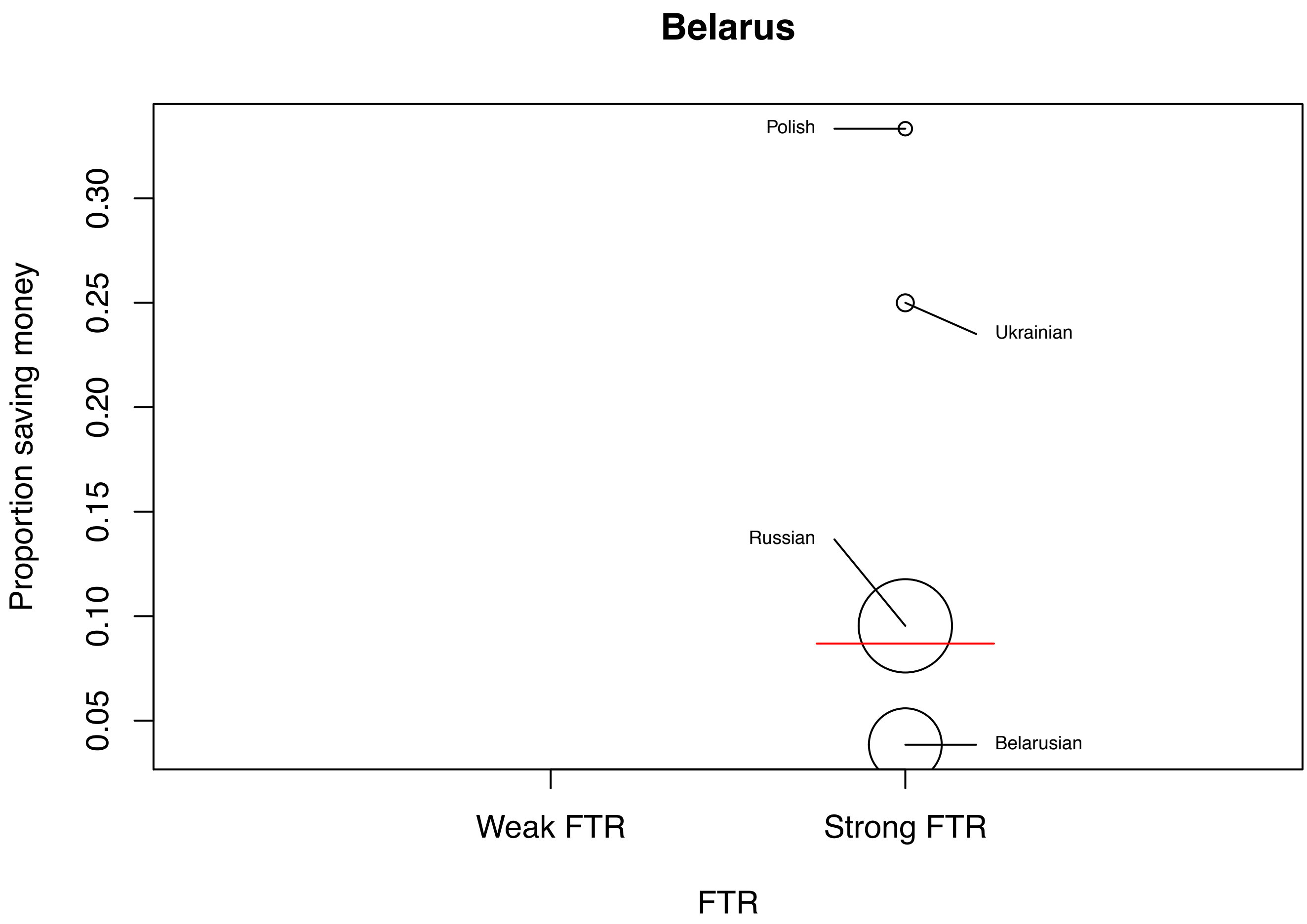
Belarus
Polish
Ukrainian
Russian
Belarusian
0.05
0.10
0.15
0.20
0.25
0.30
Proportion saving money
Weak FTR
Strong FTR
FTR

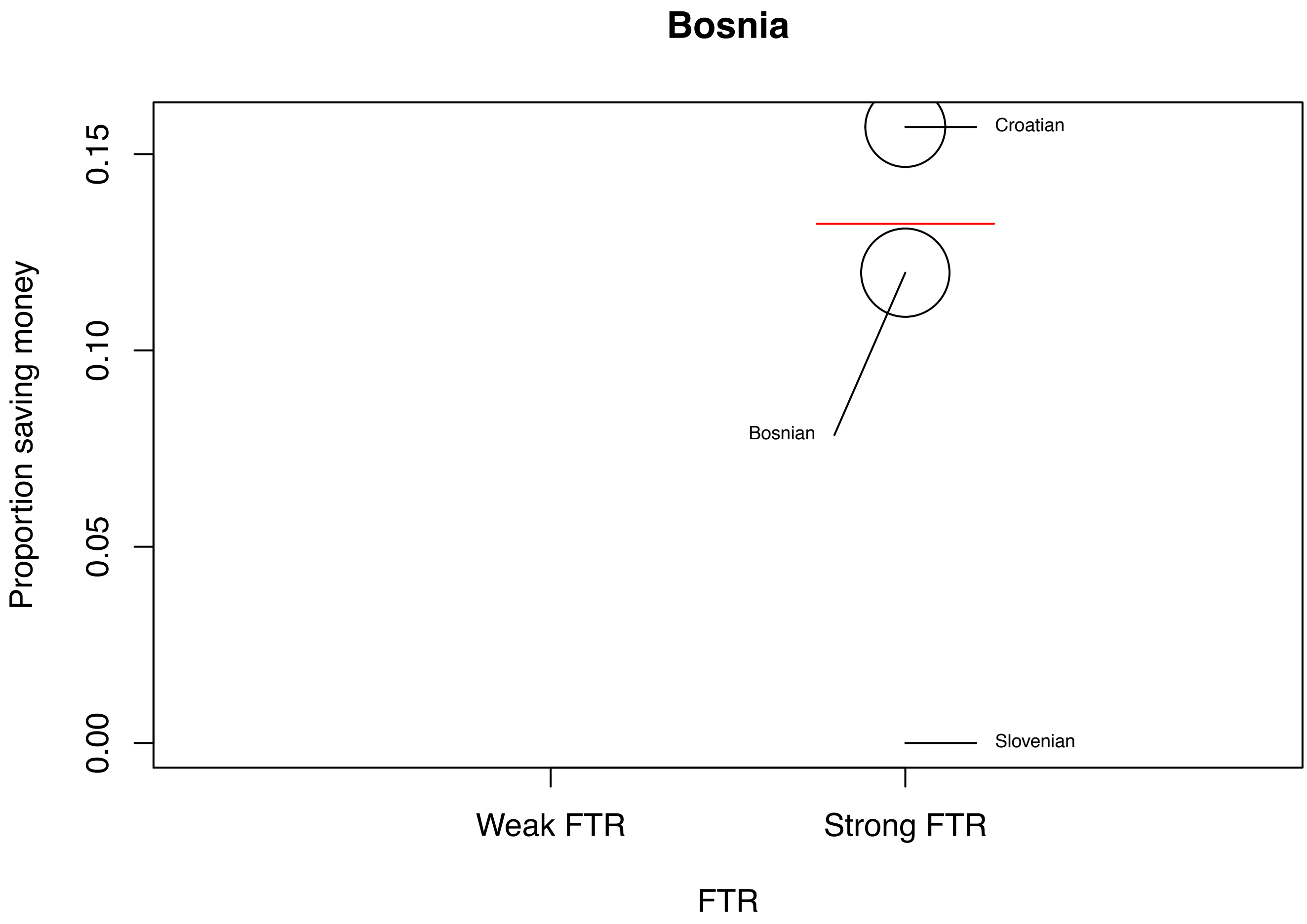
Bosnia
Croatian
Bosnian
Slovenian
0.00
0.05
0.10
0.15
Proportion saving money
Weak FTR
Strong FTR
FTR

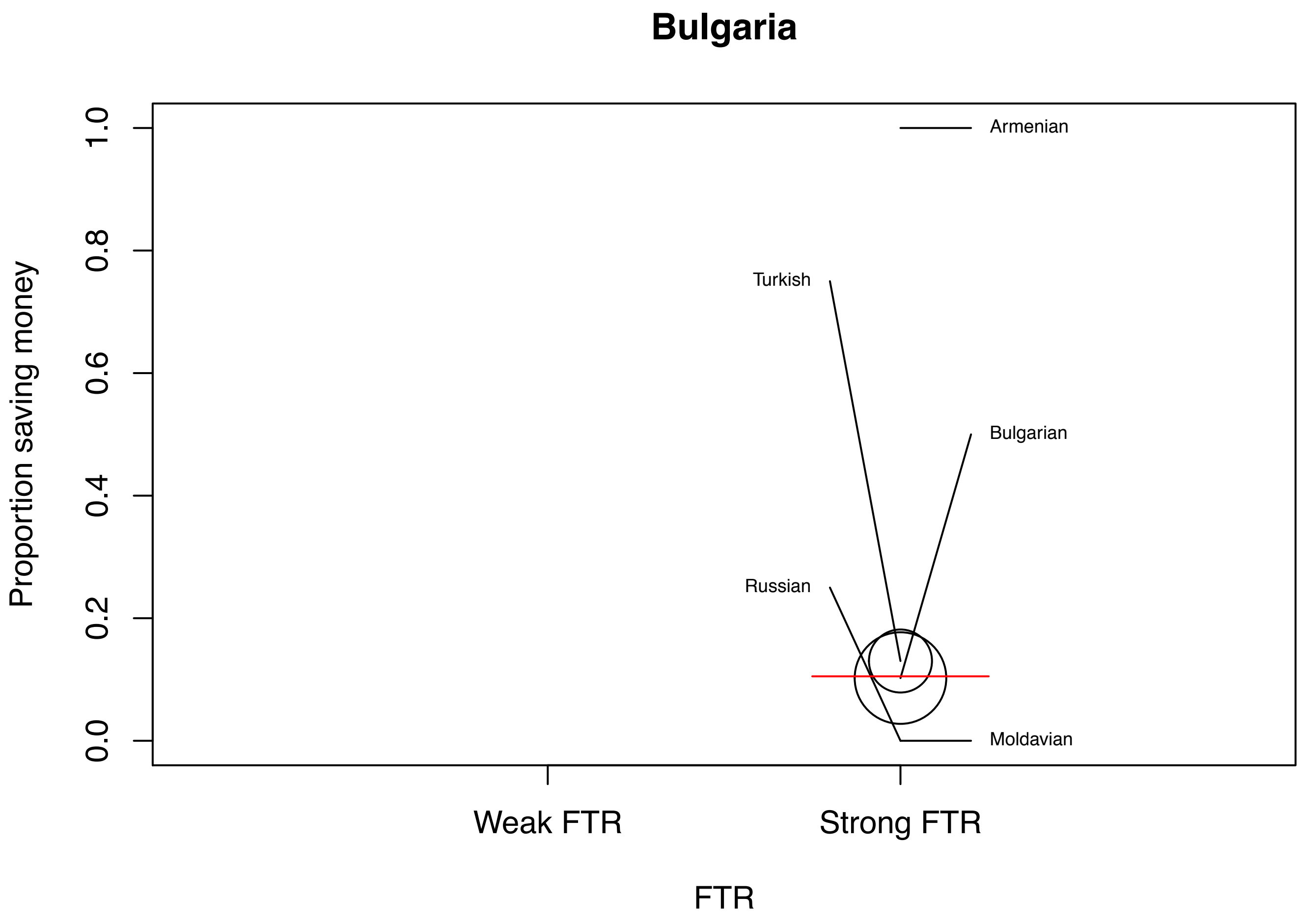
Bulgaria
Armenian
Turkish
Bulgarian
Russian
Moldavian
1.0
0.8
0.6
0.4
0.2
0.0
Proportion saving money
Weak FTR
Strong FTR
FTR

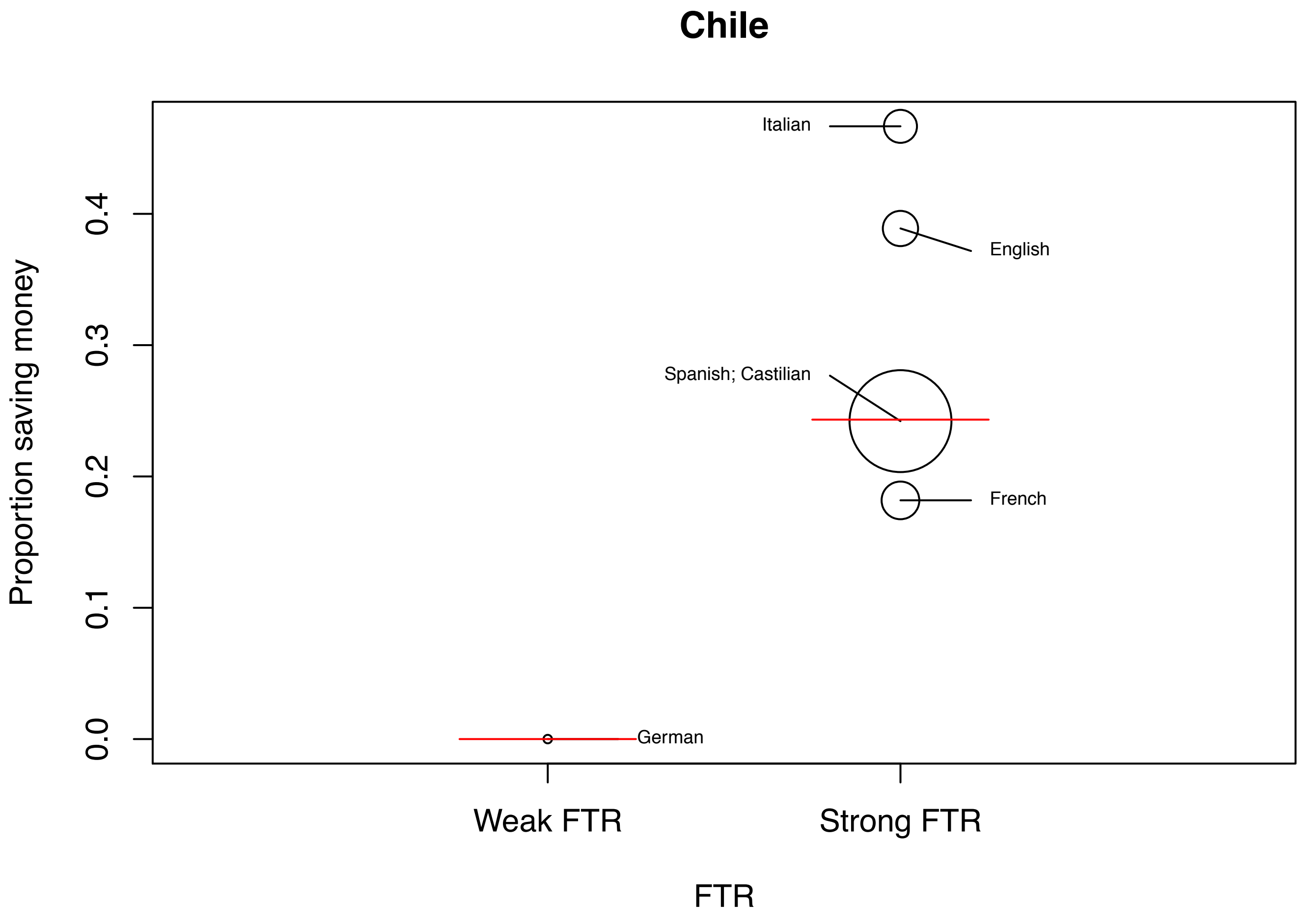
Chile
Italian
English
Spanish; Castilian
French
German
0.4
0.3
0.2
0.1
0.0
Proportion saving money
Weak FTR
Strong FTR
FTR

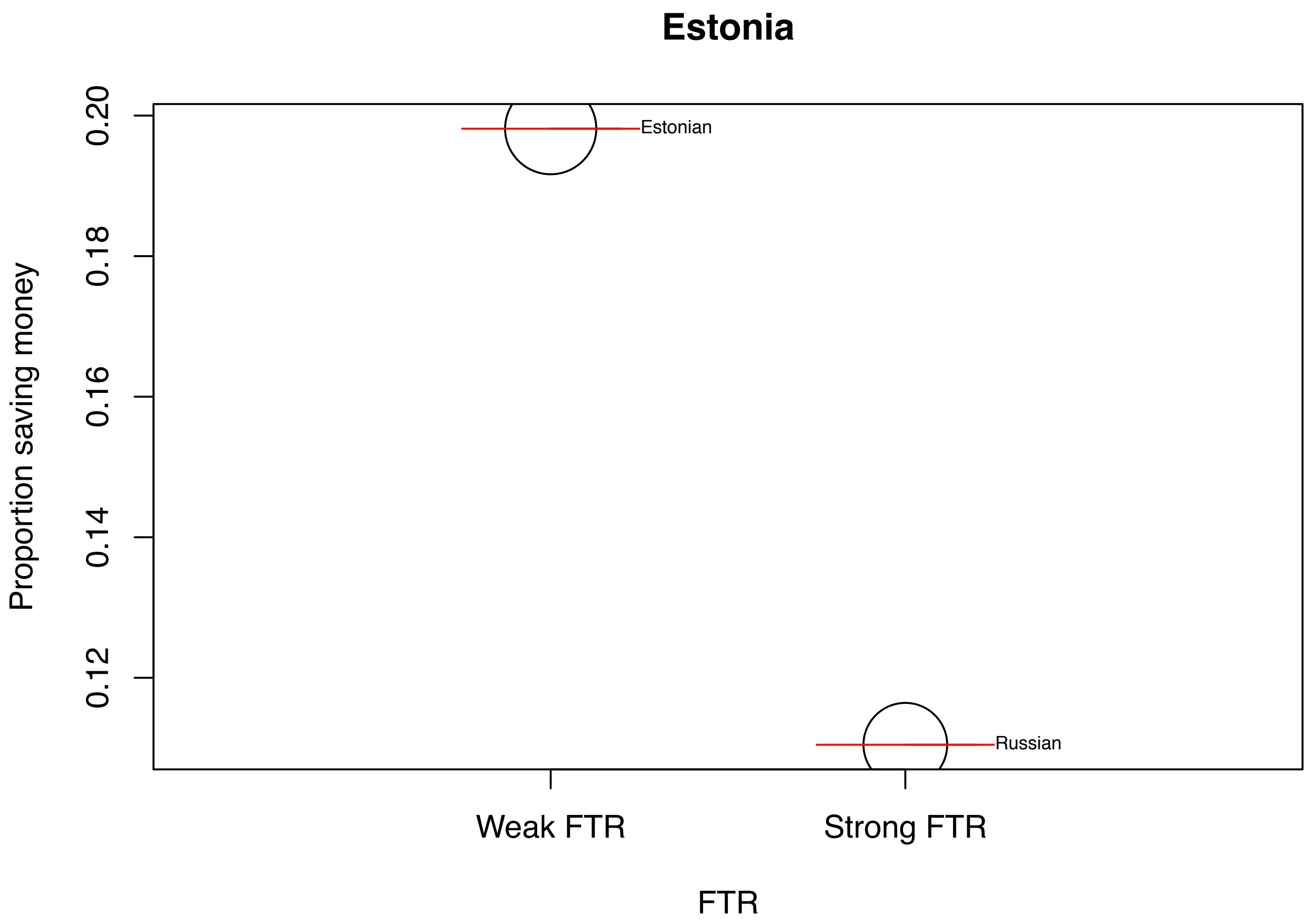
Estonia
Proportion saving money
0.20
0.18
0.16
0.14
0.12
Estonian
Russian
Weak FTR
Strong FTR
FTR

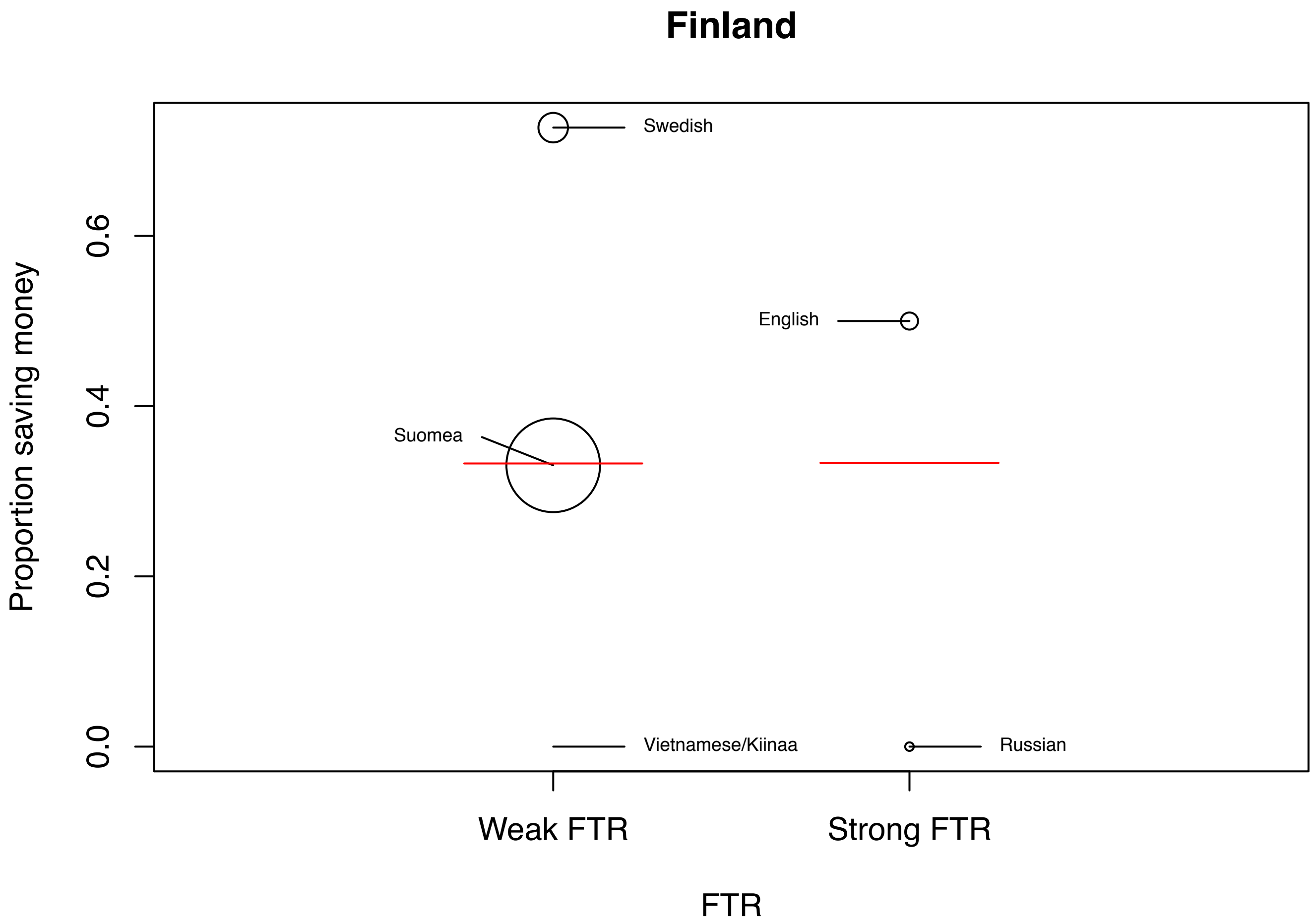
Finland
Proportion saving money
0.6
0.4
0.2
0.0
Swedish
English
Suomea
Vietnamese/Kiinaa
Russian
Weak FTR
Strong FTR
FTR

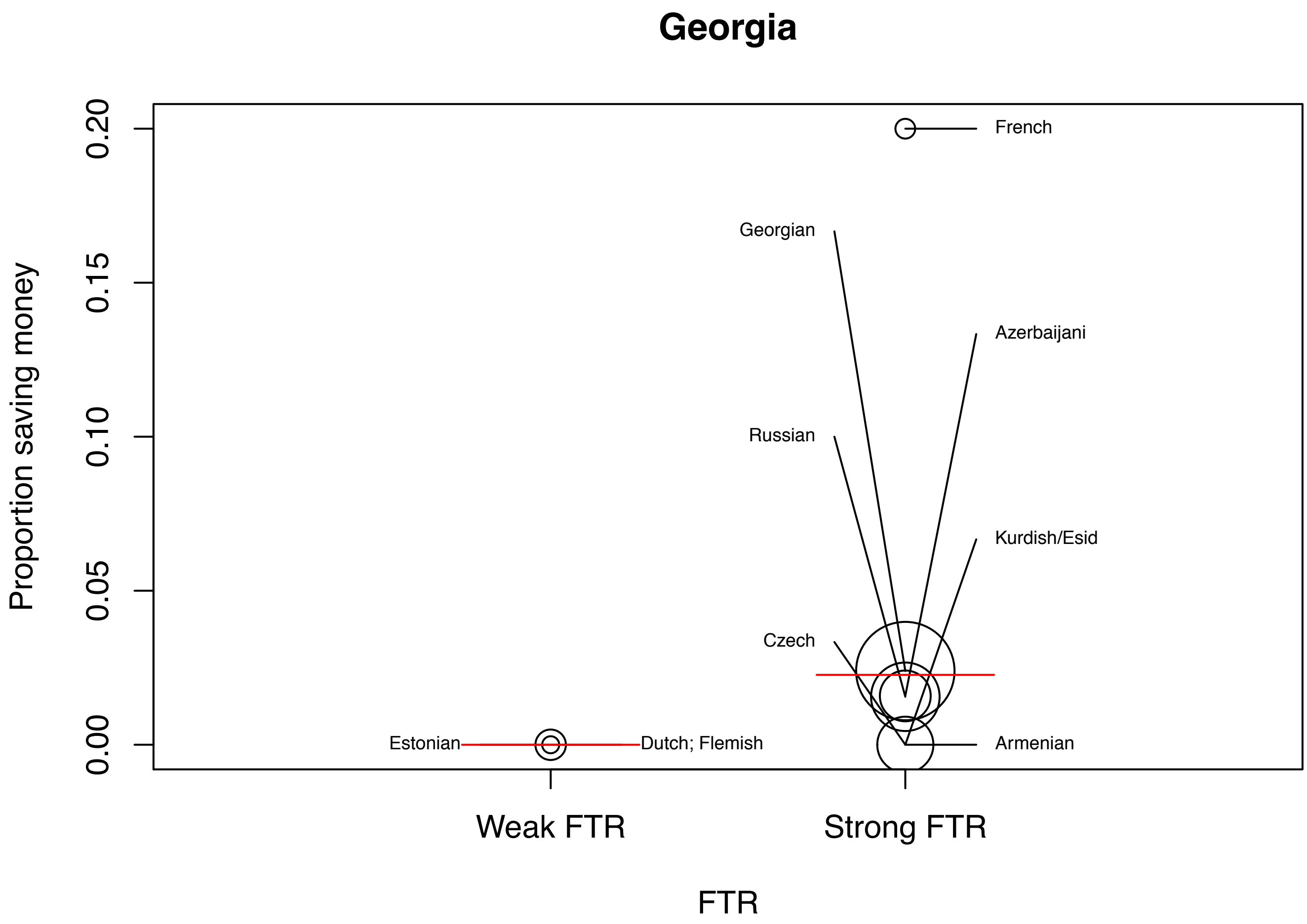

Georgia
Proportion saving money
0.00
0.05
0.10
0.15
0.20
French
Georgian
Azerbaijani
Russian
Kurdish/Esid
Czech
Estonian
Dutch; Flemish
Armenian
Weak FTR
Strong FTR
FTR

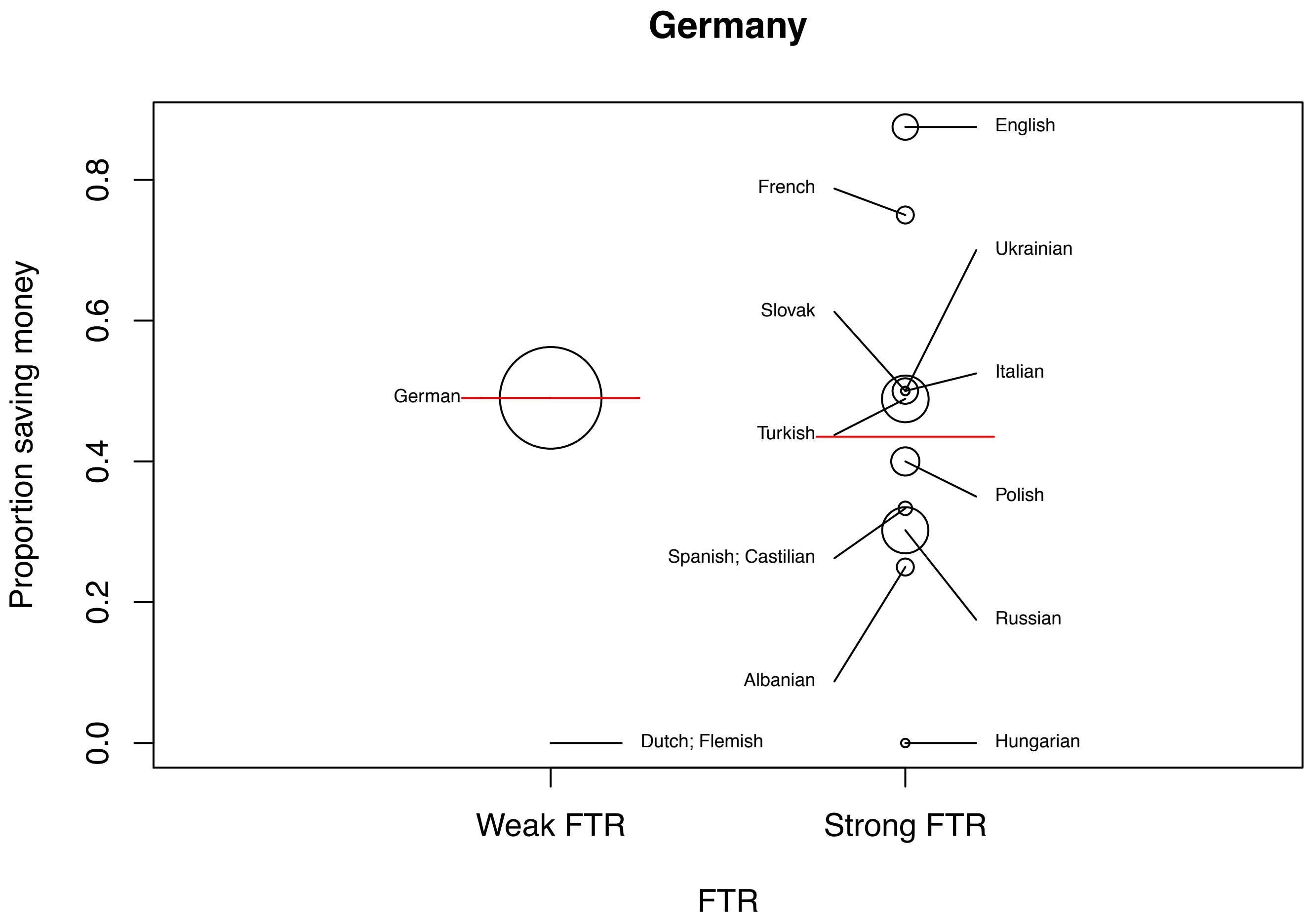

Germany
Proportion saving money
0.0
0.2
0.4
0.6
0.8
English
French
Ukrainian
Slovak
Italian
German
Turkish
Polish
Spanish; Castilian
Russian
Albanian
Dutch; Flemish
Hungarian
Weak FTR
Strong FTR
FTR

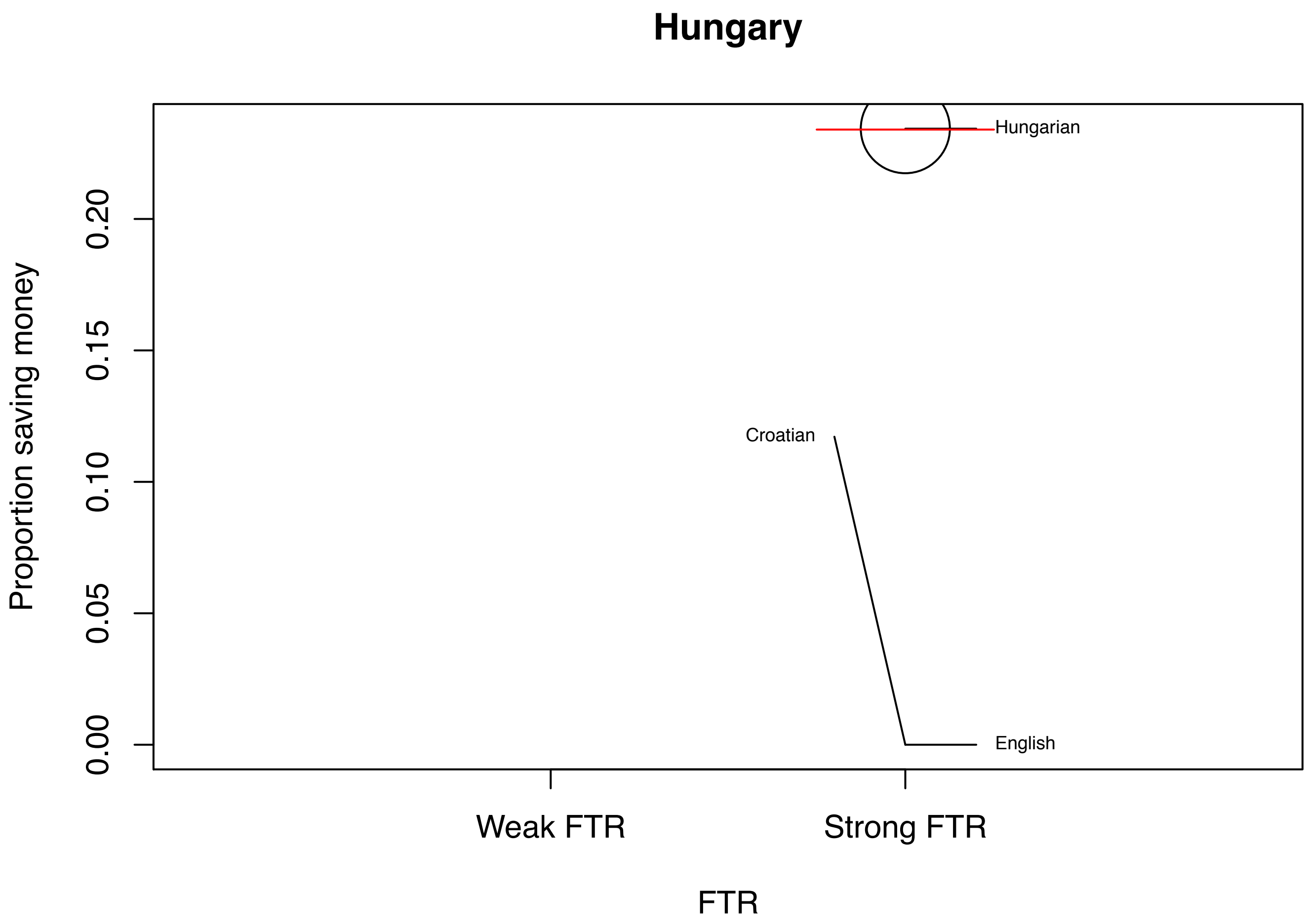
Hungary
Proportion saving money
0.00
0.05
0.10
0.15
0.20
Hungarian
Croatian
English
Weak FTR
Strong FTR
FTR

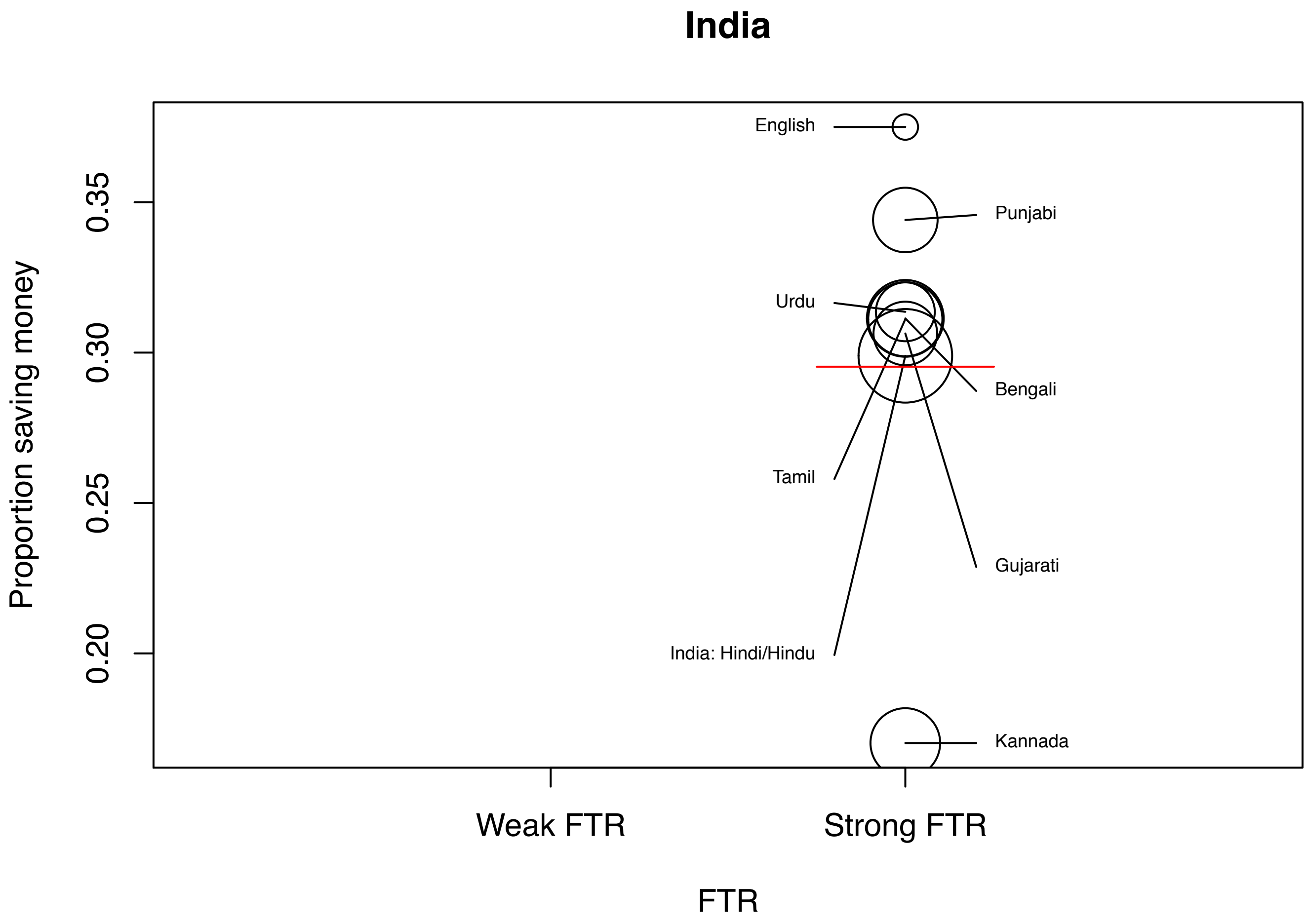
India
Proportion saving money
0.20
0.25
0.30
0.35
English
Punjabi
Urdu
Bengali
Tamil
Gujarati
India: Hindi/Hindu
Kannada
Weak FTR
Strong FTR
FTR

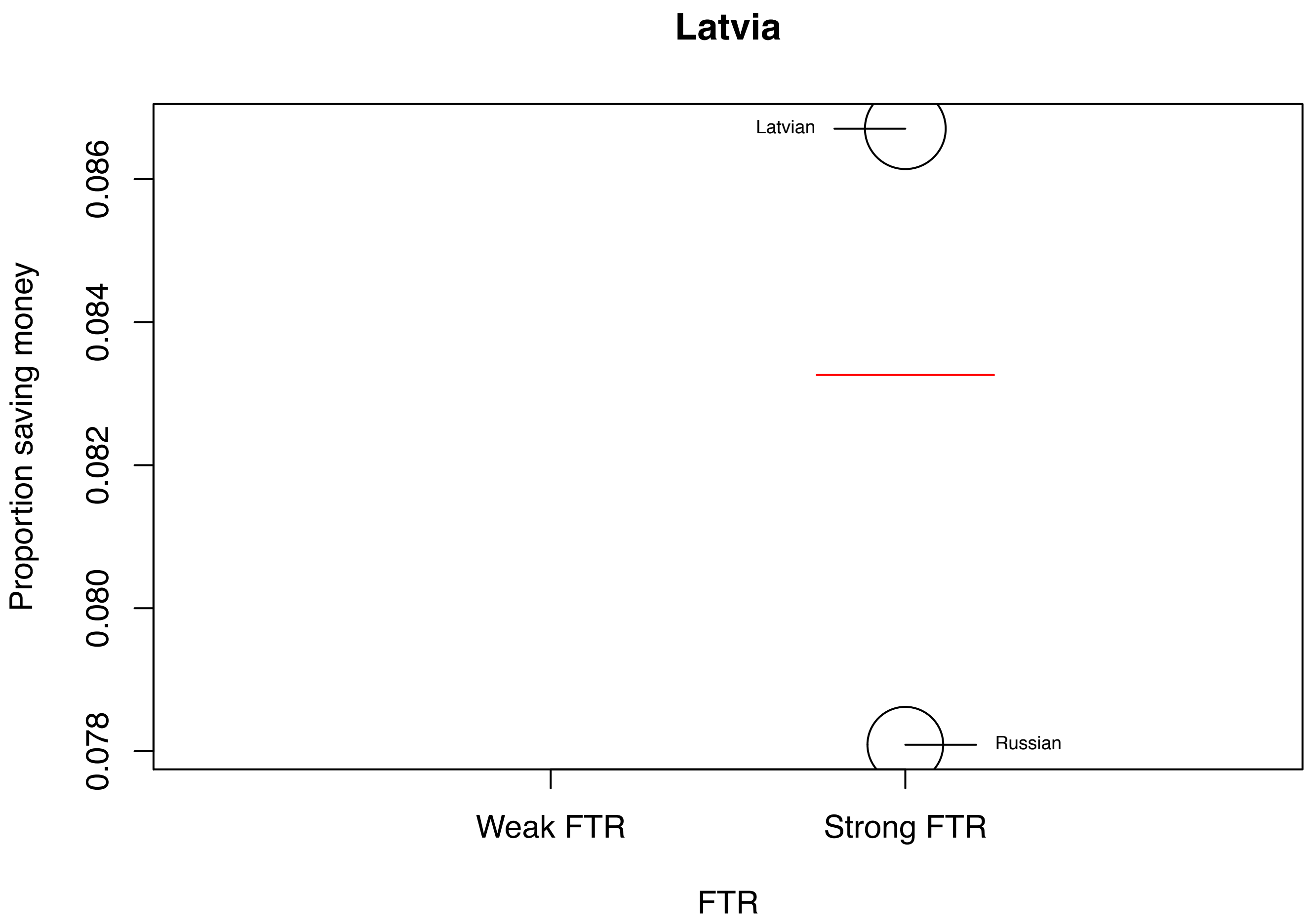

Latvia
Latvian
Russian
0.078
0.080
0.082
0.084
0.086
Proportion saving money
Weak FTR
Strong FTR
FTR

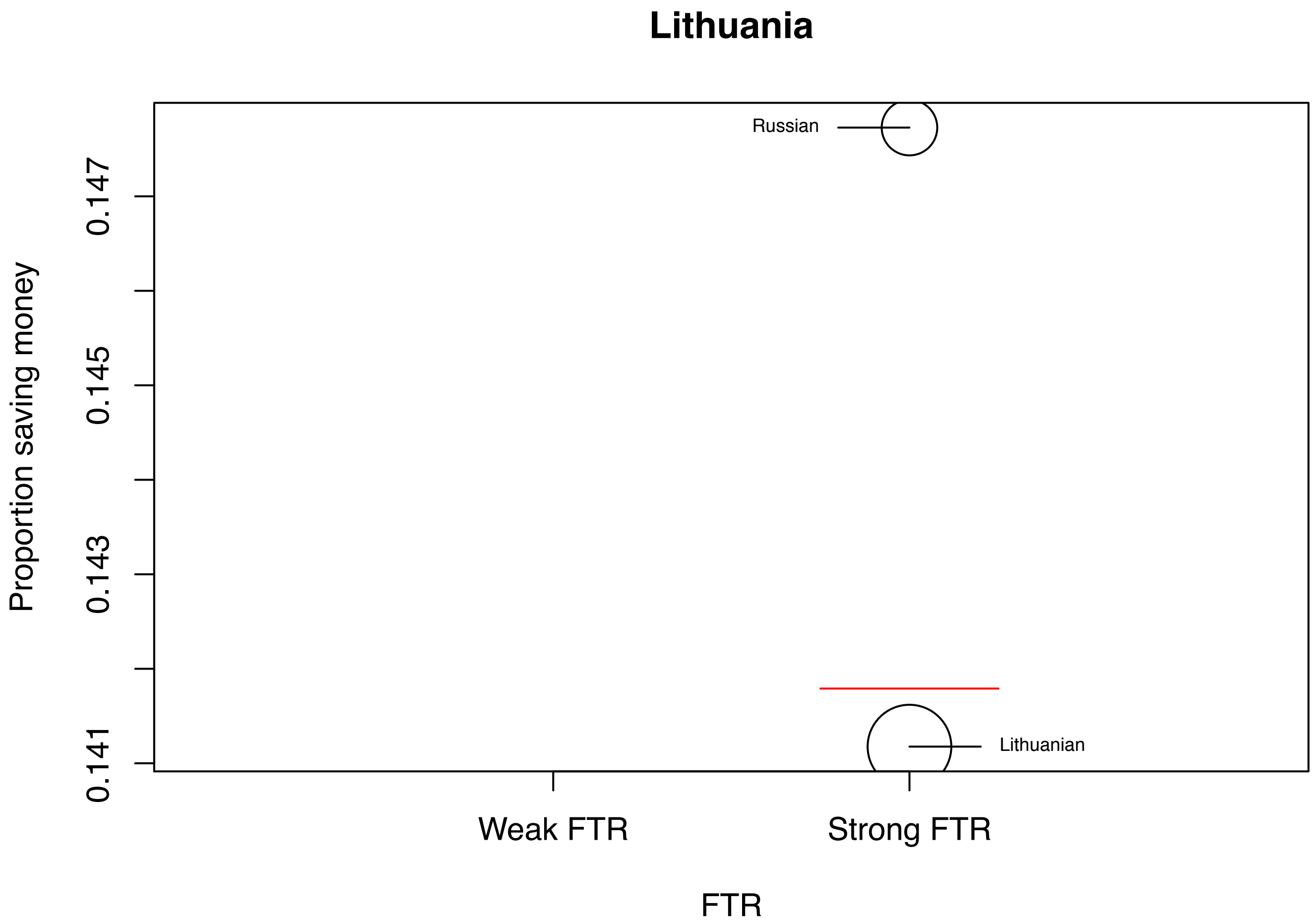

Lithuania
Russian
Lithuanian
0.141
0.143
0.145
0.147
Proportion saving money
Weak FTR
Strong FTR
FTR

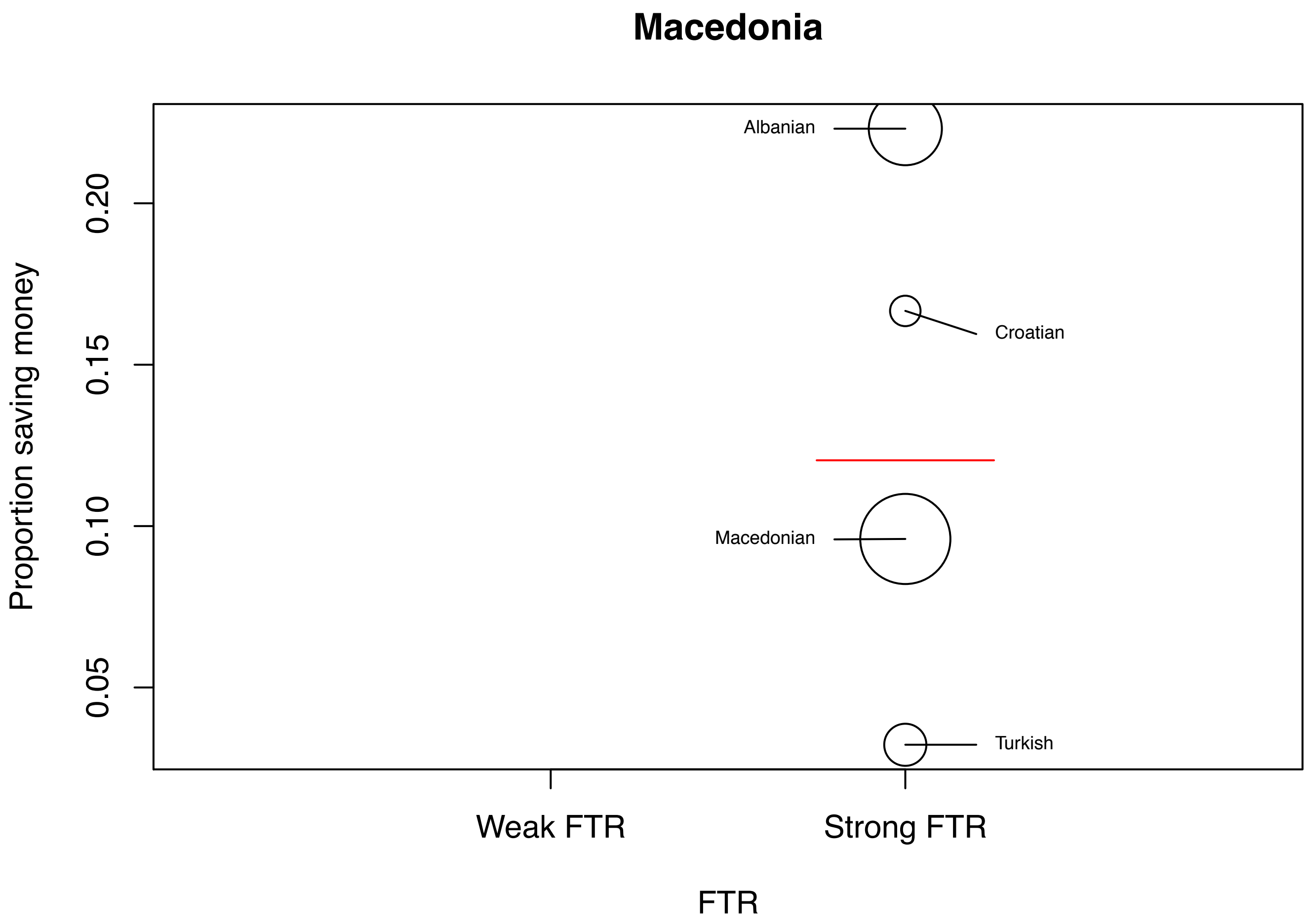

Macedonia
Albanian
Croatian
Macedonian
Turkish
0.05
0.10
0.15
0.20
Proportion saving money
Weak FTR
Strong FTR
FTR

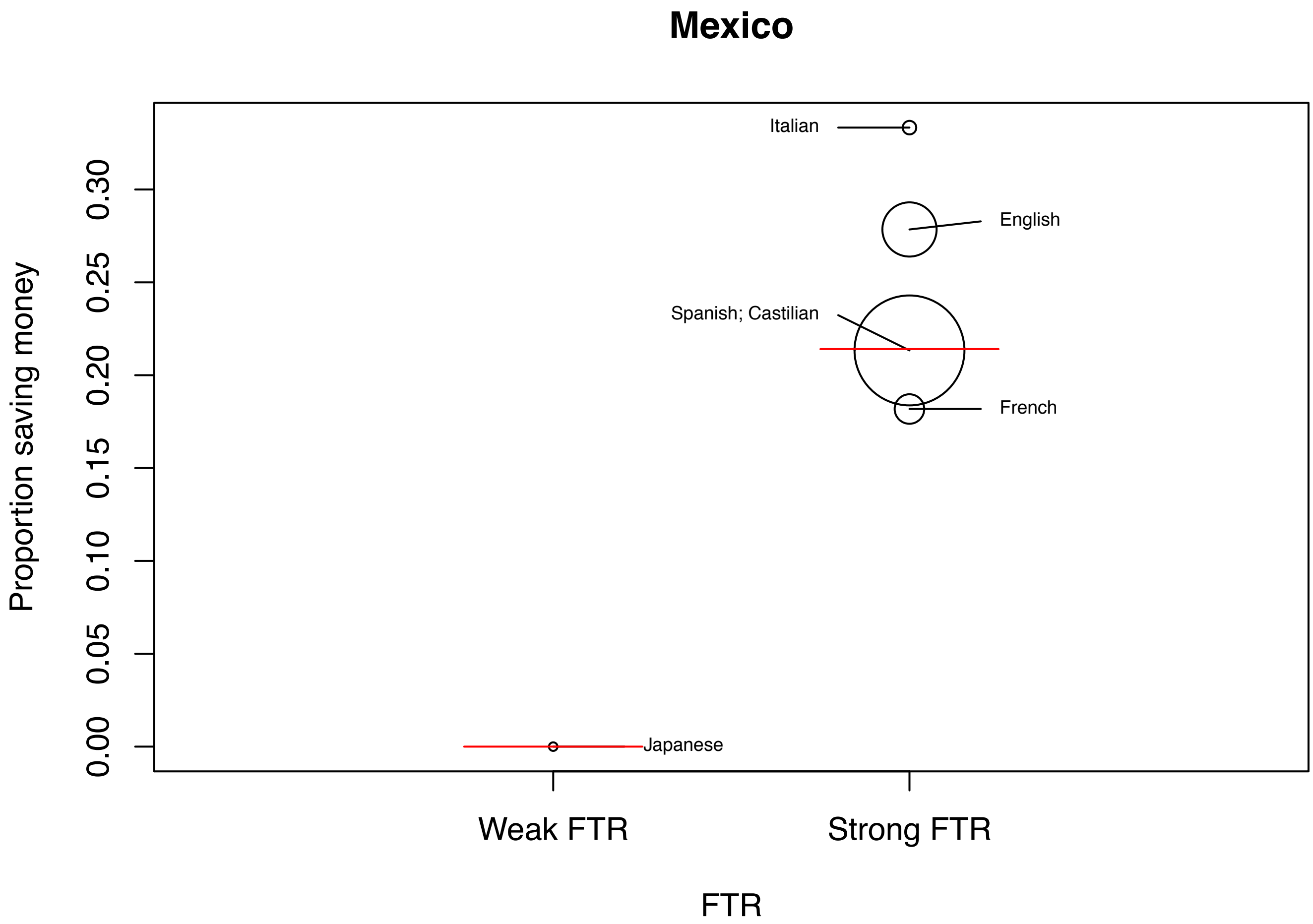

Mexico
Italian
English
Spanish; Castilian
French
Japanese
0.00
0.05
0.10
0.15
0.20
0.25
0.30
Proportion saving money
Weak FTR
Strong FTR
FTR

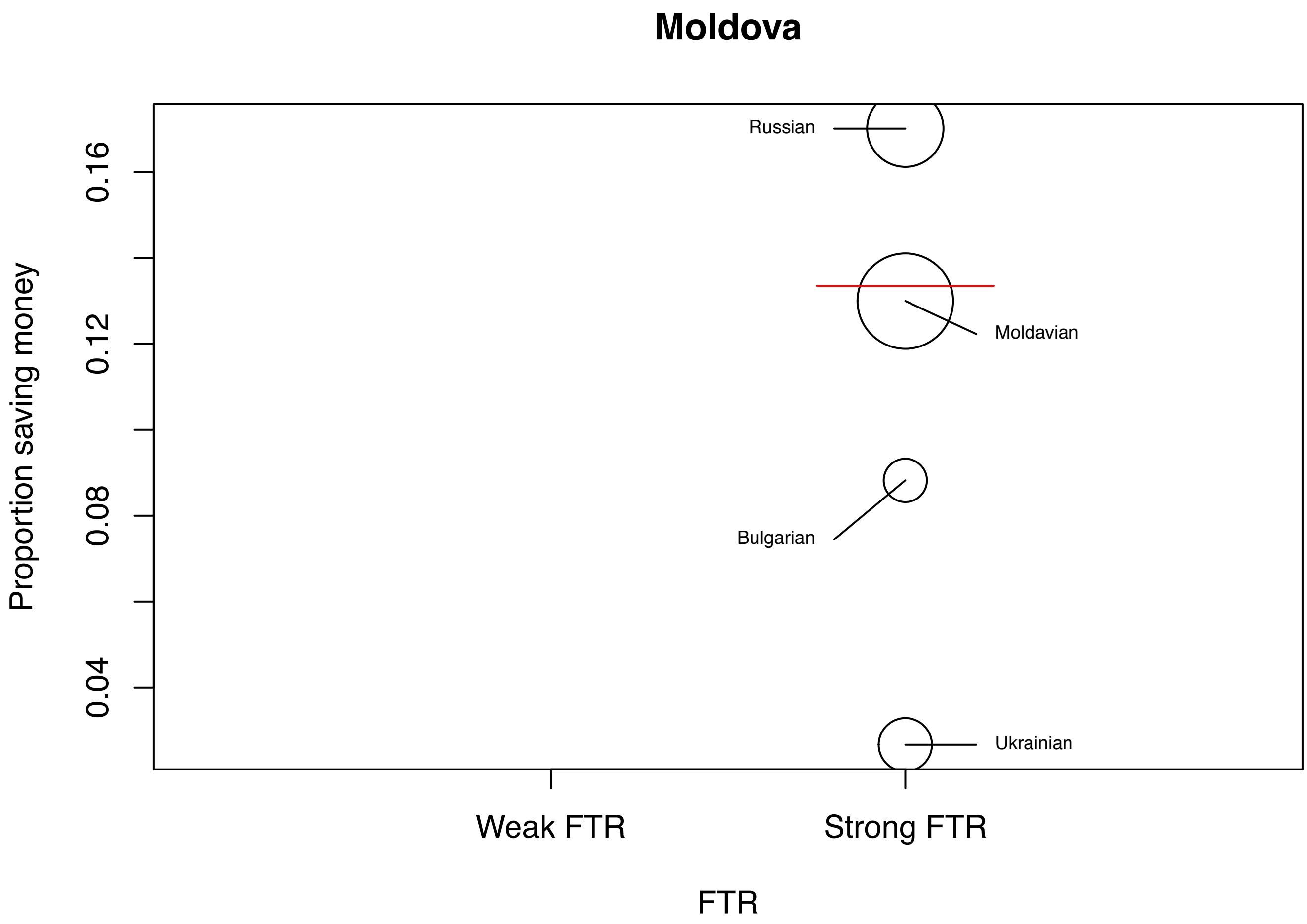

Moldova
Russian
Moldavian
Bulgarian
Ukrainian
Proportion saving money
0.04
0.08
0.12
0.16
Weak FTR
Strong FTR
FTR

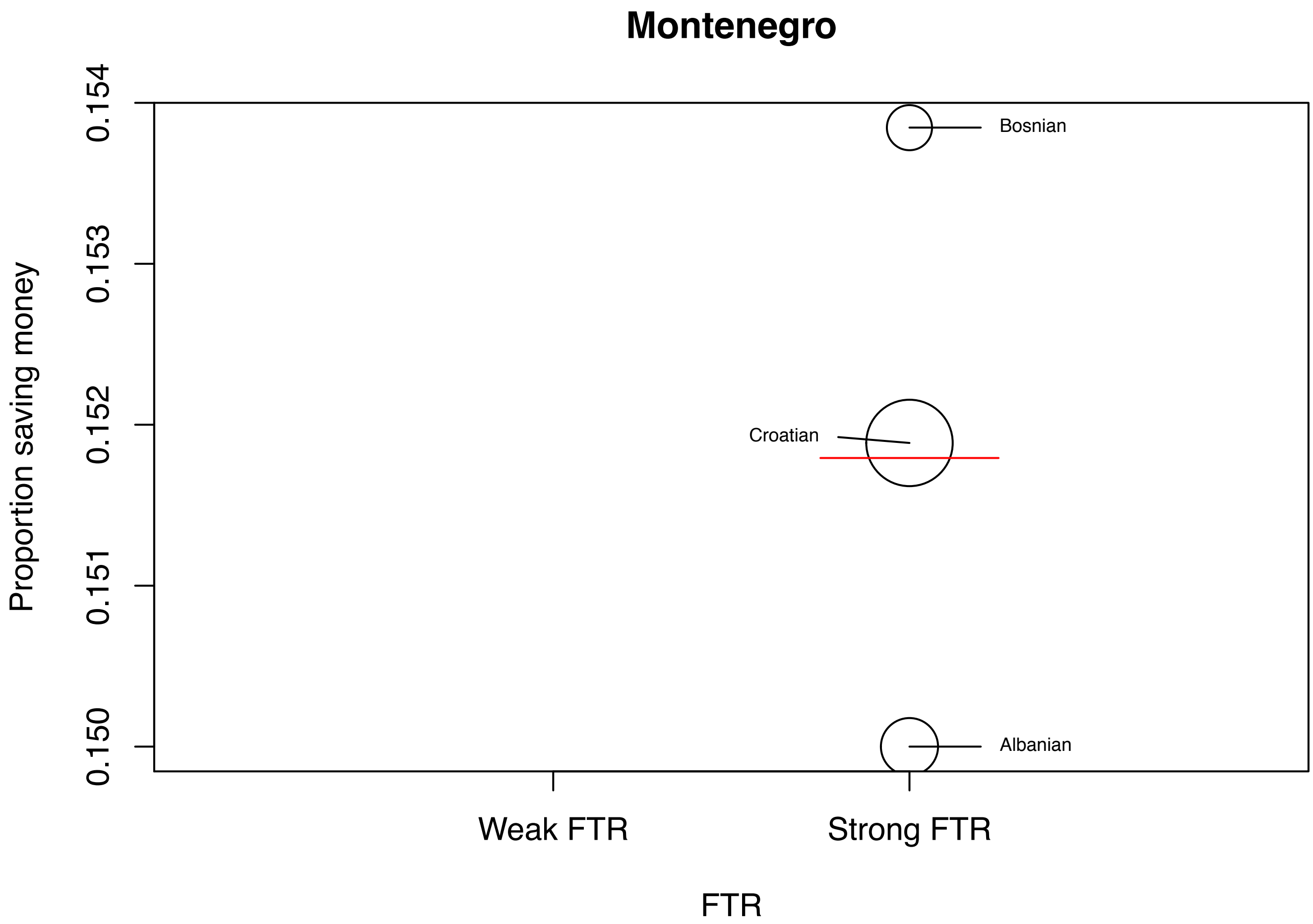

Montenegro
Bosnian
Croatian
Albanian
Proportion saving money
0.150
0.151
0.152
0.153
0.154
Weak FTR
Strong FTR
FTR

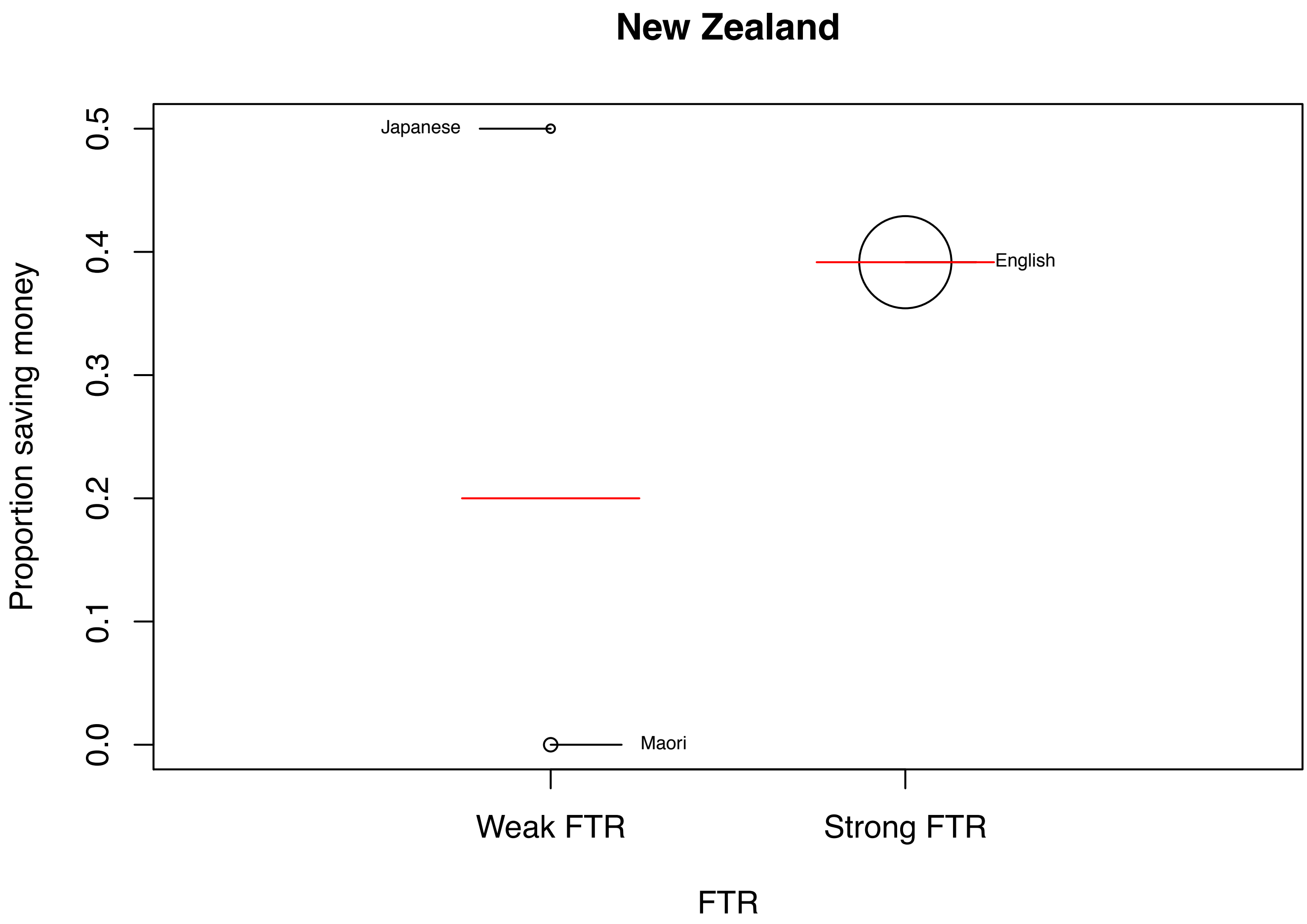
New Zealand
Proportion saving money
0.0
0.1
0.2
0.3
0.4
0.5
Japanese
English
Maori
Weak FTR
Strong FTR
FTR

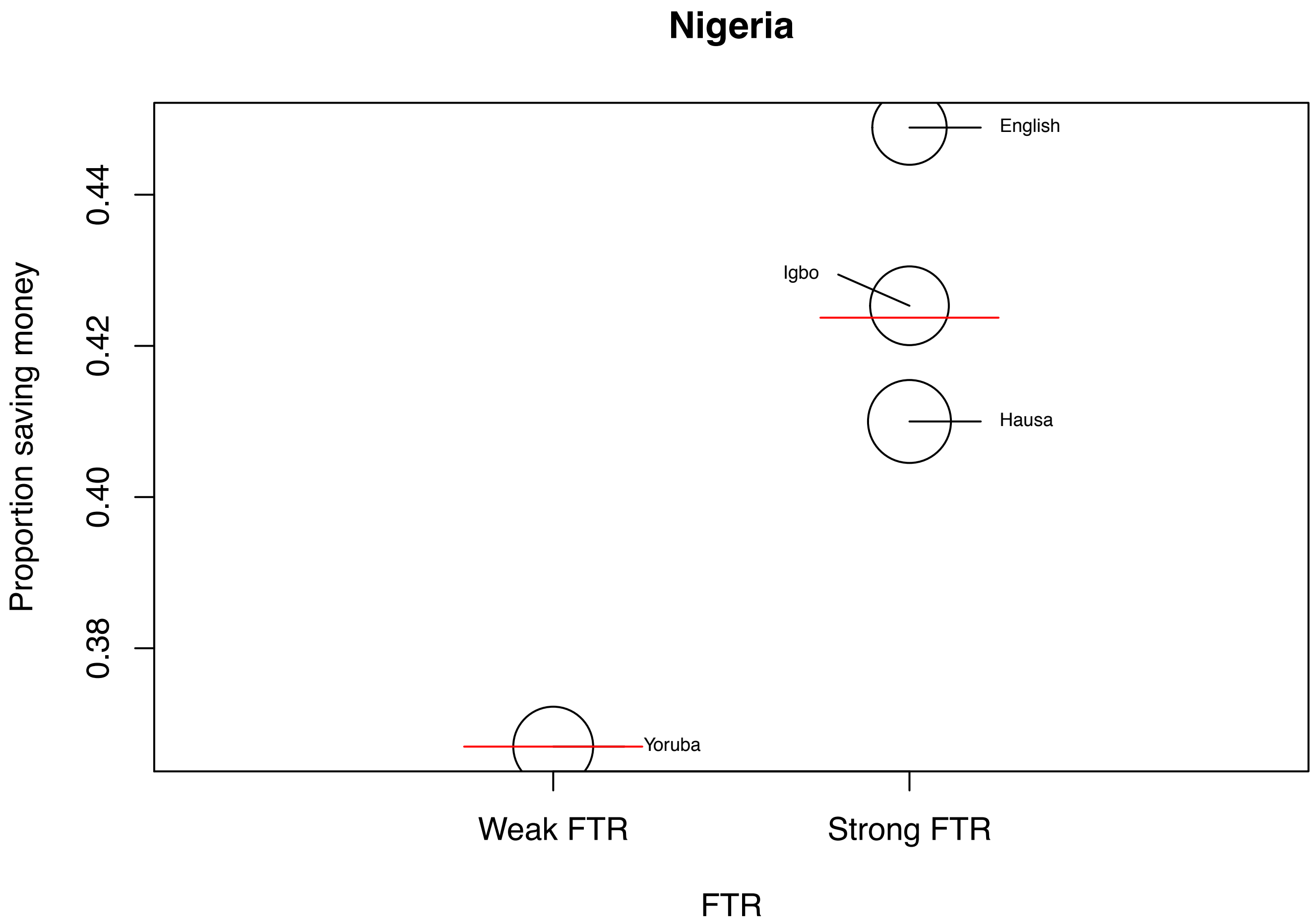
Nigeria
Proportion saving money
0.38
0.40
0.42
0.44
English
Igbo
Hausa
Yoruba
Weak FTR
Strong FTR
FTR

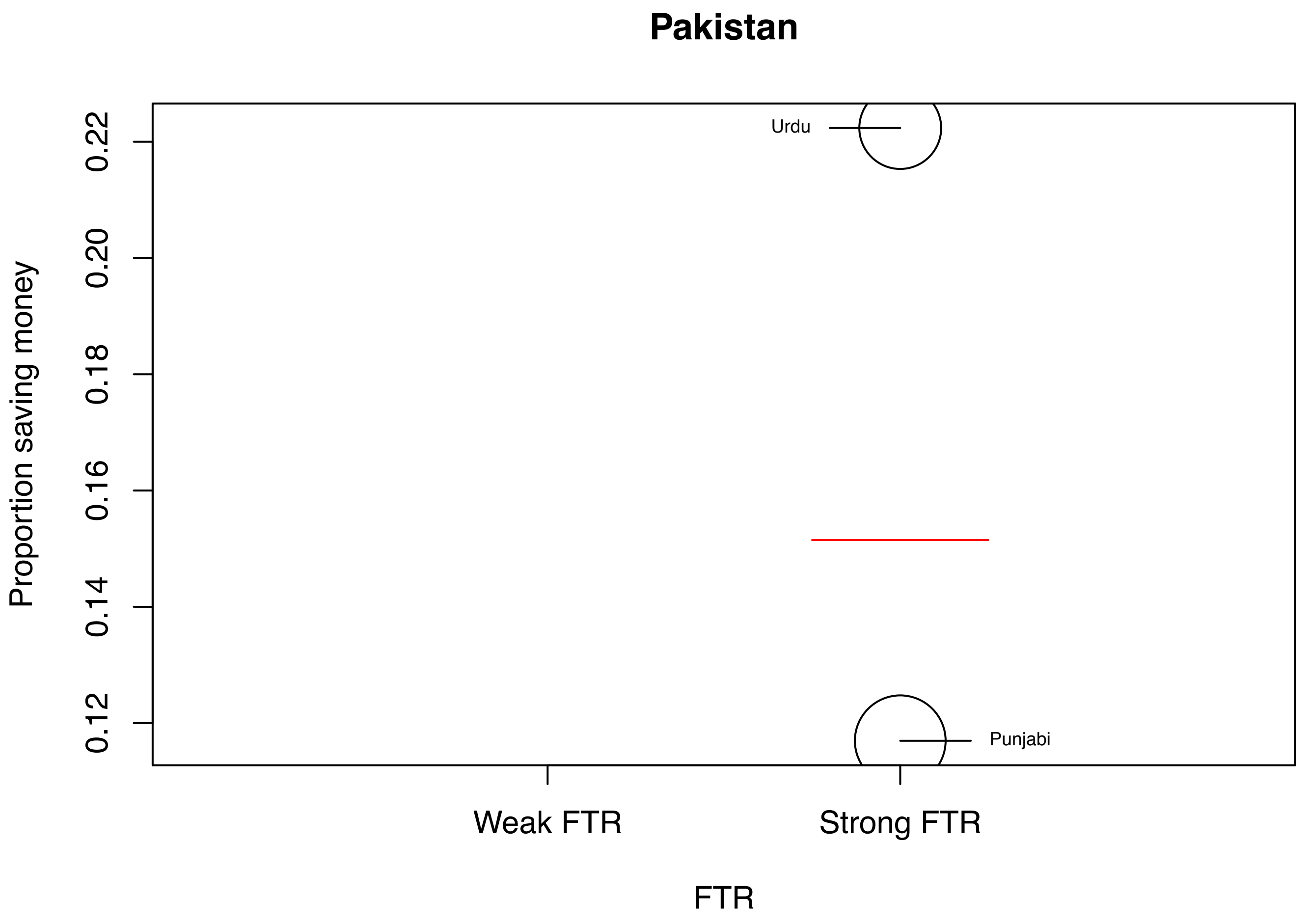
Pakistan
Urdu
Punjabi
Proportion saving money
0.12
0.14
0.16
0.18
0.20
0.22
Weak FTR
Strong FTR
FTR

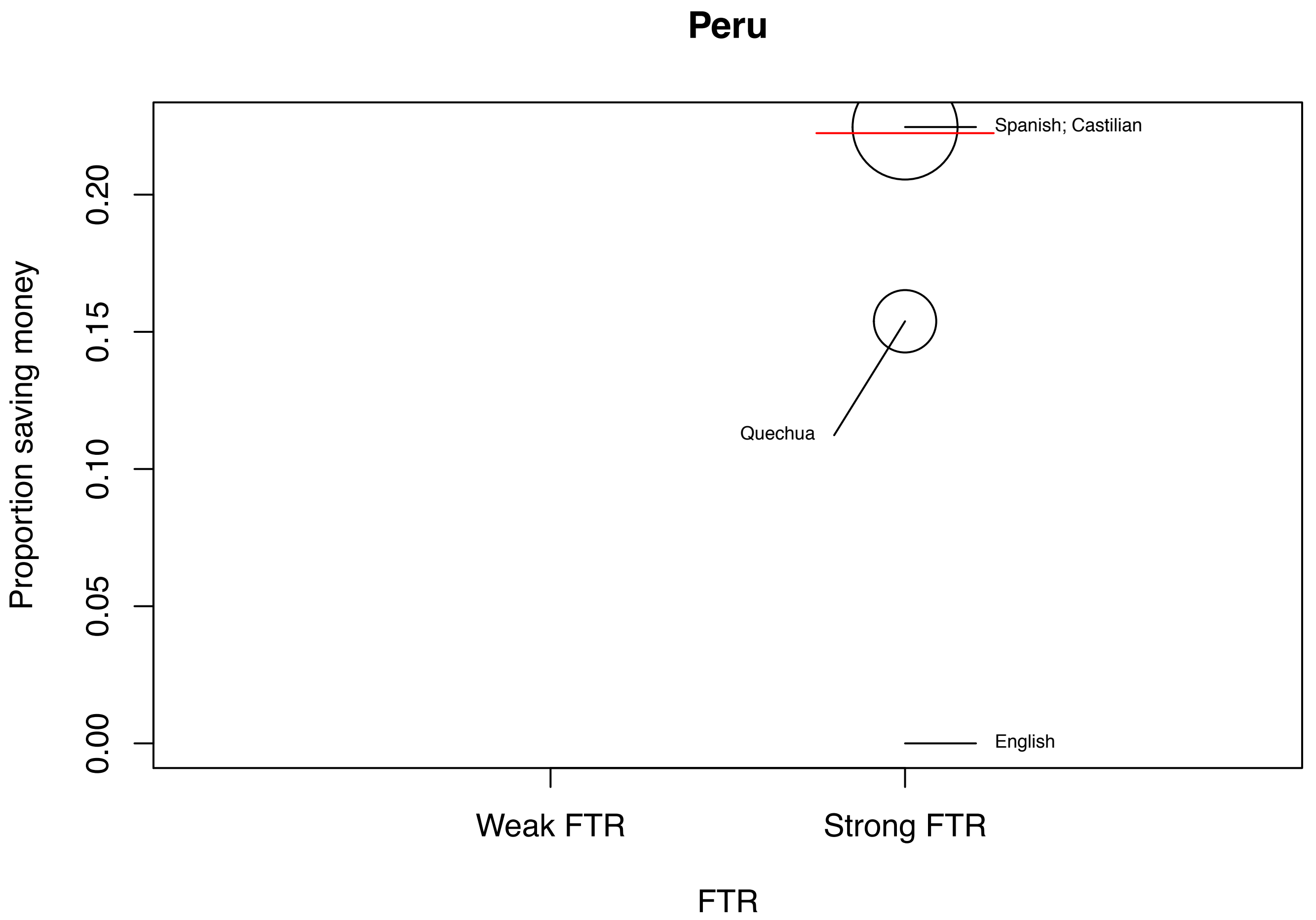
Peru
Spanish; Castilian
Quechua
English
Proportion saving money
0.00
0.05
0.10
0.15
0.20
Weak FTR
Strong FTR
FTR

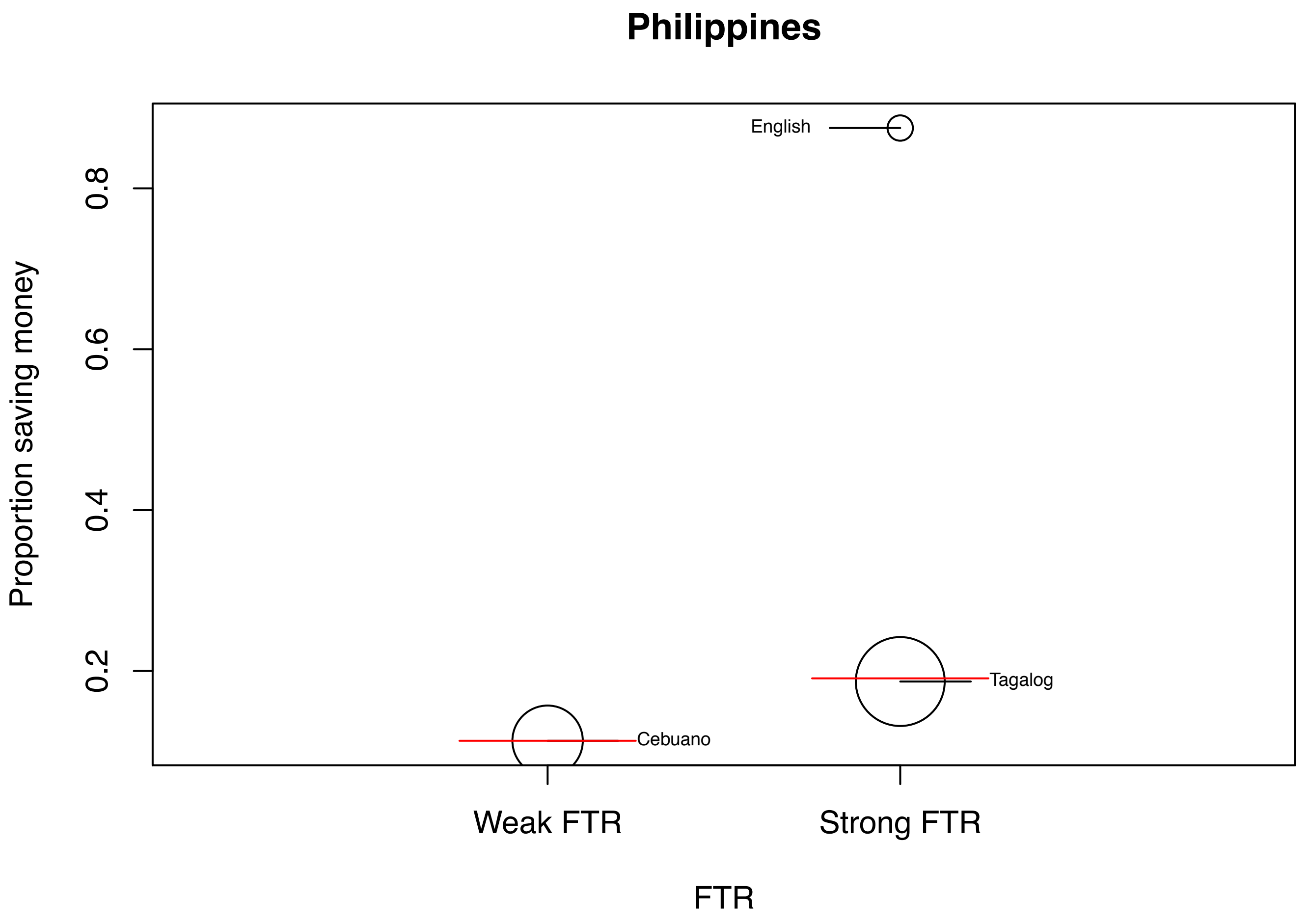
Philippines
English
Tagalog
Cebuano
Proportion saving money
0.2
0.4
0.6
0.8
Weak FTR
Strong FTR
FTR

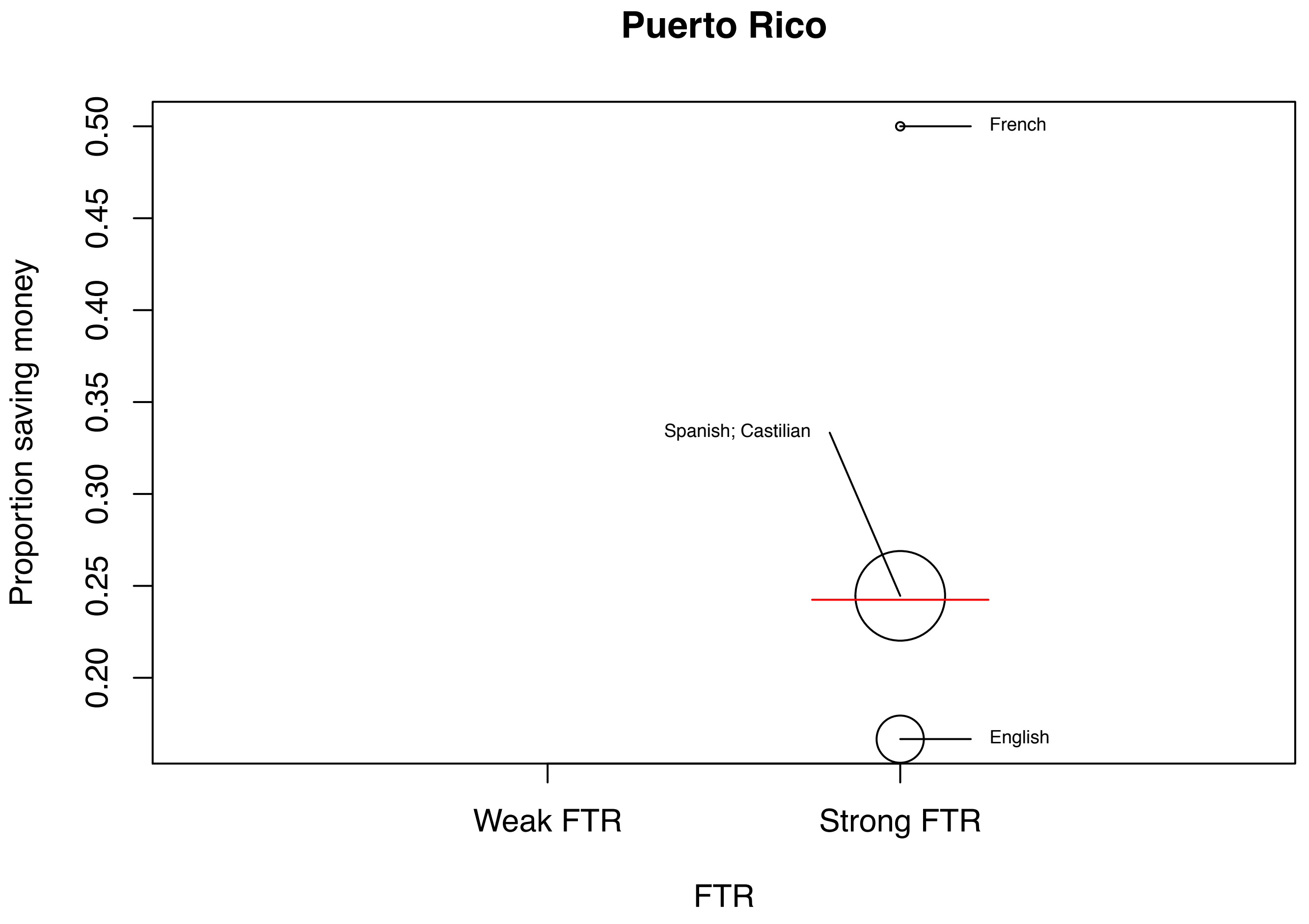
Puerto Rico
French
Spanish; Castilian
English
Proportion saving money
0.20
0.25
0.30
0.35
0.40
0.45
0.50
Weak FTR
Strong FTR
FTR

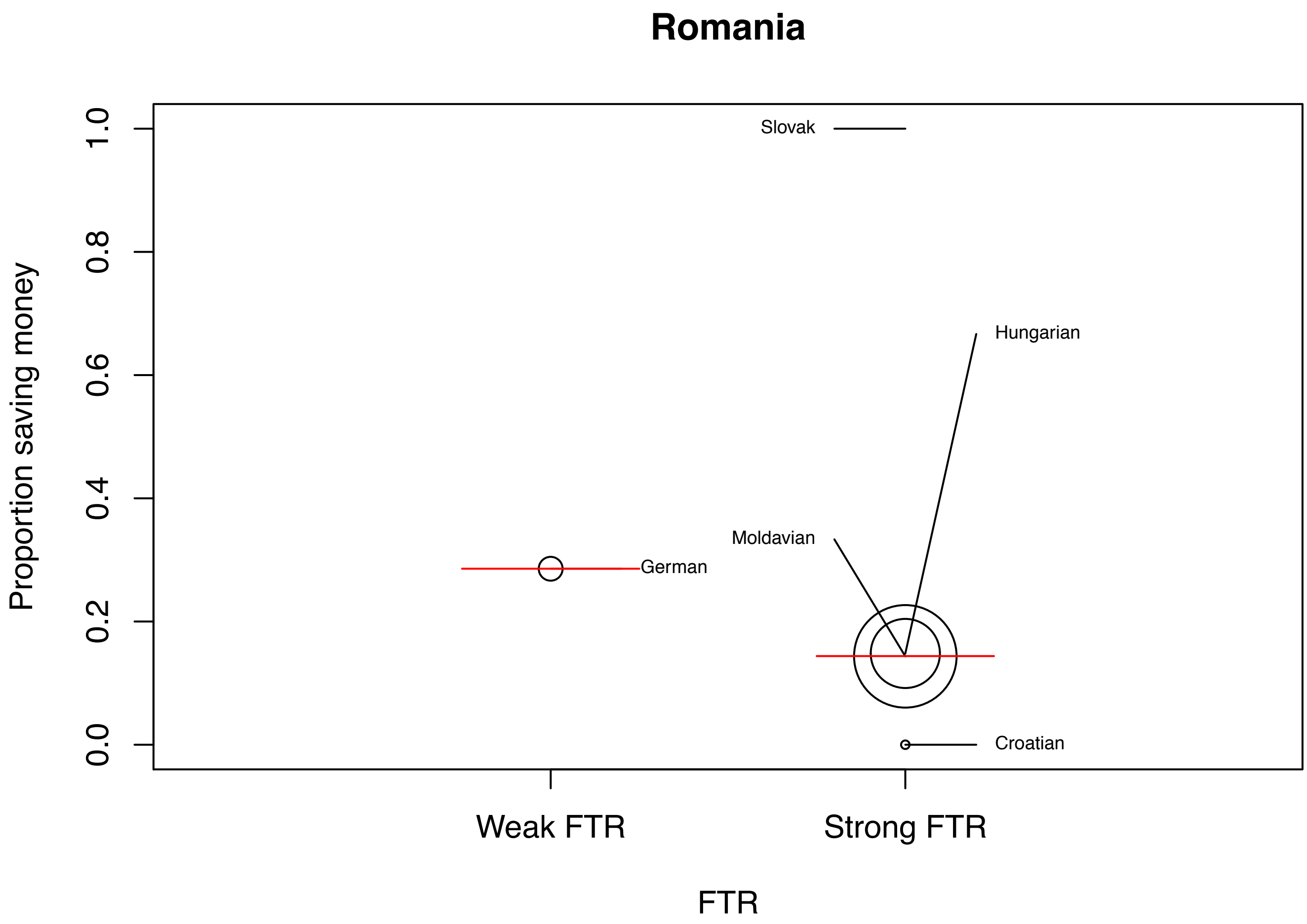
Romania
Proportion saving money
0.0
0.2
0.4
0.6
0.8
1.0
Slovak
Hungarian
Moldavian
German
Croatian
Weak FTR
Strong FTR
FTR

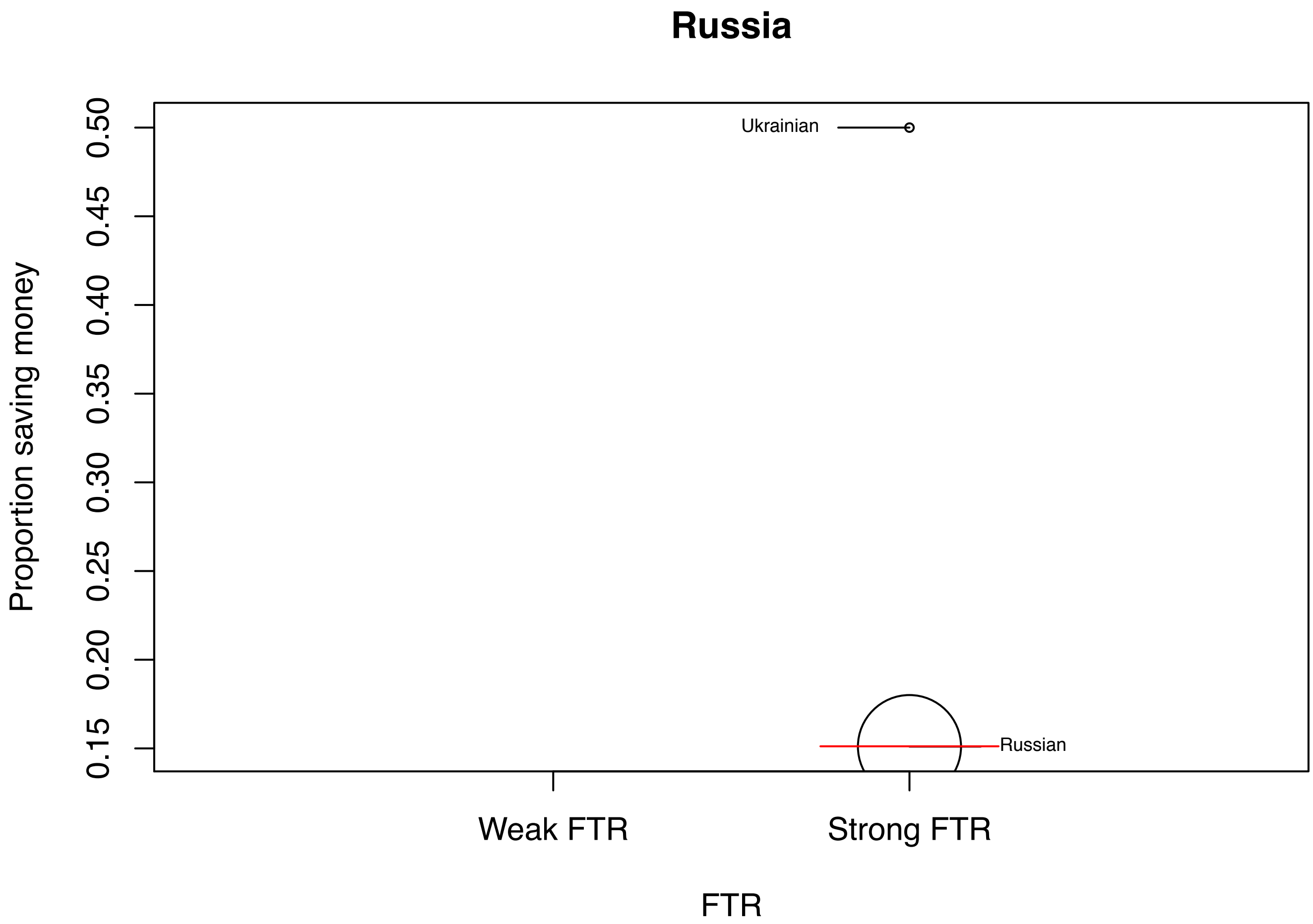
Russia
Proportion saving money
0.15
0.20
0.25
0.30
0.35
0.40
0.45
0.50
Ukrainian
Russian
Weak FTR
Strong FTR
FTR

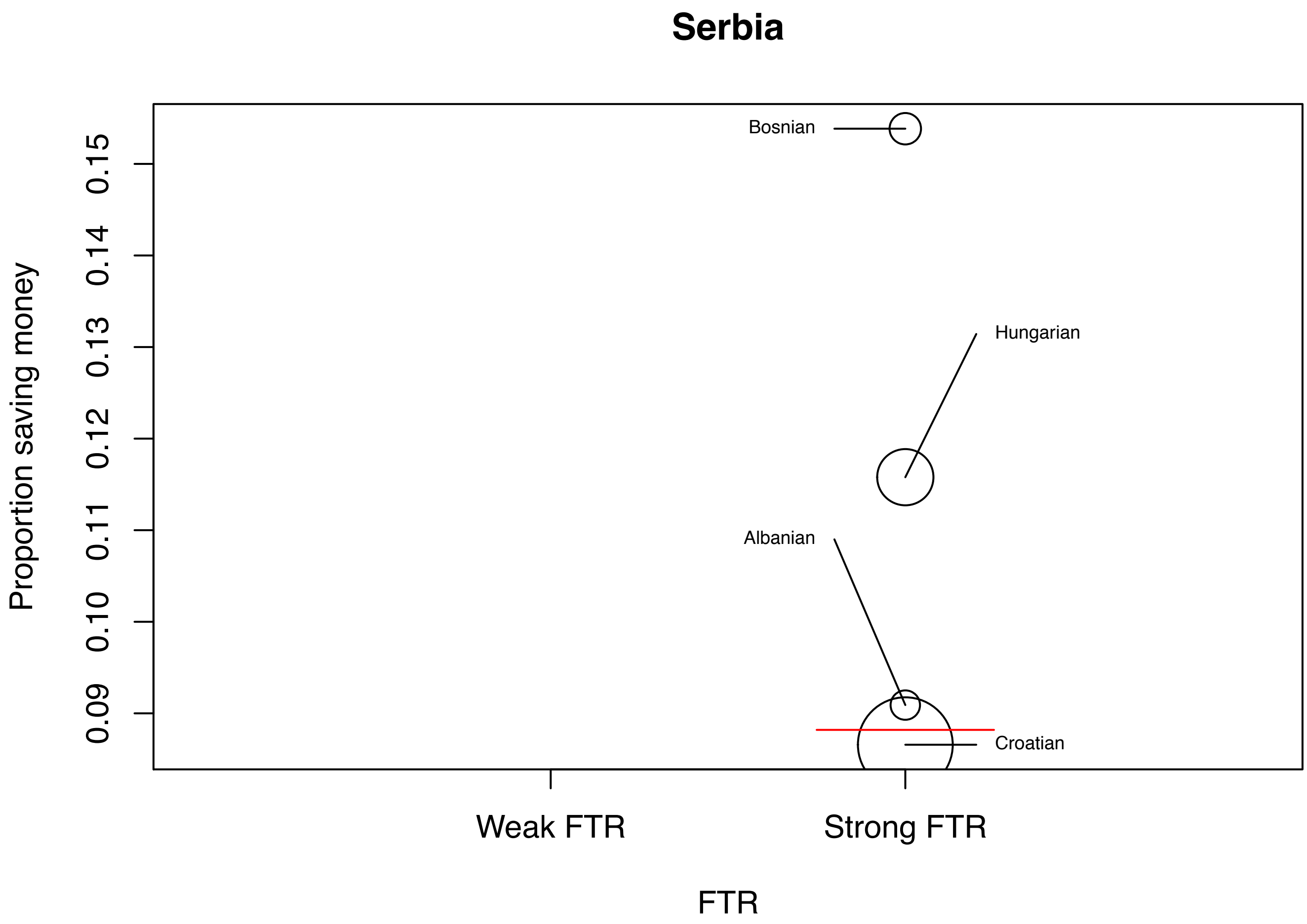

Serbia
Proportion saving money
0.09
0.10
0.11
0.12
0.13
0.14
0.15
Bosnian
Hungarian
Albanian
Croatian
Weak FTR
Strong FTR
FTR

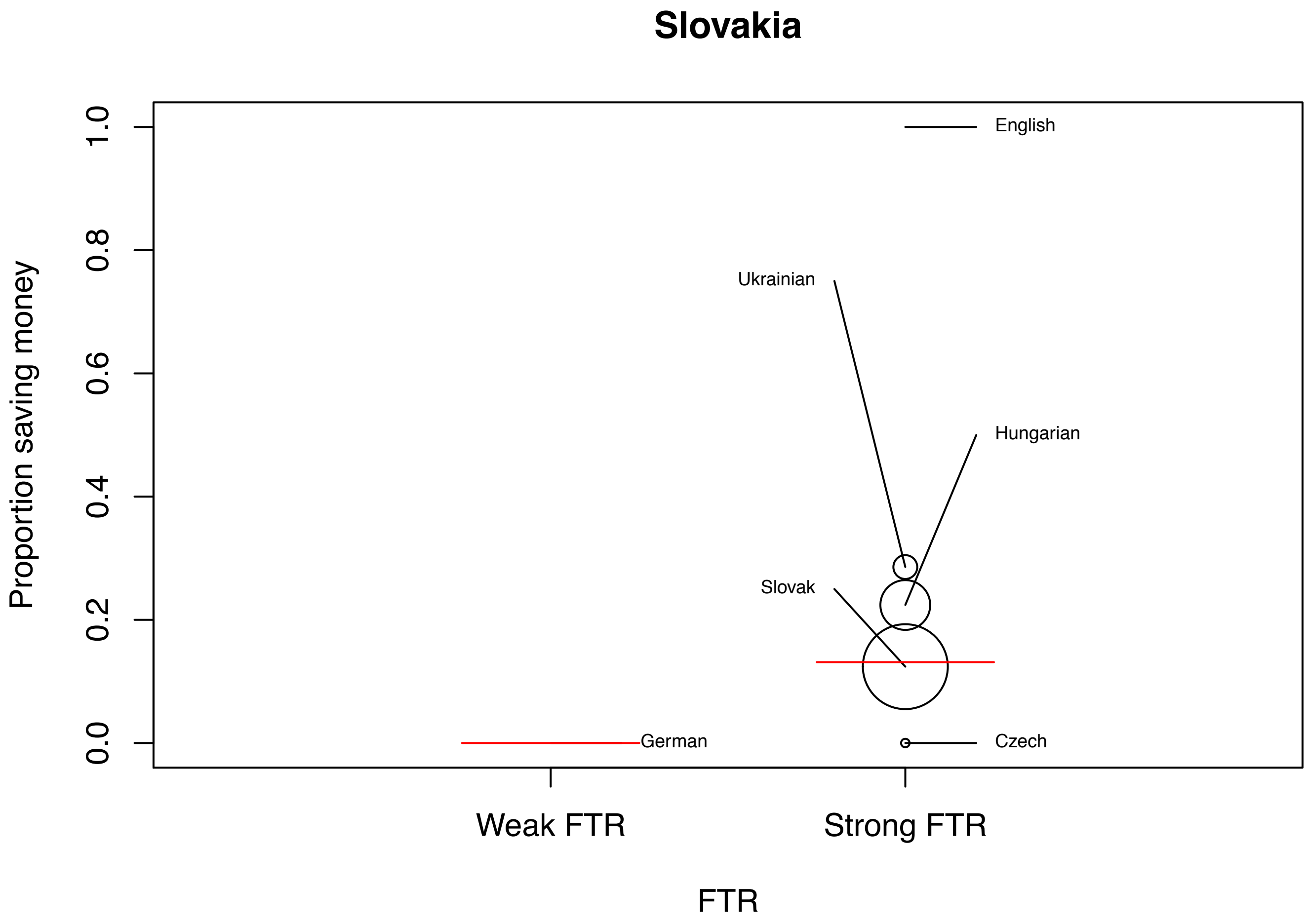

Slovakia
Proportion saving money
0.0
0.2
0.4
0.6
0.8
1.0
English
Ukrainian
Hungarian
Slovak
German
Czech
Weak FTR
Strong FTR
FTR

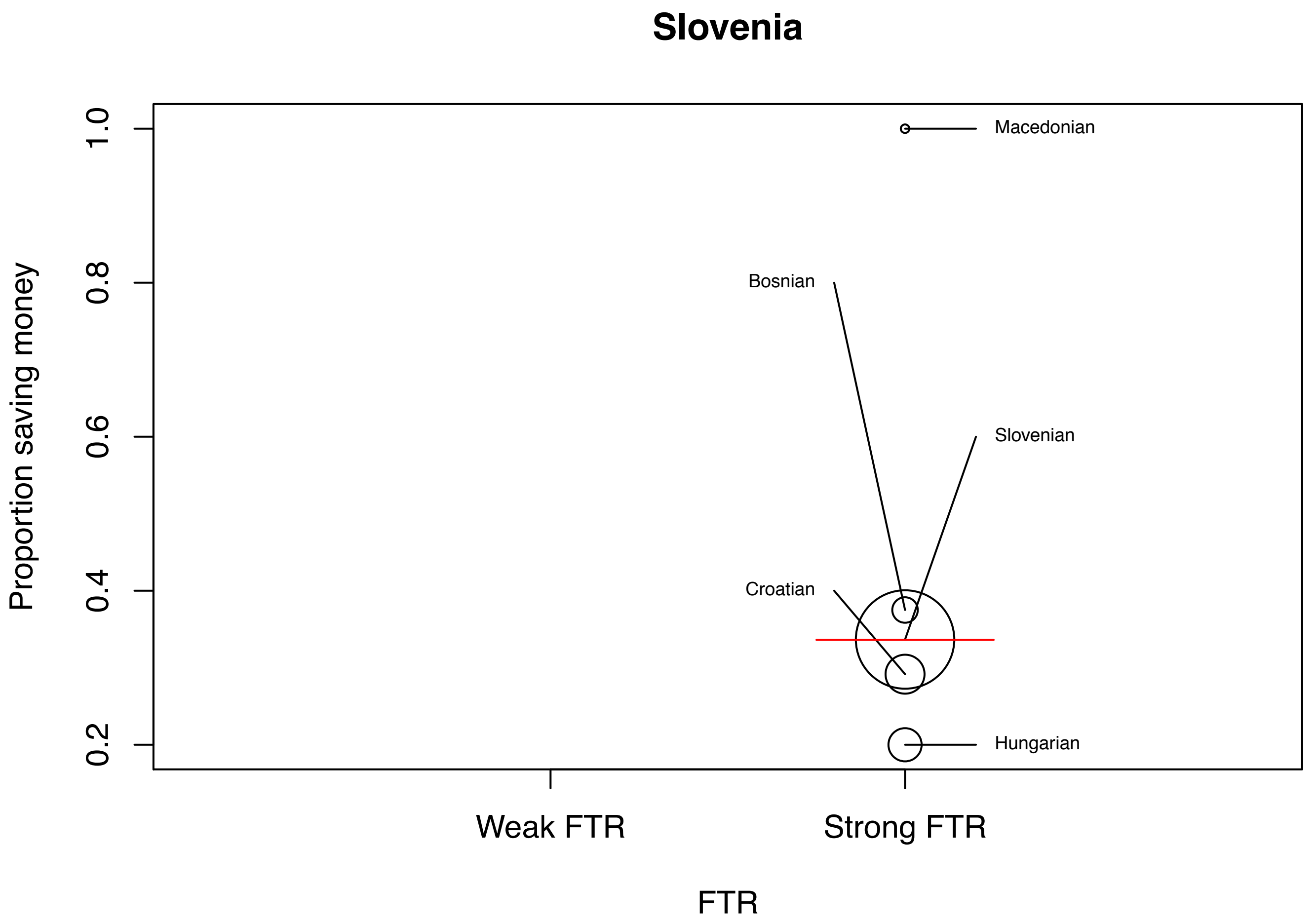
Slovenia
Proportion saving money
1.0
0.8
0.6
0.4
0.2
Macedonian
Bosnian
Slovenian
Croatian
Hungarian
Weak FTR
Strong FTR
FTR

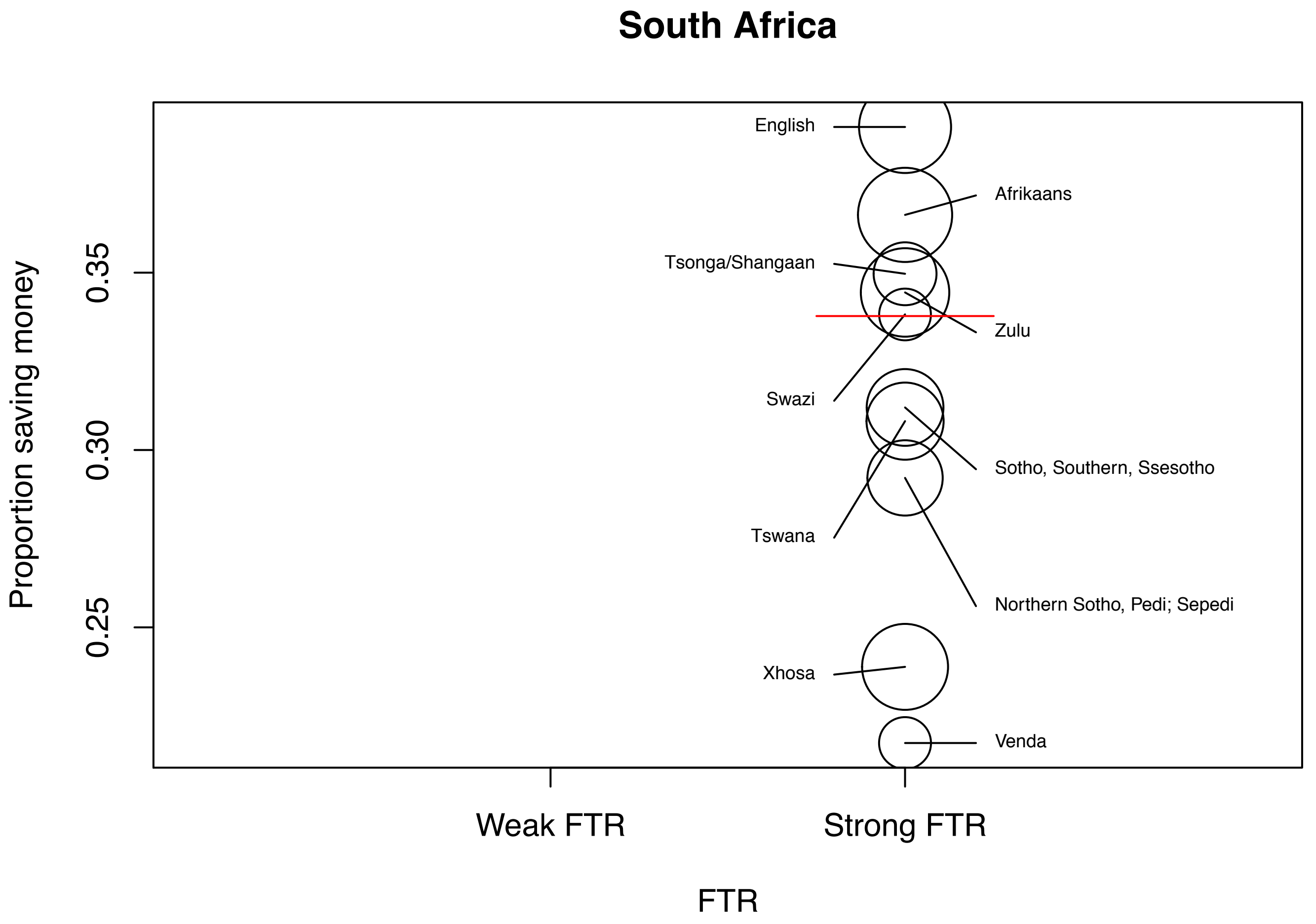
South Africa
Proportion saving money
0.35
0.30
0.25
English
Afrikaans
Tsonga/Shangaan
Zulu
Swazi
Sotho, Southern, Ssesotho
Tswana
Northern Sotho, Pedi; Sepedi
Xhosa
Venda
Weak FTR
Strong FTR
FTR

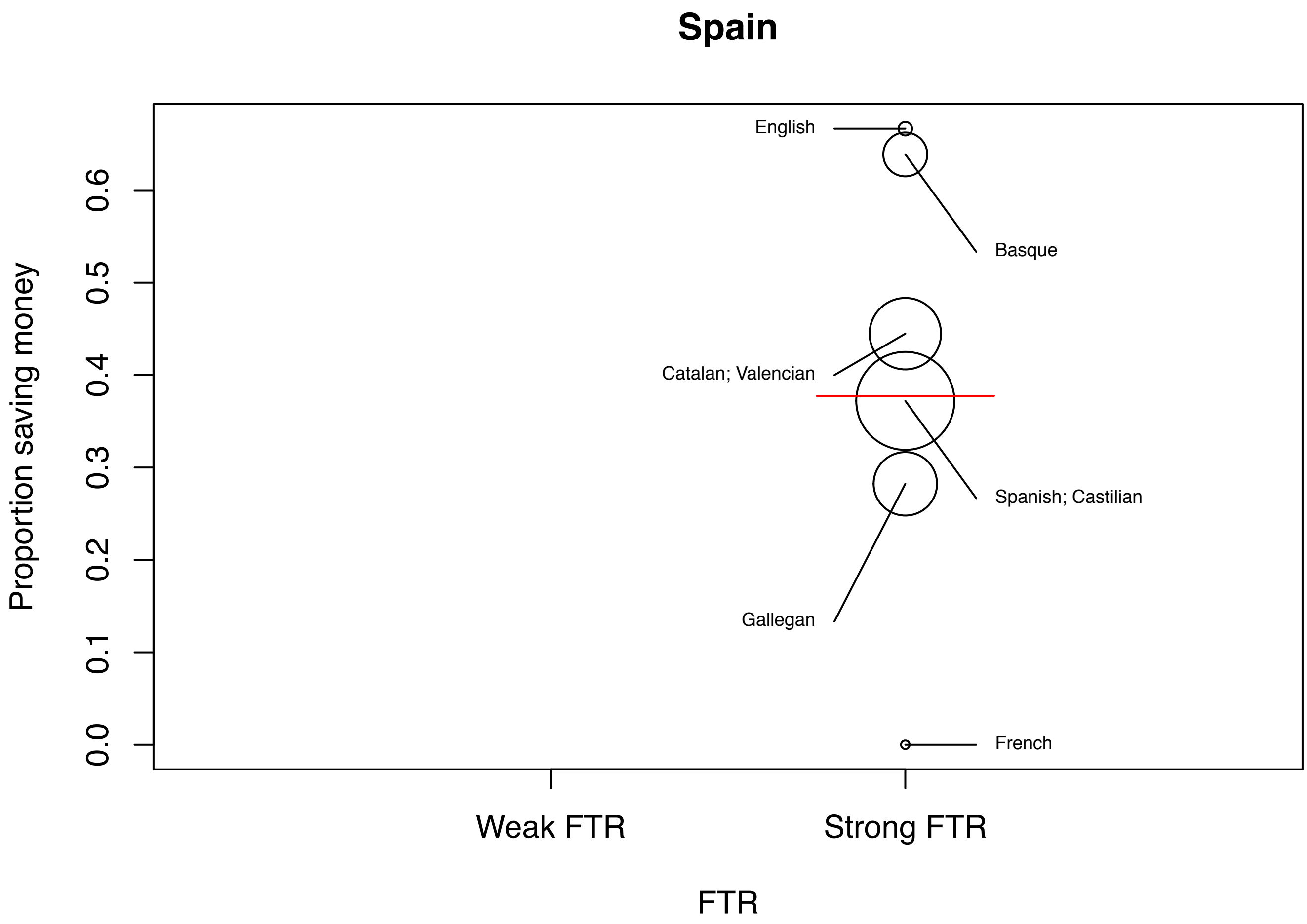
Spain
Proportion saving money
0.0
0.1
0.2
0.3
0.4
0.5
0.6
English
Basque
Catalan; Valencian
Spanish; Castilian
Gallegan
French
Weak FTR
Strong FTR
FTR

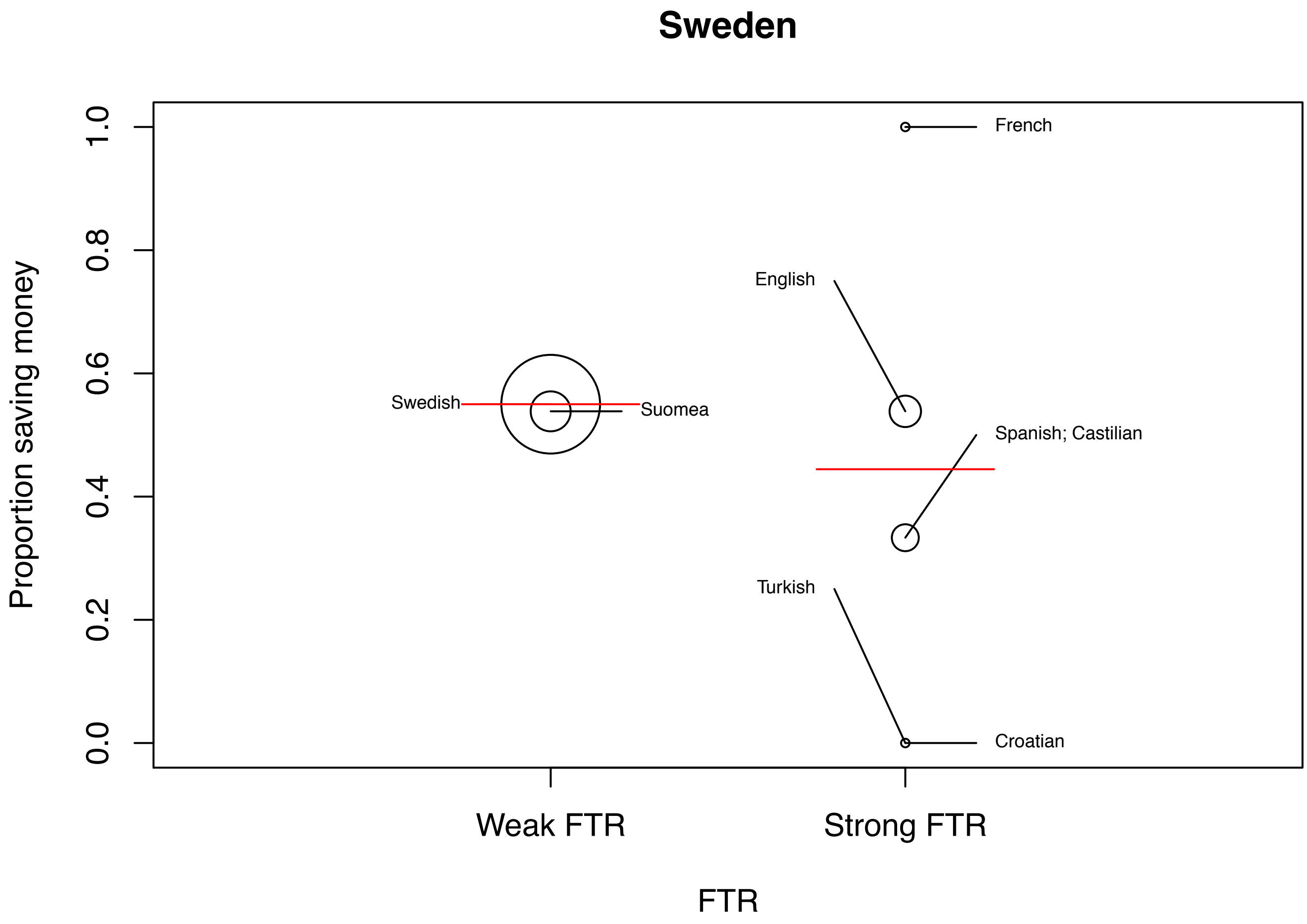
Sweden
Proportion saving money
0.0
0.2
0.4
0.6
0.8
1.0
French
English
Swedish
Suomea
Spanish; Castilian
Turkish
Croatian
Weak FTR
Strong FTR
FTR

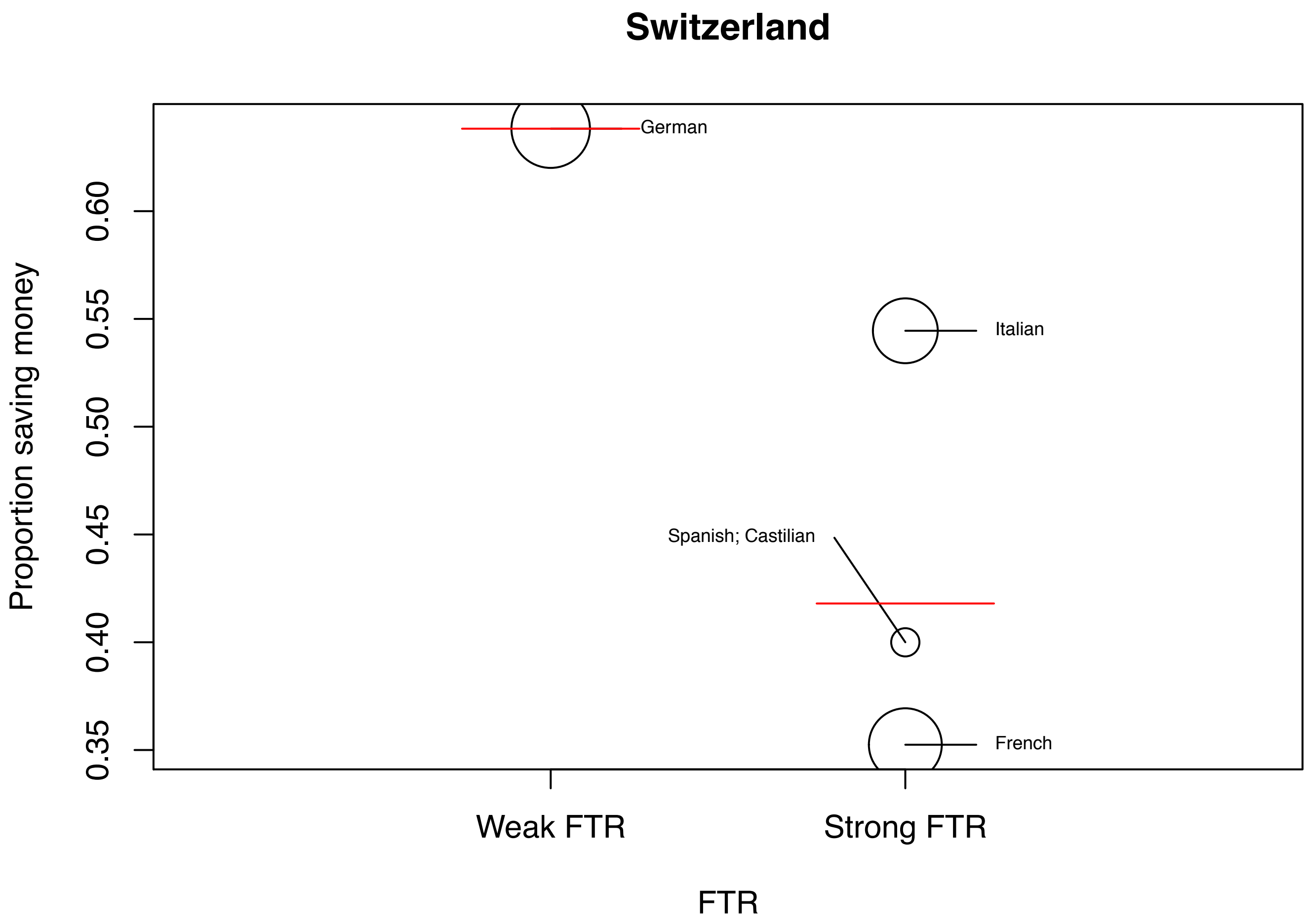
Switzerland
Proportion saving money
0.35
0.40
0.45
0.50
0.55
0.60
German
Italian
Spanish; Castilian
French
Weak FTR
Strong FTR
FTR

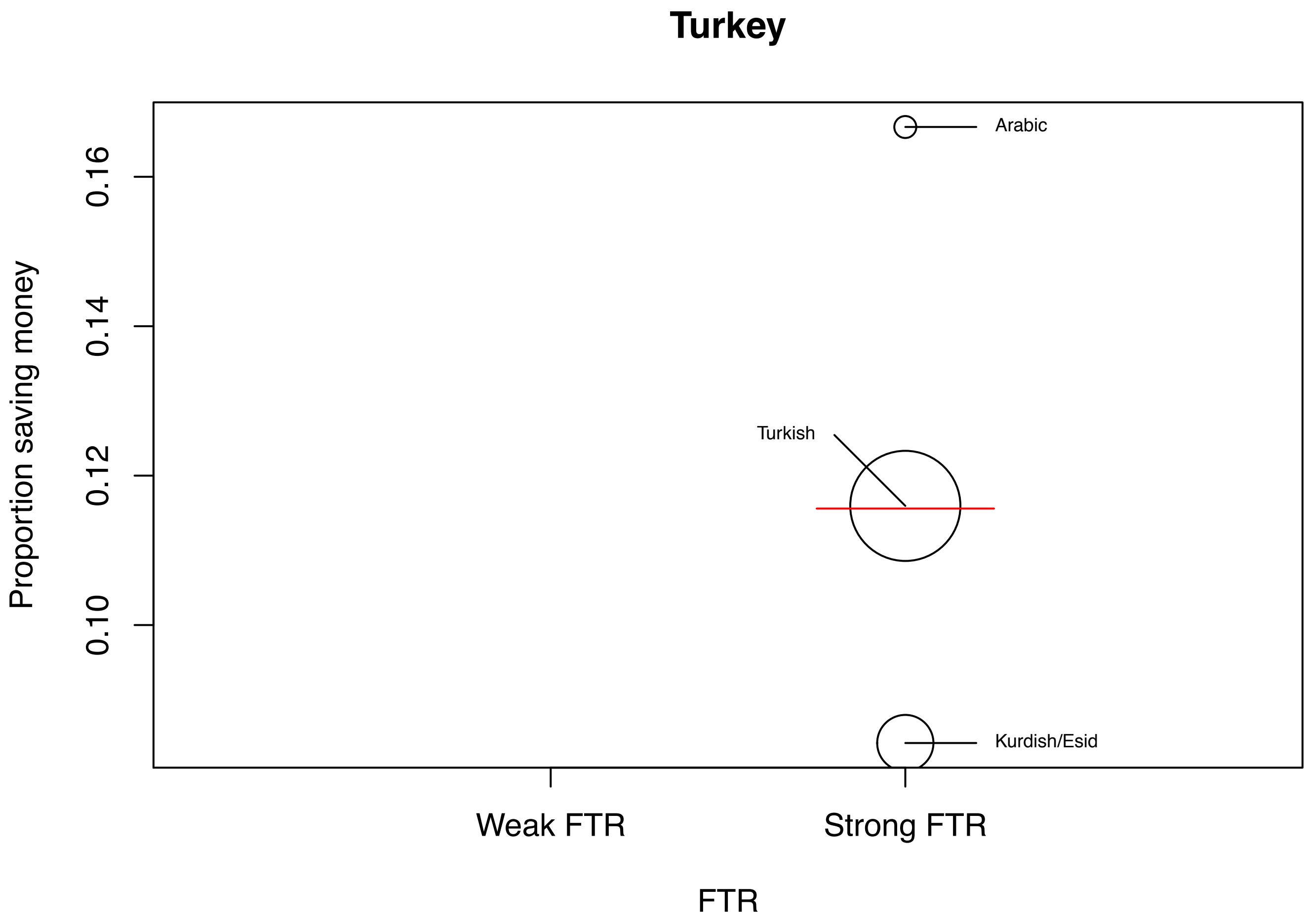
Turkey
Proportion saving money
0.10
0.12
0.14
0.16
Arabic
Turkish
Kurdish/Esid
Weak FTR
Strong FTR
FTR

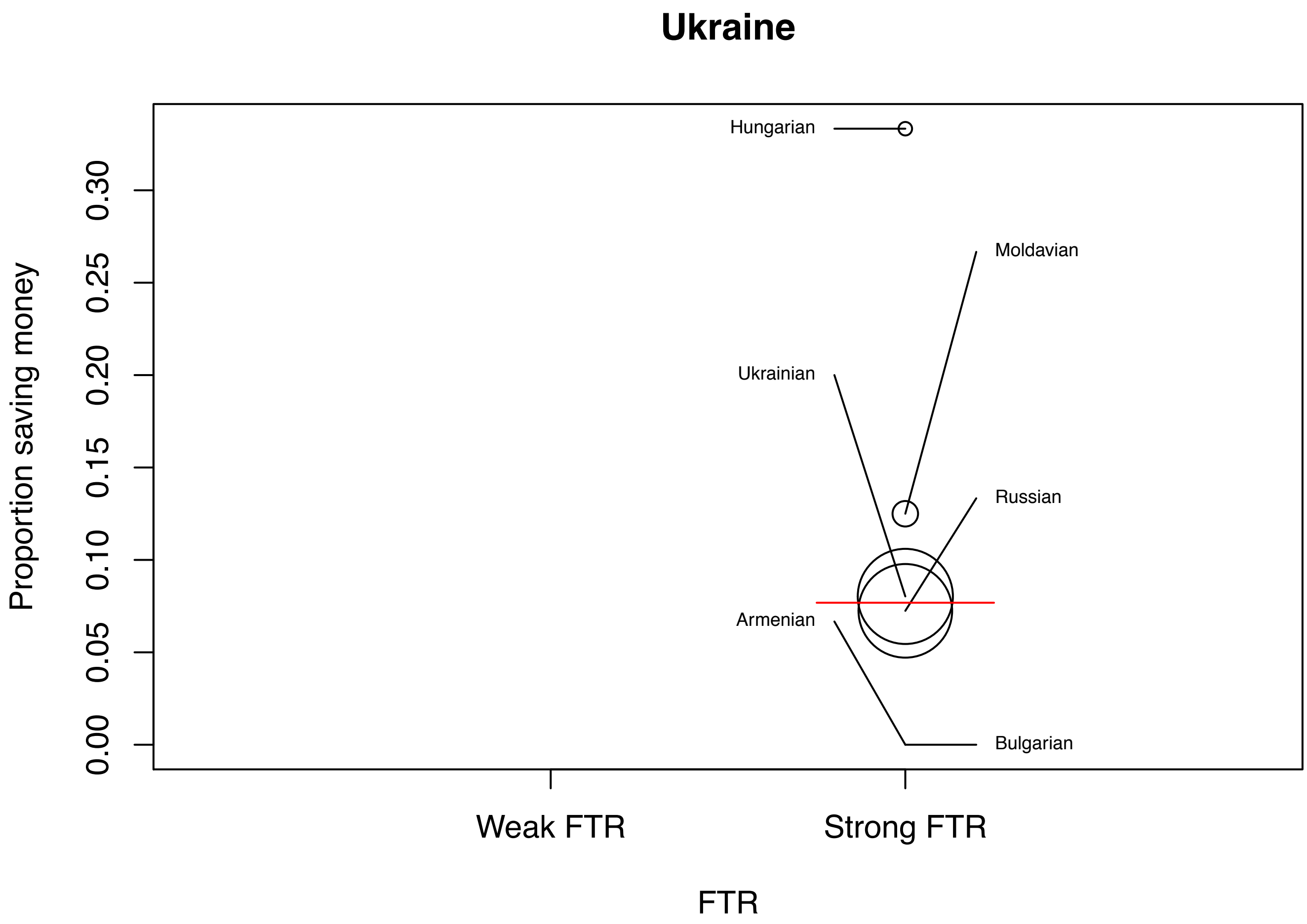
Ukraine
Proportion saving money
0.00
0.05
0.10
0.15
0.20
0.25
0.30
Hungarian
Moldavian
Ukrainian
Russian
Armenian
Bulgarian
Weak FTR
Strong FTR
FTR

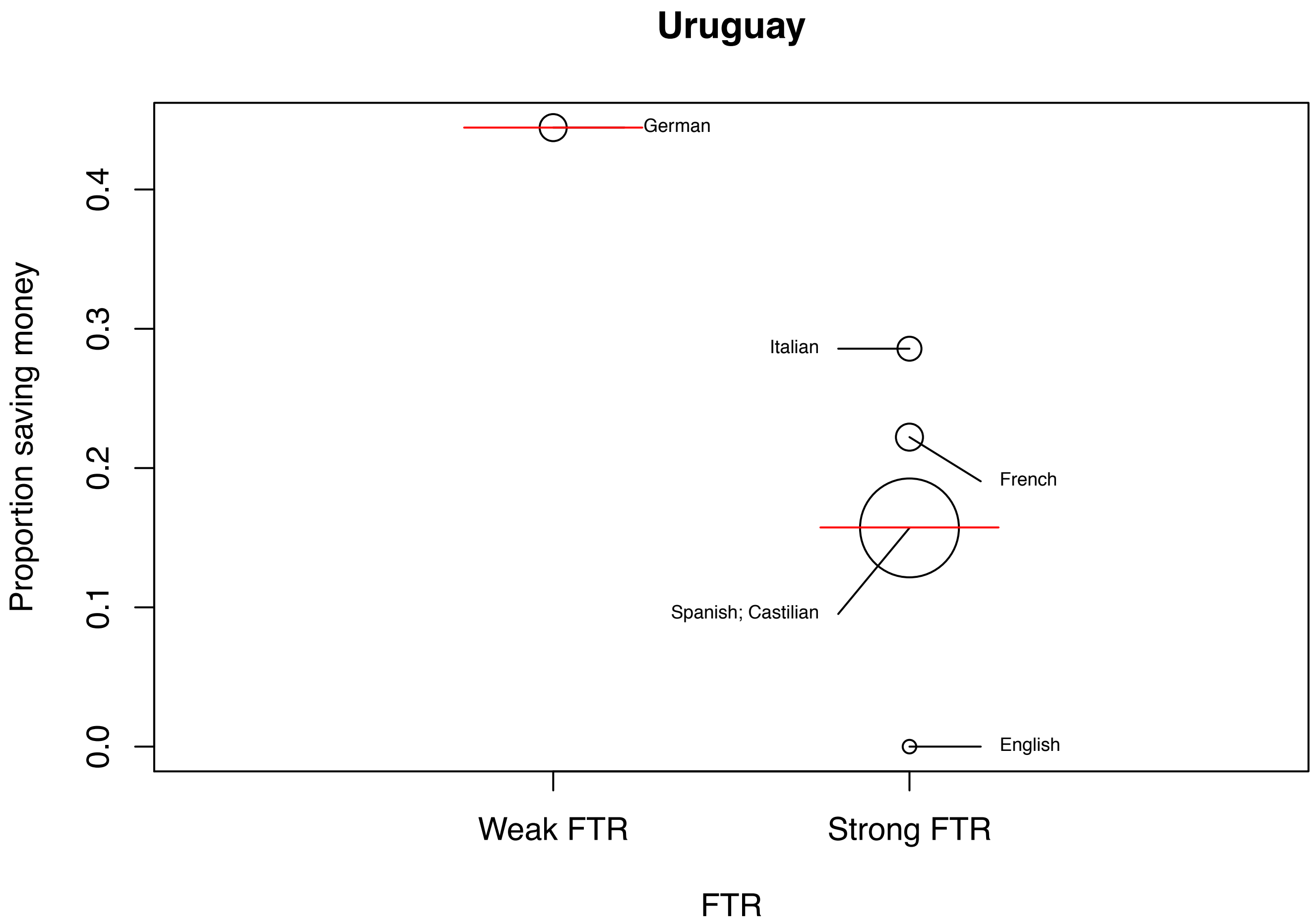
Uruguay
Proportion saving money
0.0
0.1
0.2
0.3
0.4
German
Italian
French
Spanish; Castilian
English
Weak FTR
Strong FTR
FTR

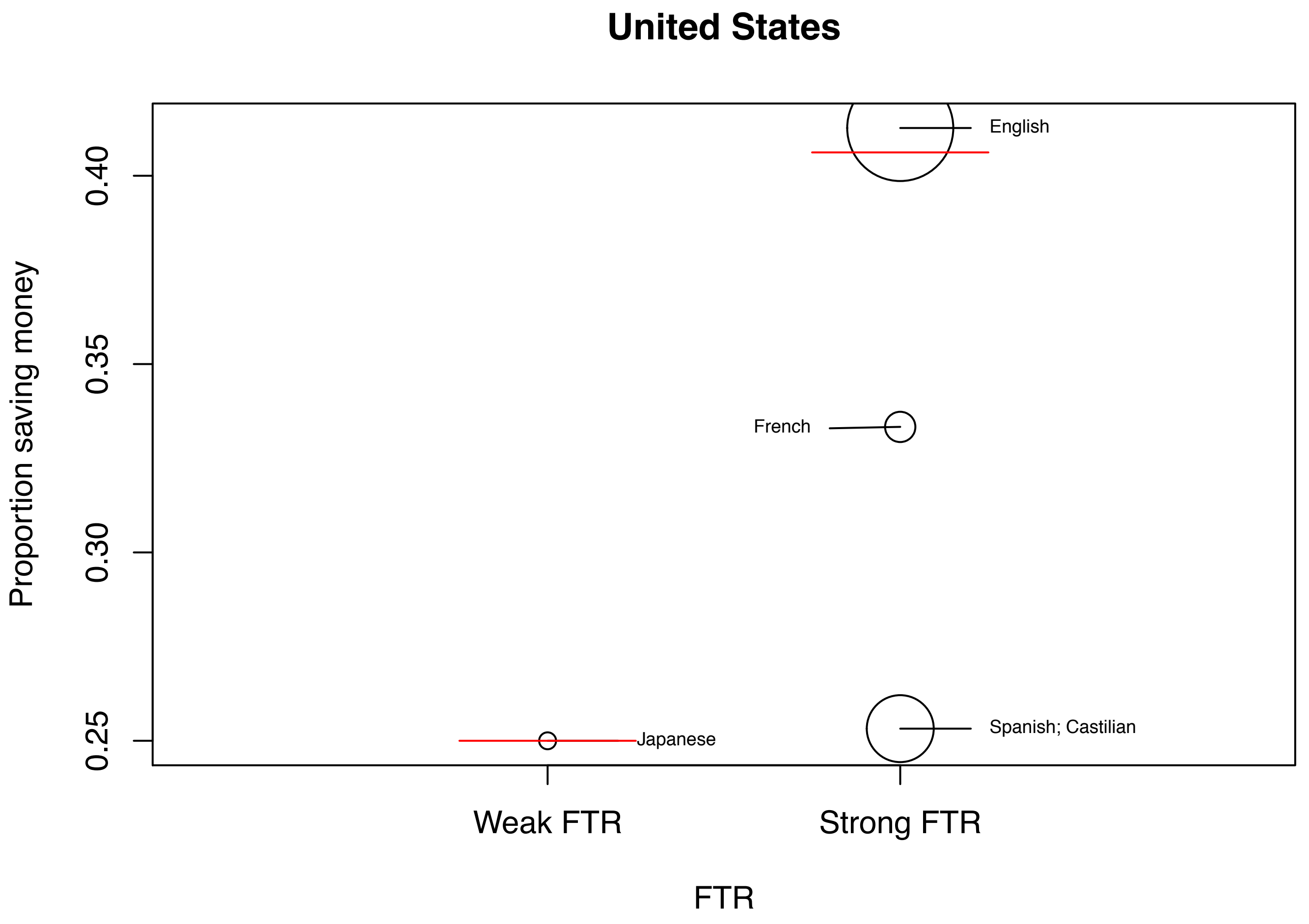
United States
Proportion saving money
0.40
0.35
0.30
0.25
English
French
Japanese
Spanish; Castilian
Weak FTR
Strong FTR
FTR

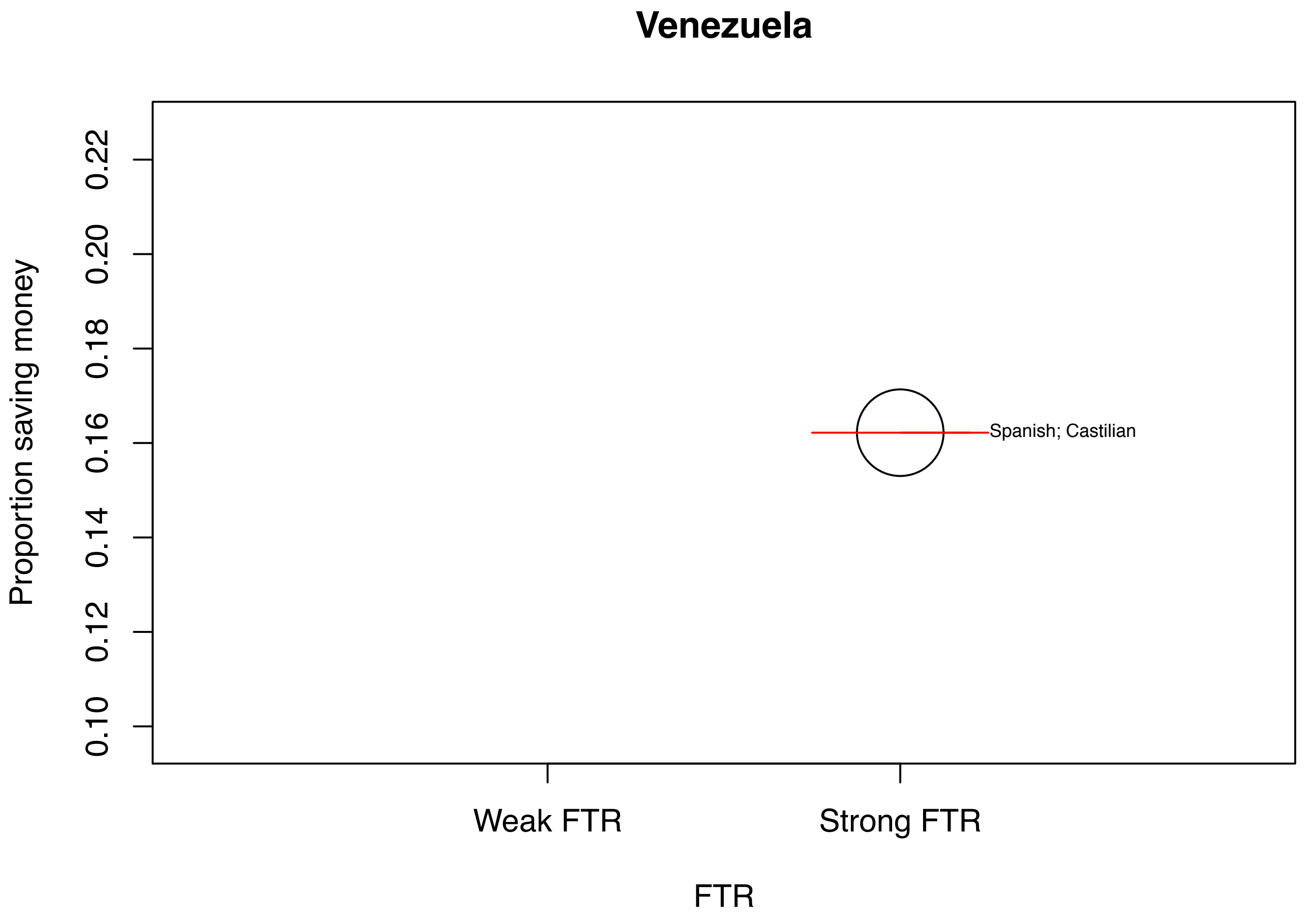
Venezuela
Proportion saving money
0.22
0.20
0.18
0.16
0.14
0.12
0.10
Spanish; Castilian
Weak FTR
Strong FTR
FTR

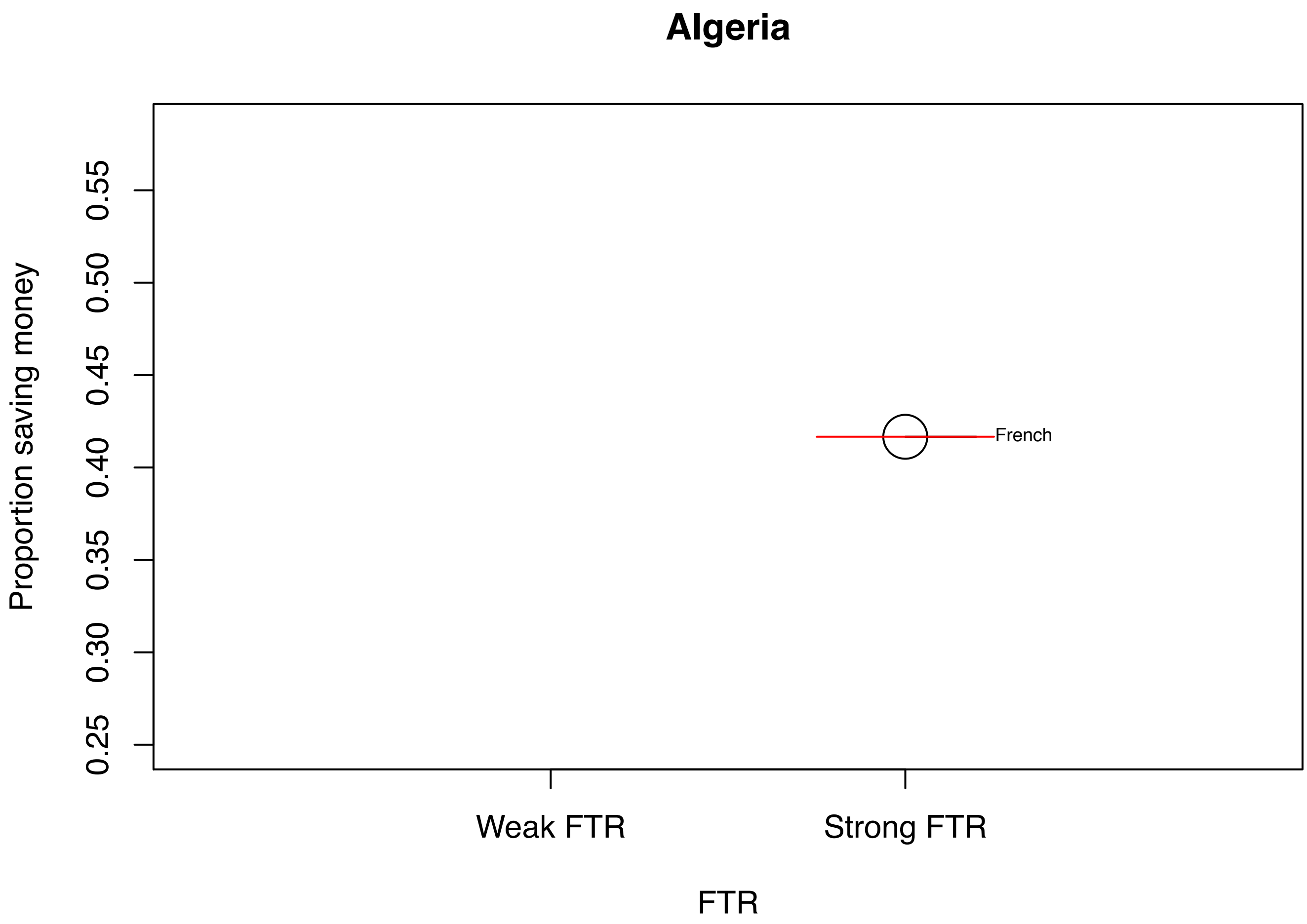
Algeria
Proportion saving money
0.25
0.30
0.35
0.40
0.45
0.50
0.55
French
Weak FTR
Strong FTR
FTR

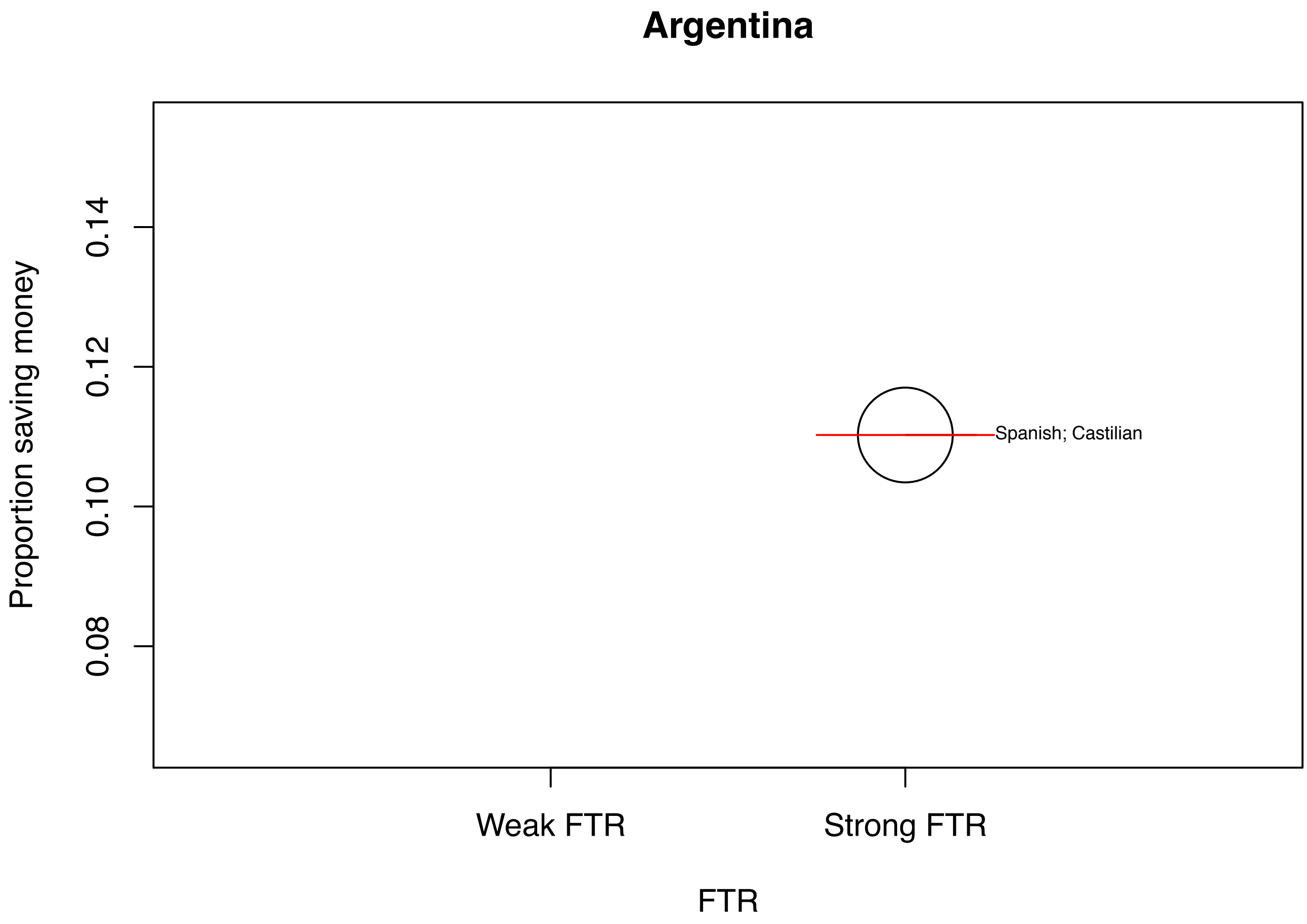
Argentina
Proportion saving money
0.08
0.10
0.12
0.14
Spanish; Castilian
Weak FTR
Strong FTR
FTR

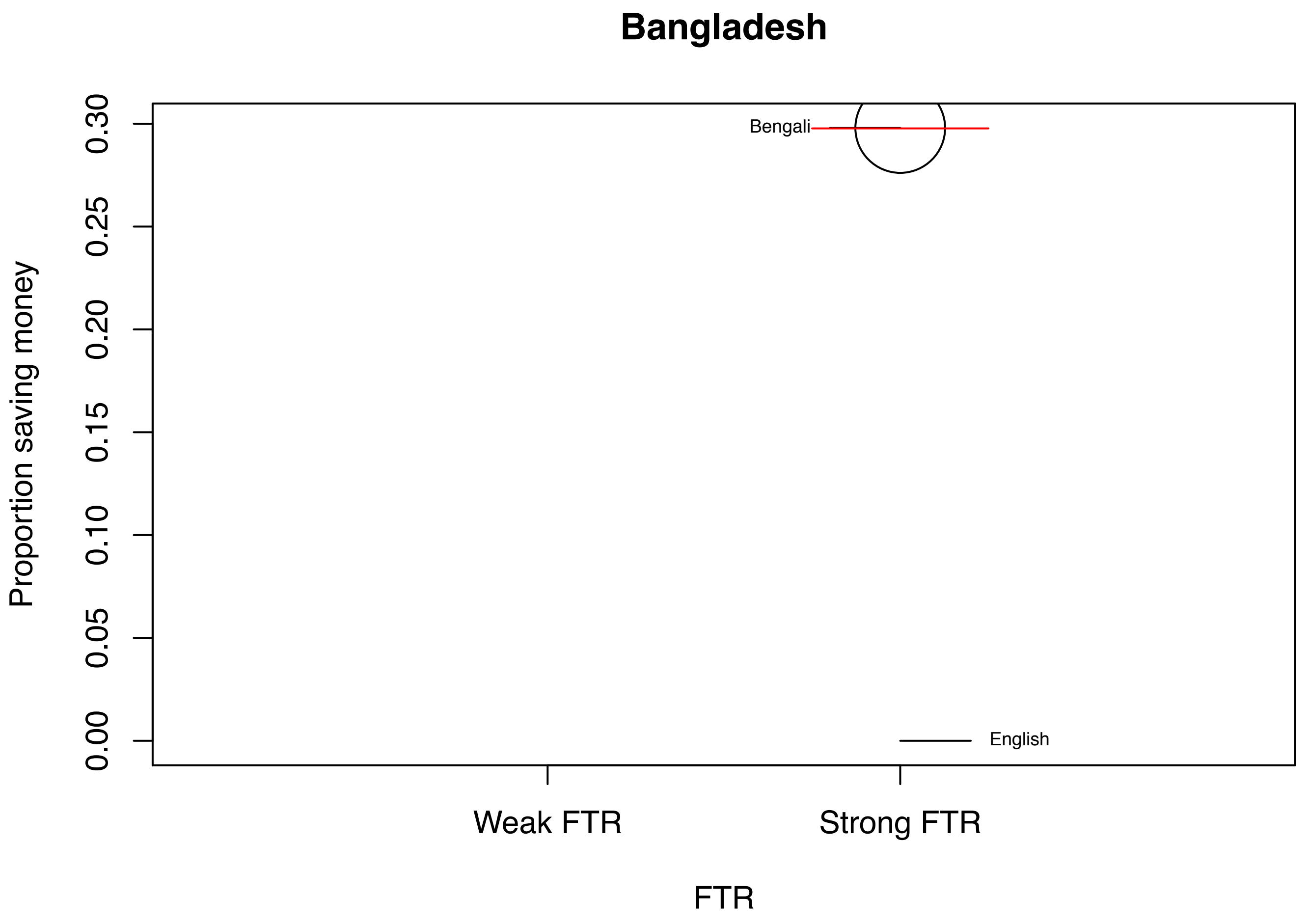

Bangladesh
Proportion saving money
0.00
0.05
0.10
0.15
0.20
0.25
0.30
Bengali
English
Weak FTR
Strong FTR
FTR

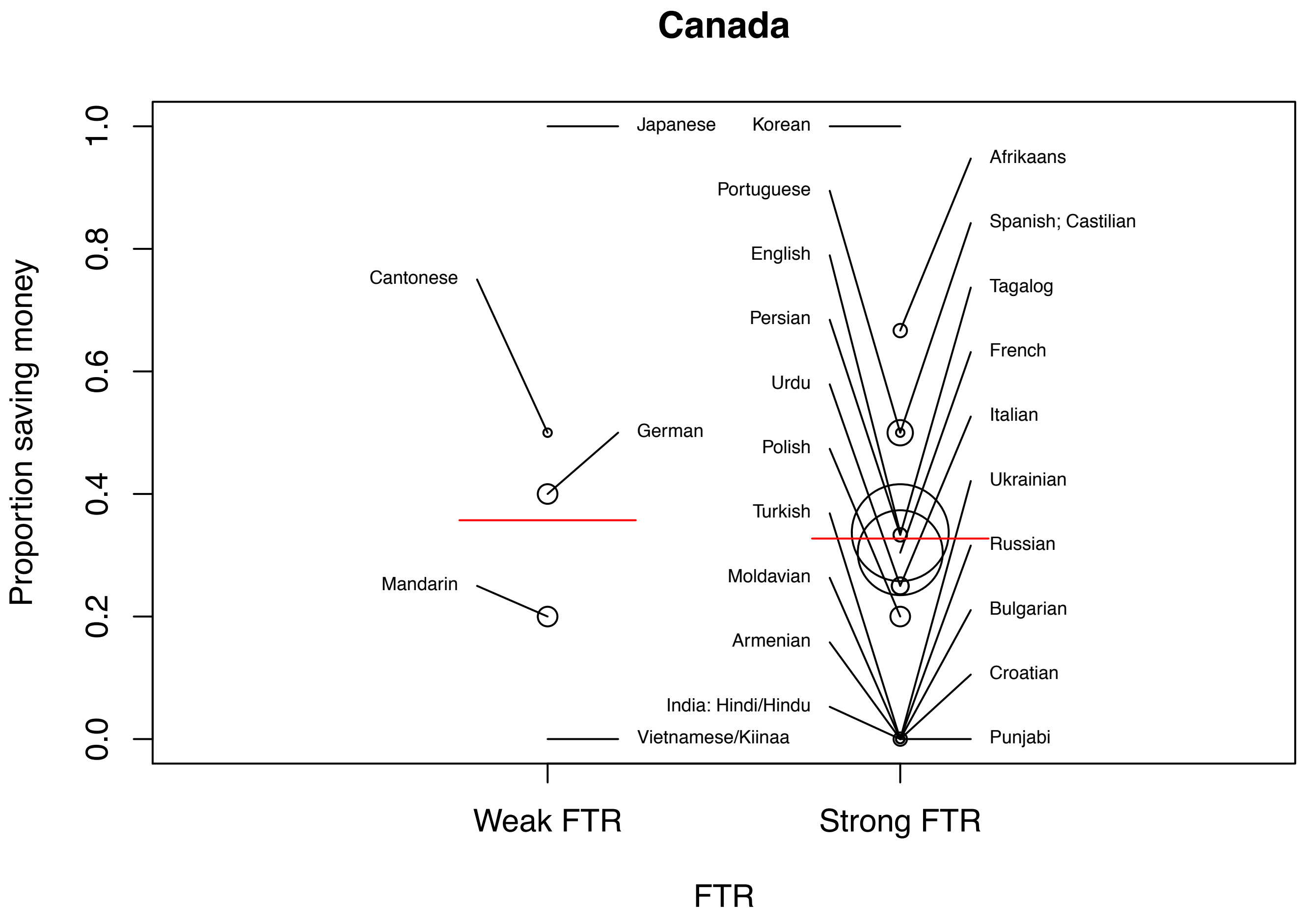

Canada
Proportion saving money
0.0
0.2
0.4
0.6
0.8
1.0
Japanese
Korean
Afrikaans
Portuguese
Spanish; Castilian
English
Cantonese
Tagalog
Persian
French
Urdu
Italian
German
Polish
Ukrainian
Turkish
Russian
Mandarin
Moldavian
Bulgarian
Armenian
Croatian
India: Hindi/Hindu
Vietnamese/Kiinaa
Punjabi
Weak FTR
Strong FTR
FTR

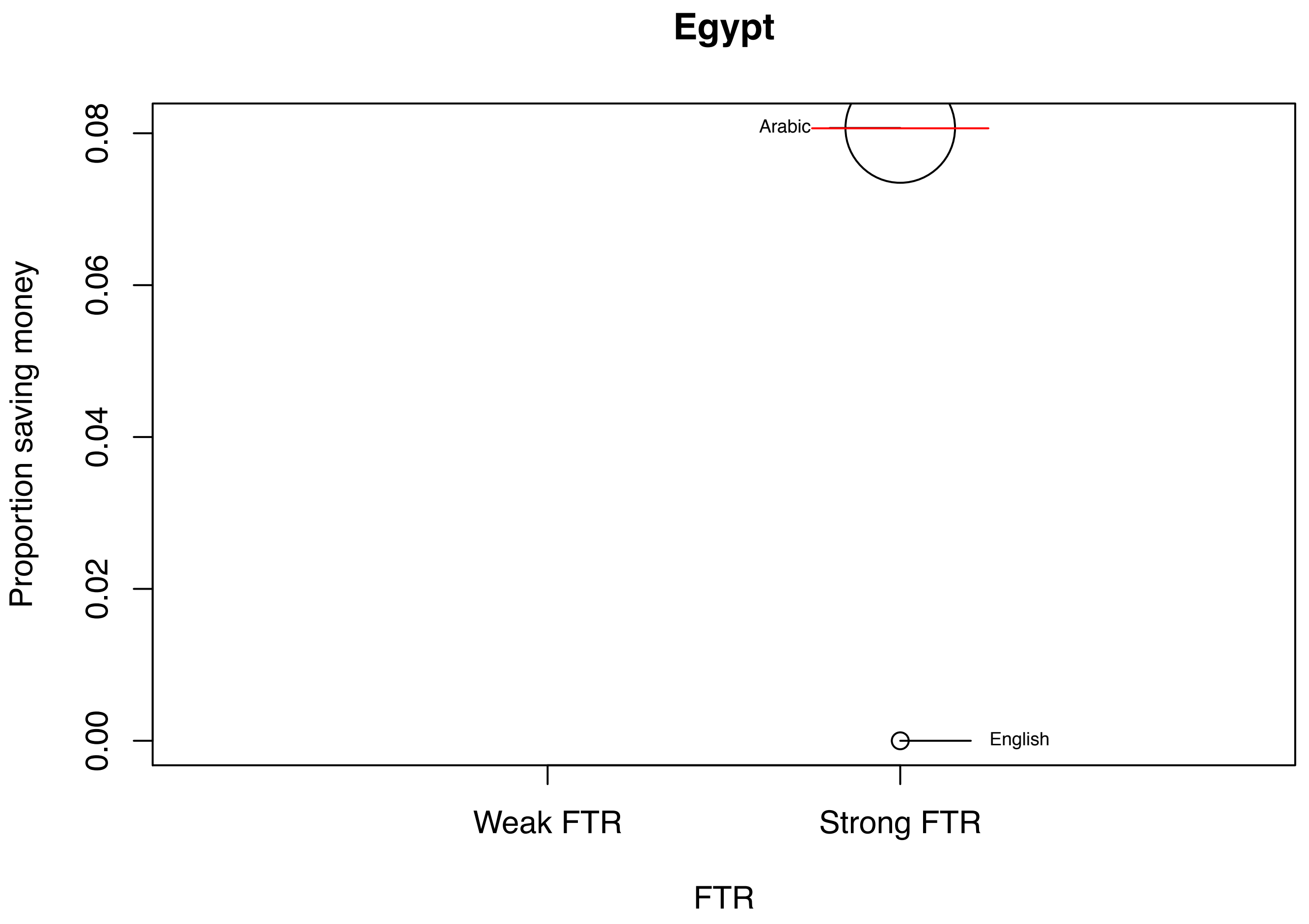
Egypt
Arabic
English
0.00
0.02
0.04
0.06
0.08
Proportion saving money
Weak FTR
Strong FTR
FTR

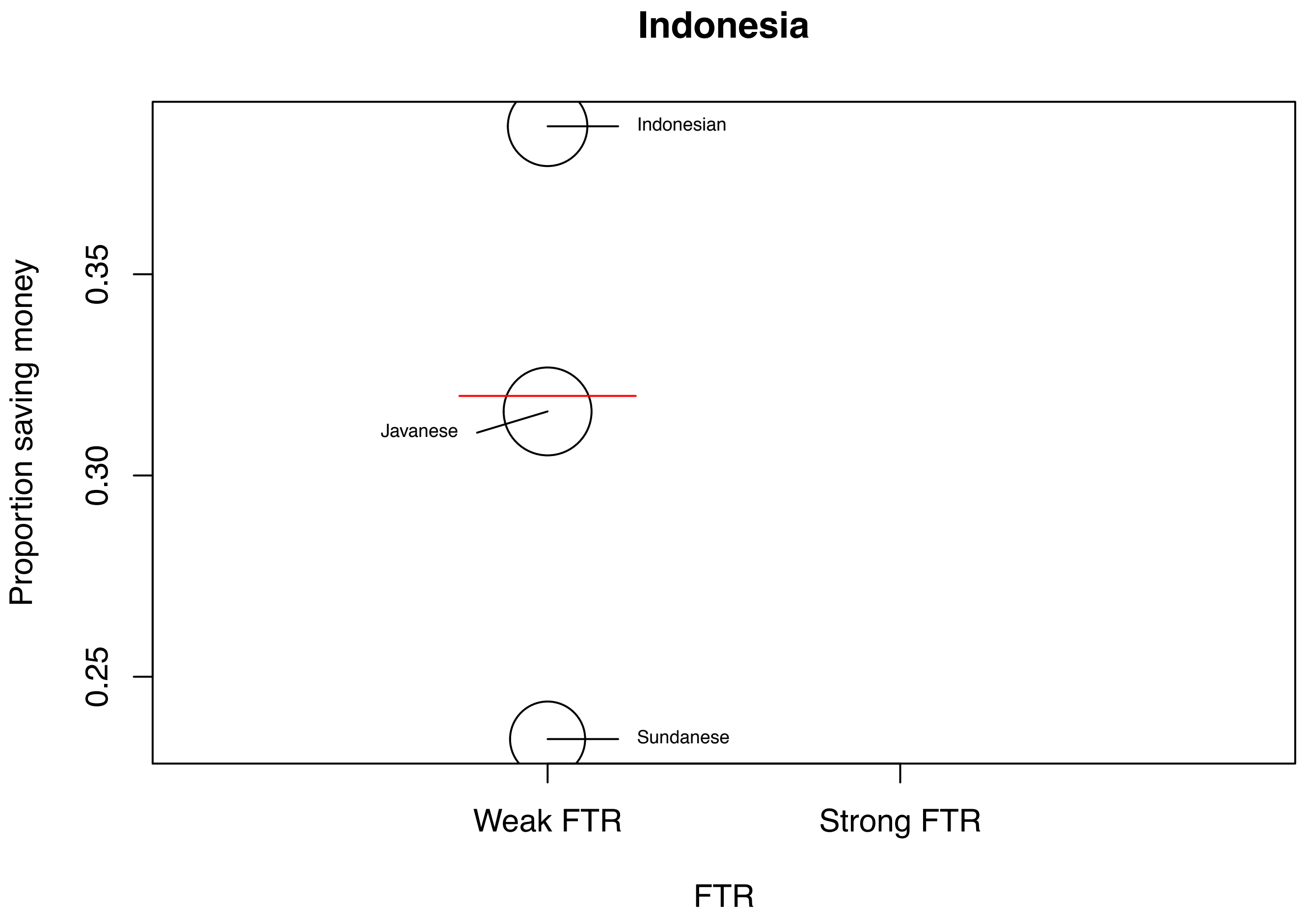
Indonesia
Indonesian
Javanese
Sundanese
0.25
0.30
0.35
Proportion saving money
Weak FTR
Strong FTR
FTR

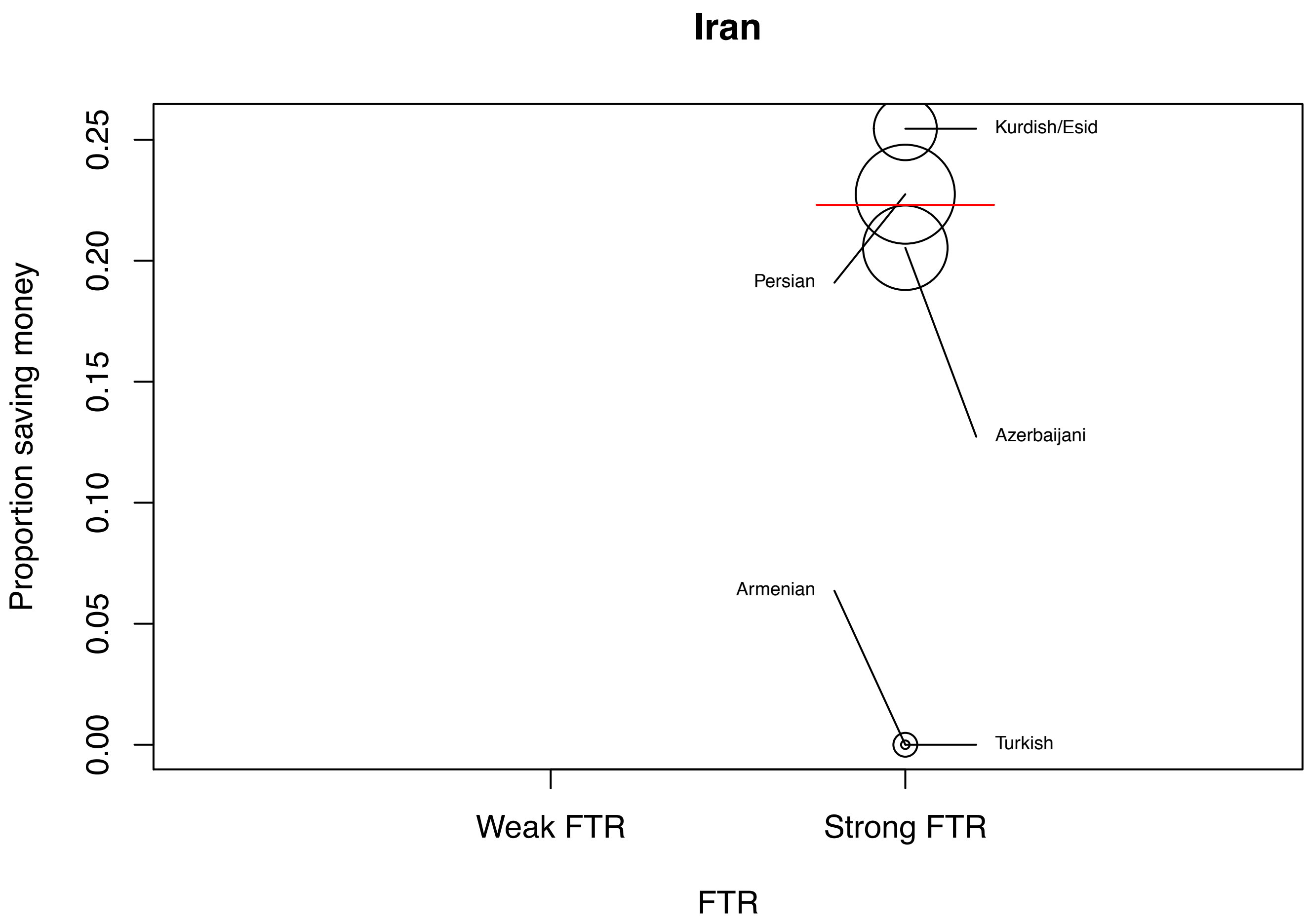
Iran
Proportion saving money
0.00
0.05
0.10
0.15
0.20
0.25
Kurdish/Esid
Persian
Azerbaijani
Armenian
Turkish
Weak FTR
Strong FTR
FTR

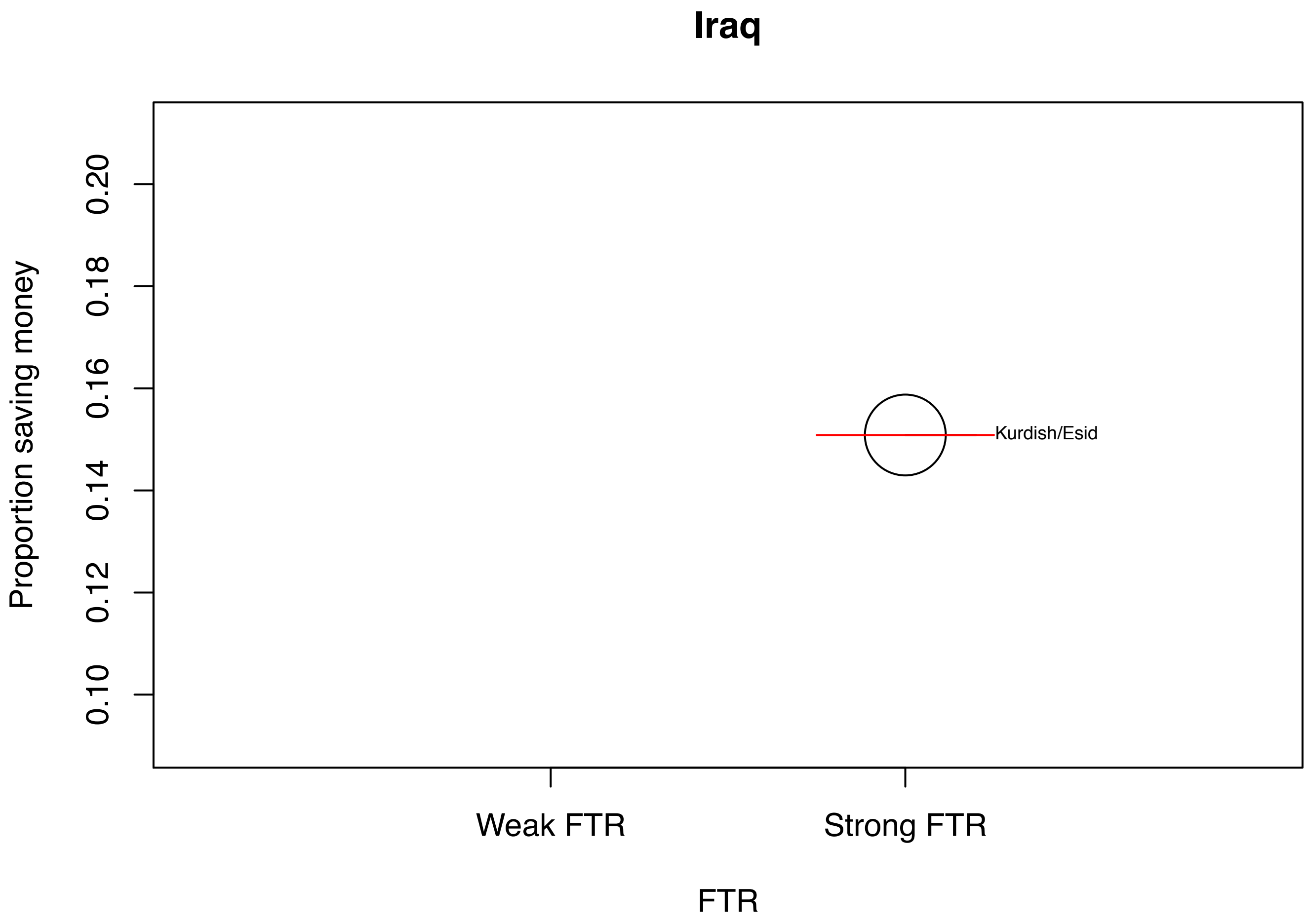
Iraq
Proportion saving money
0.10
0.12
0.14
0.16
0.18
0.20
Kurdish/Esid
Weak FTR
Strong FTR
FTR

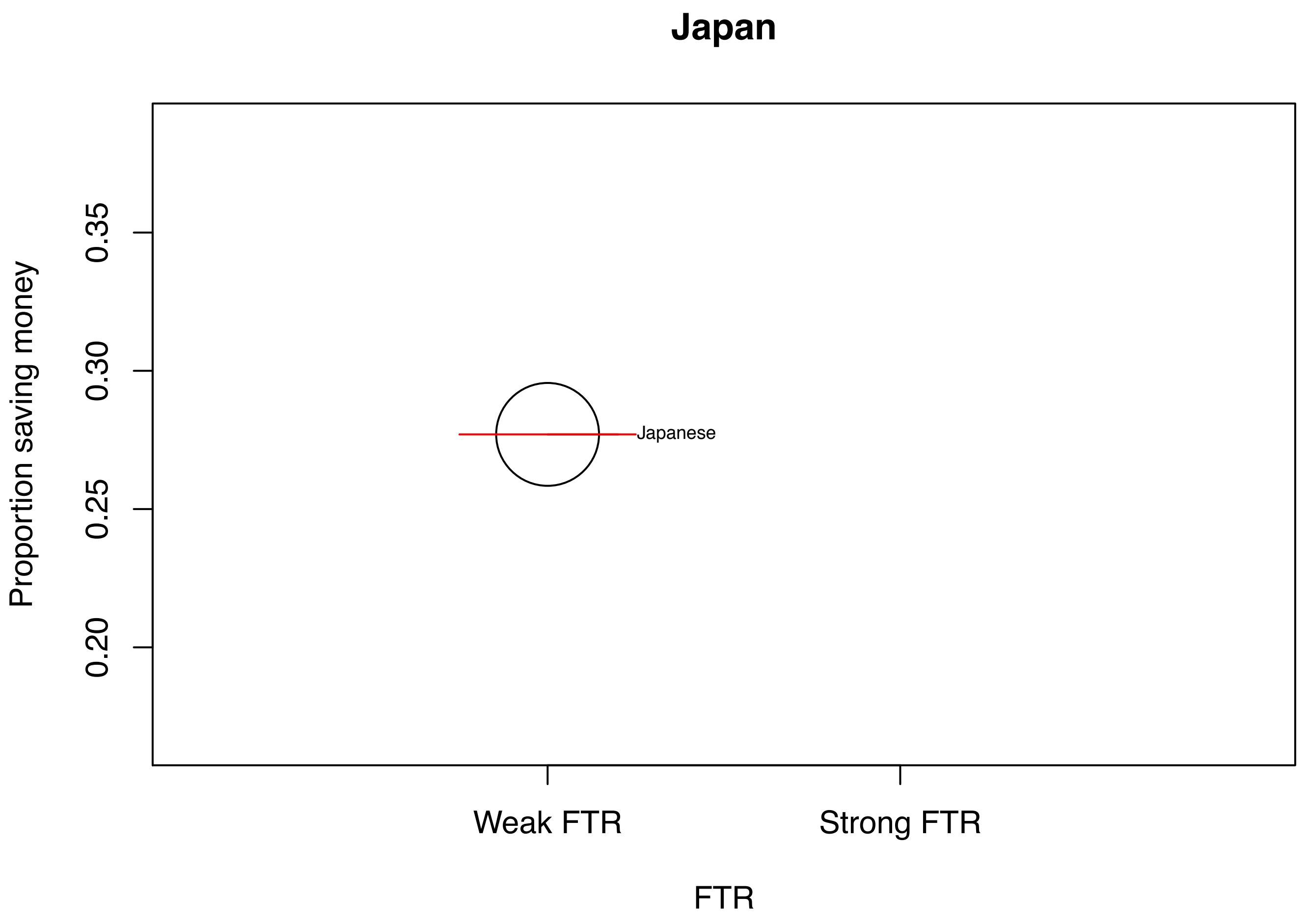
Japan
Proportion saving money
0.20
0.25
0.30
0.35
Japanese
Weak FTR
Strong FTR
FTR

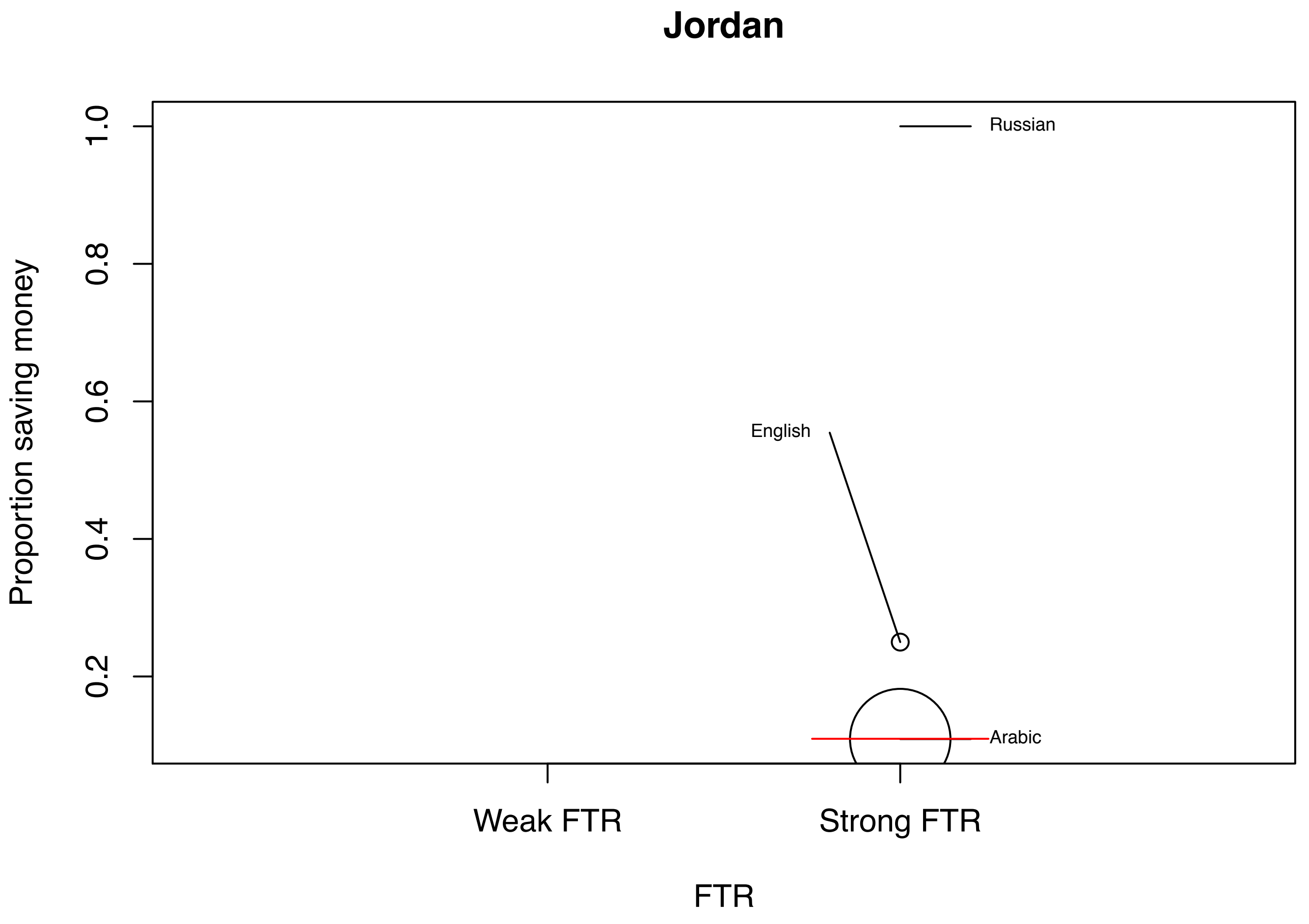
Jordan
Proportion saving money
0.2
0.4
0.6
0.8
1.0
Russian
English
Arabic
Weak FTR
Strong FTR
FTR

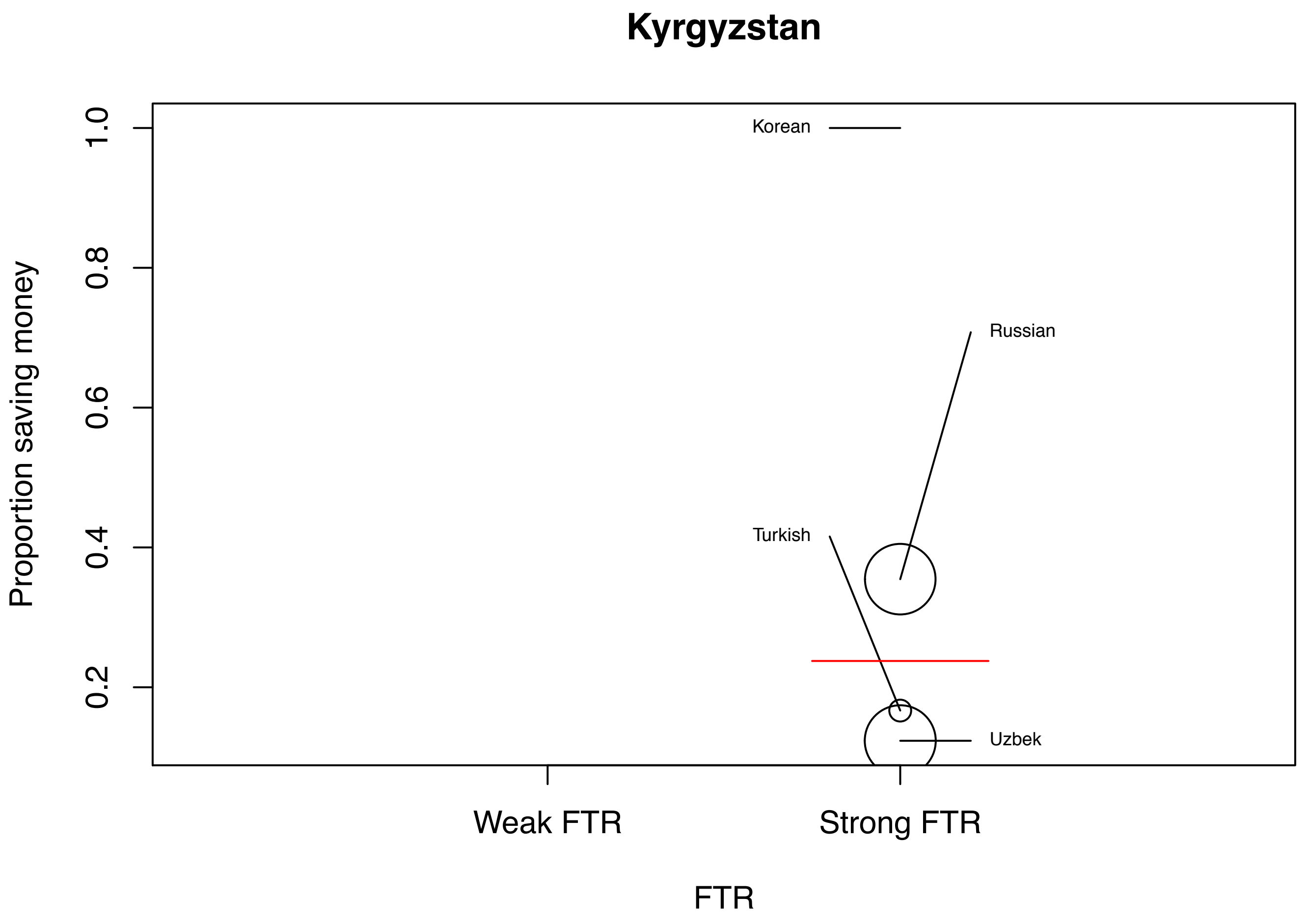
Kyrgyzstan
Proportion saving money
0.2
0.4
0.6
0.8
1.0
Korean
Russian
Turkish
Uzbek
Weak FTR
Strong FTR
FTR

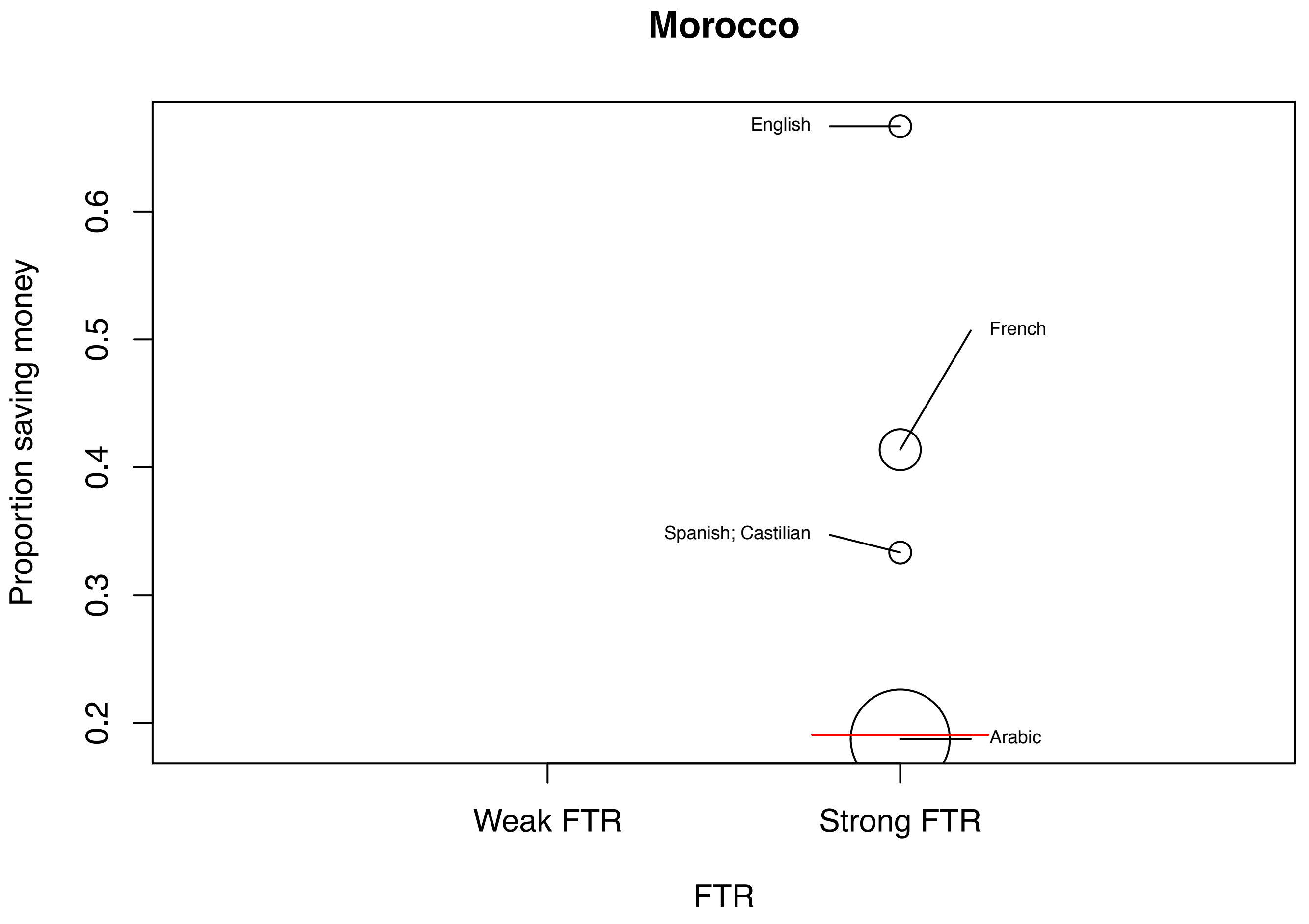
Morocco
Proportion saving money
0.2
0.3
0.4
0.5
0.6
English
French
Spanish; Castilian
Arabic
Weak FTR
Strong FTR
FTR

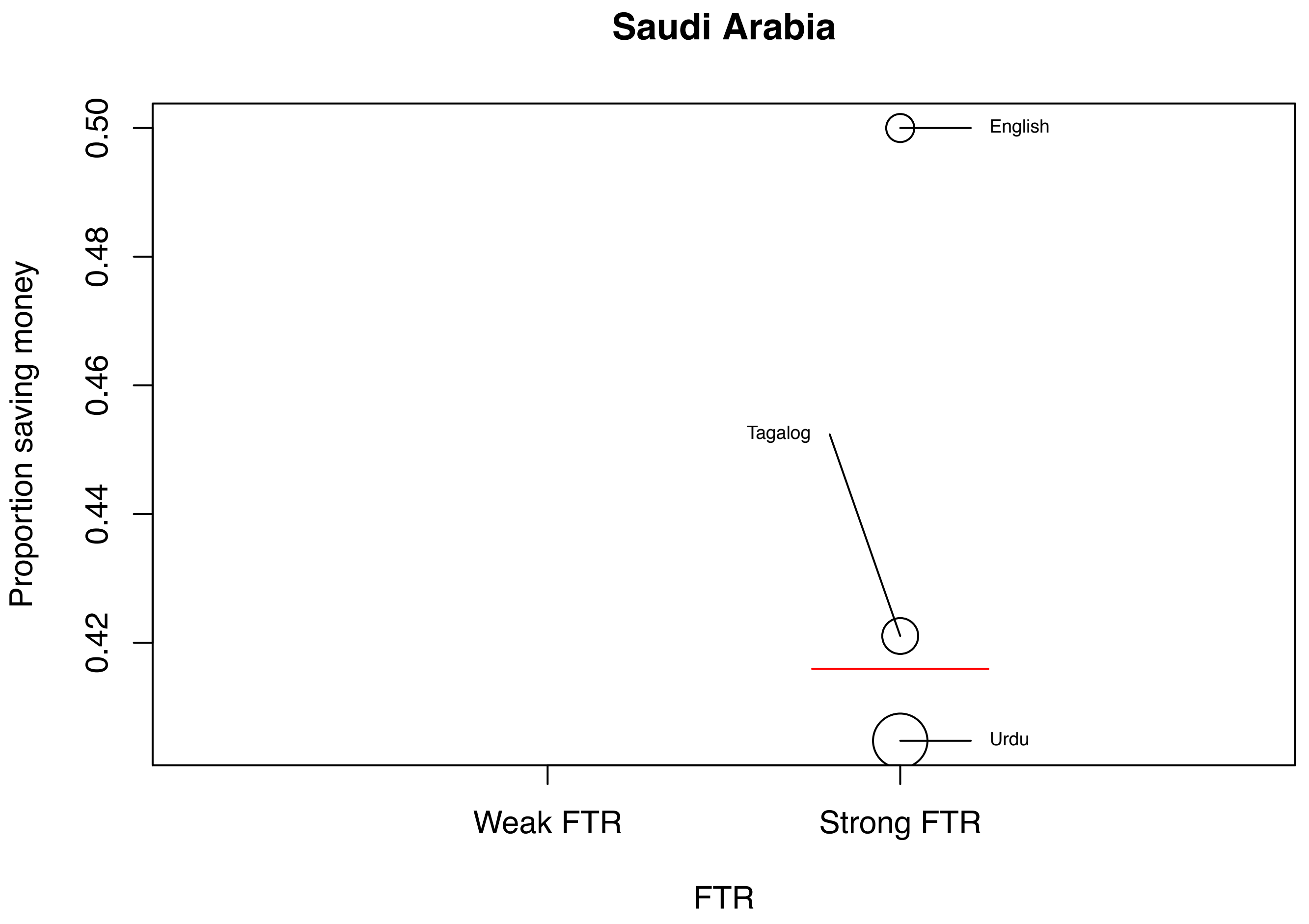

Saudi Arabia
English
Tagalog
Urdu
0.42
0.44
0.46
0.48
0.50
Proportion saving money
Weak FTR
Strong FTR
FTR

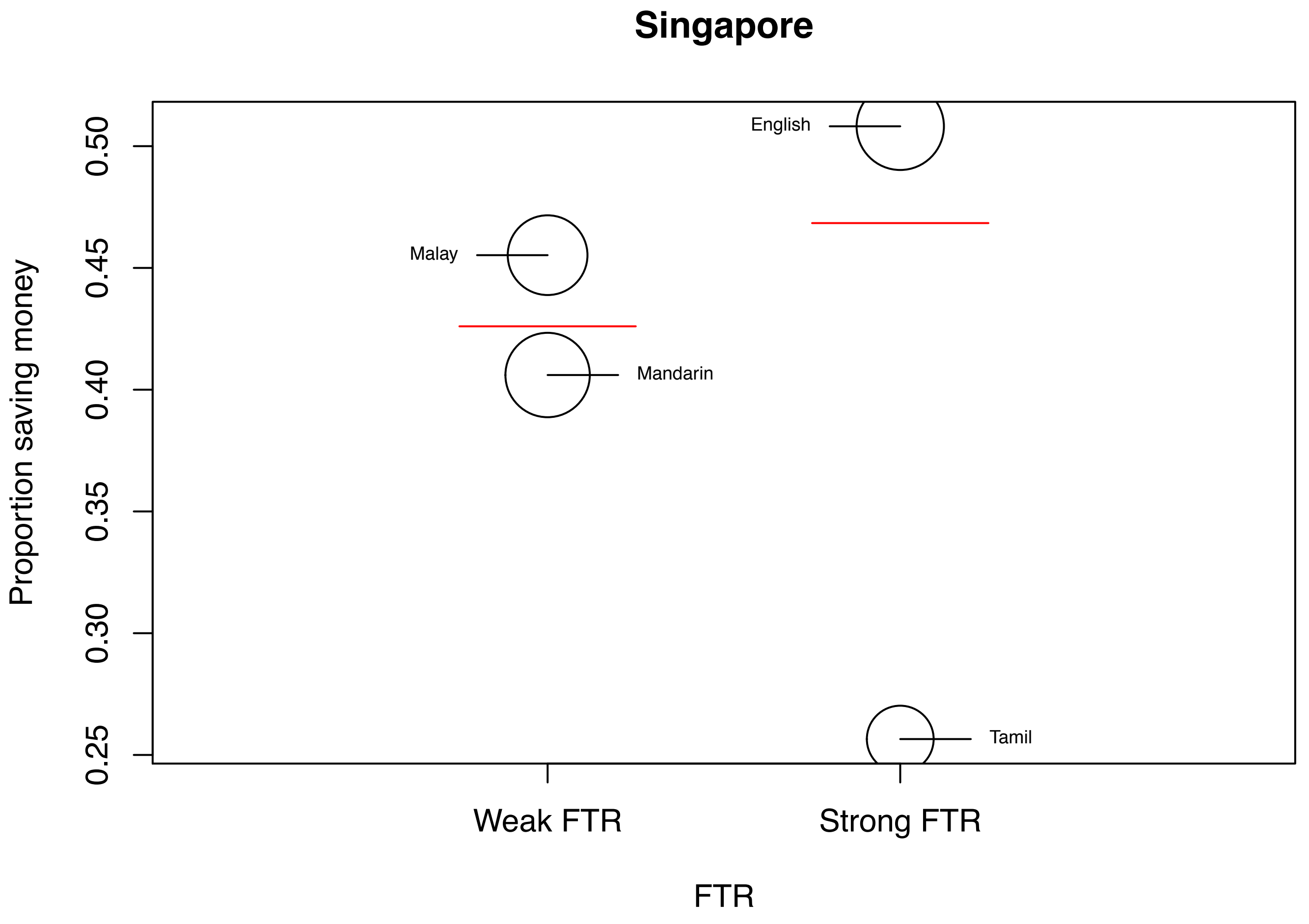

Singapore
English
Malay
Mandarin
Tamil
0.25
0.30
0.35
0.40
0.45
0.50
Proportion saving money
Weak FTR
Strong FTR
FTR

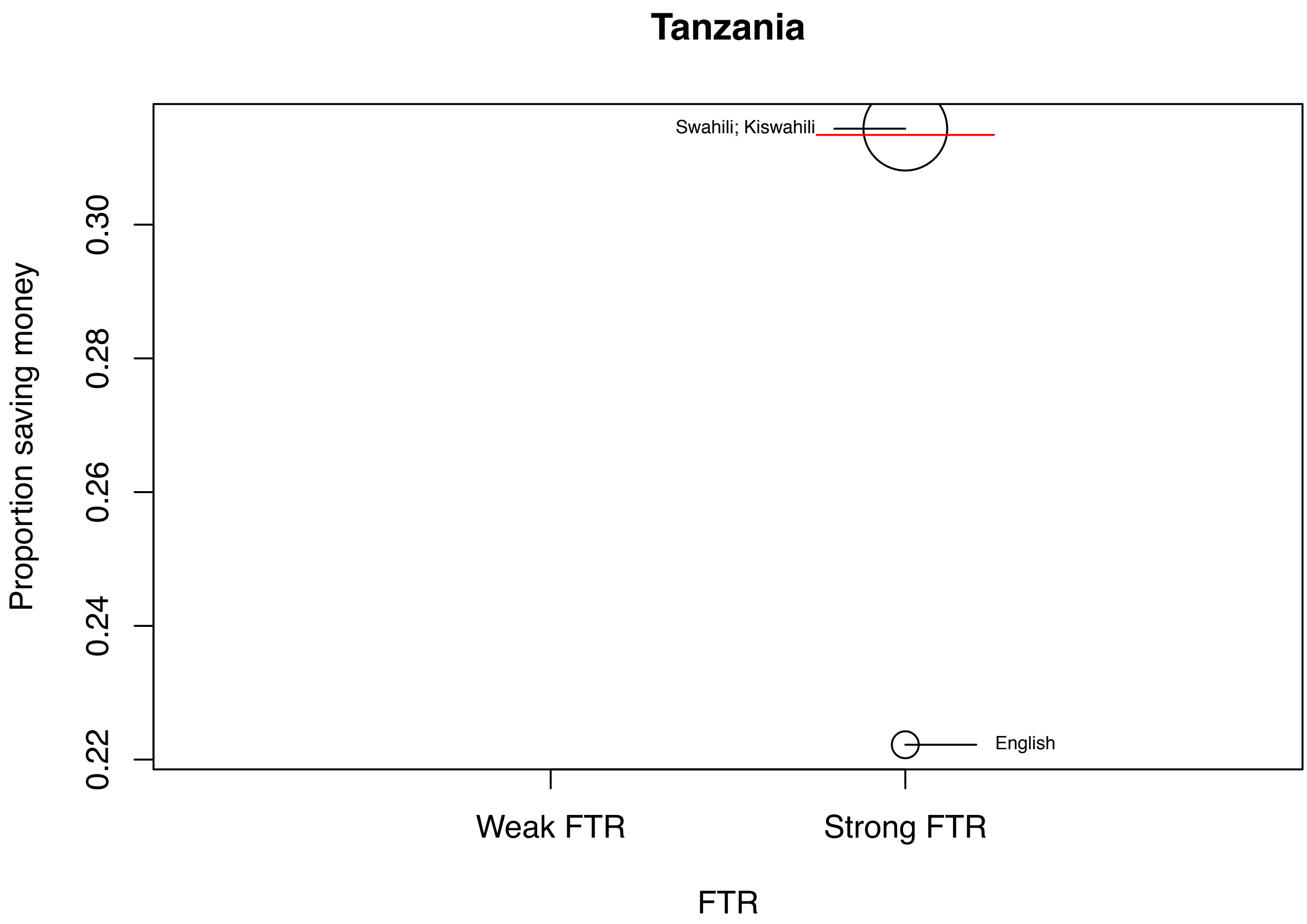
Tanzania
Swahili; Kiswahili
English
Proportion saving money
0.30
0.28
0.26
0.24
0.22
Weak FTR
Strong FTR
FTR

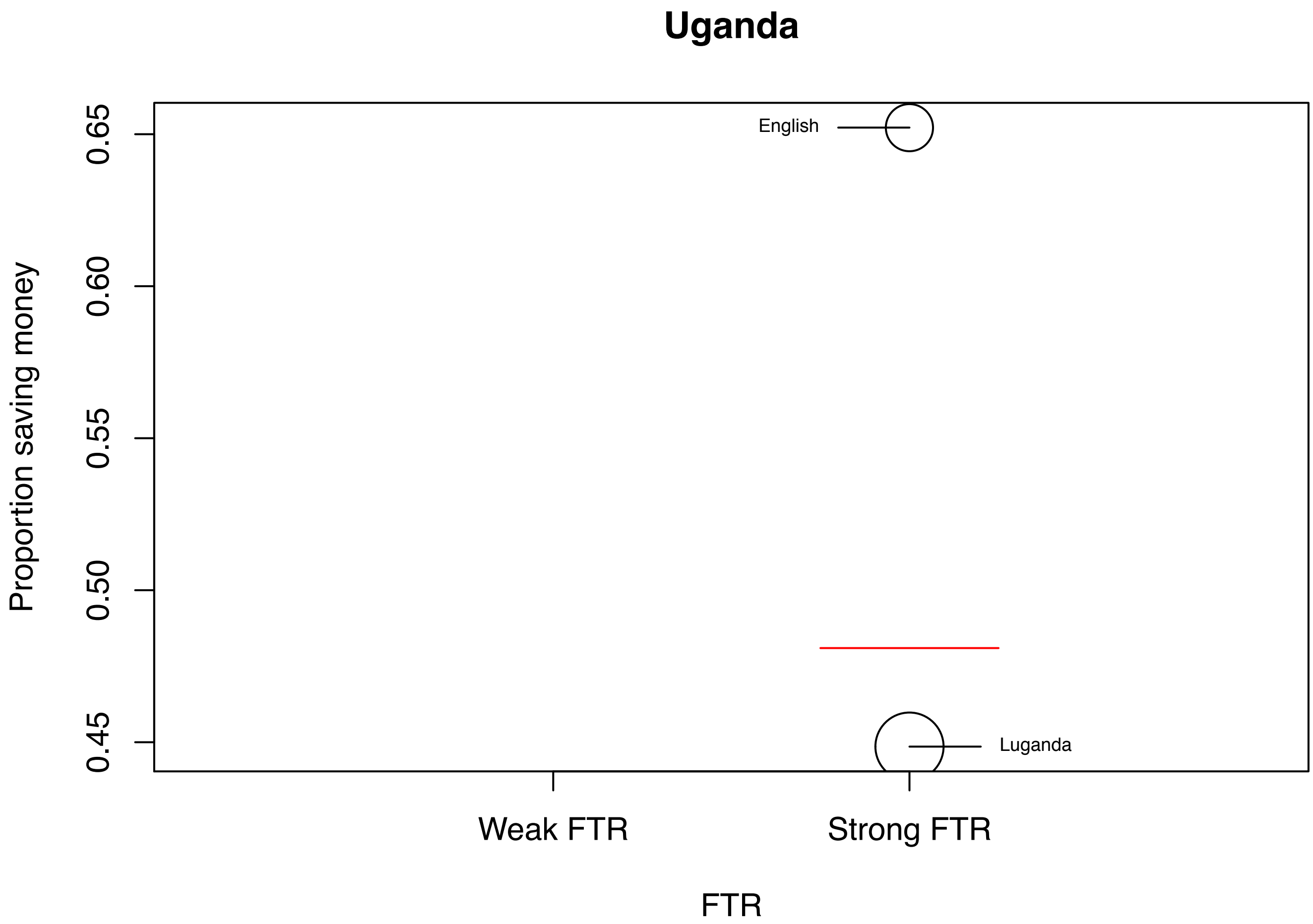
Uganda
English
Luganda
Proportion saving money
0.65
0.60
0.55
0.50
0.45
Weak FTR
Strong FTR
FTR

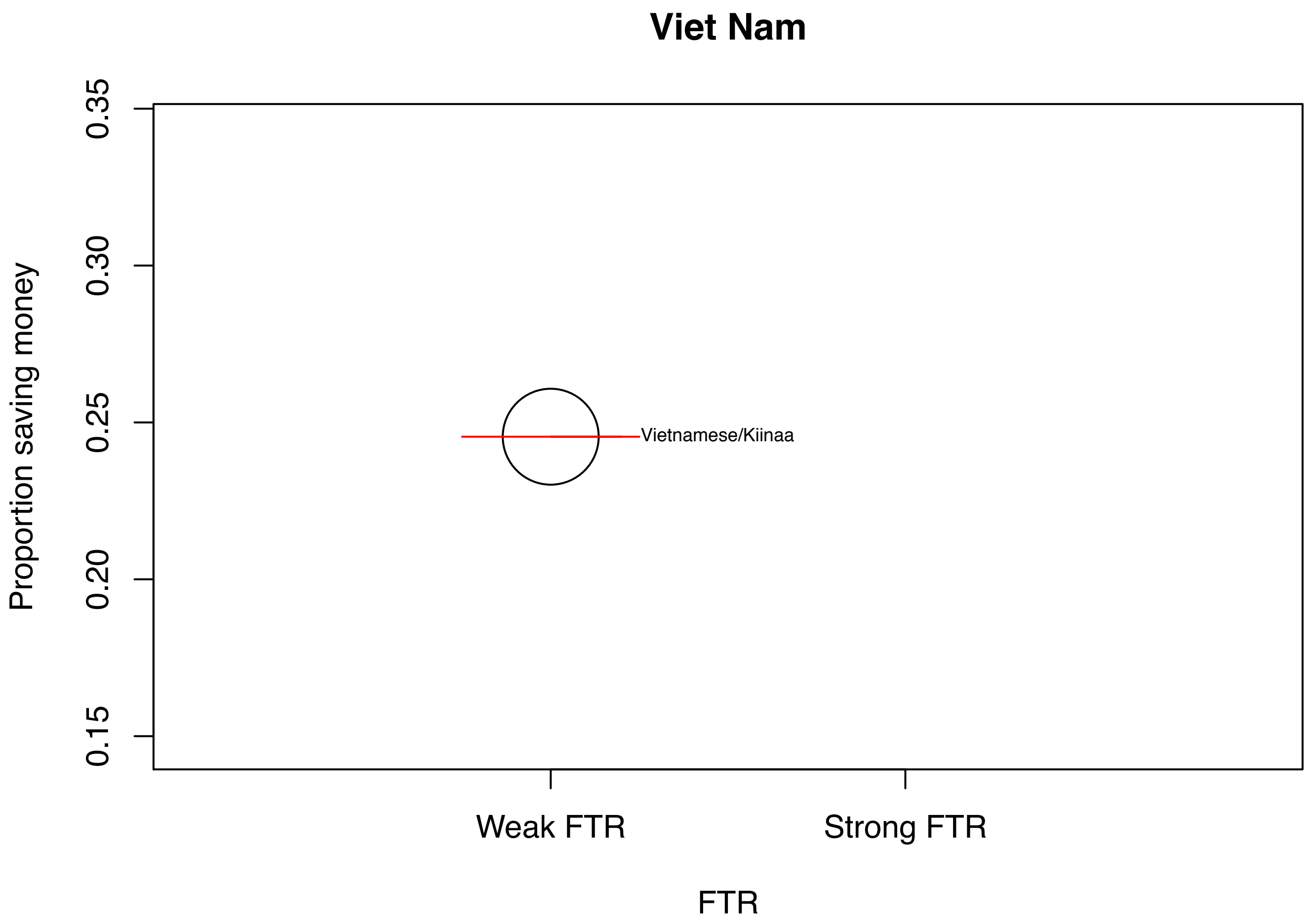
Viet Nam
Proportion saving money
0.35
0.30
0.25
0.20
0.15
Vietnamese/Kiinaa
Weak FTR
Strong FTR
FTR

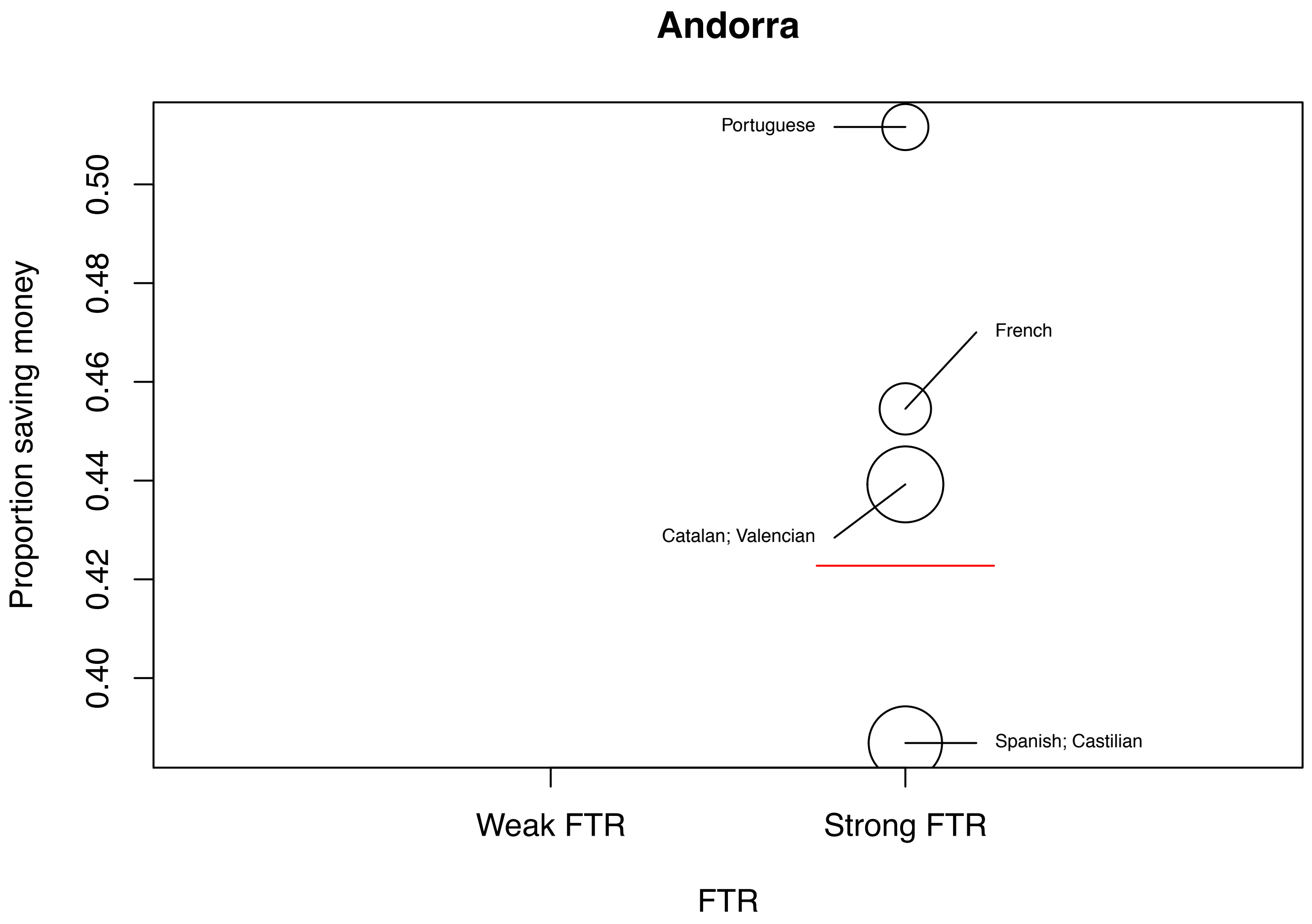
Andorra
Proportion saving money
0.50
0.48
0.46
0.44
0.42
0.40
Portuguese
French
Catalan; Valencian
Spanish; Castilian
Weak FTR
Strong FTR
FTR

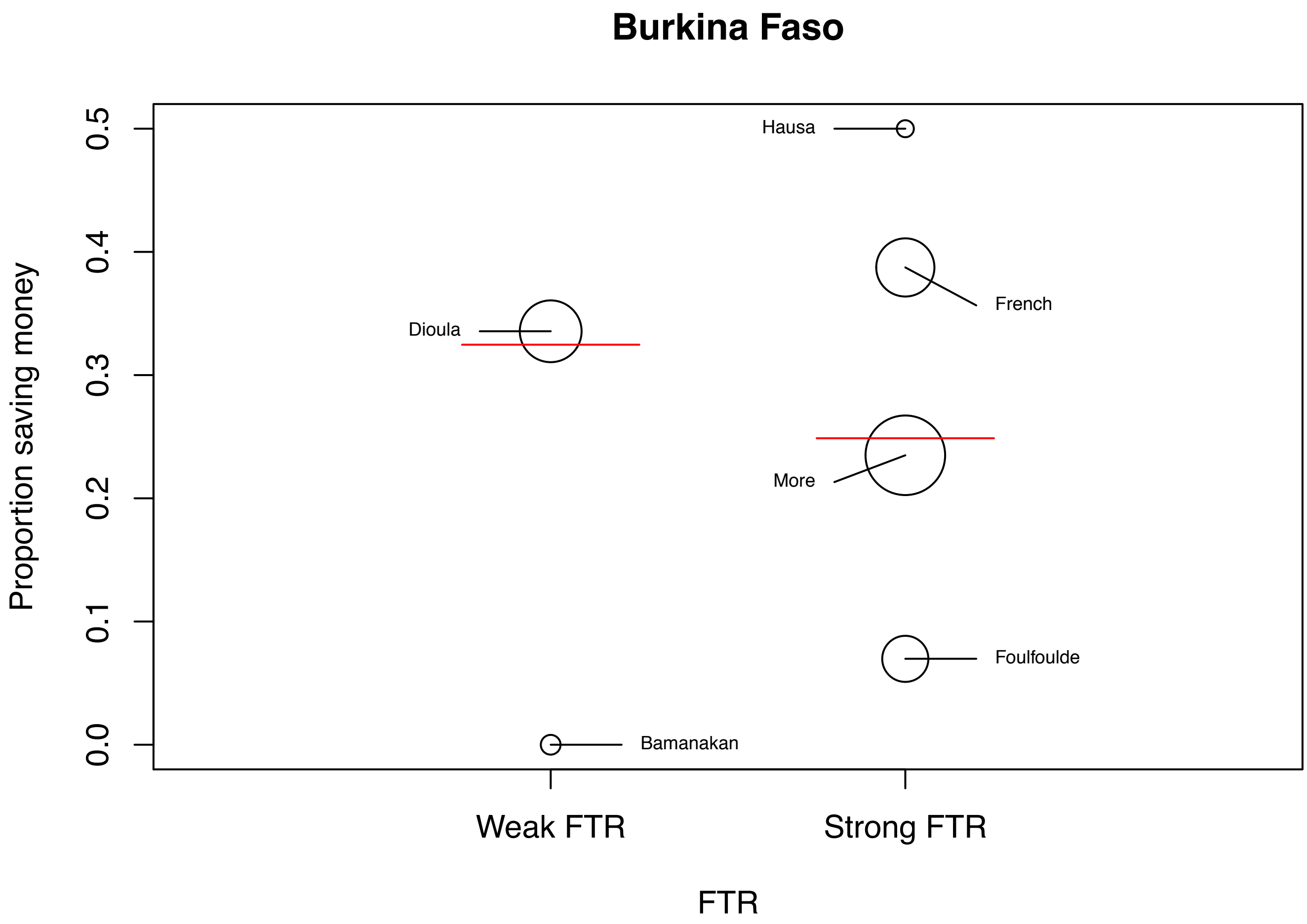
Burkina Faso
Proportion saving money
0.0
0.1
0.2
0.3
0.4
0.5
Hausa
French
Dioula
More
Foulfoulde
Bamanakan
Weak FTR
Strong FTR
FTR

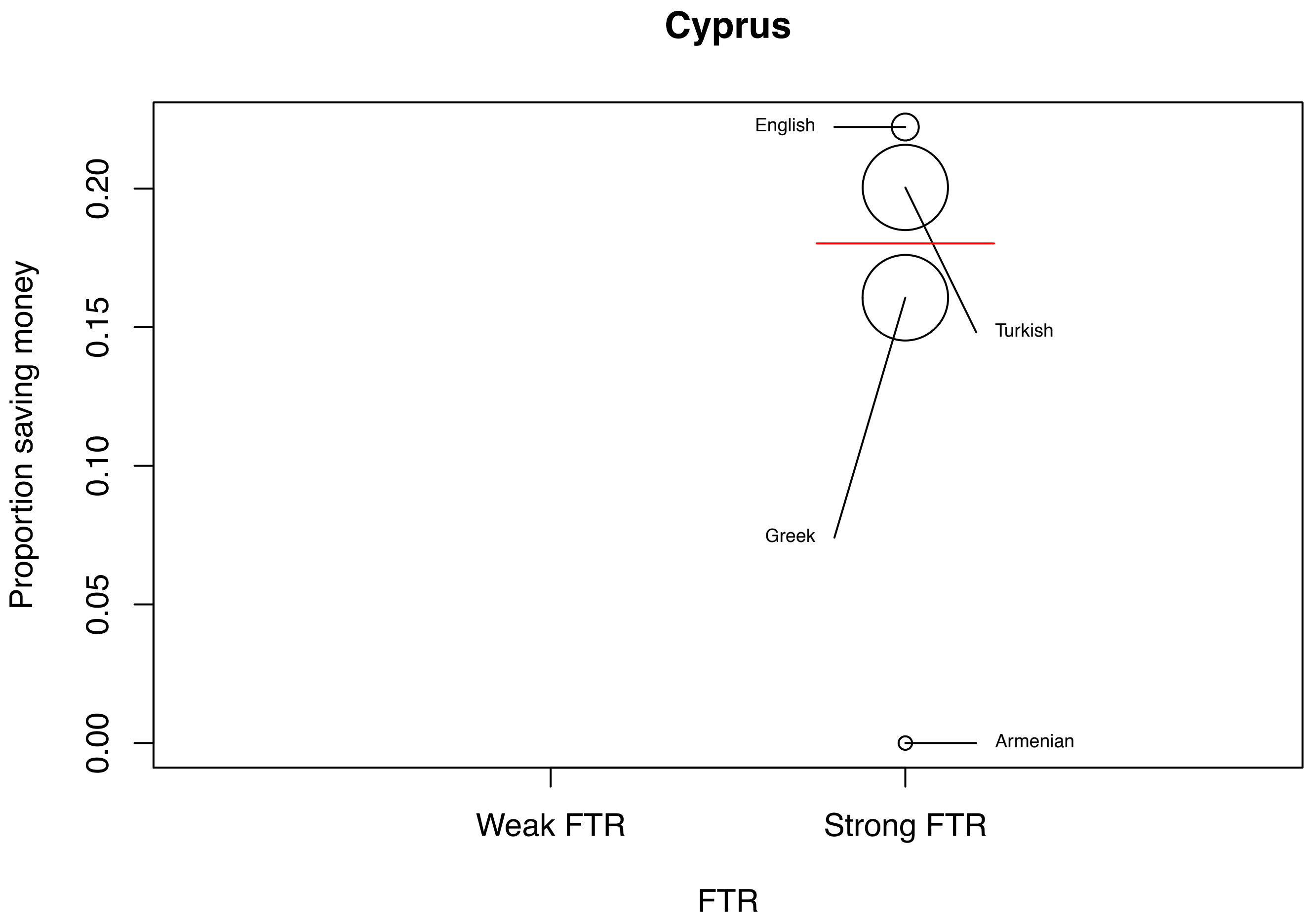
Cyprus
Proportion saving money
0.00
0.05
0.10
0.15
0.20
English
Turkish
Greek
Armenian
Weak FTR
Strong FTR
FTR

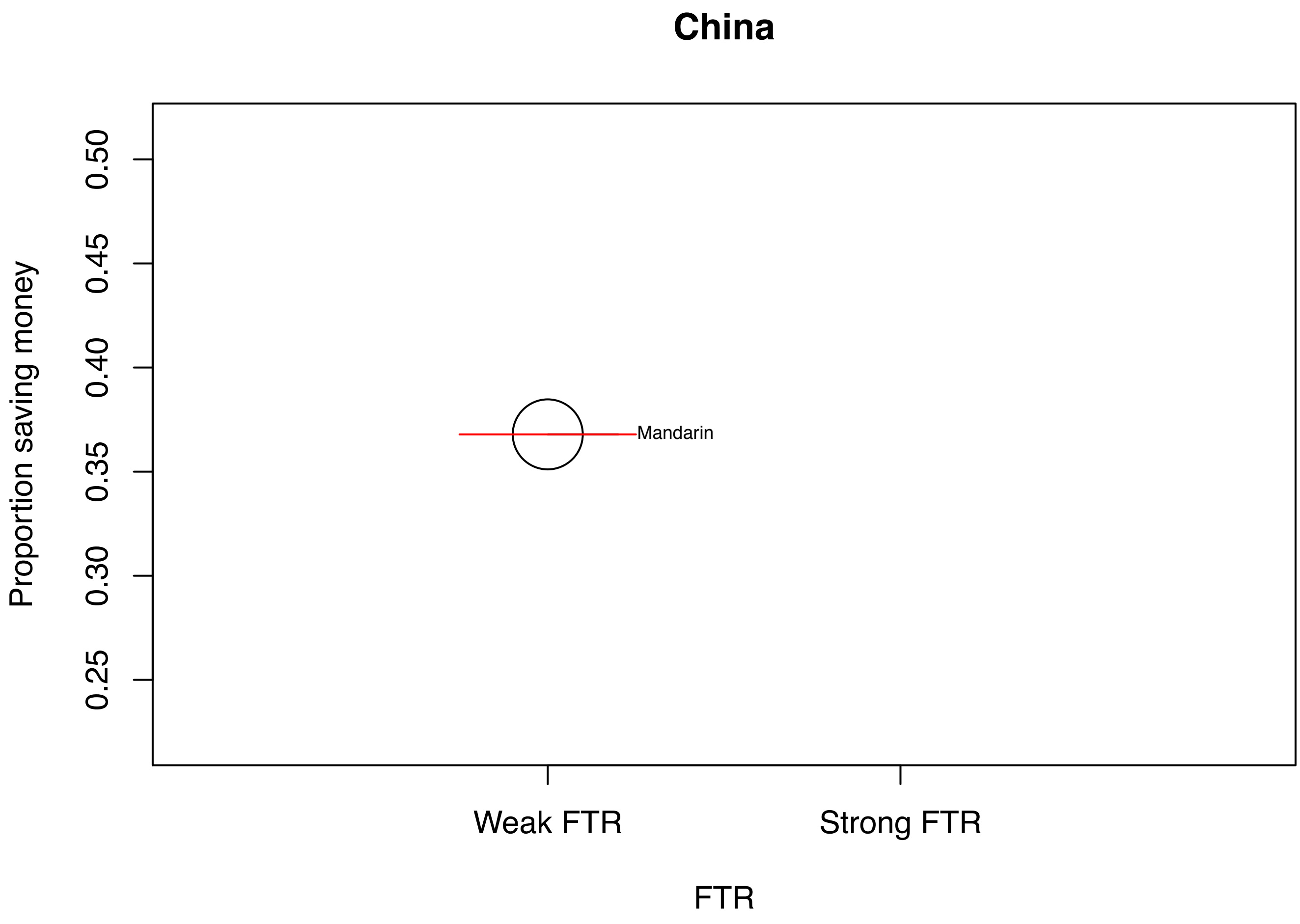
China
Proportion saving money
0.25
0.30
0.35
0.40
0.45
0.50
Mandarin
Weak FTR
Strong FTR
FTR

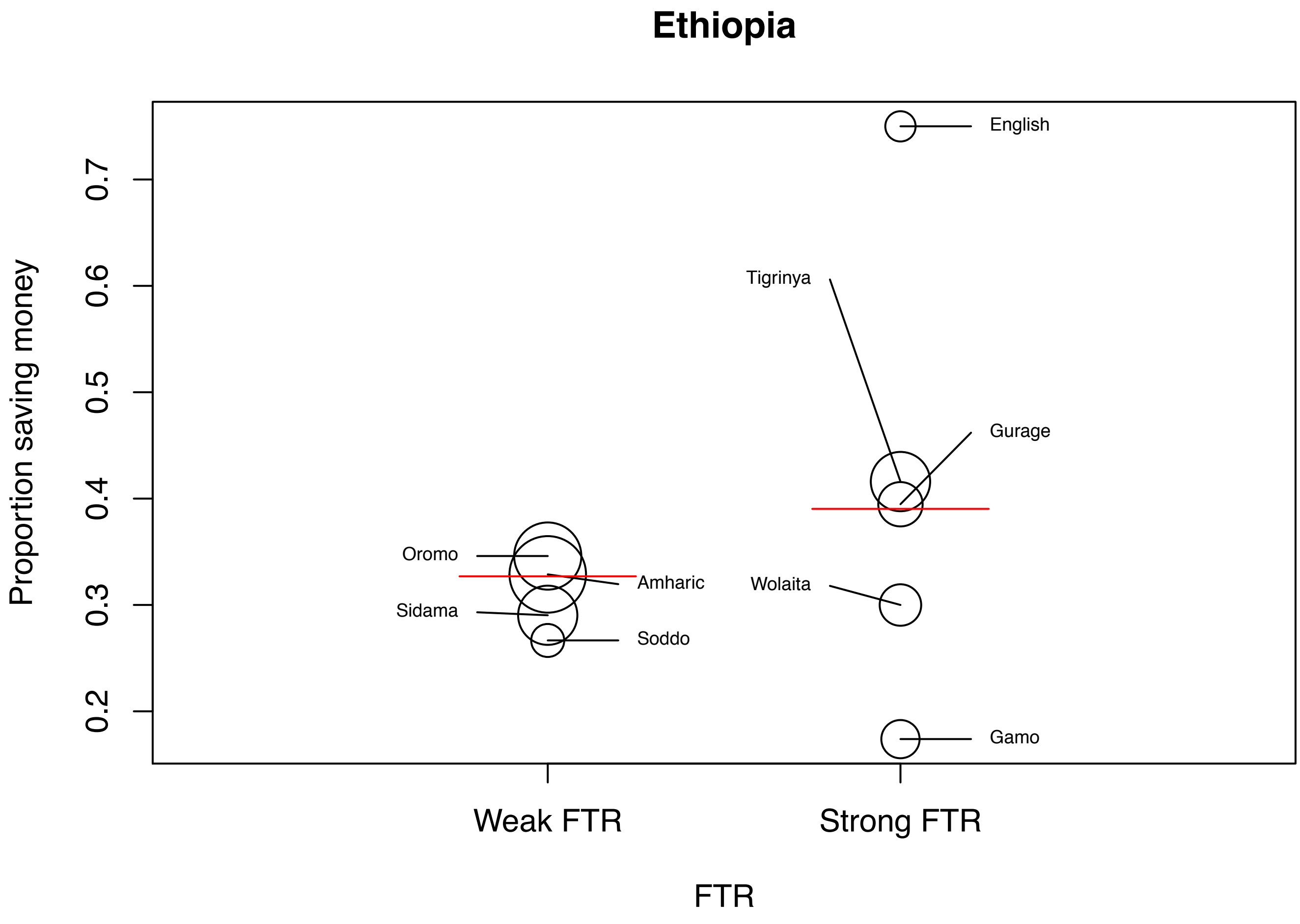
Ethiopia
Proportion saving money
0.2
0.3
0.4
0.5
0.6
0.7
English
Tigrinya
Gurage
Oromo
Amharic
Wolaita
Sidama
Soddo
Gamo
Weak FTR
Strong FTR
FTR

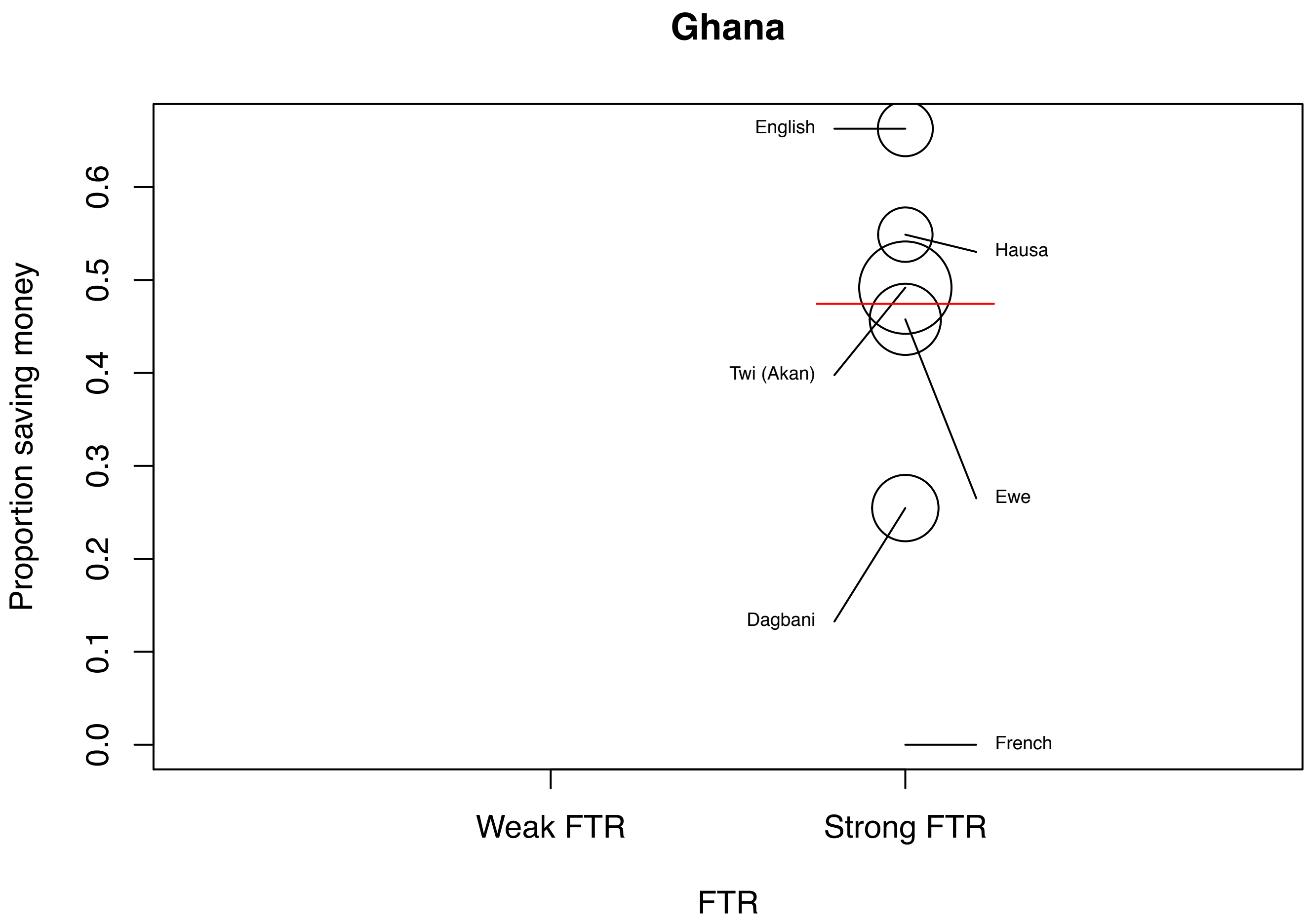
Ghana
English
Hausa
Twi (Akan)
Ewe
Dagbani
French
Proportion saving money
0.0
0.1
0.2
0.3
0.4
0.5
0.6
Weak FTR
Strong FTR
FTR

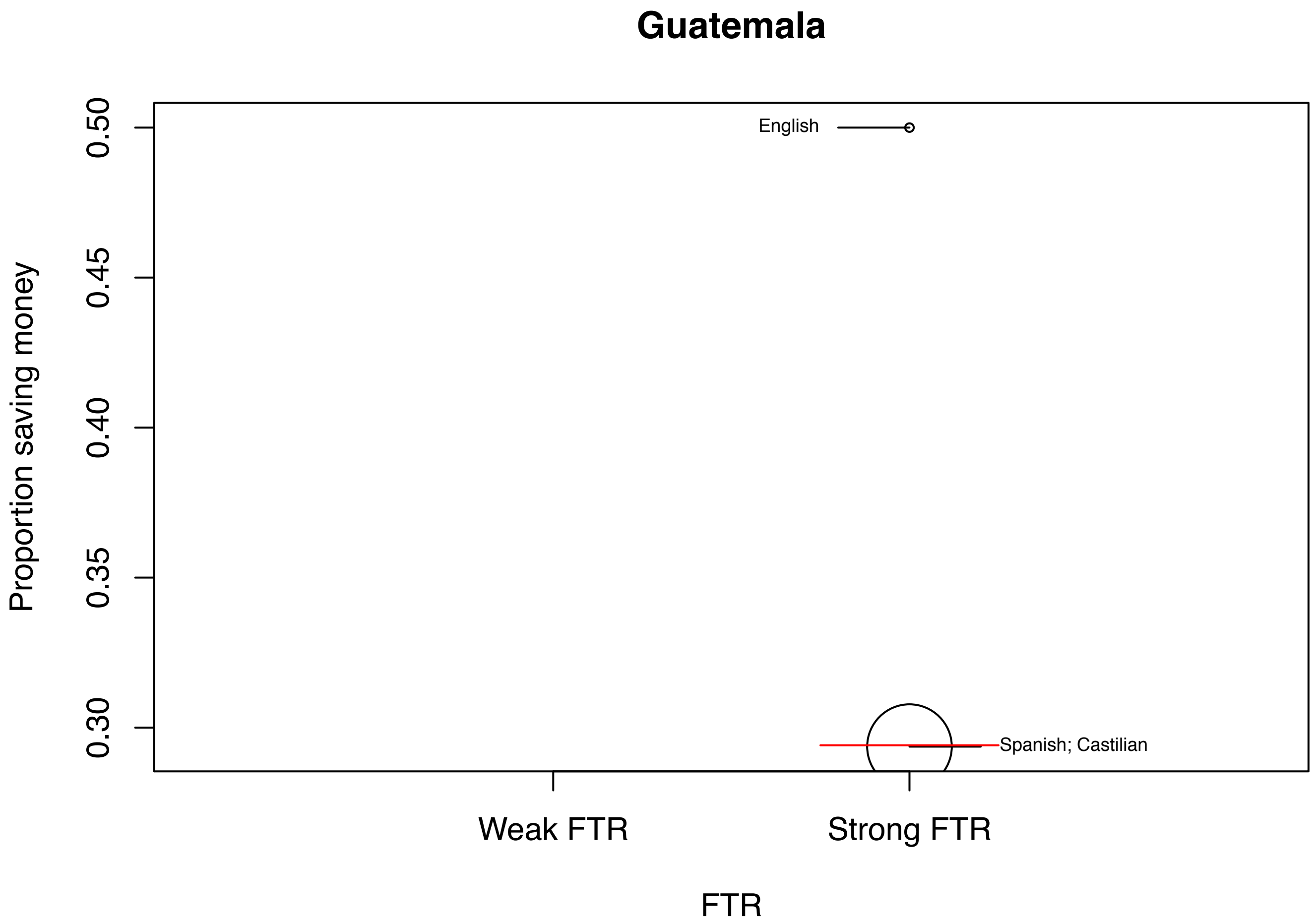
Guatemala
English
Spanish; Castilian
Proportion saving money
0.30
0.35
0.40
0.45
0.50
Weak FTR
Strong FTR
FTR

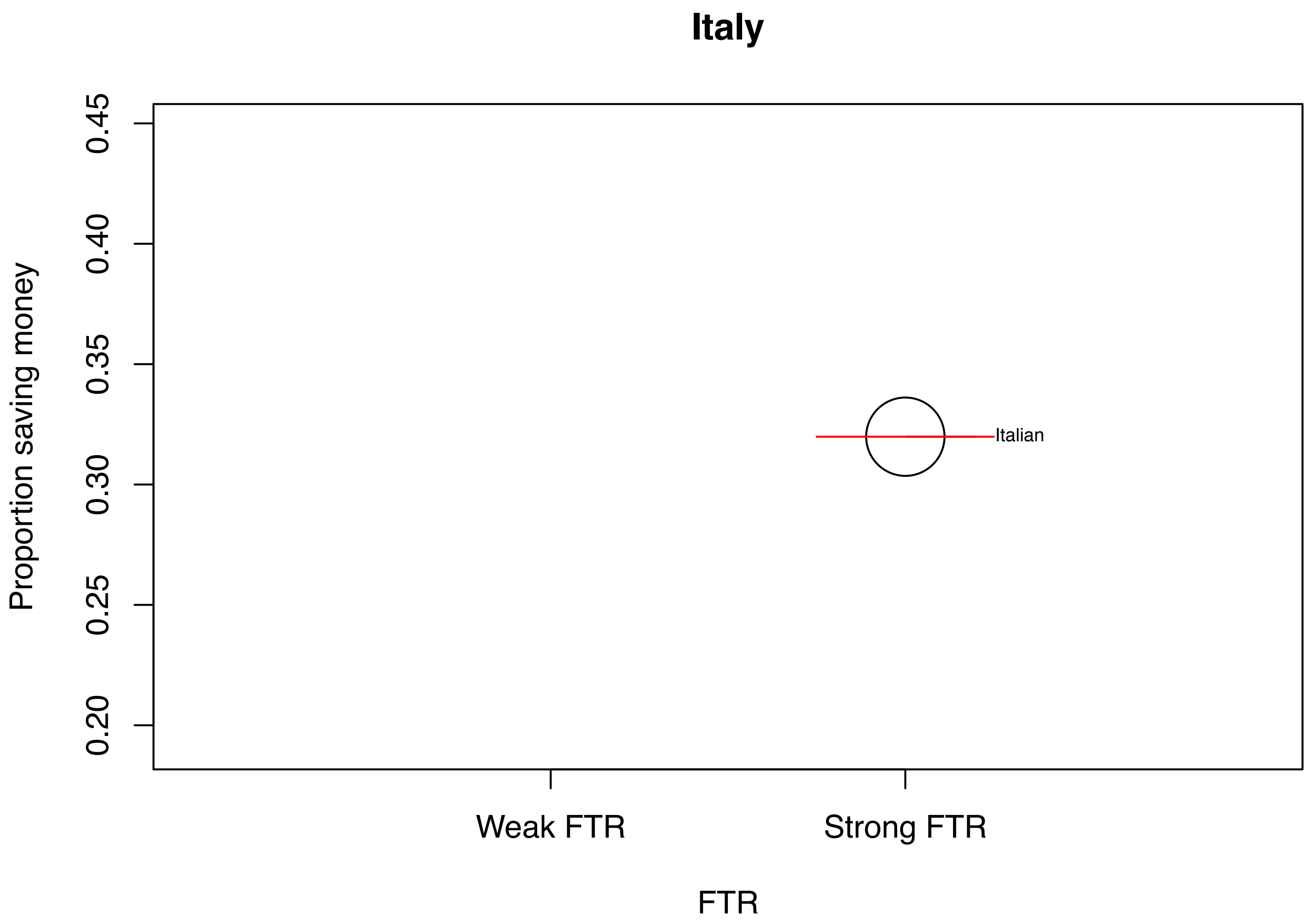

Italy
Proportion saving money
0.20
0.25
0.30
0.35
0.40
0.45
Italian
Weak FTR
Strong FTR
FTR

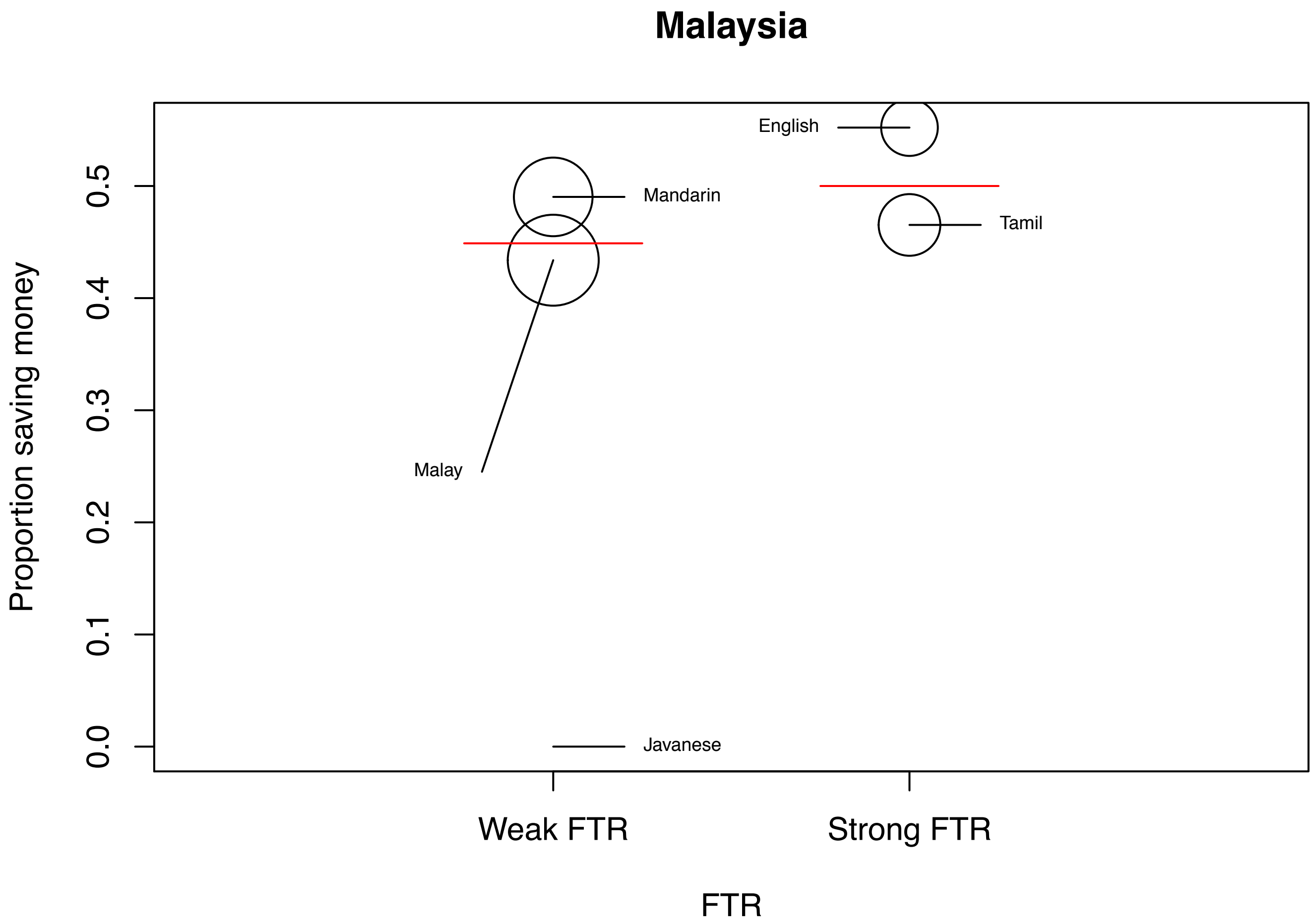

Malaysia
Proportion saving money
0.0
0.1
0.2
0.3
0.4
0.5
English
Mandarin
Tamil
Malay
Javanese
Weak FTR
Strong FTR
FTR

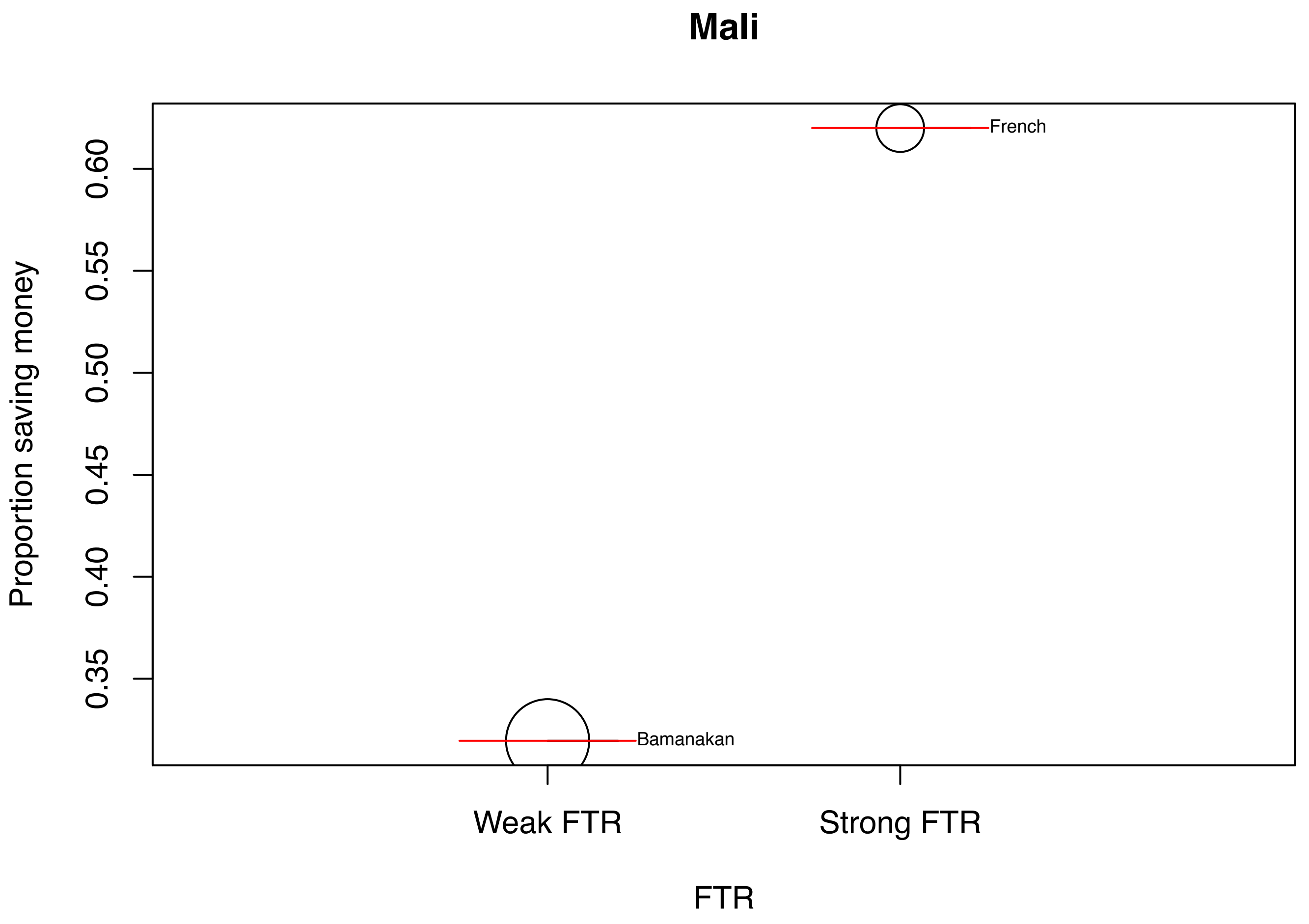
Mali
Proportion saving money
0.60
0.55
0.50
0.45
0.40
0.35
French
Bamanakan
Weak FTR
Strong FTR
FTR

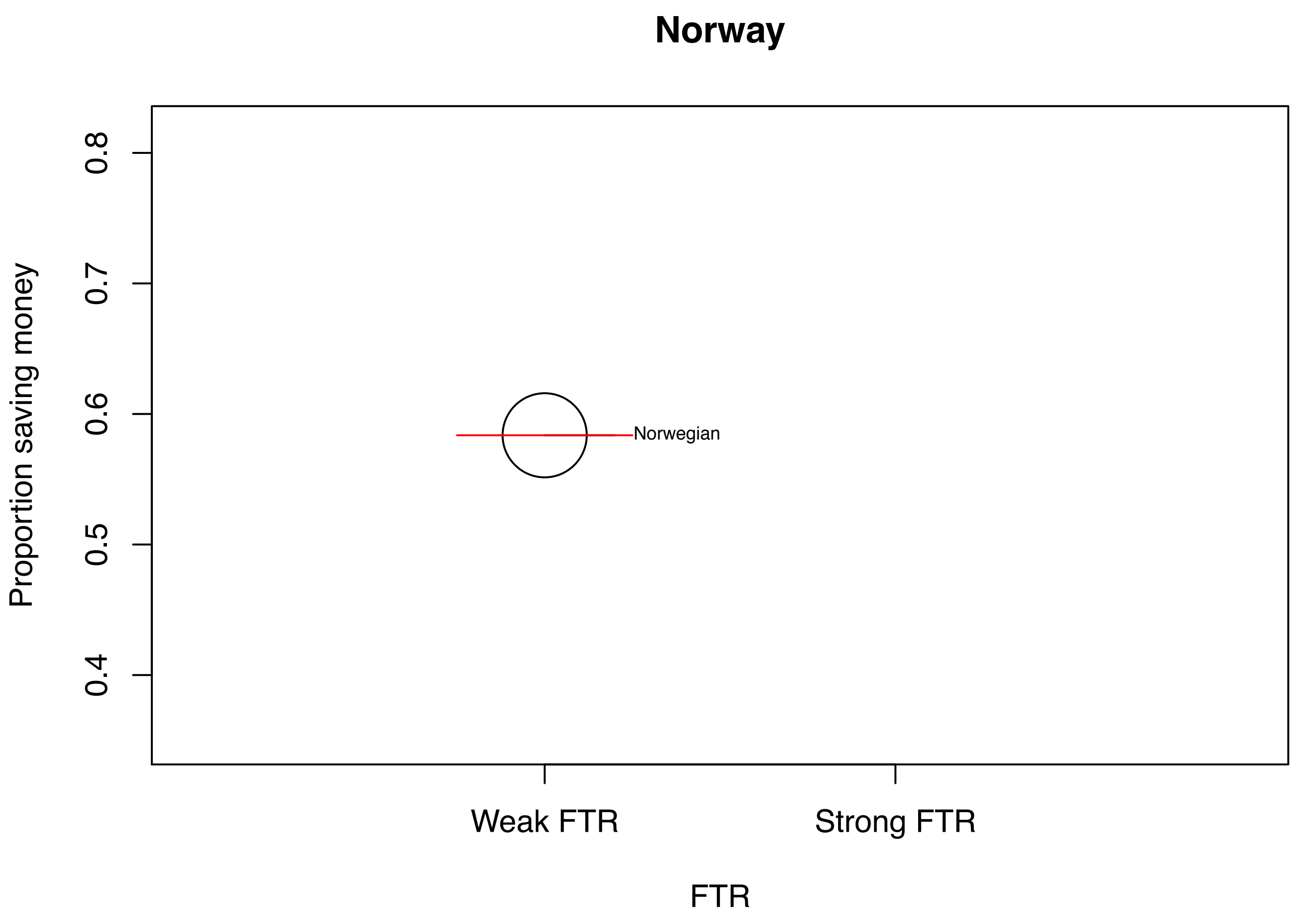
Norway
Proportion saving money
0.8
0.7
0.6
0.5
0.4
Norwegian
Weak FTR
Strong FTR
FTR

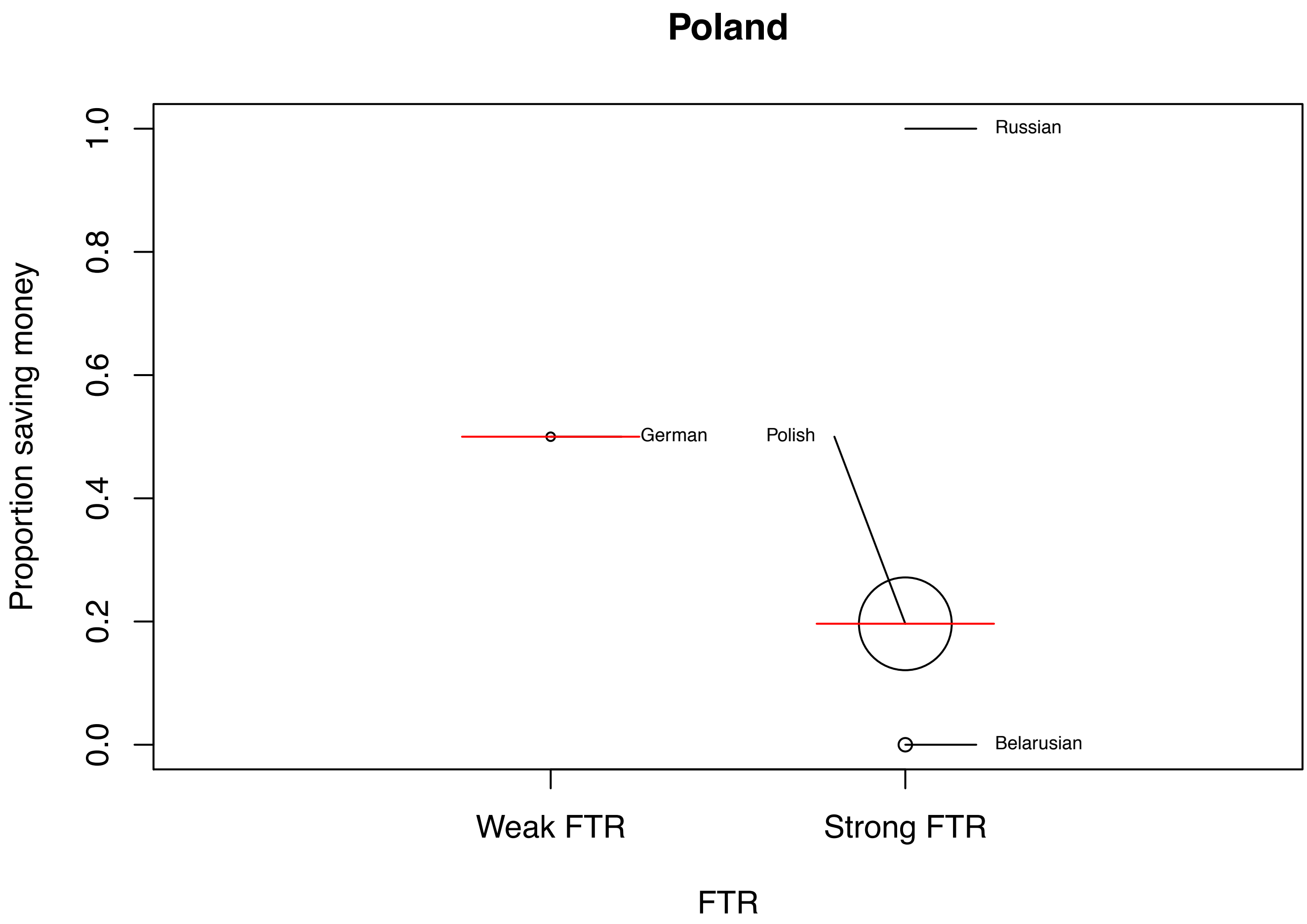

Poland
Proportion saving money
1.0
0.8
0.6
0.4
0.2
0.0
Russian
German
Polish
Belarusian
Weak FTR
Strong FTR
FTR

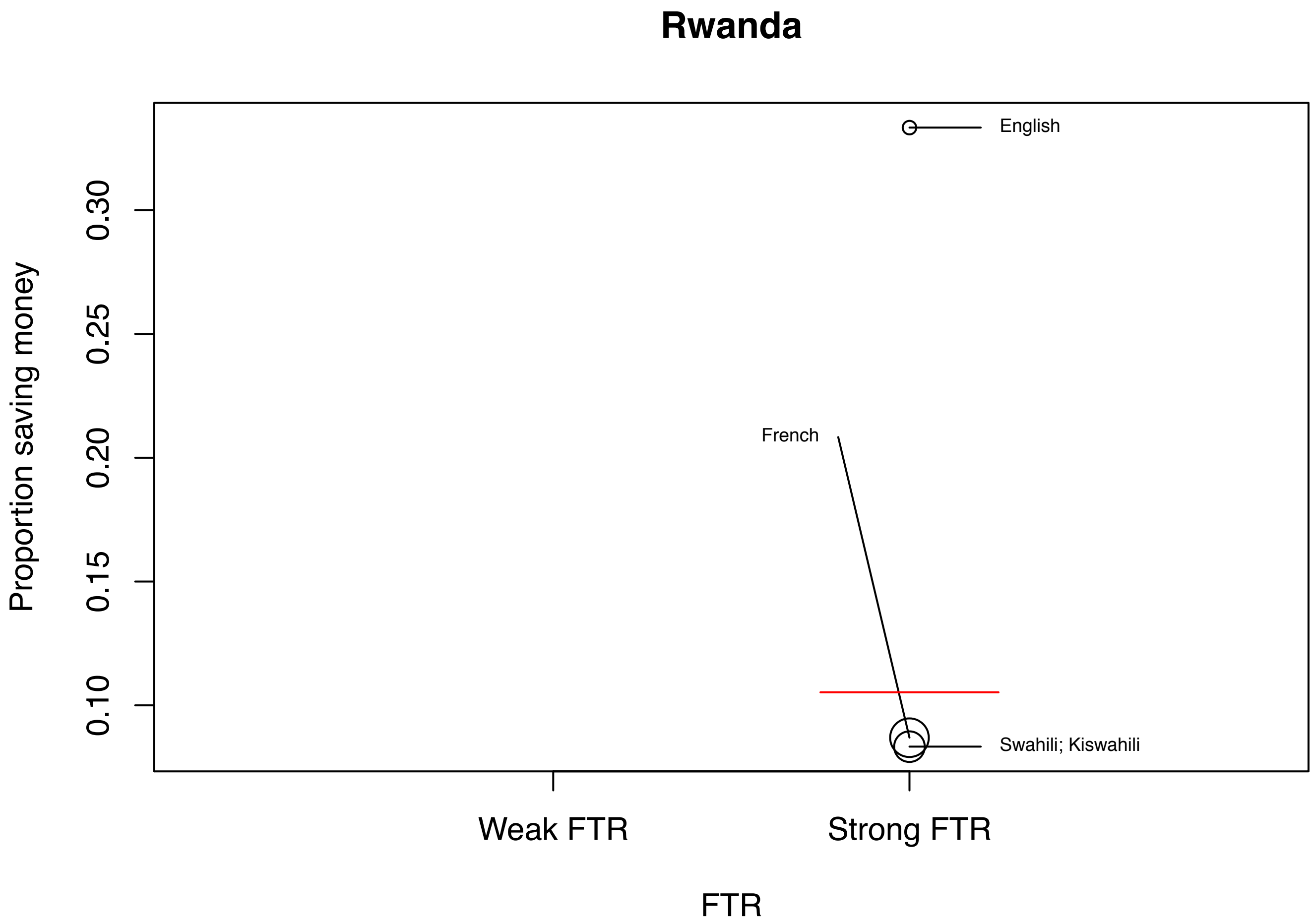

Rwanda
Proportion saving money
0.30
0.25
0.20
0.15
0.10
English
French
Swahili; Kiswahili
Weak FTR
Strong FTR
FTR

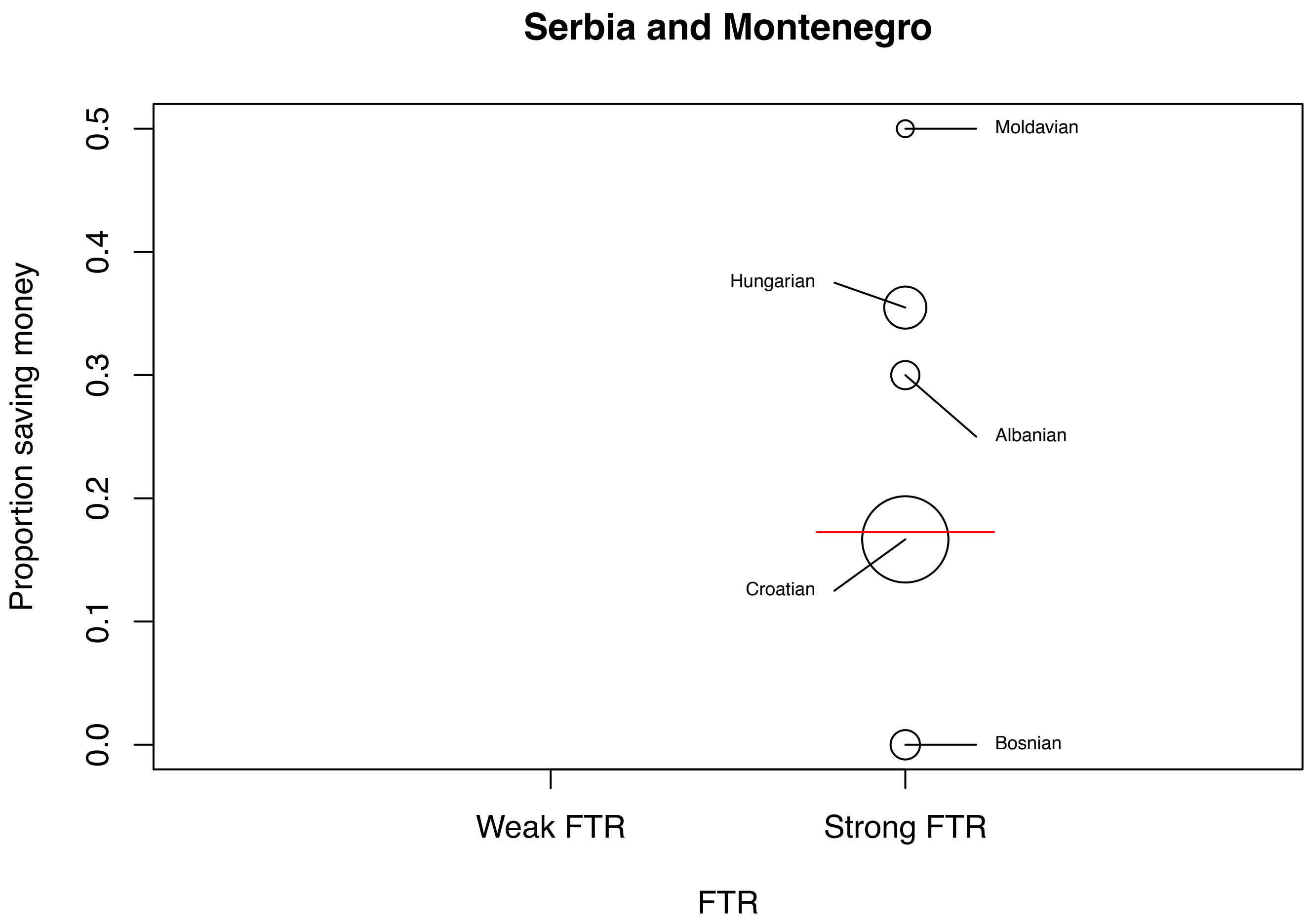
Serbia and Montenegro
Proportion saving money
0.0
0.1
0.2
0.3
0.4
0.5
Moldavian
Hungarian
Albanian
Croatian
Bosnian
Weak FTR
Strong FTR
FTR

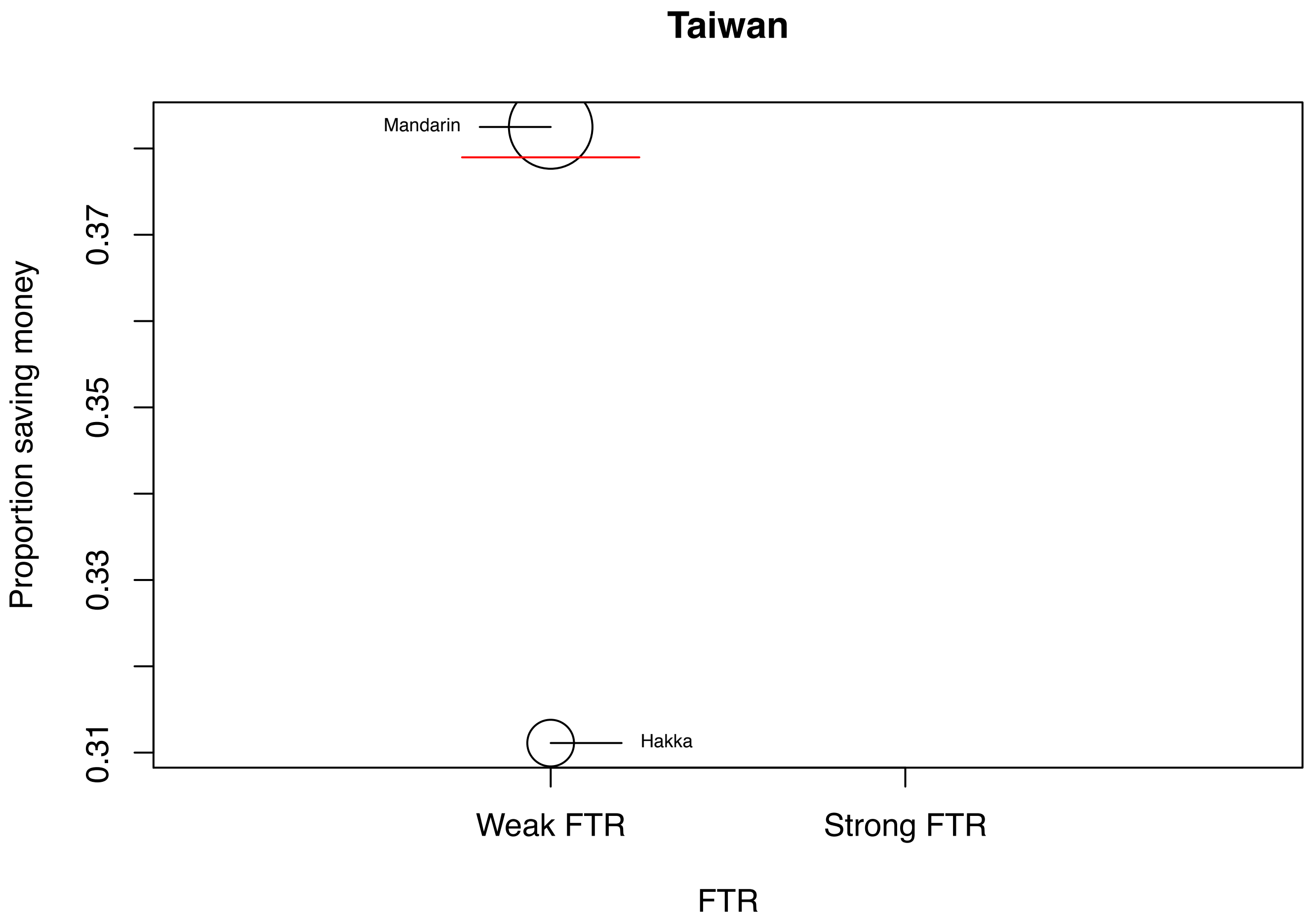
Taiwan
Proportion saving money
0.31
0.33
0.35
0.37
Mandarin
Hakka
Weak FTR
Strong FTR
FTR

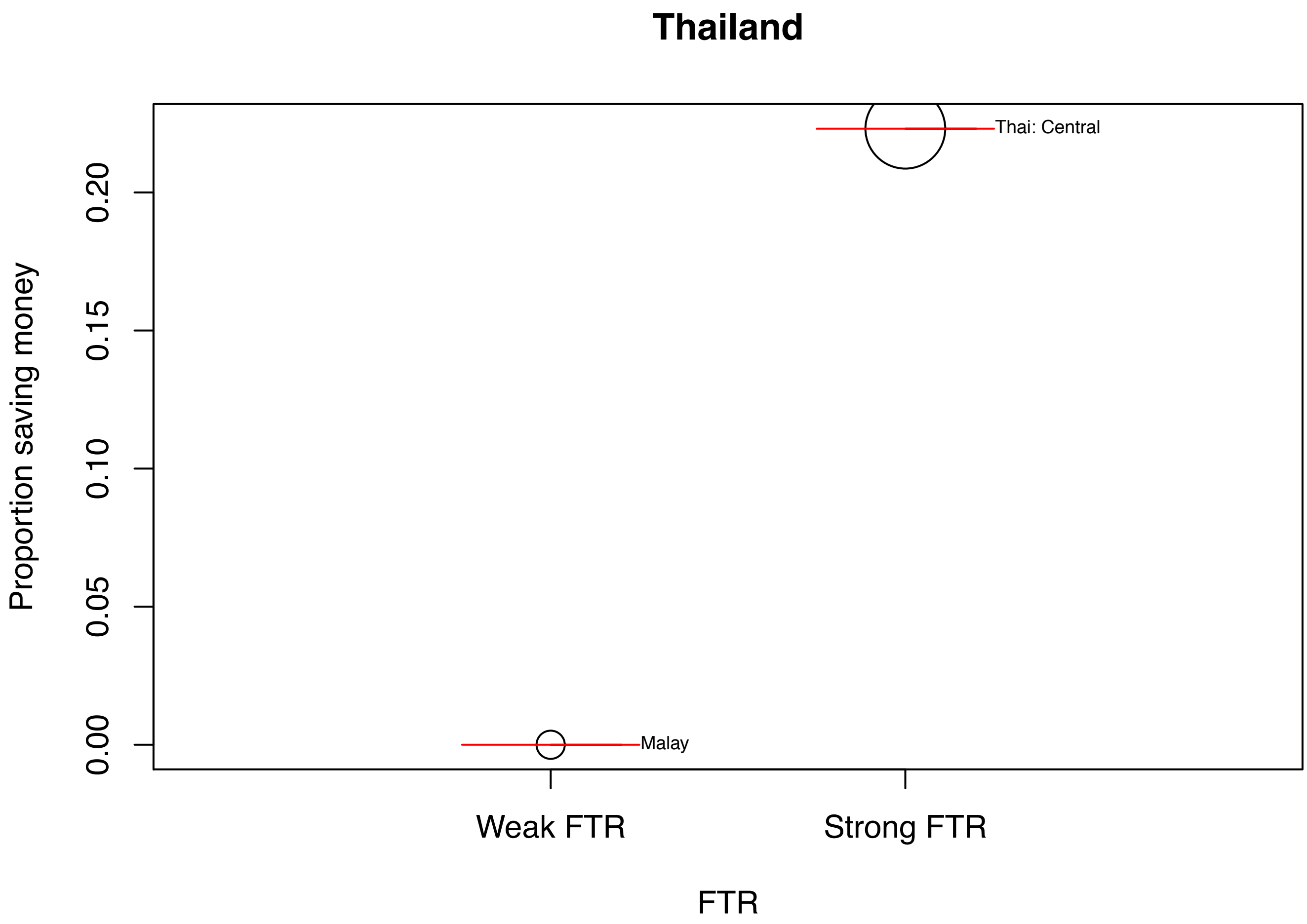

Thailand
Proportion saving money
0.00
0.05
0.10
0.15
0.20
Thai: Central
Malay
Weak FTR
Strong FTR
FTR

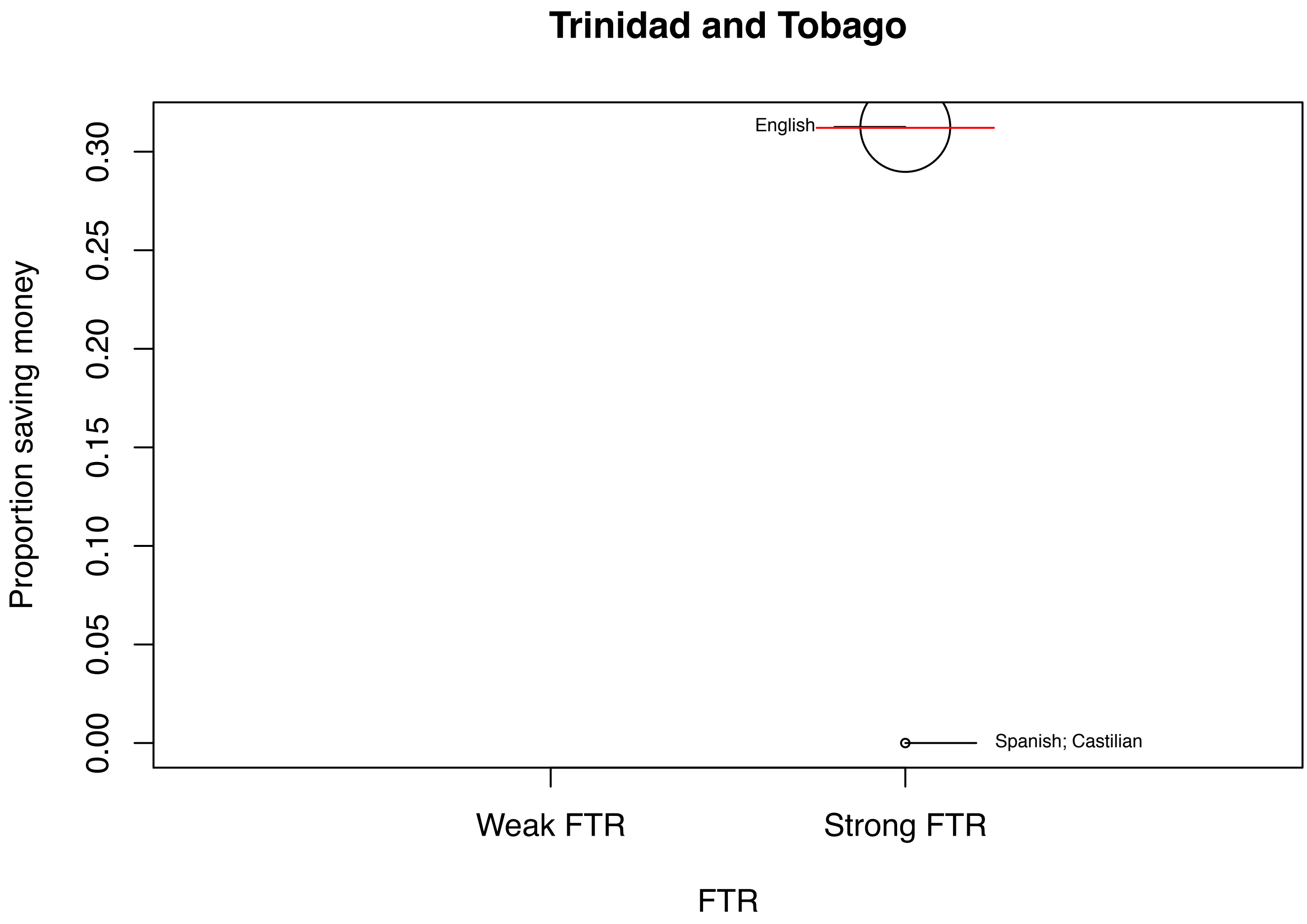

Trinidad and Tobago
Proportion saving money
0.00
0.05
0.10
0.15
0.20
0.25
0.30
English
Spanish; Castilian
Weak FTR
Strong FTR
FTR

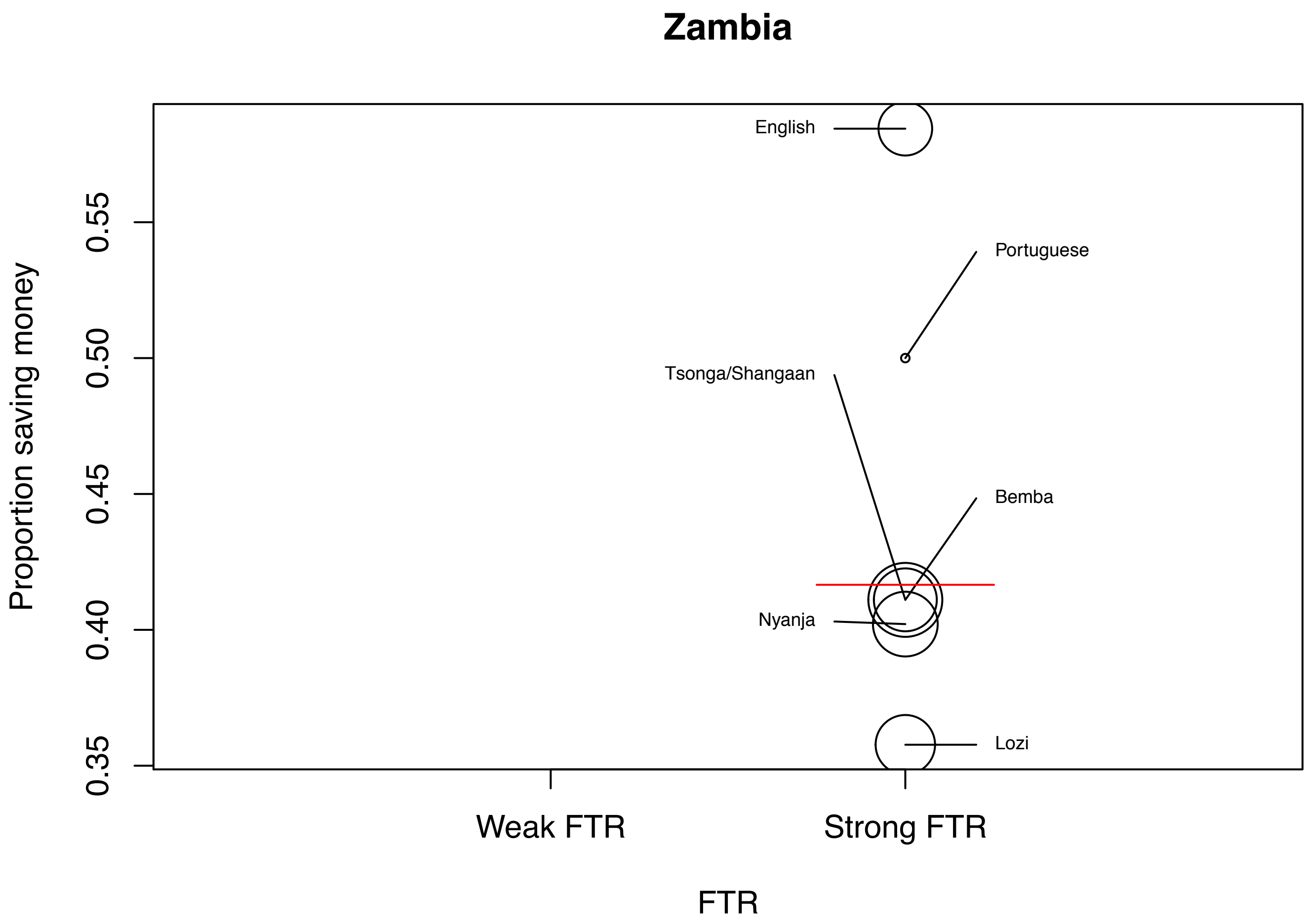
Zambia
English
Portuguese
Tsonga/Shangaan
Bemba
Nyanja
Lozi
0.35
0.40
0.45
0.50
0.55
Proportion saving money
Weak FTR
Strong FTR
FTR

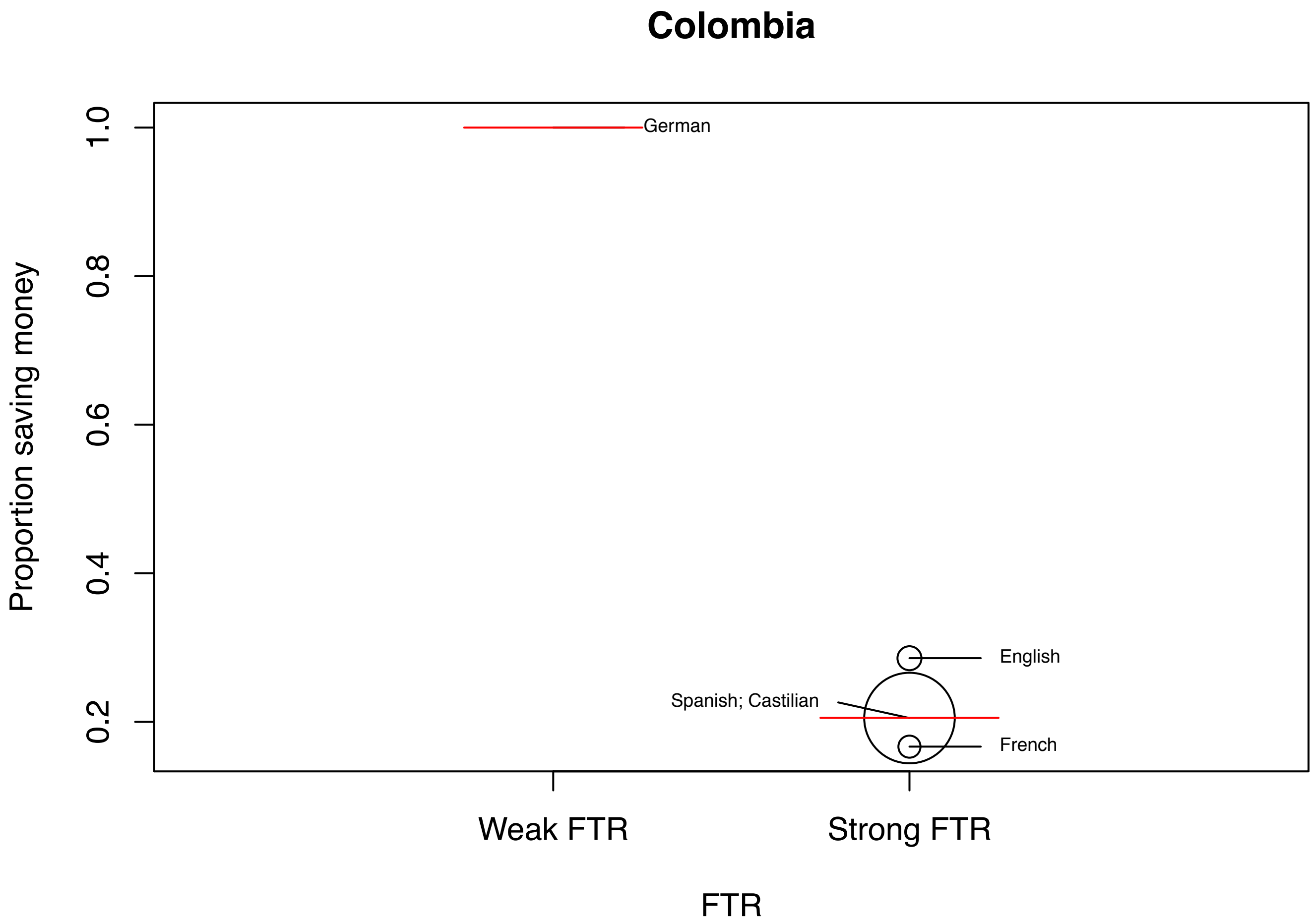
Colombia
German
English
Spanish; Castilian
French
0.2
0.4
0.6
0.8
1.0
Proportion saving money
Weak FTR
Strong FTR
FTR

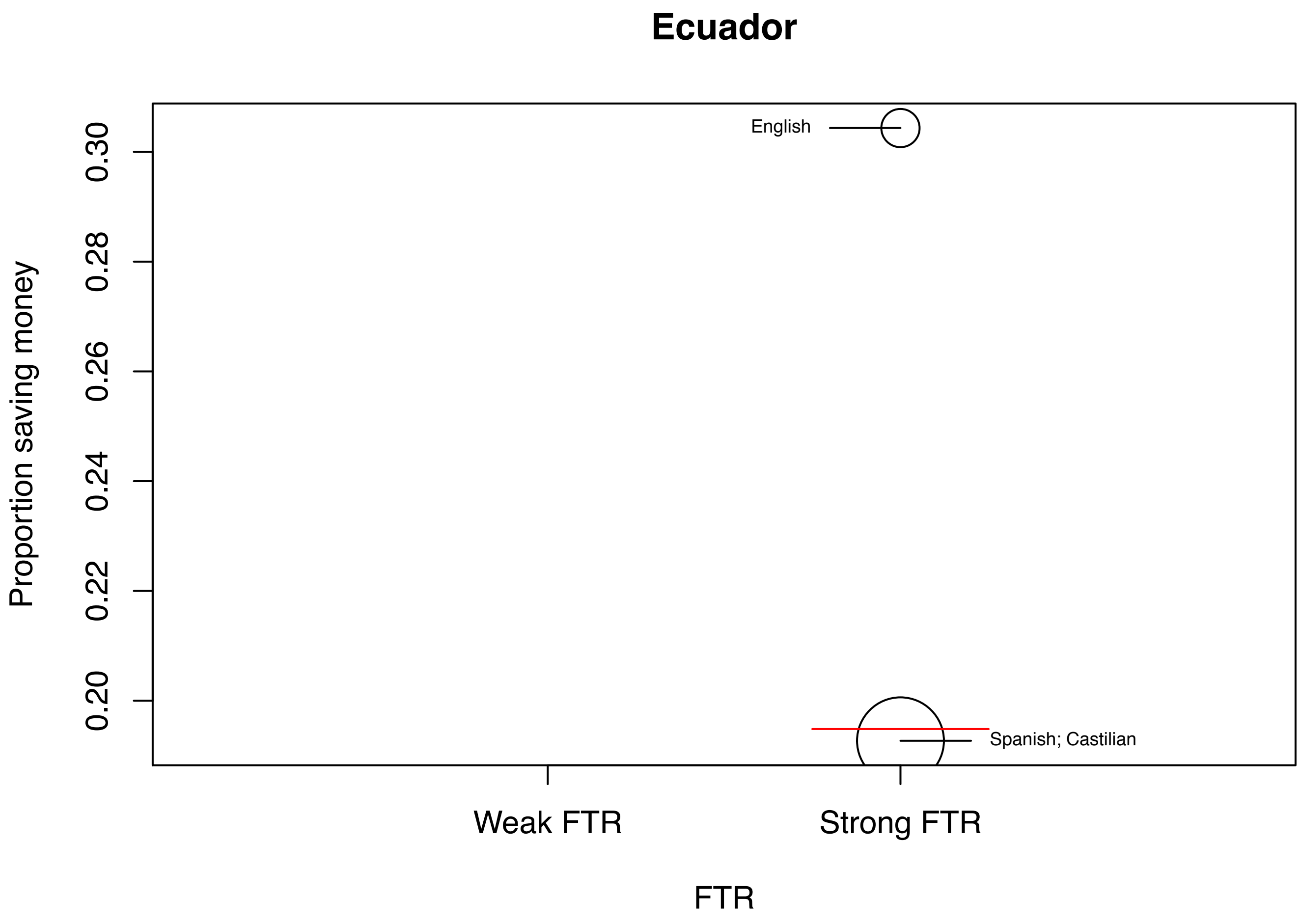
Ecuador
English
Spanish; Castilian
Proportion saving money
0.30
0.28
0.26
0.24
0.22
0.20
Weak FTR
Strong FTR
FTR

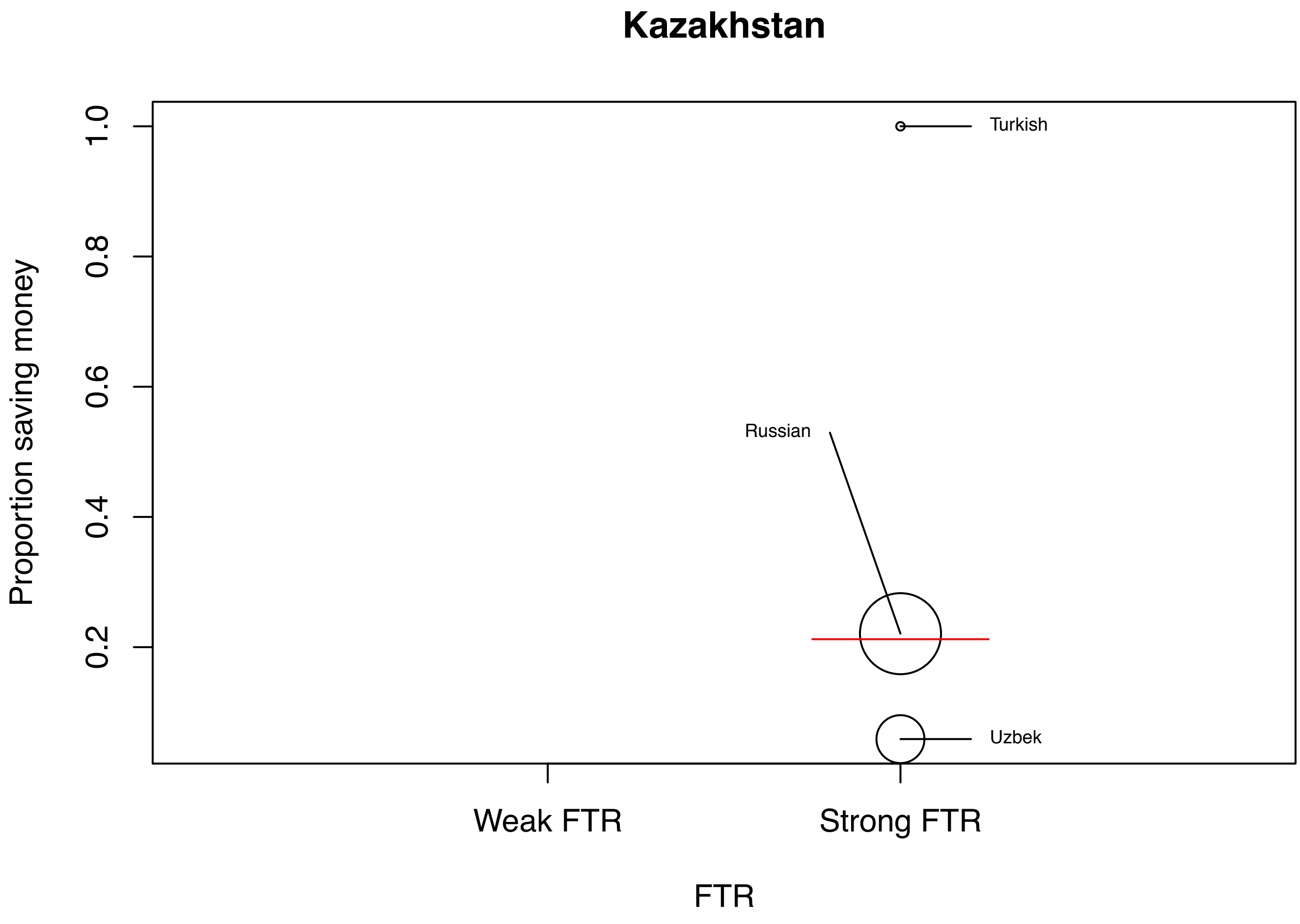
Kazakhstan
Turkish
Russian
Uzbek
Proportion saving money
1.0
0.8
0.6
0.4
0.2
Weak FTR
Strong FTR
FTR

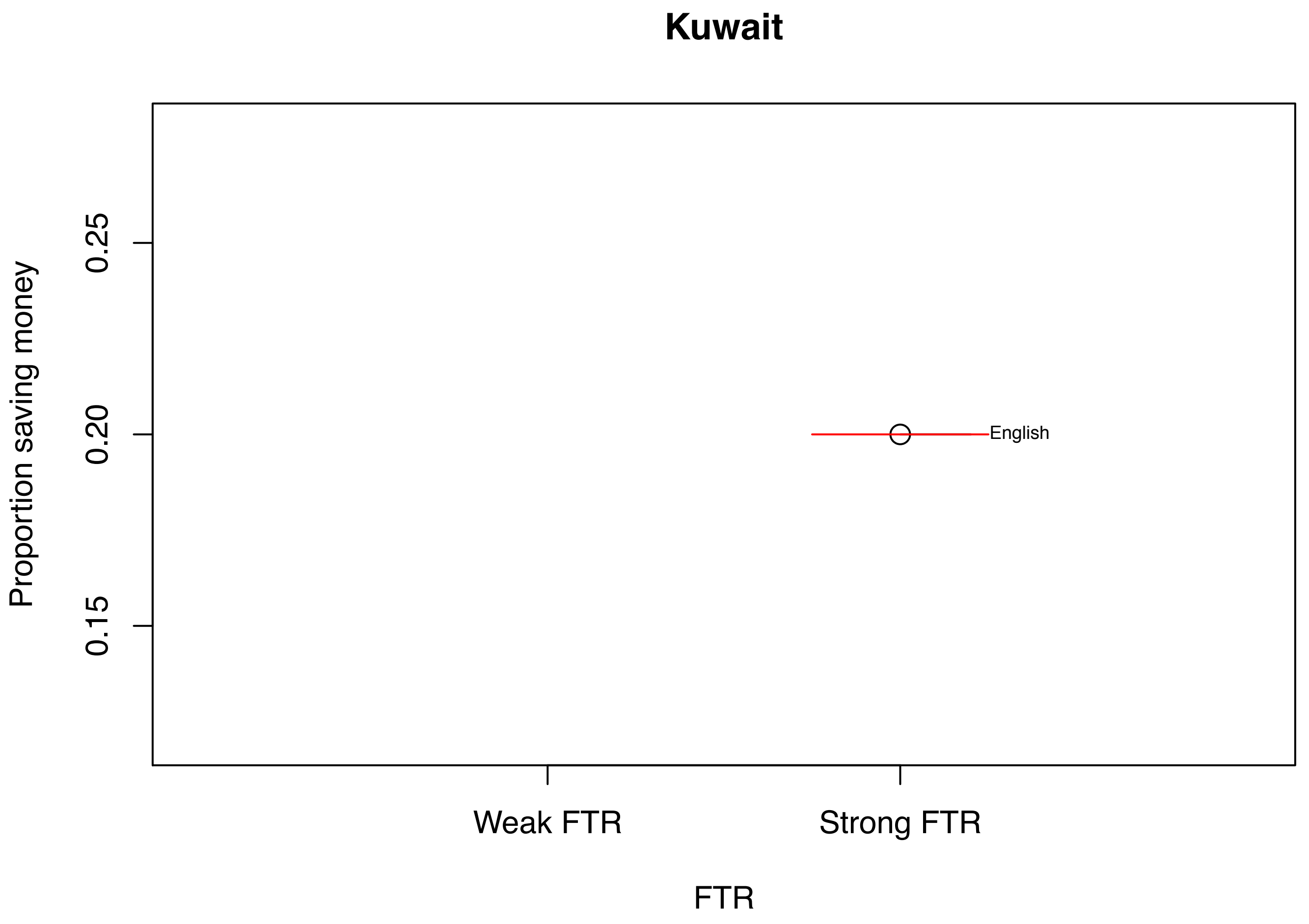
Kuwait
Proportion saving money
0.15
0.20
0.25
English
Weak FTR
Strong FTR
FTR

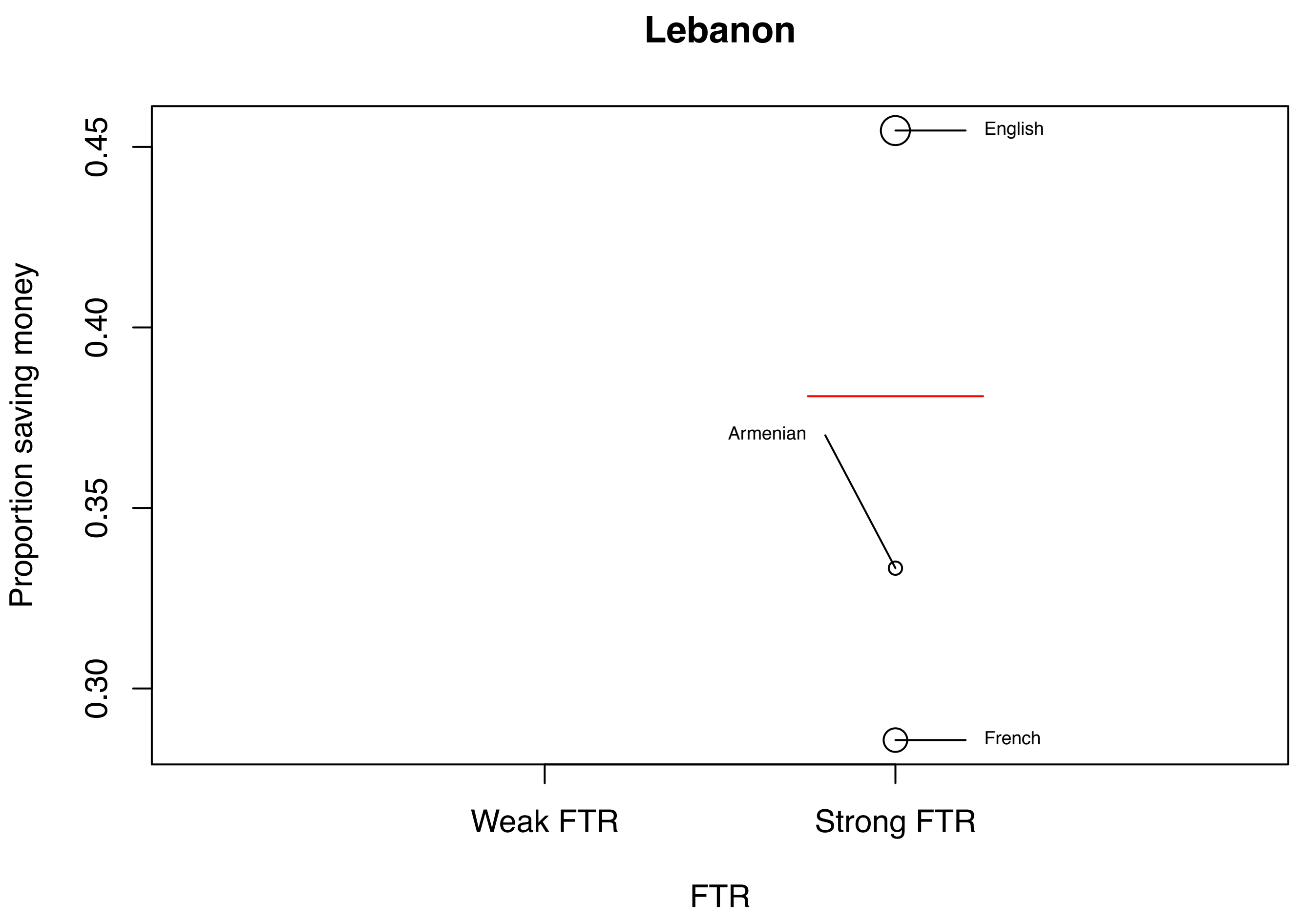
Lebanon
Proportion saving money
0.30
0.35
0.40
0.45
English
Armenian
French
Weak FTR
Strong FTR
FTR

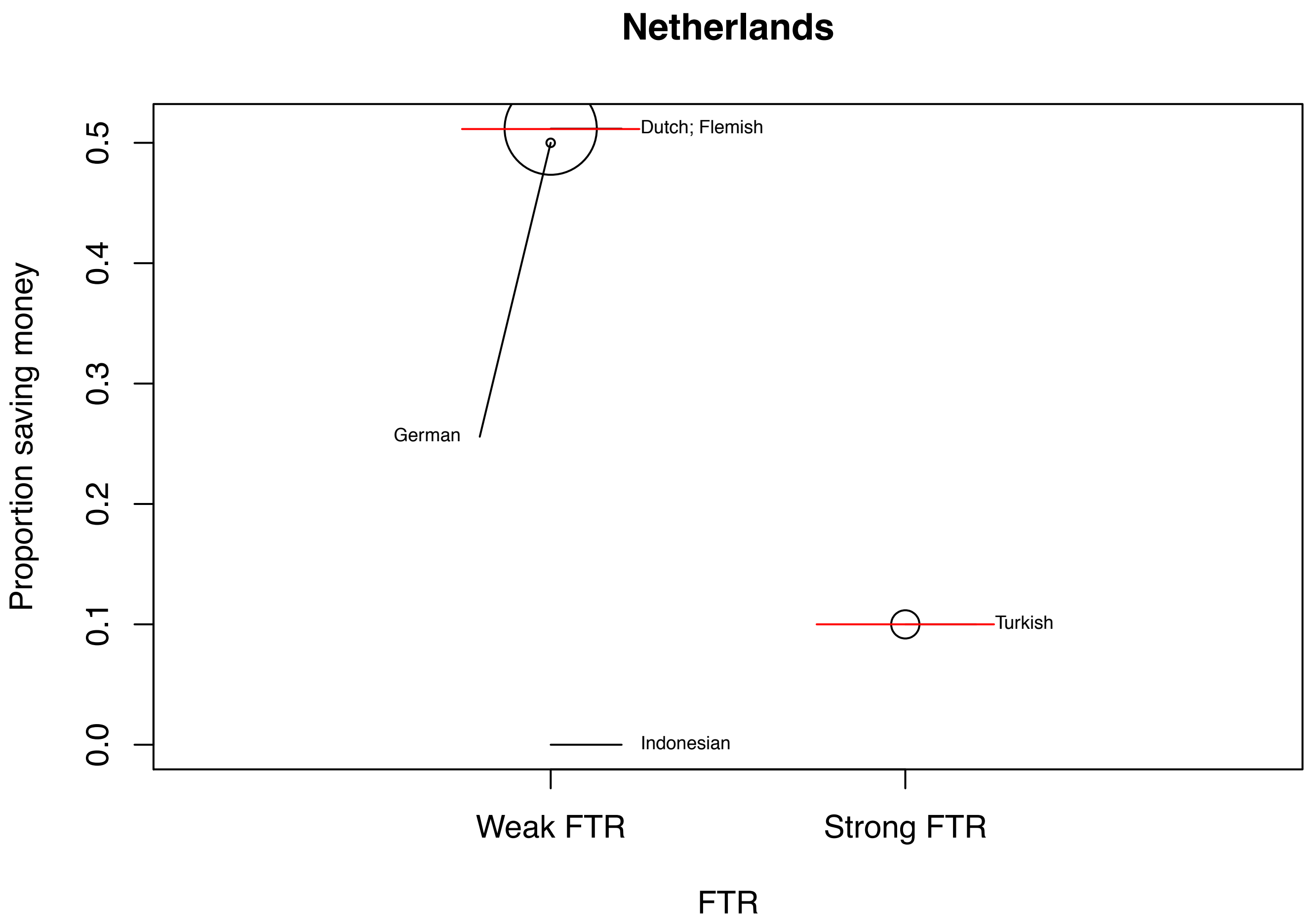
Netherlands
Dutch; Flemish
German
Turkish
Indonesian
0.0
0.1
0.2
0.3
0.4
0.5
Proportion saving money
Weak FTR
Strong FTR
FTR

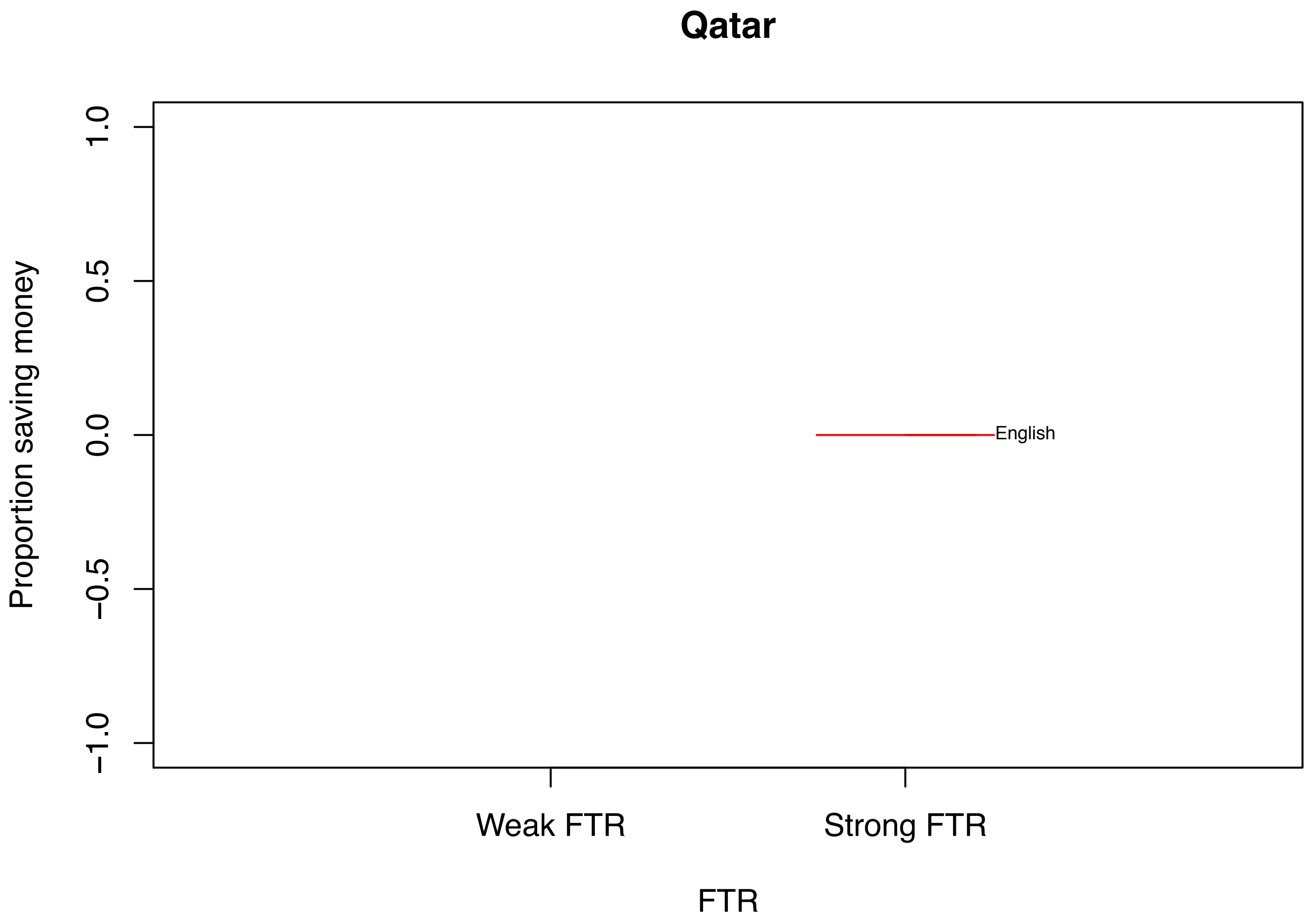
Qatar
English
−1.0
−0.5
0.0
0.5
1.0
Proportion saving money
Weak FTR
Strong FTR
FTR

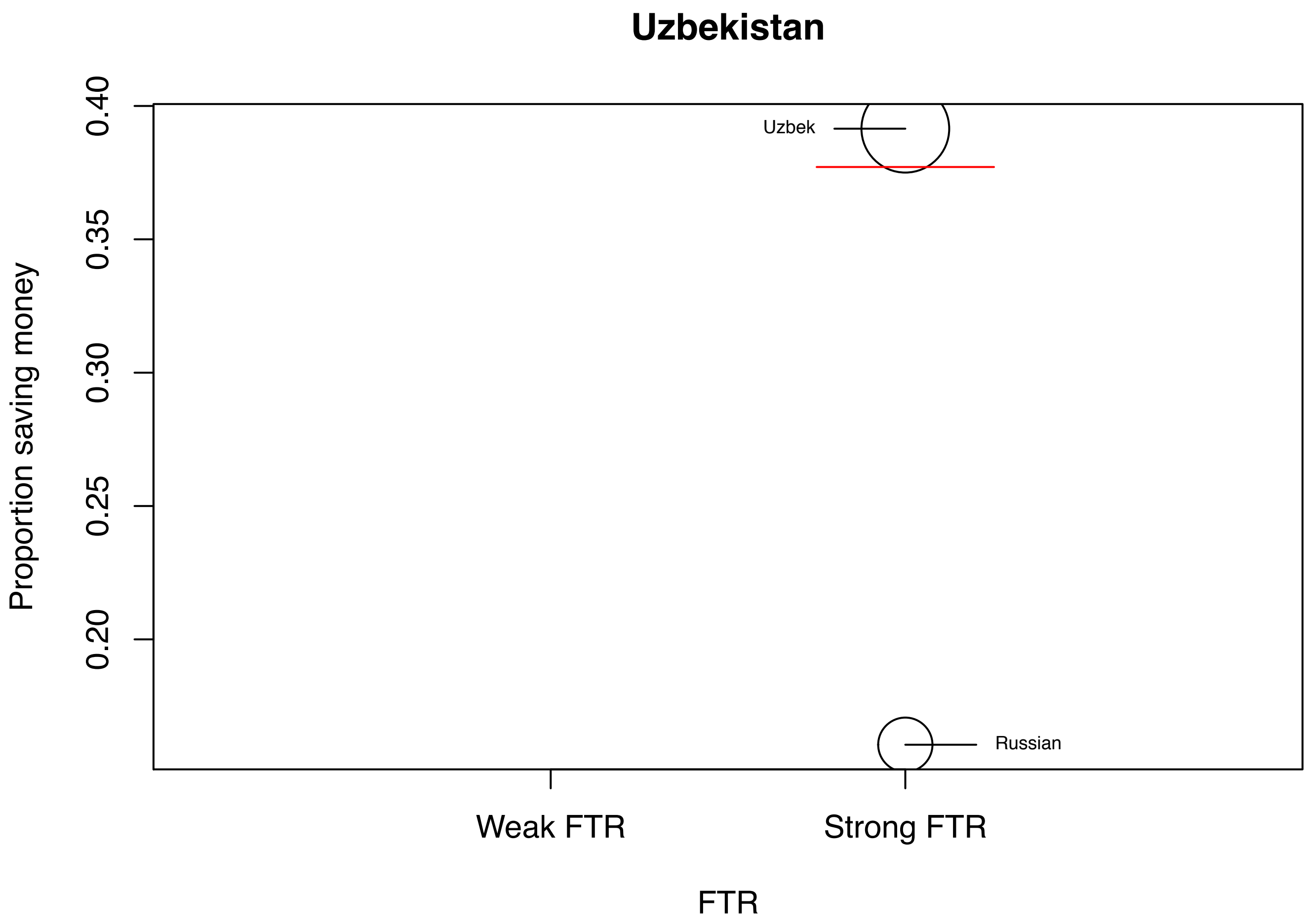
Uzbekistan
Proportion saving money
0.40
0.35
0.30
0.25
0.20
Uzbek
Russian
Weak FTR
Strong FTR
FTR

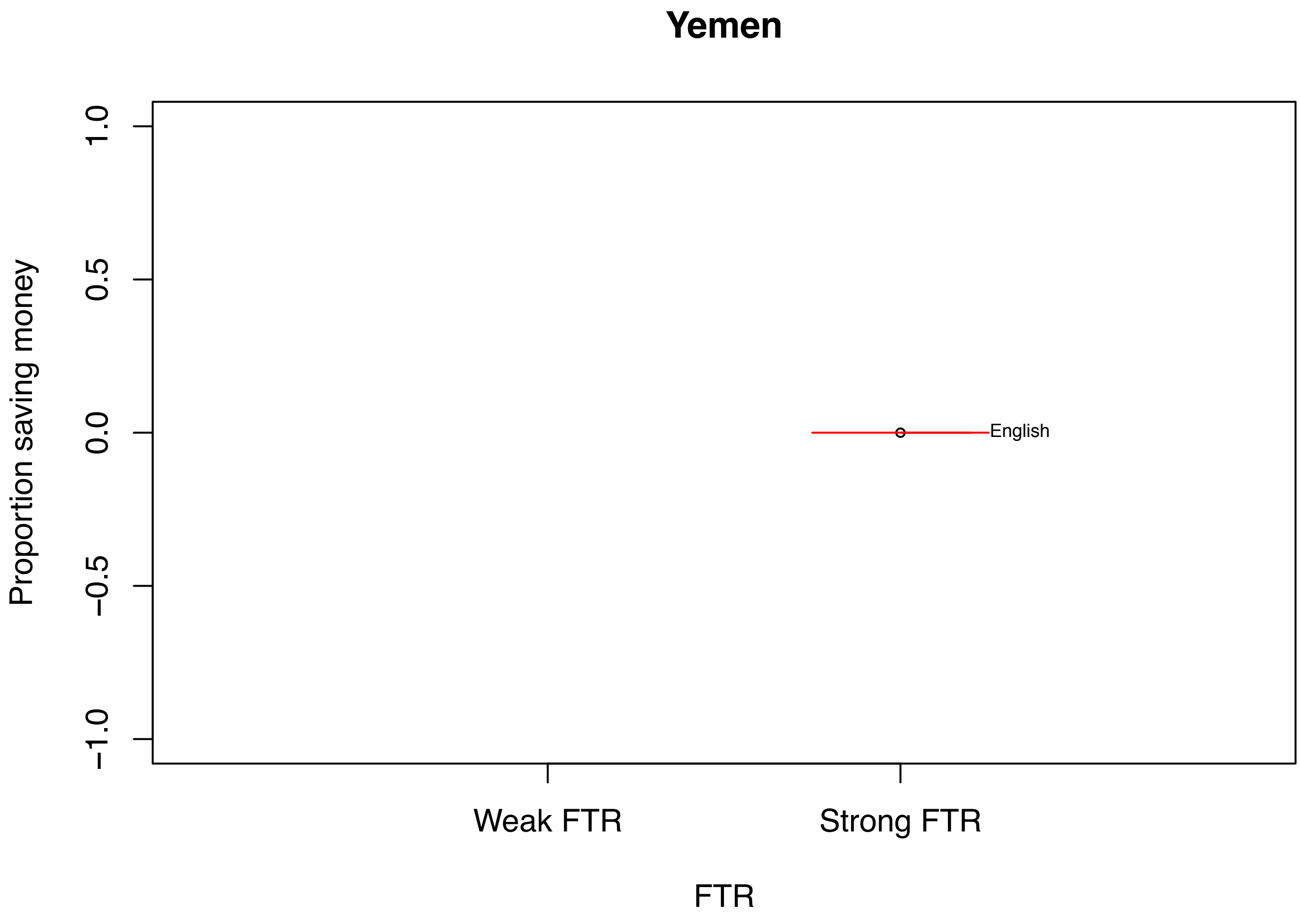
Yemen
Proportion saving money
1.0
0.5
0.0
−0.5
−1.0
English
Weak FTR
Strong FTR
FTR

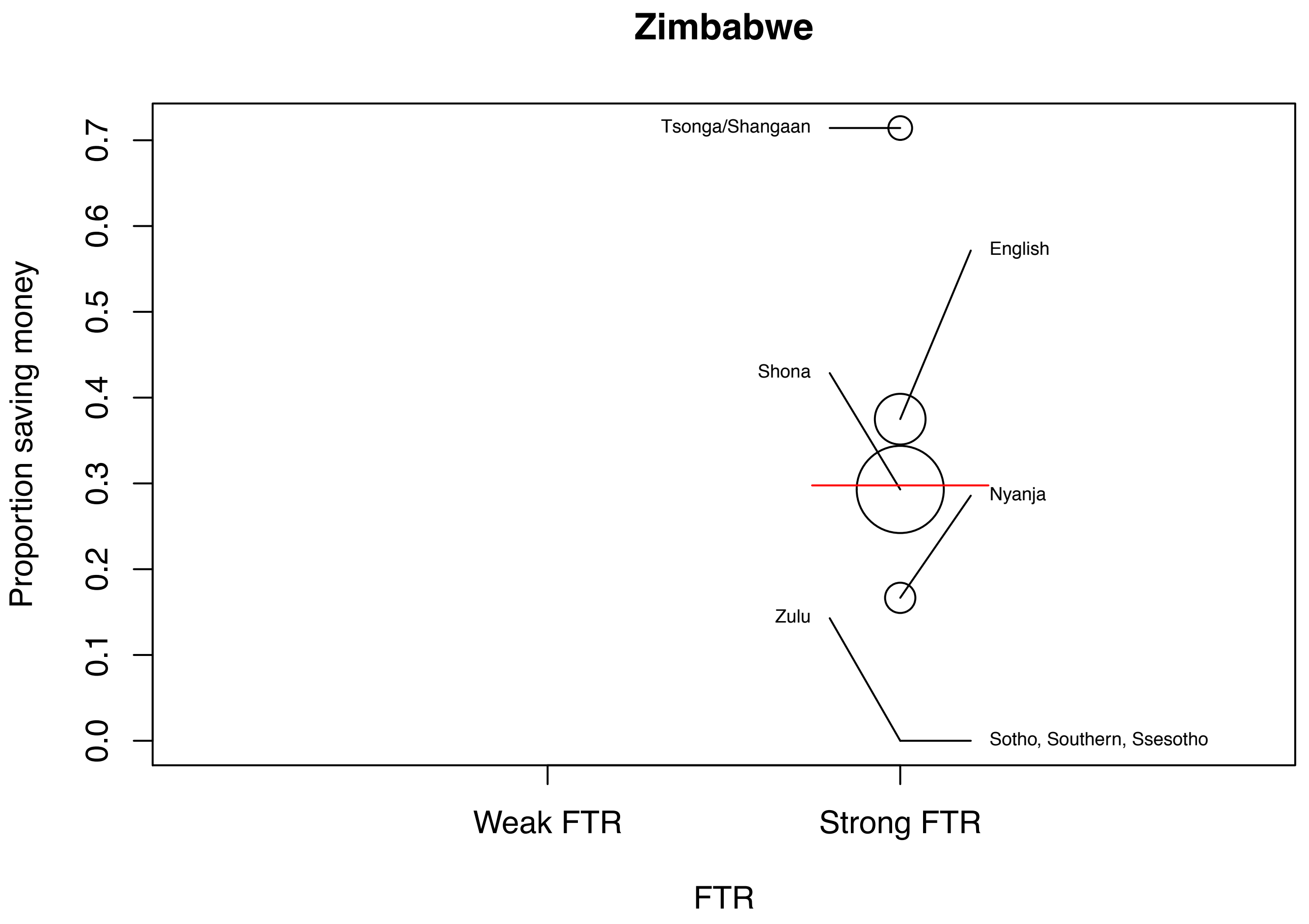

Zimbabwe
Proportion saving money
0.0
0.1
0.2
0.3
0.4
0.5
0.6
0.7
Tsonga/Shangaan
English
Shona
Nyanja
Zulu
Sotho, Southern, Ssesotho
Weak FTR
Strong FTR
FTR
