# Supplementary material for: Future Tense and Economic Decisions: Controlling for Cultural Evolution
Source: PLoS One. 2015 Jul 17;10(7):e0132145. doi: 10.1371/journal.pone.0132145 (PMC4506144; doi:10.1371/journal.pone.0132145)
Supplement: S11 Appendix — Figures illustrating the manipulations of the phylogenetic tree used in the robustness tests for the PGLS analyses. (PDF) [file pone.0132145.s011.pdf]

Future tense and saving:
PGLS tree manipulation details

This document provides some more detail on the tests of robustness for the PGLS results, including manipulating the assumptions about branch length and branch depth. The text is as in the main paper, but here we have added extra figures for illustration.

Branch length assumptions in PGLS

The phylogenetic trees used in the analysis above involved assumptions about the branch lengths (time depth) of the connections within and between language families. To test the dependence of the result on these assumptions, the same analysis was run with different assumptions about the time depth of the phylogenetic tree within and between language families. The time depth within language families was varied between 0 and 12,000 years (the main tree assumes 6,000 years) and the time depth between language families was varied between 0 and 80,000 years (the main tree assumes 60,000 years). Fig. 1 shows three manipulations of the tree with different values for within- and between-family time.

Fig. 2 shows how the significance of the correlation varies with different branch lengths. The correlation between FTR and savings remained significant at the 0.05 level for all branch length assumptions tested (all correlations were negative). The most significant results come from short within-family branch lengths. The between-family branch lengths have little impact on the results. This suggests that the results of the PGLS analysis are robust against branch length assumptions. However, we note that we are assuming fairly simple branch length manipulations. Further tests could be carried out by estimating branch lengths from lexical data or cognates, etc.

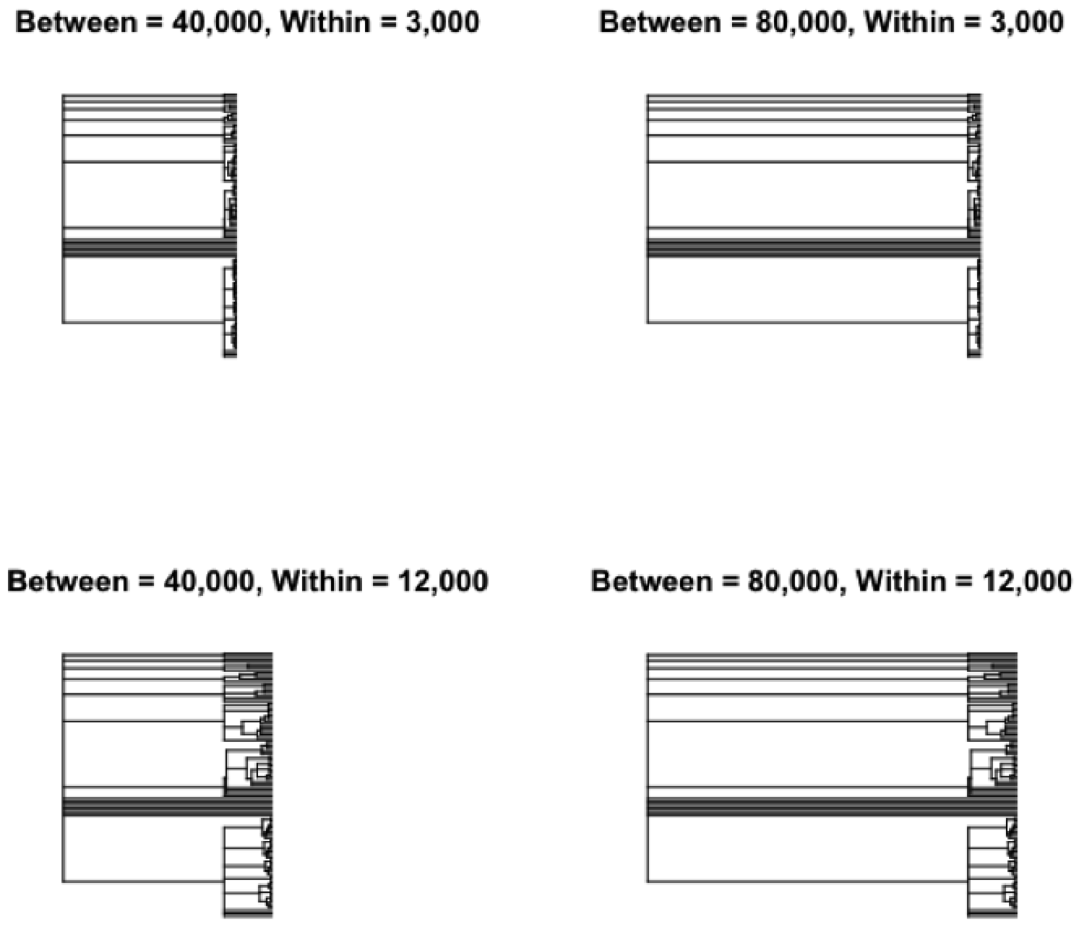

Figure 1: **Phylogenetic branch length manipulation.** Examples of the phylogenetic tree with different branch lengths between and within language families.

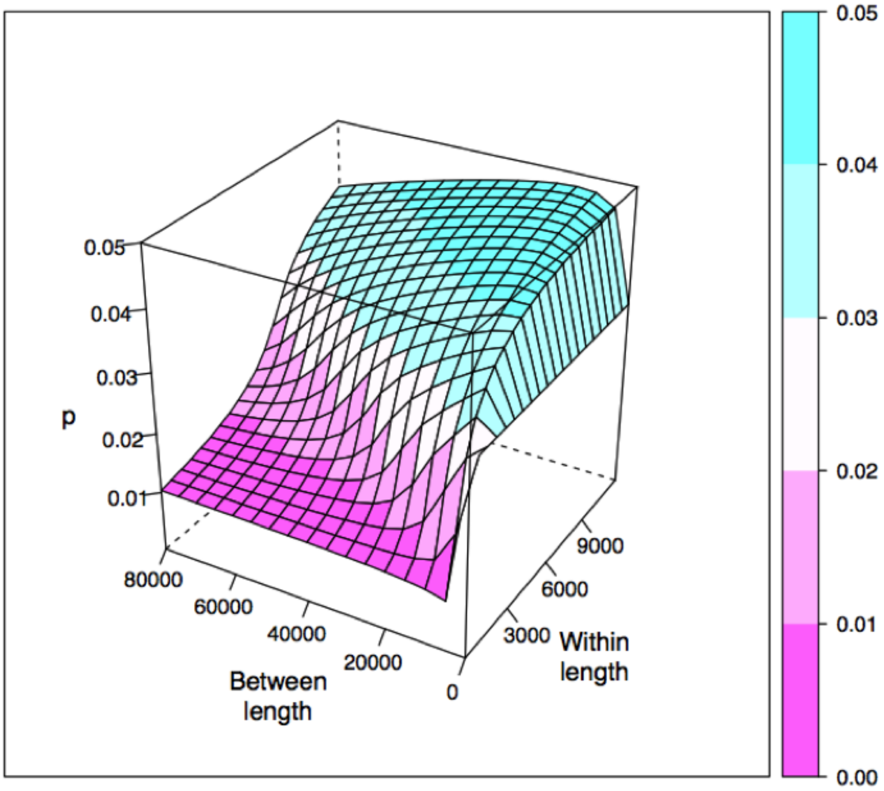

Figure 2: **How the probability of the correlation between FTR and savings (p, vertical axis) varies with different assumptions about between-family branch lengths and within-family branch lengths.** Lengths can be interpreted in years, though practically only the relative lengths matter.

1 Branch depth assumptions in PGLS

The analyses above assume that splits in the phylogenetic tree happen at particular interval, as well as assumptions about the overall time-depth. In order to test this assumption about intervals, the branch lengths of the phylogenetic tree was scaled according to Grafen's method. Internal nodes on the tree are assigned a height based on the number of descendants that node has. The heights are scaled so that the root height is 1, and then raised to the power ρ. Small values of ρ (<1) make the splits appear earlier in the tree and larger values of ρ make the splits appear later.

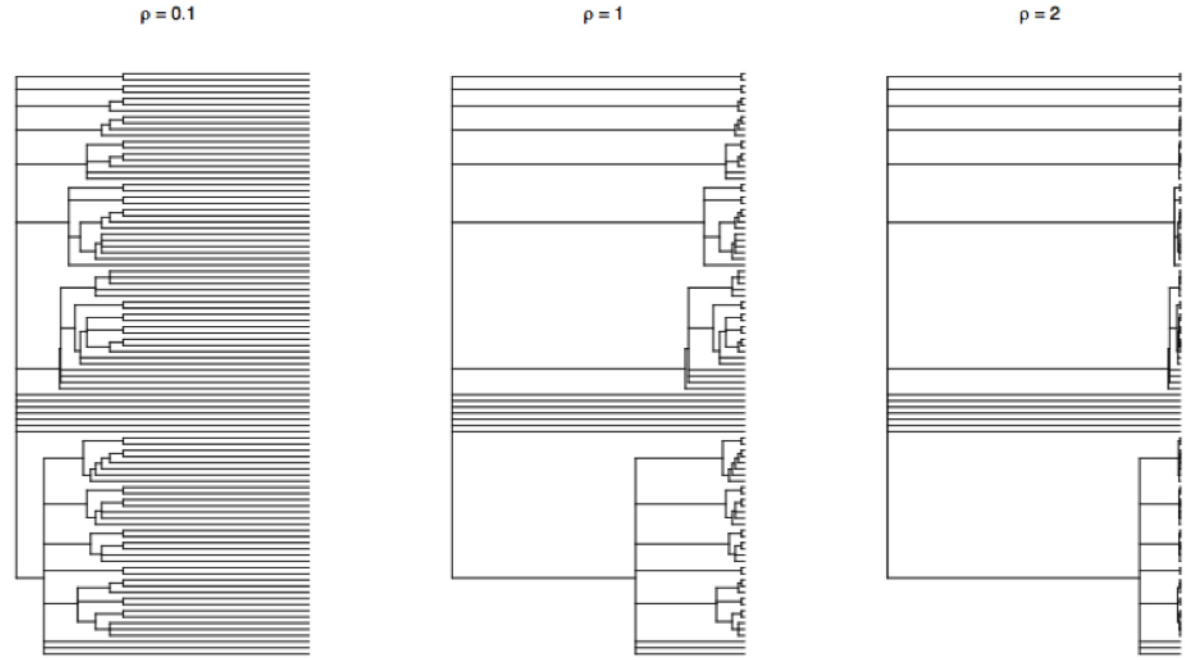

Figure 3: **Phylogenetic branch depth manipulation.** The phylogenetic tree with the branch lengths scaled according to Grafen's method with different values of the power variable. As the power increases, the splits in the tree become more recent.

Note that this method disrupts the distinctions between branch lengths within and between language families so that, for instance, language families with a larger number of languages tend to have common ancestors further back in time. In other words, this assumes a common rate of linguistic divergence for the whole tree, while the analyses above only make this assumption for the branches between language families.

The analysis above was run on trees using this method for a range of ρ values from 0.01 to 3. If we assume that the whole tree spans 60,000 years, when ρ is 0.01, 1 and 3, then 90% of the splits in the tree occur within the last 58,000, 16,600 and 350 years, respectively. Another way to think about this is that, when ρ is 0.01, 1 and 3, then the last divergence between two languages happened 57,000, 630, and 0.07 years ago. Clearly, $\rho = 0.01$ is too low and $\rho = 3$ is too high for a plausible estimate. Fig. 4 shows the results.

The fit of the model is best for values of ρ around 0.15 (best model: 90% of splits occur within the last 37,500 years, last split 30,351 years ago, log likelihood = -170.8; worst model: $\rho = 3$, 90% of splits occur within the last 350 years, last split 0.07 years ago, log likelihood = -177.9). For the best-fitting model, the correlation between FTR and savings behaviour is not significant (correlation coefficient = -0.713, t = -1.79, p = 0.076). The test is significant at the 0.05 level for values of ρ above 1. That is, the correlation between FTR and savings behaviour is only robust, given this tree topology, when the cultures we have data for diverge relatively recently (within the last 16,600 years). This is fairly plausible given that we don't have information on the phylogeny between language families. Put another way, the correlation is robust if we assume that the last divergence in languages happened less than 630 years ago. Given that the data includes Dutch and Afrikaans, which diverged in the 17th century Ponelis (1993), this seems like a reasonable assumption.

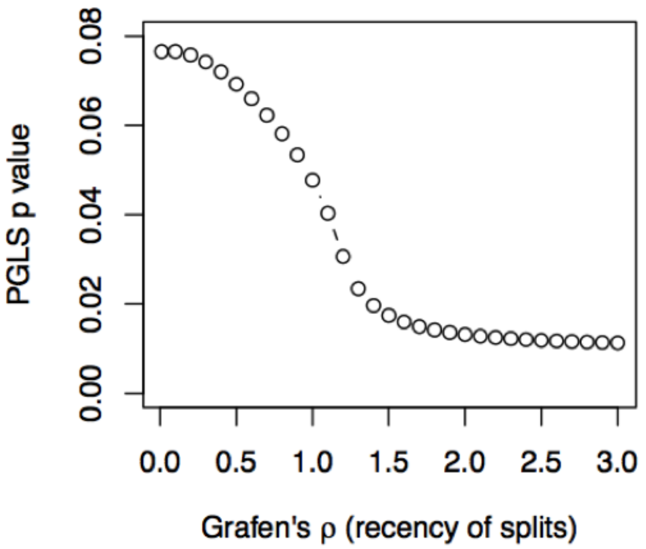

Figure 4: **Results for the phylogenetic generalised least squares test for different values of the branch length scaling power.**

PLGS for residuals from alternative regression

Since the repeated logits invert much more reliably with regression 9 than 11 (see section 'Regressions with language family fixed effects'), selected tests were run with the residuals generated from regression 9. There were no qualitative differences. The correlation between savings and FTR was negative and significant (Pagel's model -210.2021, FTR r= -1.529, t = -2.597 p = 0.01). The results were stronger, although the overall fit worse, for the Ornsten-Uhlenbeck model (log likelihood = -217.726, FTR r =-2.116, t= -3.710, p = 0.0004). Pagel's model resulted in a better fit than the Brownian motion model (Brownian motion log likelihood = -252.704, FTR r = 0.675, t = 1.006, p = 0.317; log likelihood difference = 42.51, L.ratio = 85.003, p < 0.0001). Manipulating the branch length assumptions, as above, did not result in p-values for Pagel's model above 0.033 (Fig. 5).

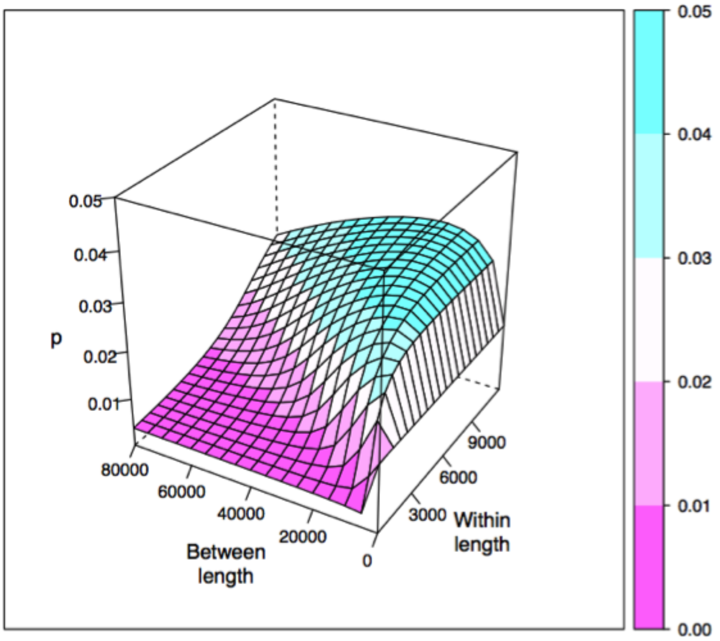

Figure 5: **Manipulating branch lengths for regression 3.** How the probability of the correlation between FTR and savings (p, vertical axis) varies with different assumptions about between-family branch lengths and within-family branch lengths, according to the PGLS regression.

References

Ponelis, F. A. (1993). *The development of Afrikaans*. Frankfurt am Main & New York: Peter Lang. [5]
