# Supplementary material for: Future Tense and Economic Decisions: Controlling for Cultural Evolution
Source: PLoS One. 2015 Jul 17;10(7):e0132145. doi: 10.1371/journal.pone.0132145 (PMC4506144; doi:10.1371/journal.pone.0132145)
Supplement: S8 Appendix — See README files within the various sub-folders. (ZIP) [file pone.0132145.s008.zip › S3_Code/Residuals_new/PhyloDist/AlgorithmTreeFromLabels/AlgTreeFromLabInstructions.pdf]

Hans-Jörg Bibiko

Program for creating nexus files from classificatory language labels (version 1.0) [2009]

Description

This program was developed by Bibiko on the basis of an algorithm devised by Søren Wichmann. It takes as input a set of classificatory labels and creates a file in a format which can be loaded into Splitstree (Huson and Bryant 2006)[1] such that the corresponding phylogeny can be viewed and analyzed.

For instance, the following 8 labels represent the classification in *Ethnologue* (Lewis 2009) of some Germanic languages (where some arbitrary abbreviations are used here to designate the different subgroups).

Afrikaans.InEu.Ger.W.AD.LoFr
Danish.InEu.Ger.N.AK.AL.AM.Dani
Dutch.InEu.Ger.W.AD.LoFr
English.InEu.Ger.W.Eng
Faroese.InEu.Ger.N.WSc
German.InEu.Ger.W.AD.LoSa
Icelandic.InEu.Ger.N.WSc
Swedish.InEu.Ger.N.AK.AL.Swe

The general format is: the language name followed by a dot followed by subgroup labels from the highest to the lowest level of classification, separated by dots.

The program outputs a file called "example.nex" in the standard input format for Splitstree. The example above yields the following output:

```
#nexus

BEGIN Taxa;
DIMENSIONS ntax=8;
TAXLABELS
[1]  'Afrikaans'
[2]  'Danish'
[3]  'Dutch'
[4]  'English'
[5]  'Faroese'
[6]  'German'
[7]  'Icelandic'
```

[1] Freely available at http://www.splitstree.org/.

```
[8]  'Swedish'
;
END;  [TAXA]

BEGIN Characters;
DIMENSIONS nchar=19;
FORMAT
        missing=?
        gap=-
        symbols="0 1"
        labels
        no transpose
        no interleave
;
MATRIX
'Afrikaans'      1000000010001000100
'Danish'         0100000001010010001
'Dutch'          0010000010001000100
'English'        0001000010000100000
'Faroese'        0000100001100000000
'German'         0000010010001001000
'Icelandic'      0000001001100000000
'Swedish'        0000000101010010010
;
END;  [Characters]
BEGIN st_Assumptions;
        chartransform=Hamming;
        disttransform=SplitDecomposition;
        splitstransform=EqualAngle;
        SplitsPostProcess filter=dimension value=4;
        autolayoutnodelabels;
END;  [st_Assumptions]
```

Loading this file into Splitstree allows the user to view the phylogeny implied by the labels, to export a description of the tree in Newick format, and to use other Splitstree functionalities.

For instance, a picture of the phylogeny implied by the labels above looks as follows.

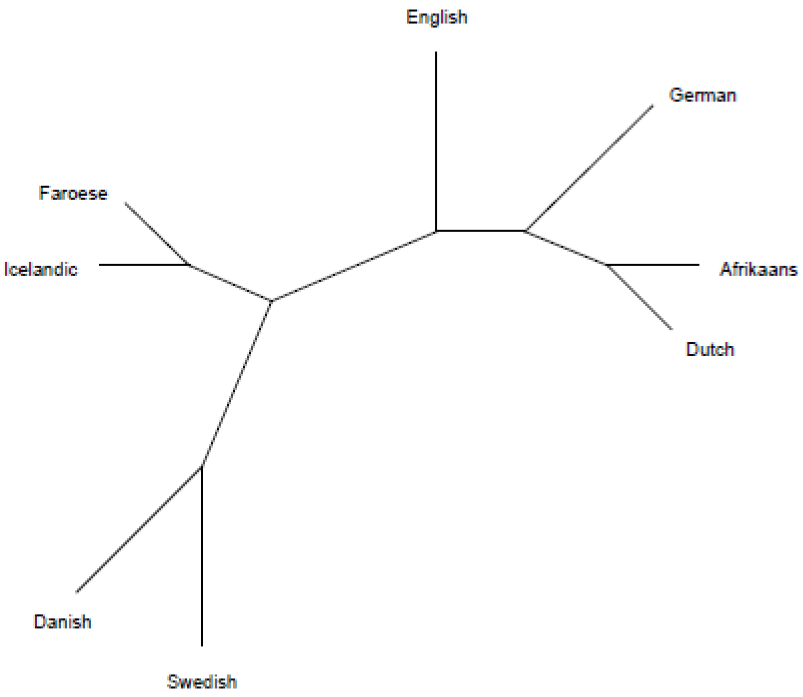

Instructions
The program runs under python 2.6 (but there are some problems with later versions). To run it, it is necessary to have python 2.6 installed. Python is preinstalled on Macs but PC users need to download it, for instance from

http://www.python.org/download/releases/2.6/.

Once you've installed python, you can invoke the program by going to the folder where python is installed (typically c:\python26) and write the following line in the dos command prompt:

```
python AlgorithmTreeFromLabels.py example.txt
```

Instead of "example.txt" some other filename can be used. The output is called "FILENAME.nex", where FILENAME is the name chosen.

The output can be loaded directly into Splitstree. When doing so, the phylogeny will be depicted. The tree can be exported by clicking File -> Export Image. The corresponding Newick file, giving a description of the tree in bracketed format, can be exported by clicking File -> Export -> Network -> NewickTree -> Apply. The example will look as follows.

((Dutch:0.05263158,(German:0.10526316,(English:0.10526316,((Icelandic:0.05263158, Faroese:0.05263158):0.05263158,(Swedish:0.10526316,Danish:0.10526316):0.10526316):0.10526316):0.05263158):0.05263158):0.02631579,Afrikaans:0.02631579):0;

This Newick description is useful, among other things, for comparing different phylogenies for a given set of taxa (for instance using treedist.exe by Joseph Felsenstein, see http://evolution.genetics.washington.edu/phylip.html).

References

Huson, D. H. and D. Bryant. 2006. Application of phylogenetic networks in evolutionary studies. *Mol. Biol. Evol.* 23(2): 254-267.

Lewis, M. P. (ed.). 2009. *Ethnologue*. 16th Edition. Dallas: SIL International. <www.ethnologue.com>.
