# Supplementary material for: Future Tense and Economic Decisions: Controlling for Cultural Evolution
Source: PLoS One. 2015 Jul 17;10(7):e0132145. doi: 10.1371/journal.pone.0132145 (PMC4506144; doi:10.1371/journal.pone.0132145)
Supplement: S8 Appendix — See README files within the various sub-folders. (ZIP) [file pone.0132145.s008.zip › S3_Code/Residuals_new/PhyloLogit/BranchLengthManip_Scale.pdf]

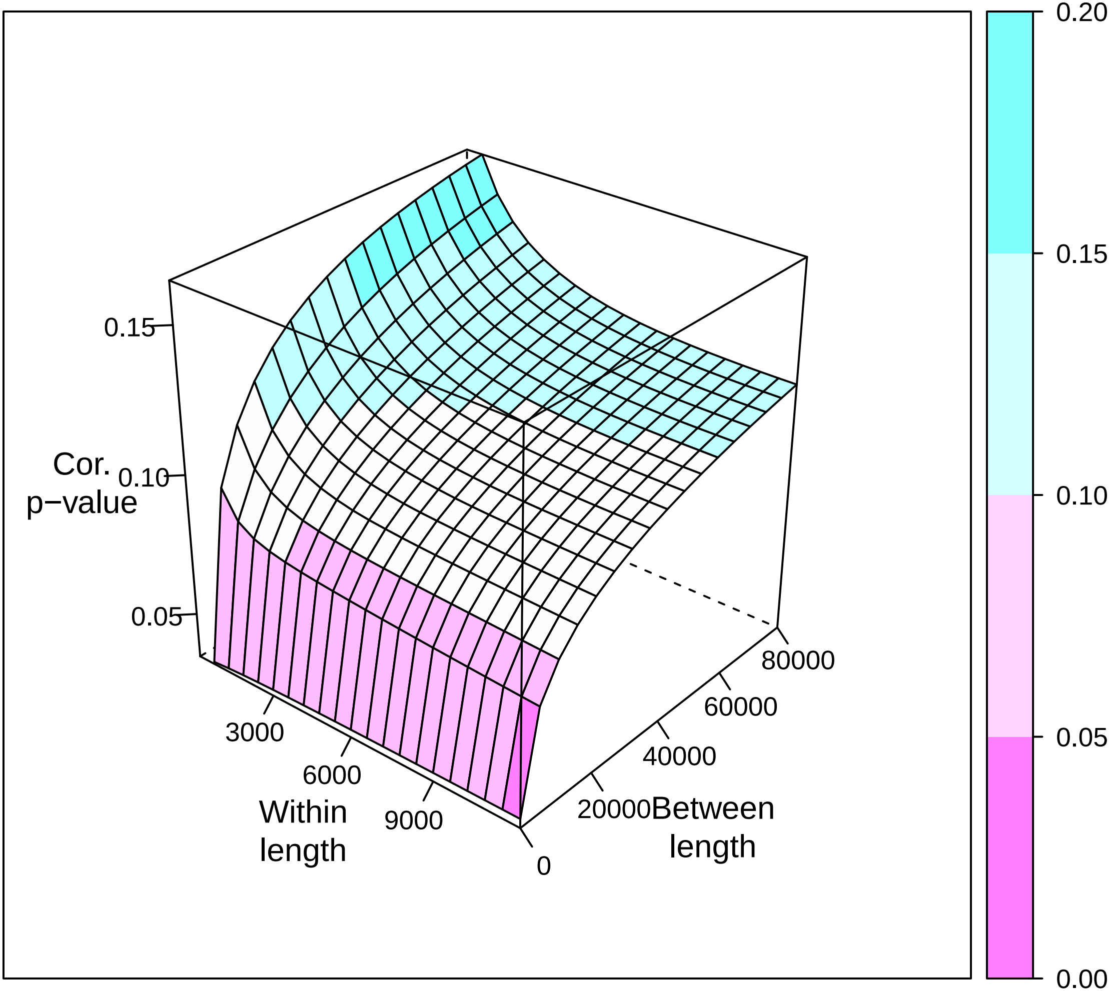

0.20
0.15
0.10
0.05
0.00
0.15
0.10
0.05
Cor.
p−value
3000
6000
9000
Within
length
0
20000
40000
60000
80000
Between
length
