# Supplementary material for: Future Tense and Economic Decisions: Controlling for Cultural Evolution
Source: PLoS One. 2015 Jul 17;10(7):e0132145. doi: 10.1371/journal.pone.0132145 (PMC4506144; doi:10.1371/journal.pone.0132145)
Supplement: S8 Appendix — See README files within the various sub-folders. (ZIP) [file pone.0132145.s008.zip › S3_Code/Residuals_new/PhyloLogit/RhoManip_Ethno.pdf]

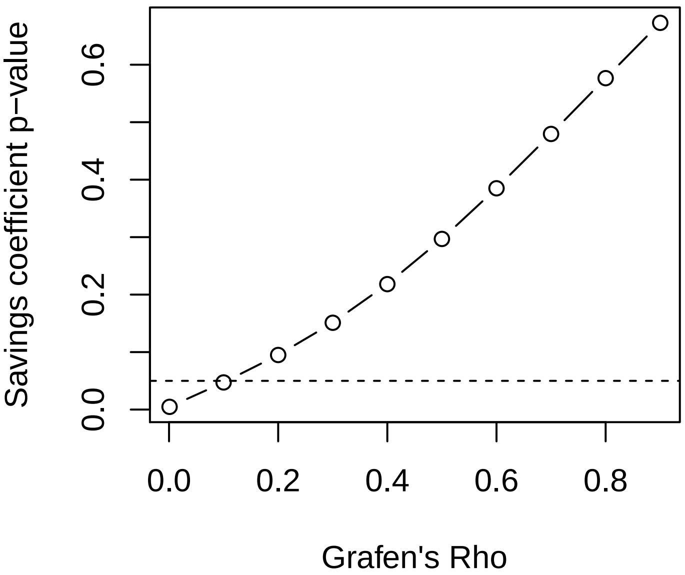
Savings coefficient p−value
0.0
0.2
0.4
0.6
0.0
0.2
0.4
0.6
0.8
Grafen's Rho
