# Supplementary material for: Future Tense and Economic Decisions: Controlling for Cultural Evolution
Source: PLoS One. 2015 Jul 17;10(7):e0132145. doi: 10.1371/journal.pone.0132145 (PMC4506144; doi:10.1371/journal.pone.0132145)
Supplement: S8 Appendix — See README files within the various sub-folders. (ZIP) [file pone.0132145.s008.zip › S3_Code/Residuals_new_Reg3/PGLS/BranchLengthManip_PGLS.pdf]

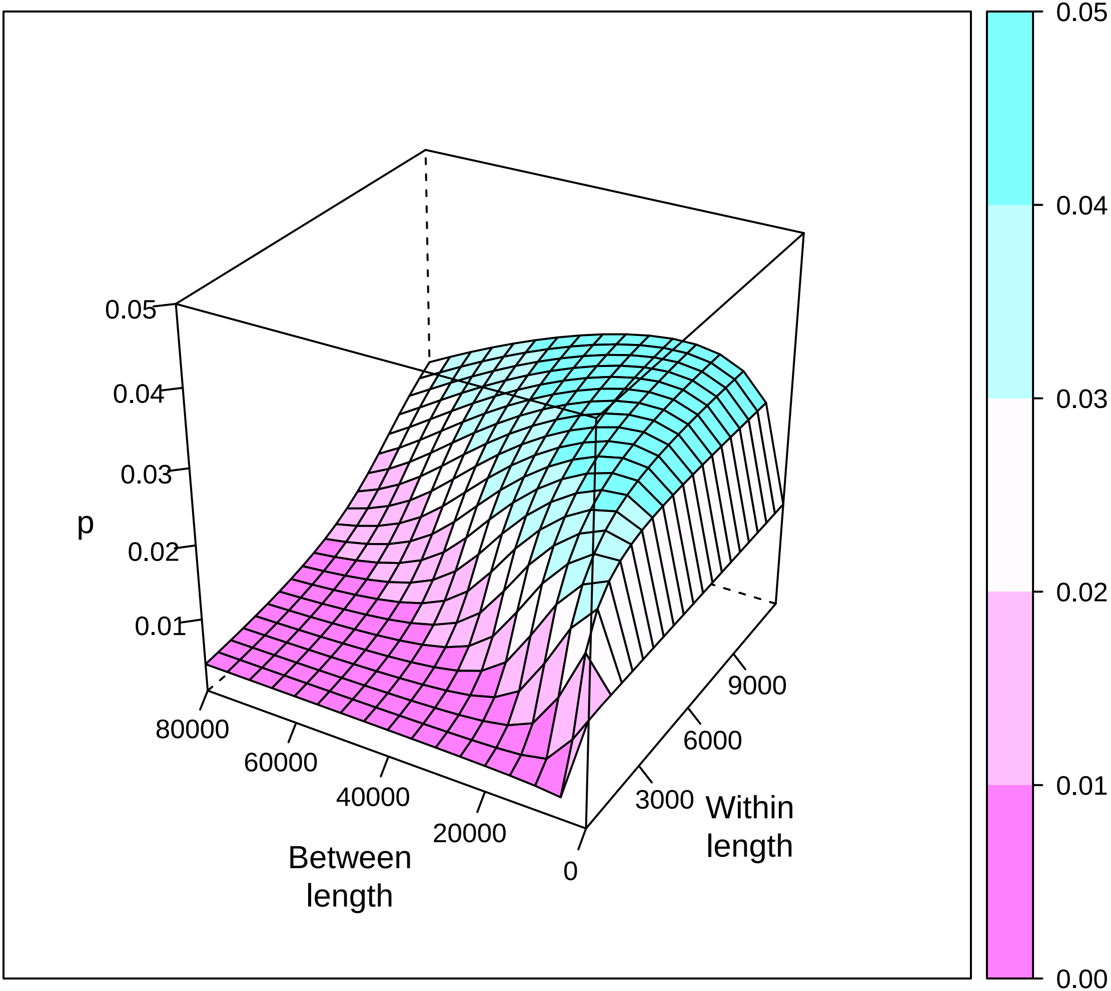

0.05
0.04
0.03
0.02
0.01
p
80000
60000
40000
20000
0
Between length
9000
6000
3000
Within length
0.05
0.04
0.03
0.02
0.01
0.00
